# CO_2_‐DBU‐Triggered Photoredox‐Catalyzed Direct α‐C‐H Alkylation of Alcohols

**DOI:** 10.1002/advs.202507490

**Published:** 2025-06-09

**Authors:** Zeyu Zhang, Zongchang Han, Yuhao Shang, Han‐Shi Hu, Jun Li, Chanjuan Xi

**Affiliations:** ^1^ Department of Chemistry MOE Key Laboratory of Bioorganic Phosphorus Chemistry & Chemical Biology Tsinghua University Beijing 100084 P. R. China; ^2^ Department of Chemistry and Engineering Research Center of Advanced Rare‐Earth Materials of Ministry of Education Tsinghua University Beijing 100084 P. R. China; ^3^ State Key Laboratory of Elemento‐Organic Chemistry Nankai University Tianjin 300071 P. R. China

**Keywords:** alcohols, carbon dioxide, C‐H alkylation, hydrogen‐atom transfer, photoredox catalysis

## Abstract

An efficient photoinduced and metal‐free method for direct α‐C–H monoalkylation of alcohols utilizing the CO_2_‐DBU‐system as a hydrogen bond acceptor (HBA) catalyst in the presence of water (H_2_O) is reported. This protocol allows for selective functionalization of alcohols with a broad substrate scope, demonstrating yields up to 88% in 36 examples. Systematic computational analysis using DFT calculations reveals insights into the mechanism by identifying the key intermediates assembled via intermolecular hydrogen bond between DBU‐CO_2_ adduct and alcohol. This strategy opens new avenues for efficient alkylation of ordinary alcohols, offering an environmentally friendly approach to complex molecular synthesis.

## Graphical abstract

An efficient photoinduced and metal‐free method is developed for direct α‐C–H monoalkylation of alcohols utilizing the CO_2_‐DBU system as the hydrogen bond acceptor (HBA) in the presence of H_2_O, allowing for selective functionalization of alcohols with a broad substrate scope.

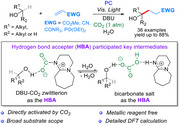

## 1 Introduction

**Scheme 1:**
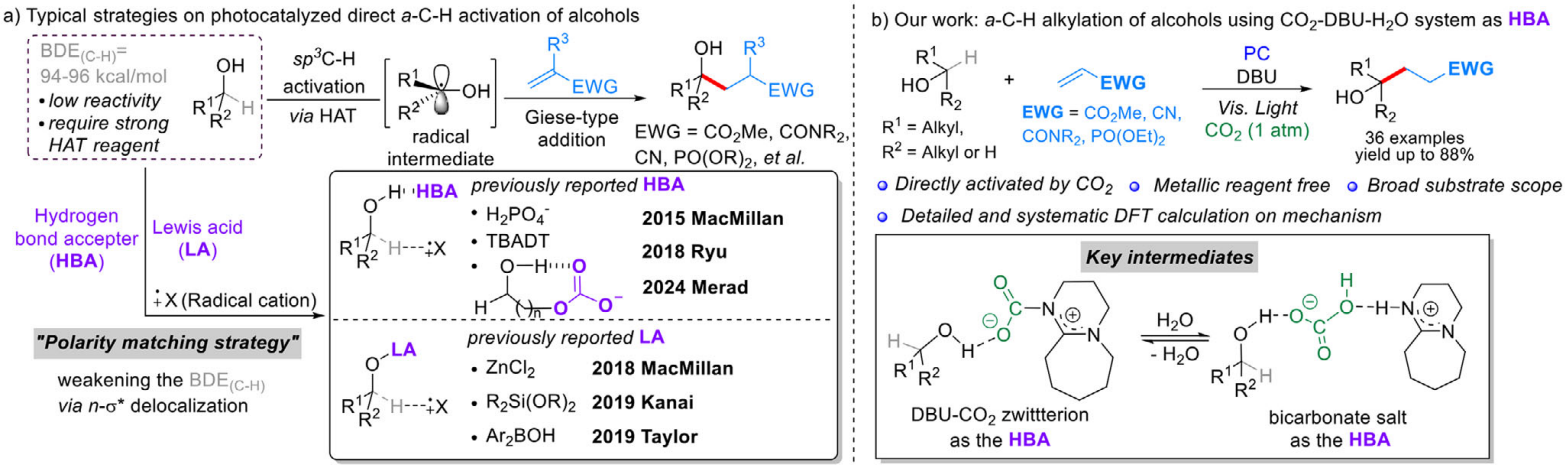
a) Typical approaches in α‐C‐H activation of Alcohols, b) Our work: α‐C‐H alkylation of alcohols using CO_2_‐DBU system as HBA.

The activation of C─H bonds represents one of the most valuable and challenging transformations in organic synthesis.^[^ ^1^ ^]^ Transition metal‐catalyzed selective activation of aromatic and alkene C─H bonds^[^ ^2^ ^]^ and direct functionalization of alkyl C─H bonds^[^ ^3^ ^]^ have already witnessed significant advancements over the past decades. For C(sp^3^)‐H activation reactions where selectivity is challenging to control,^[^ ^4^ ^]^ various catalytic systems based on directing groups (DG) have demonstrated remarkable effectiveness.^[^ ^5^ ^]^ Of all these substrates, alcohols are prevalent organic molecules, commonly found in natural substances such as sugars, steroids, and proteins, and widely utilized in synthetic pharmaceuticals.^[^ ^6^ ^]^ Despite their abundance, the activation of α‐C─H bonds (BDE = 94–96 kcal mol^−1^) remains a significant challenge.^[^ ^7^ ^]^ This difficulty arises from precise selective activation without affecting other functional groups in the same molecule. Obviously, direct α‐C‐H homolysis by strong HAT catalysts (BDE > 96 kcal mol^−1^) was bound to encounter limitations on both substrate scope and practical scalability (**Scheme** **1**a, top).^[^ ^8^ ^]^

As for the HAT process, it is acknowledged that the presence of polar functionalities can influence neighboring C─H bonds and their reactivity for alcohols, in addition to the relative stability of the generated radical.^[^ ^8^, ^9^ ^]^ As a result, the polarity match between the character of the C─H bond to be cleaved and the hydrogen abstractor could affect the difficulty of the HAT step.^[^ ^9^, ^10^ ^]^ With these considerations in mind, the “polarity matching strategy” becomes the key concept, which aims to weaken the bond dissociation energy (BDE) of the α‐C─H bond of alcohols by hyperconjugation *via n*‐*σ* ^*^ interactions.^[^ ^7^, ^11^ ^]^ In recent years, modern strategies have achieved remarkable progress through two complementary activation paradigms: hydrogen bond acceptors (HBAs)^[^ ^11^, ^12^ ^]^ and Lewis acids (LAs)^[^ ^7^, ^13^ ^]^ catalysis specifically in photoredox fashion. In 2015, the Macmillan group reported the photoredox α‐alkylation of alcohols with methyl acrylate catalyzed by *tetra*‐*n*‐butylammonium phosphate as the HBA.^[^ ^11c^ ^]^ Subsequently, similar pathways were accomplished by the Ryu group with tetrabutylammonium decatungstate (TBADT) in 2018.^[^ ^12b^ ^]^ Very recently, the Merad group presented a photoinduced selective α‐C‐H monoalkylation of symmetric polyols in the presence of CO_2_ aided by intramolecular hydrogen bonds, while this system was limited to symmetric diols.^[^ ^12d^ ^]^ As for the LA systems, previous reports employed ZnCl_2_,^[^ ^13b^ ^]^ R_2_Si(OR)_2_^[^ ^7b^ ^]^ or Ar_2_BOH^[^ ^13d^ ^]^ to achieve C‐H polarization through hydroxyl coordination, generating transient alkyl radicals for downstream transformations (Scheme 1a, bottom). However, the utilization of strong Lewis acid limits the functional group compatibility. Therefore, developing a metal‐free, environmentally friendly, and broadly applicable strategy remains a significant challenge.

Our research group has been dedicated to CO_2_‐promoted functional group transformations of alcohols and has developed a C─O bond cleavage reaction system via transition metal catalysis.^[^ ^14^ ^]^ Consequently, the more challenging α‐C–H activation of alcohols promoted by CO_2_ has become our primary focus. Interestingly, although the carbonic esters formed from CO_2_ and alcohols facilitate the oxidative addition process, their formation inhibits α‐C–H activation.^[^ ^12d^ ^]^ Inspired by these findings, we envision a catalytic system that harnesses CO_2_'s dual role as a dynamic activator and selective modulator. Herein, we disclose a catalytic platform triggered by CO_2_ and 1,8‐diazabicyclo[5.4.0]undec‐7‐ene (DBU), combined with a photoinduced HAT process (Scheme 1b). Through the reversible reaction between CO₂ and DBU to form a zwitterion, a newly designed HBA reagent efficiently assists in the direct α‐C–H monoalkylation of alcohols. Systematic DFT calculations identify the most favorable transition states, thereby providing a precise catalytic cycle for this reaction.

## 2 Results and Discussion

### 2.1 Reaction Conditions Optimization

We began our investigations by studying the interaction between phenylethanol **1a** and methyl acrylate **2a** under photoredox and CO_2_‐DBU assisted system as a model reaction for optimal reactions (**Table** **1**; Tables S1–S9, Supporting Information for more details). After a series of preliminary experiments, we successfully detected substituted 1,4‐butyrolactone **3a** in 71% ^1^H NMR yield. Notably, 1,4‐butyrolactone **3a** was observed as an exclusive product, which is attributed to the rapid and thermodynamically favored intramolecular lactonization. The optimal reaction condition was identified as 1 mol% Ir[dF(CF_3_)ppy]_2_(dtbbpy)PF_6_ **(Ir‐1)** as photocatalyst (PC), 20 mol% quinuclidine as the HAT reagent, and 50 mol% DBU as the base under a CO_2_ atmosphere (1 atm, bubbled for 2 min) in a mixture of CH_3_CN and H_2_O (v/v = 20:1) at 40 °C under 15 W blue LEDs irradiation for 12 h (entry 1). As another typical HAT reagent together with Ir photocatalysts, DABCO did not perform well in this system (entry 2).^[^ ^8^, ^15^ ^]^ The selection of the photocatalysts was crucial, as replacing **Ir‐1** with other catalysts such as Ir[dF(Me)ppy]_2_(dtbbpy)PF_6_ (**Ir‐2**) or 2,4,6‐tris(diphenylamino)‐5‐fluoroisophthalonitrile (3DPAFIPN) resulted in significantly lower yields, while Ru(bpy)_3_(PF_6_)_2_ (**Ru**‐**1**) failed to yield any product (entry 3–5). Replacing DBU with 1,1,3,3‐tetramethylguanidine (TMG) or 1,3‐bis(2,4,6‐trimethylphenyl)imidazol‐2‐ylidene chloride (IMesCl) led to diminished yields (entry 6–7). Although bases with strong nucleophilicity can all form adducts with CO_2_, we suppose that the alkalinity distinctions and different stabilities of these base‐CO_2_ adducts in the reaction system lead to varying outcomes.^[^ ^16^ ^]^ When alcohol **1a** was reduced to 1.0 equivalence, the yield dropped sharply to 25% due to the formation of hydrocarbonate, which proved to be inert to the α‐H HAT step (entry 8). The inhibitor in **2a** such as hydroquinone methylether (MEHQ) will suppress the activity of the Giese addition (entry 9).^[^ ^17^ ^]^ Variations in temperature or solvents led to a diminished yield (entry 10−11). H_2_O was vital for high yields (entry 12), as it may act as the sacrificial agent and facilitate proton transfer via a hydrogen bonding network. The details will be discussed in the DFT calculations and mechanism sections. Additionally, the reaction cannot occur without PC, DBU, CO_2_, or light (entries 13−16), and the experiment in which CO_2_ was not bubbled also confirmed its necessity (entry 17).

**Table 1:** Optimization of the Reaction Conditions.

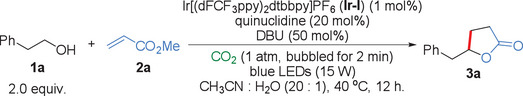		
Entry^a)^	Deviation from standard conditions	Yield of 3a [%]^b)^
1	none	71
2	DABCO instead of quinuclidine	15
3	Ir‐2 instead of Ir‐1	37
4	3DPAFIPN instead of Ir‐1	14
5	Ru‐1 instead of Ir‐1	0
6	TMG instead of DBU	38
7	IMesCl instead of DBU	0
8	1.0 equiv. alcohol	25
9	2a with 10 ppm MEHQ	65
10	DMF as solvent	27
11	20 °C instead of 40 °C	35
12	no H_2_O	11
13	no photocatalyst	0
14	no light	0
15	no DBU	Trace
16	no CO_2_	5
17	Without CO_2_ bubbling	57
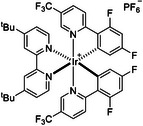 Ir[dF(CF_3_)ppy]_2_(dtbbpy)PF_6_ (**Ir** ‐ **1**)	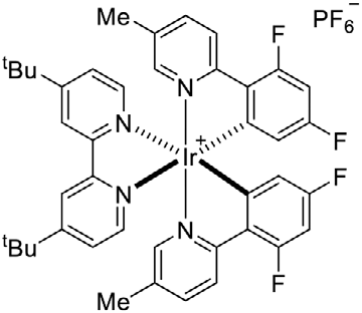 Ir[dF(Me)ppy]_2_(dtbbpy)PF_6_ (**Ir** ‐ **2**)	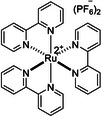 Ru(bpy)_3_(PF6)_2_ (**Ru** ‐ **1**)
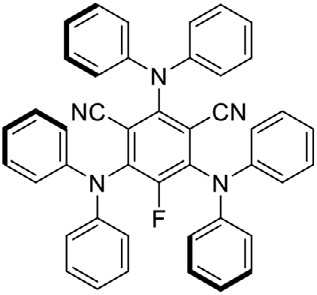 3DPAFIPN	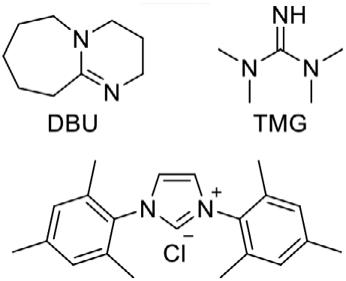 IMesCl	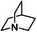 quinuclidine 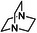 DABCO

^a)^ Reaction conditions: **1a** (0.6 mmol), **2a** (0.3 mmol), quinuclidine (20 mol%), DBU (50 mol%), Ir[(dFCF_3_ppy)_2_dtbbpy]PF_6_ (1 mol%) in 1 mL CH_3_CN and 50 µL H_2_O at 40 °C for 12 h under 15 w blue LEDs in CO_2_ atmosphere (1 atm) unless otherwise stated;

^b)^ Yields of product **3a** determined by crude ^1^H NMR, CH_2_Br_2_ as internal standard.

### 2.2 Substrates Scope

**Scheme 2:**
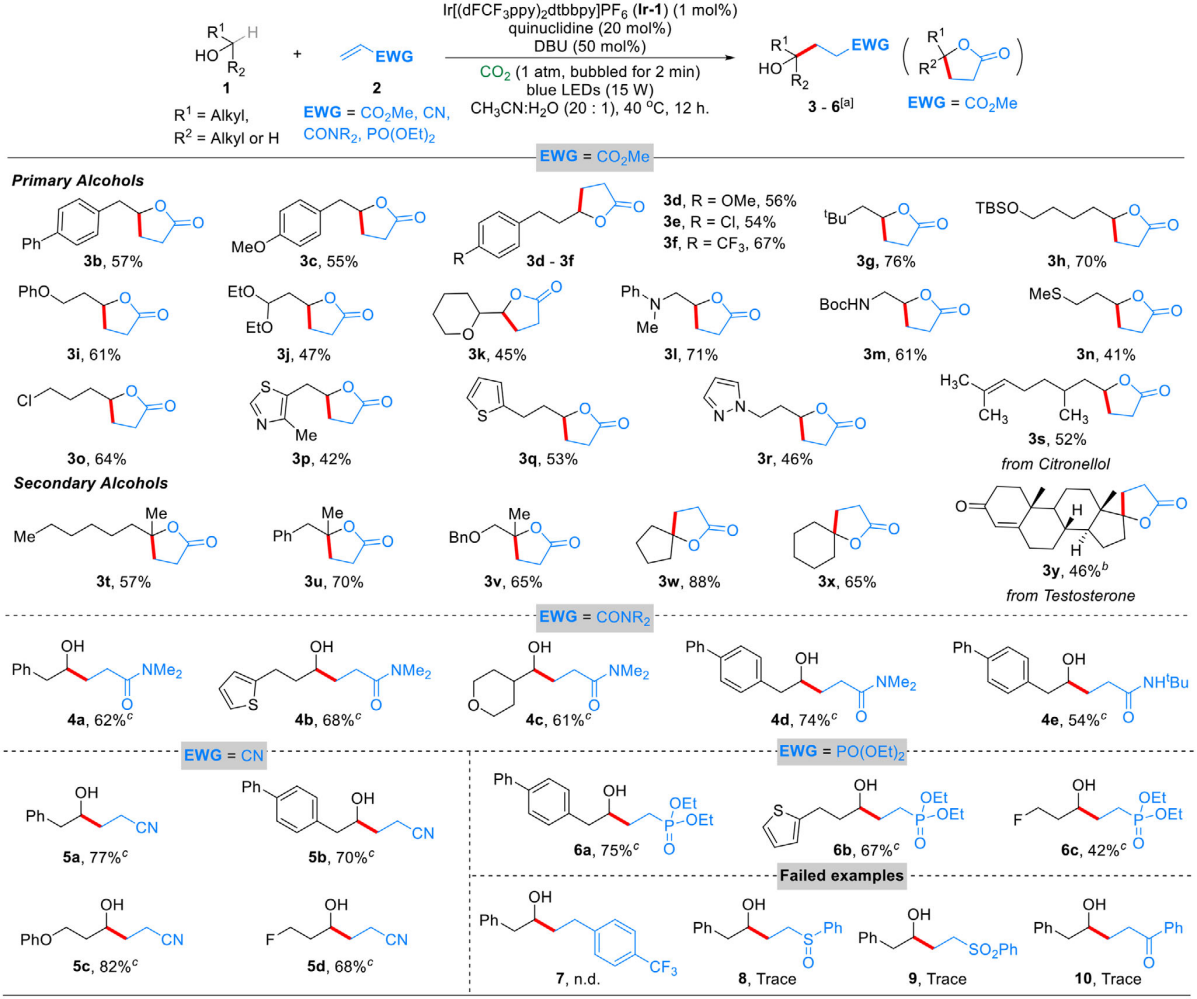
Substrates scope. [a] Reaction conditions: **1** (0.6 mmol), **2** (0.3 mmol), quinuclidine (20 mol%), DBU (50 mol%), Ir[(dFCF_3_ppy)_2_dtbbpy]PF_6_ (1 mol%) in 1 mL CH_3_CN and 50 µL H_2_O at 40 °C for 12 h under 15 w blue LEDs in CO_2_ atmosphere (1 atm) unless otherwise stated. Isolated Yield. [b] 2.5 equiv. of alcohol, reaction time extended to 24 h. [c] 1.5 equiv. of alcohols instead of 2.0 equiv.

The photoredox and DBU‐CO_2_ adducts assisted α‐C–H alkylation of alcohols was systematically explored across a wide range of substrates under the optimized reaction conditions, demonstrating excellent versatility and functional group tolerance (**Scheme** **2**). It turned out that the reactions worked smoothly for substituted primary alcohols. For alcohols with aromatic rings bearing different groups, products **3b–3f** could be isolated in 55%–67% yield. The reaction is insensitive to steric hindrance, as **3g** was also successfully obtained in 76% yield. Notably, the C─H bonds at the α‐position of other oxygen atoms in the alcohols did not undergo C‐H cleavage, and products **3h–3k** were obtained in moderate to high yields with excellent chemoselectivity. *N*‐substituted amines were also well tolerated, giving **3l** and **3m** in 71% and 61% yield, respectively. The methylthio‐substituted product **3n** was synthesized in 41% yield in spite of the reducing properties of the low‐valent sulfur atom. Chlorine atom could also be tolerated in product **3o**, which facilitates subsequent functional group modifications. A series of heterocyclic compounds were also suitable for this catalytic process, including thiazole (for **3p**), thiophene (for **3q**), and imidazole (for **3r**). For the substrates with moderate yields (such as **3j**, **3k**, **3n**, **3p,** and **3r**), the structures all contain heteroatomic groups (such as alkoxy, methylthio, thiazole, and imidazole). The presence of these functional groups may weaken the hydrogen bond combination between the substrate alcohol and the DBU‐CO_2_ adduct, thereby reducing the efficiency of the reaction. We also accomplished the modification of the natural product citronellol, which contains a double bond, achieving **3s** in 52% yield. Considering the reaction's insensitivity to steric hindrance, secondary alcohols were further attempted. Interestingly, although the tertiary radicals are more stable, the yields increased slightly. Products **3t–3v** were synthesized in 57%–70% yields. Much to our delight, derivatives of spirane lactones, which are widely used in drug synthesis and natural product research,^[^ ^18^ ^]^ could also be conveniently obtained through our developed methods, giving **3w** and **3x** in 88% and 65% yield. In addition, the spiro‐lactonization modification of testosterone also demonstrated the applicability of our protocol for late‐stage functionalization (for **3y**).

On the other hand, a range of acrylate derivatives could also be used as alkylating agents. When using acrylamide, γ‐hydroxy amides **4a**–**4e** were afforded moderate to considerable yield. Moreover, the screening of alternative olefins has allowed the preparation of γ‐hydroxy nitriles **5a**–**5d**, and γ‐hydroxy phosphonate **6a**–**6c**. Unfortunately, the low reactivity of substituted styrene (for **7**), vinyl sulfones (for **8**), and sulphoxide (for **9**) in the standard conditions did not allow their use as effective alkylating reagents, while vinyl ketone also failed to give **10** due to rapid proton exchange.

### 2.3 Mechanistic Studies

**Scheme 3:**
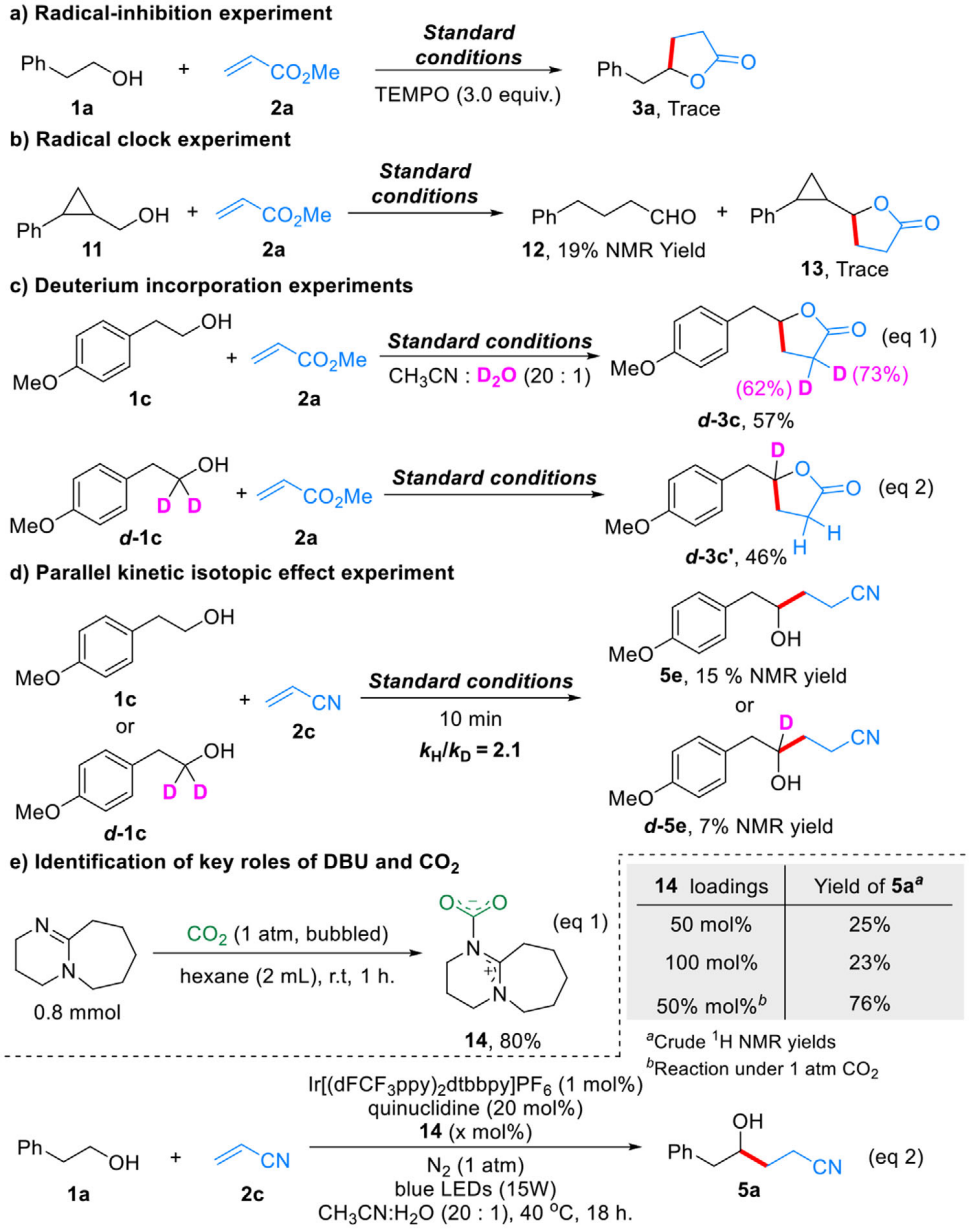
Control Experiments Regarding the Reaction Mechanism.

After assessing the practical efficiency of the transformation, a series of control experiments were conducted to investigate the reaction mechanism (**Scheme** **3**). We suppose that the α‐C‐H cleavage from the hydroxyl group of substrates is the most challenging step. Therefore, the capture and identification of the α‐hydroxy radical is the priority. A radical inhibition experiment was first conducted by introducing 2,2,6,6‐tetramethyl‐1‐piperidinyloxy (TEMPO) under standard reaction conditions. As shown in Scheme 3a, the reaction did not proceed to the desired product **3a**, while the possible capture compounds were not detected by HRMS possibly due to their instability. To get direct evidence for the existence of radical intermediate, a radical clock experiment was conducted using substrate **11** (Scheme 3b). As expected, the ring‐open product 4‐phenylbutanal **12** was detected in 19% NMR yield and the desired product **13** was scarcely detected, which suggests the existence of radical intermediates. Next, deuterium incorporation experiments were conducted, where H_2_O was replaced by D_2_O (Scheme 3c, eq. 1). Deuterium incorporation rates for the two hydrogen atoms at the α‐position of the ester group in ***d*‐3c** were found to be 62% and 73%, respectively. In contrast, when deuterated alcohol ***d*‐1c** was utilized as the starting material, no deuterium incorporation was observed in product ***d*‐3c'**, indicating that H_2_O is the primary hydrogen source, although the rapid proton exchange catalyzed by DBU cannot be ruled out (Scheme 3c, eq. 2).^[^ ^12^, ^19^ ^]^ Subsequently, a parallel kinetic isotope experiment gave a kinetic isotope effect (KIE) of 2.1, suggesting that the hydrogen transfer step was the rate‐determining step (Scheme 3d).^[^ ^11^, ^13^, ^20^ ^]^ Finally, the DBU‐CO_2_ adduct **14** was synthesized by bubbling a CH_3_CN solution of DBU with CO_2_ (Scheme 3e, eq. 1). Replacing DBU and the CO_2_ atmosphere by 50 mol% compound **14** led to 25% NMR yield for **5a**, and when the amount of **14** doubled, the yield of **5a** remained the same. In contrast, performing the reaction with 50 mol% of **14** under CO_2_ atmosphere restored the yield for **5a** to 76% (Scheme 3e, eq. 2). Considering that this adduct would be decomposed by H_2_O and that the CO_2_ atmosphere inhibits this process, we reckon that the dynamic equilibrium of the DBU‐CO_2_‐H_2_O system is key to the efficient catalytic process.^[^ ^19^, ^21^ ^]^

**Scheme 4:**
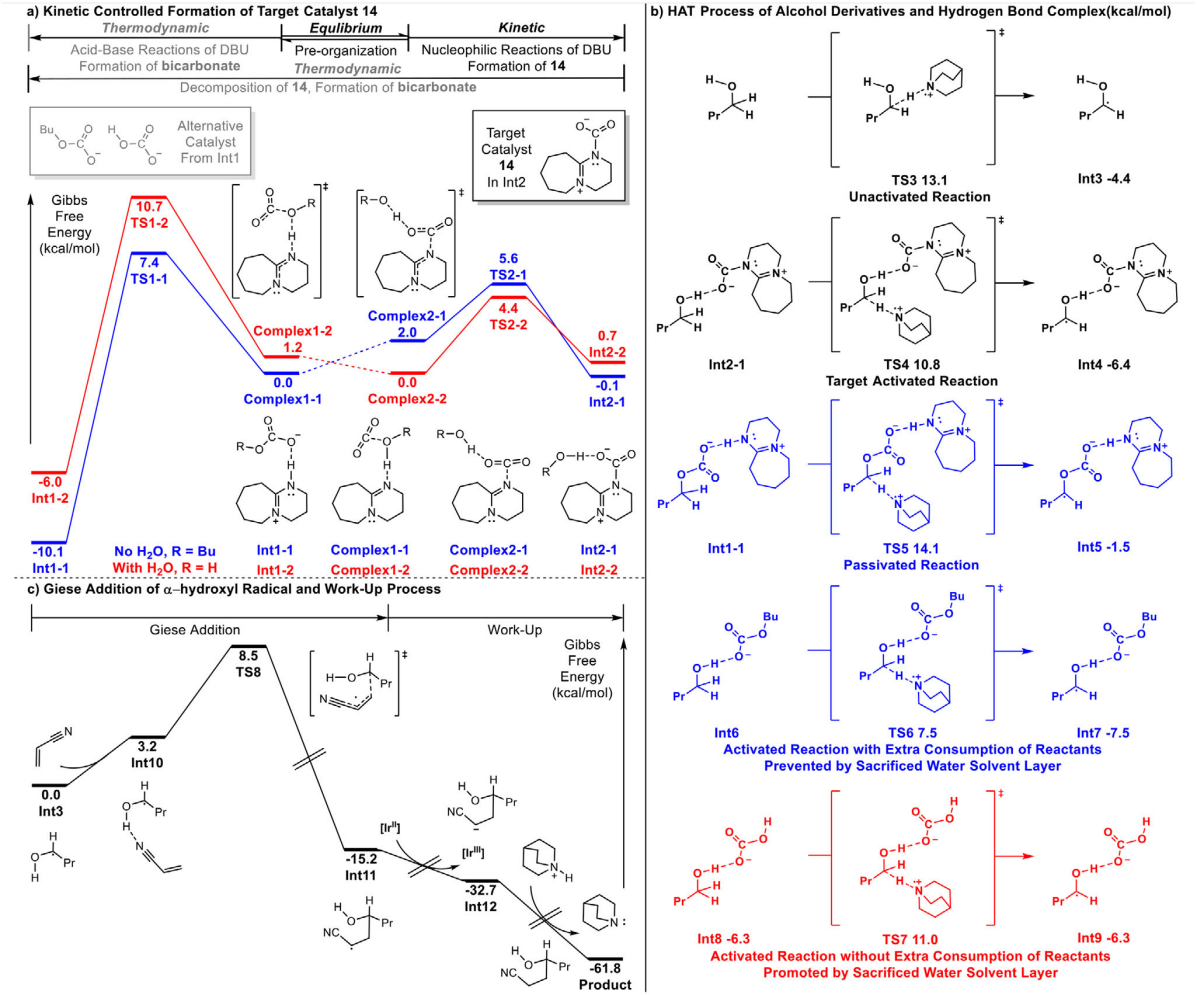
Computational studies on the reaction mechanism.

The theoretical calculations and mechanistic discussions are summarized in **Scheme** **4**. All calculations were performed using *n*‐butanol as the model reactant at the PWBP95‐D4(BJ)/def2‐TZVPP//B3LYP‐D3(BJ)/def2‐SVP level (For detailed information, see Section S5, Supporting Information).^[^ ^22^ ^]^ The DBU‐CO_2_ adduct **14**, which forms from DBU and CO₂, has been previously reported^[^ ^19^, ^23^ ^]^ and verified in Scheme 3e. It is postulated to activate the α‐C─H bond of alcohols via O···H─O hydrogen bonding. However, the previous report and the observed decomposition of **14** upon storage (For detailed information, see Section S4e, Supporting Information) suggest its susceptibility to alcohol/H_2_O‐induced degradation into bicarbonate or alkyl carbonate iminium salts. Scheme 4a reveals the kinetic‐controlled formation of target catalyst **14** and the thermodynamic‐controlled formation of the alternative catalyst bicarbonate. Alcohol‐assisted nucleophilic addition of DBU to CO₂ exhibits a low activation energy of 5.6 kcal mol^−1^, and the reverse is comparable, being 5.7 kcal mol^−1^. The DBU‐mediated alcohol deprotonation to form alkyl carbonate salts also presents a low activation energy (7.4 kcal mol^−1^) and is exothermic by 10.1 kcal mol^−1^, indicating that alcoholysis of **14** is also kinetically and thermodynamically favored. This means that kinetics will drive the generation of the catalyst **14**, while thermodynamics will finally drive the decomposition of the catalyst **14**. H_2_O slightly alters the kinetic preference; the formation activation energy decreases to 4.4 kcal mol^−1^ versus a decomposition one of 10.7 kcal mol^−1^. Moreover, H_2_O solvation directs the decomposition pathway toward bicarbonate rather than alkyl carbonate species. This in situ‐generated bicarbonate exhibits enhanced catalytic activity compared to metal‐stabilized counterparts due to improved solubility and reduced aggregation in organic solvent. Scheme 4b compares C─H activation across different model systems. Modal reactions with hydrogen bond network show consistent trends (for detailed information see Schemes S9 and S10, Supporting Information). The unactivated alcohol undergoes HAT with an energy barrier of 13.1 kcal mol^−1^ (ΔG = −4.4 kcal mol^−1^). Hydrogen bonding‐induced electron density modulation at the oxygen atom critically regulates HAT reactivity: electron‐donating hydrogen bonds lower the activation energy (**TS4**, **Int4**), while electron‐withdrawing effects from carbamate conjugation (**Int1‐1**) increase the barrier (**TS5**, **Int5**) unlike the electrostatic activated carbamate‐directed HAT.^[^ ^7e^ ^]^ Although bicarbonate salts can activate HAT through hydrogen bonding (**TS6**, barrier reduction), the formation of a two‐alcohol complex leads low atom economy. Water‐mediated pathways (**TS7**) restore the single‐alcohol complex, rationalizing the observed yield improvement with controlled water addition. However, excessive water dilutes reactants, reverting to unactivated conditions (See Table S4, Supporting Information). Scheme 4c illustrates the Giese addition of alcohol‐derived radicals to acrylonitrile, followed by reduction and protonation. The single‐step activation energy of 8.5 kcal mol^−1^ and substantial exothermicity align with the observed spontaneity and high efficiency of this transformation.

**Scheme 5:**
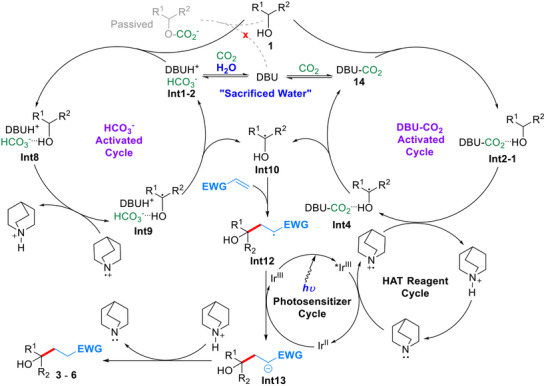
Proposed catalytic cycle.

Based on the above results and discussions, the proposed catalytic cycle is delineated in **Scheme** **5**. In CH_3_CN‐H_2_O mixed solvent, DBU undergoes rapid nucleophilic capture of CO₂. This leads to the form of metastable DBU–CO₂ adduct **14**, a reaction that effectively sequesters CO₂ in the solution phase.^[^ ^19^, ^23^ ^]^ This adduct subsequently engages in hydrogen‐bonding association with the alcohol to generate a complex **Int2‐1**. Concurrently, the **Ir^III^** photosensitizer undergoes photoexcitation from the S_0_ to T_1_ state, initiating SET with quinuclidine to yield **Ir^II^** and quinuclidine radical cation.^[^ ^13^, ^15^, ^24^ ^]^ The latter participates in HAT with **Int2‐1**, producing alcohol radical‐containing hydrogen‐bonded complex **Int4**. Dissociation of **Int4** releases free alcohol radical **Int10** while regenerating catalyst **14**. The H_2_O environment introduces critical mechanistic bifurcation. H_2_O‐mediated decomposition of **14** preferentially generates bicarbonate species **Int1‐2** rather than alkyl carbonate derivatives.^[^ ^21^, ^23^ ^]^ This bicarbonate intermediate similarly functions as a hydrogen‐bonding proton acceptor, coordinating with alcohol substrates to form complex **Int8**. **Int8** undergoes an analogous HAT with the quinuclidine radical cation. This reaction yields radical complex **Int9** that releases free alcohol radical **Int10** upon dissociation, thereby completing the bicarbonate regeneration cycle. The alcohol radical **Int10** engages in Giese addition with electron‐deficient olefins, forming radical intermediate **Int12**. Subsequent SET reduction by **Ir^II^** regenerates the **Ir^III^** photosensitizer and anion **Int13**. Final proton transfer from quinuclidinium salt yields the desired product and restores the quinuclidine mediator, thereby closing the catalytic cycle.

## 3 Conclusion

We have presented a novel photoredox catalytic platform for the direct α‐C‐H monoalkylation of alcohols, utilizing a synergistic combination of CO_2_, DBU, and H_2_O within a tunable hydrogen‐bonding network. This approach leverages the dual function of CO_2_, which acts as both a dynamic activator and a selectivity modulator. Consequently, it overcomes previous challenges in C‐H activation, such as harsh reaction conditions and competing activation pathways. The method exhibits excellent functional group tolerance and scalability, offering an efficient, sustainable strategy for the direct alkylation of alcohols. Furthermore, mechanistic insights gained from DFT calculations with possible key intermediates support the proposed catalytic cycle, marking a significant advancement in the field of C‐H activation for alcohols.

## Conflict of Interest

The authors declare no conflict of interest.

## Supporting information



Supporting Information

## Data Availability

The data that support the findings of this study are available in the supplementary material of this article.
