# Supplementary material for: CO2‐DBU‐Triggered Photoredox‐Catalyzed Direct α‐C‐H Alkylation of Alcohols
Source: Adv Sci (Weinh). 2025 Jun 9;12(31):e07490. doi: 10.1002/advs.202507490 (PMC12376653; doi:10.1002/advs.202507490)
Supplement: Supplementary file 1 — Supporting Information [file ADVS-12-e07490-s001.docx]

***Supporting Information***

***for***

**CO_2_-DBU-Triggered Photoredox-Catalyzed Direct α-C-H Alkylation of Alcohols**

Zeyu Zhang^‡^*^a^,* Zongchang Han^‡^*^b^*, Yuhao Shang*^a^*, Han-Shi Hu*^b^*, Jun Li*^b^*, Chanjuan Xi^*^*^ac^*

*^a^*MOE Key Laboratory of Bioorganic Phosphorus Chemistry & Chemical Biology, Department of Chemistry, Tsinghua University, Beijing 100084, China;

*^b^*Department of Chemistry and Engineering Research Center of Advanced Rare-Earth Materials of Ministry of Education, Tsinghua University, Beijing 100084, China;

*^c^*State Key Laboratory of Elemento-Organic Chemistry, Nankai University, Tianjin 300071, China.

*E-mail: [cjxi@tsinghua.edu.cn](mailto:cjxi@tsinghua.edu.cn)

**Contents**

1. General information………………………………………………….……...........S2
2. Optimization of reaction conditions……....……………………….....……...........S3

3. Procedure for CO_2_-induced photocatalyzed α-C-H alkylation of alcohols as well as analytical data of products…………...................................................................…. S12

4. Mechanism experiments………………………………...............…………….....S26

5. Details and notes on DFT calculations...................................................................S40

6. References………………………………………………………………..…......S103

7. Copies of NMR spectra for products …...……………………..................….....S106

**1. General information**

All the reactions were carried out using pre-dried Schlenk tubewith Teflon-lined-septum under CO_2_ atmosphere. Unless otherwise noted, the reagents were purchased from commercially available suppliers and used without further purification. All of the solvents were dried prior to use. Among them, the acetonitrile (CH_3_CN) (99.9%, extra dry, with molecular sieves, water < 30 ppm) is purchased from Energy Chemical. All the photocatalysts are directly purchased from Bidepharm. Column chromatography is performed using silica gel size of 10-40 µm purchased from Ocean Chemical Factory of Yantai, China. All NMR spectra are collected on 400 MHz spectrometer at ambient temperature with CDCl_3_ as the solvent. All chemical shifts are reported in δ‐scale as parts per million [ppm] (multiplicity, coupling constant *J*, number of protons) relative to TMS (Me_4_Si) and d-solvent peaks, respectively. Coupling constants (*J*) are given in Hertz [Hz]. Abbreviations used for signal multiplicity. ^1^H and ^19^F NMR: s = singlet, d = doublet, t = triplet, q = quartet, dd = doublet of doublets, dt = doublet of triplets, td = triplet of doublets, ddd = doublet of doublets of doublets, tt = triplet of triplets, and m = multiplet. HRMS(EI^+^) analysis was performed on a Shimadzu GCMS-FT/TOF spectrometer. HRMS(ESI+) analysis was performed on a Shimadzu LCMS-IT/TOF spectrometer.

Information about the photoreactor: the photoreactor (Type RLH-18CU, Series Number: 221A4400) used in this research was purchased from Rogertech, Beijing, China. The photoreactor was made up of of 8 blue LED bulbs (15 W for each) with a circulating coolant pump to keep the reactor at the set temperature. Spectral distribution: 425 nm. In the reaction, each Schlenk tube is irradiated by one of the light bulbs separately. The approximate distance of the tube to the closest light bulb is 2 cm. A magnetic stirrer is placed under the photoreactor to keep the reaction stirred.


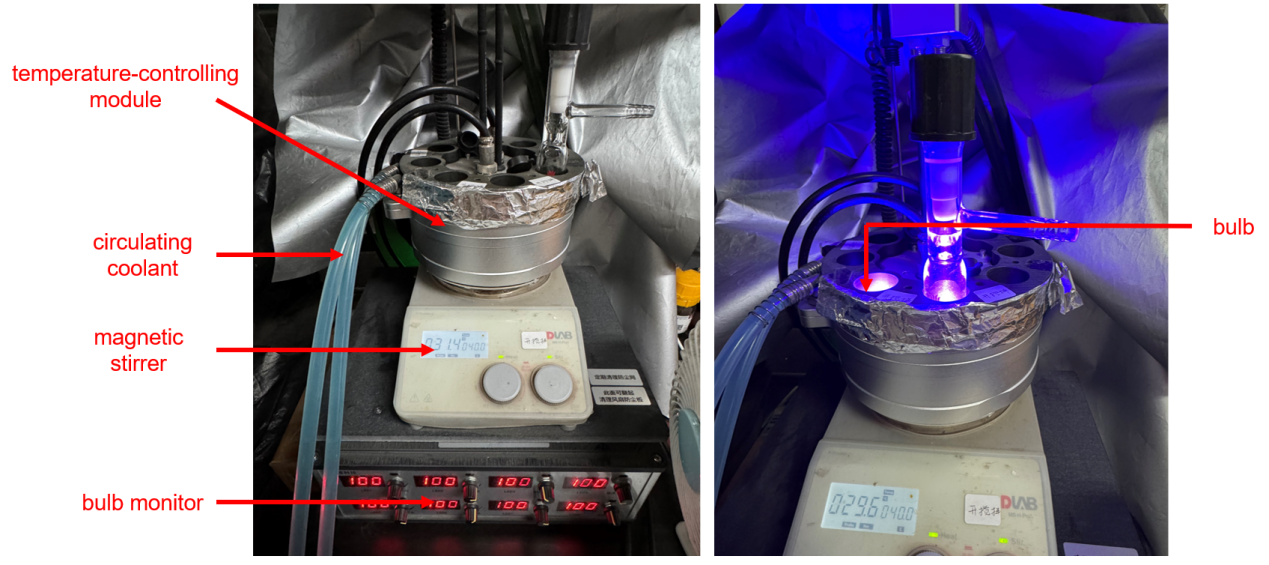


**Figure. S1** Photos of the photoreactor

1. **Optimization of reaction conditions**

**Table S1.** The screening of ratio of reactants*^a^*

*^a^*Reaction conditions: phenylethanol **1a** (X mmol), methyl acrylate **2a** (Y mmol), Ir[(dFCF_3_ppy)_2_dtbbpy]PF_6_ (1 mol%), quiniclidine (20 mol%), and DBN (50 mol%) in the mixture of 1 mL CH_3_CN and 100 μL H_2_O at room temperature for 18 h under 15 W blue LEDs in CO_2_ atmosphere (1 atm) unless otherwise stated.

*^b^*Yields of product **3a** determined by crude ^1^H NMR, CH_2_Br_2_ as internal standard.

**Table S2.** The screening of base*^a^*

*^a^*Reaction conditions: phenylethanol **1a** (0.6 mmol), methyl acrylate **2a** (0.3 mmol), Ir[(dFCF_3_ppy)_2_dtbbpy]PF_6_ (1 mol%), quiniclidine (20 mol%), and base (50 mol%) in 1 mL CH_3_CN and and 100 μL H_2_O at room temperature for 18 h under 15W blue LEDs in CO_2_ atmosphere (1 atm) unless otherwise stated.

*^b^*Yields of product **3a** determined by crude ^1^H NMR, CH_2_Br_2_ as internal standard.

**Table S3.** Preliminary control experiments*^a^*

*^a^*Reaction conditions: phenylethanol **1a** (0.6 mmol), methyl acrylate **2a** (0.3 mmol), Ir[(dFCF_3_ppy)_2_dtbbpy]PF_6_ (1 mol%), quiniclidine (20 mol%), and DBU (50 mol%) in 1 mL CH_3_CN and and 100 μL H_2_O at room temperature for 18 h under 15 W blue LEDs in CO_2_ atmosphere (1 atm) unless otherwise stated.

*^b^*Yields of product **3a** determined by crude ^1^H NMR, CH_2_Br_2_ as internal standard.

As shown in **Table S3**, entry 3, considering that CO_2_ bubbling could increase the reaction yield by increasing the solubility of CO_2_ in the solution, subsequent reactions are carried out with CO_2_ bubbling for 2 minutes.

**Table S4.** The screening of equivalence of H_2_O*^a^*

*^a^*Reaction conditions: phenylethanol **1a** (0.6 mmol), methyl acrylate **2a** (0.3 mmol), Ir[(dFCF_3_ppy)_2_dtbbpy]PF_6_ (1 mol%), quiniclidine (20 mol%), and DBU (50 mol%) in 1 mL CH_3_CN and and X μL H_2_O at room temperature for 18 h under 15 W blue LEDs in CO_2_ atmosphere (1 atm) unless otherwise stated.

*^b^*Yields of product **3a** determined by crude ^1^H NMR, CH_2_Br_2_ as internal standard.

**Table S5.** The screening of photocatalyst*^a^*

*^a^*Reaction conditions: phenylethanol **1a** (0.6 mmol), methyl acrylate **2a** (0.3 mmol), photocatalysts (1 mol%), quiniclidine (20 mol%), and DBU (50 mol%) in 1 mL CH_3_CN and and 50 μL H_2_O at room temperature for 18 h under 15 W blue LEDs in CO_2_ atmosphere (1 atm) unless otherwise stated.

*^b^*Yields of product **3a** determined by crude ^1^H NMR, CH_2_Br_2_ as internal standard.

**Table S6.** The screening of the solvents*^a^*

*^a^*Reaction conditions: phenylethanol **1a** (0.6 mmol), methyl acrylate **2a** (0.3 mmol), Ir[(dFCF_3_ppy)_2_dtbbpy]PF_6_ (1 mol%), quiniclidine (20 mol%), and DBU (50 mol%) in 1 mL solvent and and 50 μL H_2_O at room temperature for 18 h under 15 W blue LEDs in CO_2_ atmosphere (1 atm) unless otherwise stated.

*^b^*Yields of product **3a** determined by crude ^1^H NMR, CH_2_Br_2_ as internal standard.

**Table S7.** The screening of temperature*^a^*

*^a^*Reaction conditions: phenylethanol **1a** (0.6 mmol), methyl acrylate **2a** (0.3 mmol), Ir[(dFCF_3_ppy)_2_dtbbpy]PF_6_ (1 mol%), quiniclidine (20 mol%), and DBU (50 mol%) in 1 mL CH_3_CN and and 50 μL H_2_O at screened temperatures for 18 h under 15 W blue LEDs in CO_2_ atmosphere (1 atm) unless otherwise stated.

*^b^*Yields of product **3a** determined by crude ^1^H NMR, CH_2_Br_2_ as internal standard.

**Table S8.** The substitutaion of methyl acrylate without stabilizer

*^a^*Reaction conditions: phenylethanol **1a** (0.6 mmol), methyl acrylate (without stabilizer) **2a** (0.3 mmol), Ir[(dFCF_3_ppy)_2_dtbbpy]PF_6_ (1 mol%), quiniclidine (20 mol%), and DBU (50 mol%) in 1 mL CH_3_CN and and 50 μL H_2_O at 40 ^o^C for 18 h under 15 W blue LEDs in CO_2_ atmosphere (1 atm) unless otherwise stated.

*^b^*Yields of product **3a** determined by crude ^1^H NMR, CH_2_Br_2_ as internal standard.

As shown in **Table S8**, considering that commercially available acrylates usually contain 10-30 ppm stabilizers (such as MEHQ) to prevent the polymerization of acrylates, which may inhibit the radical addition process, another substrate screening was performed using a methyl acrylate without stabilizers (standard for GC-MS). The results in **Table S8**, entry 3 showed that methyl acrylate without stabilizer could give higher yield compared to that with MEHQ, so we chose the former one for the best yield.

With all the results from **Table S1** - **S8**, subsequent control experiments were conducted which deviated from standard conditions. And the results are shown in **Table S9**.

**Table S9.** Control experiments deviated from standard conditions*^a^*

*^a^*Standard Reaction conditions: phenylethanol **1a** (0.6 mmol), methyl acrylate **2a** (0.3 mmol), Ir[(dFCF_3_ppy)_2_dtbbpy]PF_6_ (1 mol%), quiniclidine (20 mol%), and DBU (50 mol%) in 1 mL CH_3_CN and and 50 μL H_2_O at 40 ^o^C for 12 h under 15 W blue LEDs in CO_2_ atmosphere (1 atm) unless otherwise stated.

*^b^*Yields of product **3a** determined by crude ^1^H NMR, CH_2_Br_2_ as internal standard.

**3. Procedure for CO_2_-induced photocatalyzed α-C-H alkylation of alcohols as well as analytical data of products**

To a 25 mL sealed tube was added Ir[(dFCF_3_ppy)_2_dtbbpy]PF_6_ (3.4 mg, 1 mol%), quinucidine (6.7 mg, 20 mol%), and DBU (22.8 mg, 50 mol%). The tube was degassed by vacuum evacuation and back-filled with CO_2_ for three times. Then anhydrous CH_3_CN (1 mL) and 50 μL H_2_O was added into the tube use syringe. Afterwards, the corresponding alcohol **1** (0.45 mmol - 0.6 mmol, 1.5 equiv. - 2.0 equiv.) and compound **2** (0.3 mmol) were added through a micro-syringe. Before the reaction starts, CO_2_ was bubbled into the solution for 2 minutes. Then the reaction tube was sealed and stirred at 40 ^o^C for 12 h under the irradiation of 15 W blue LED lamps. After completion of the reaction, it was carefully quenched with 2 M HCl (or H_2_O) and the mixture was extracted 3 times with CH_2_Cl_2_. The combined organic layers were dried over anhydrous Na_2_SO_4_ and concentrated under reduced pressure. The residual product was purified by flash chromatography on silica gel to afford the corresponding product **3 - 6**.

**5-([1,1'-Biphenyl]-4-ylmethyl)dihydrofuran-2(3*H*)-one (3b):** white solid, 43.1 mg, 57% yield, (PE: EA = 15:1). ^1^H NMR (400 MHz, CDCl_3_) δ 7.62 – 7.49 (m, 4H), 7.47 – 7.38 (m, 2H), 7.37 – 7.26 (m, 3H), 4.75 (dq, *J* = 13.0 Hz, 6.3 Hz, 1H), 3.09 (dd, *J* = 14.1 Hz, 6.2 Hz, 1H), 2.96 (dd, *J* = 14.1 Hz, 6.1 Hz, 1H), 2.55 – 2.35 (m, 2H), 2.28 (dddd, *J* = 12.8 Hz, 9.4 Hz, 6.7 Hz, 4.8 Hz, 1H), 1.97 (dtd, *J* = 12.8 Hz, 9.4 Hz, 7.7 Hz, 1H).^13^C NMR (101 MHz, CDCl_3_) δ 177.1, 140.8, 140.0, 135.1, 130.0, 128.9, 127.5, 127.4, 127.1, 80.9, 41.1, 28.8, 27.3. HRMS (EI+) calculated m/z for C_17_H_16_O_2_^+^ [M]^+^ : 252.1145, found 252.1146.

**5-(4-Methoxybenzyl)dihydrofuran-2(3*H*)-one (3c)**^1^**:** colorless oil, 34.0 mg, 55% yield, (PE: CH_2_Cl_2_ = 5:1). ^1^H NMR (400 MHz, CDCl_3_) δ 7.14 (d, *J* = 8.6 Hz, 2H), 6.85 (d, *J* = 8.6 Hz, 2H), 4.79 – 4.60 (m, 1H), 3.79 (s, 3H), 3.00 (dd, *J* = 14.1 Hz, 5.9 Hz, 1H), 2.88 (dd, *J* = 14.1 Hz, 6.2 Hz, 1H), 2.51 – 2.30 (m, 2H), 2.29 – 2.18 (m, 1H), 1.94 (dtd, *J* = 12.8 Hz, 9.2 Hz, 7.6 Hz, 1H).^13^C NMR (101 MHz, CDCl_3_) δ 177.3, 158.7, 130.6, 127.9, 114.2, 81.1, 55.4, 40.5, 28.8, 27.1.

**5-(4-Methoxyphenethyl)dihydrofuran-2(3*H*)-one (3d)**^2^**:** colorless oil, 37.0 mg, 56% yield, (PE: CH_2_Cl_2_ = 5:1). ^1^H NMR (400 MHz, CDCl_3_) δ 7.11 (d, *J* = 8.6 Hz, 2H), 6.91 – 6.78 (m, 2H), 4.46 (ddd, *J* = 14.8 Hz, 8.1 Hz, 4.9 Hz, 1H), 3.79 (s, 3H), 2.85 – 2.62 (m, 2H), 2.59 – 2.47 (m, 2H), 2.38 – 2.24 (m, 2H), 2.07 – 1.98 (m, 1H), 1.95 – 1.79 (m, 2H). ^13^C NMR (101 MHz, CDCl_3_) δ 177.3, 158.2, 132.9, 129.5, 114.1, 80.0, 55.4, 37.7, 30.8, 30.0, 28.1.

**5-(4-Chlorophenethyl)dihydrofuran-2(3*H*)-one (3e):** yellow oil, 36.3 mg, 54% yield, (PE: EA = 15:1). ^1^H NMR (400 MHz, CDCl_3_) δ 7.32 – 7.20 (m, 2H), 7.13 (d, *J* = 8.4 Hz, 2H), 4.46 – 4.43 (m, 3H), 2.88 – 2.65 (m, 2H), 2.58 – 2.50 (m, 2H), 2.36 – 2.26 (m, 1H), 2.01 (dtd, *J* = 14.1 Hz, 8.8 Hz, 5.4 Hz, 1H), 1.94 – 1.83 (m, 2H). ^13^C NMR (101 MHz, CDCl_3_) δ 177.1, 139.3, 132.0, 129.9, 128.7, 79.7, 37.3, 31.1, 28.8, 28.0. HRMS (EI+) calculated m/z for C_12_H_13_O_2_Cl^+^ [M]^+^ : 224.0599, found 224.0598.

**5-(4-(Trifluoromethyl)phenethyl)dihydrofuran-2(3*H*)-one (3f):** yellow oil, 51.9 mg, 67% yield, (PE: CH_2_Cl_2_ = 10:1). ^1^H NMR (400 MHz, CDCl_3_) δ 7.55 (d, *J* = 8.1 Hz, 2H), 7.32 (d, *J* = 8.0 Hz, 2H), 4.46 (tdd, *J* = 8.3 Hz, 6.8 Hz, 4.4 Hz, 1H), 3.03 – 2.76 (m, 2H), 2.63 – 2.49 (m, 2H), 2.40 – 2.27 (m, 1H), 2.05 (dtd, *J* = 14.2 Hz, 8.9 Hz, 5.3 Hz, 1H), 1.98 – 1.82 (m, 2H). ^13^C NMR (101 MHz, CDCl_3_) δ 177.0, 145.0, 128.9, 128.7 (q, *J* = 22.2 Hz), 125.6 (q, *J* = 4.0 Hz), 124.4 (q, *J* = 273.7 Hz), 79.6, 37.2, 31.7, 28.9, 28.1. ^19^F NMR (376 MHz, CDCl_3_) δ -62.4. HRMS (EI+) calculated m/z for C_13_H_13_O_2_F_3_^+^ [M]^+^ : 258.0864, found 258.0865.

**5-Neopentyldihydrofuran-2(3*H*)-one (3g)**^3^**:** colorless oil, 35.6 mg, 76% yield, (PE: EA = 20:1). ^1^H NMR (400 MHz, CDCl_3_) δ 4.64 – 4.53 (m, 1H), 2.54 – 2.47 (m, 2H), 2.34 (dq, *J* = 18.9 Hz, 6.3 Hz, 1H), 1.84 (ddd, *J* = 19.4 Hz, 12.6 Hz, 9.6 Hz, 1H), 1.71 (dd, *J* = 14.7 Hz, 8.4 Hz, 1H), 1.49 (dd, *J* = 14.7 Hz, 3.5 Hz, 1H), 0.98 (s, 9H). ^13^C NMR (101 MHz, CDCl_3_) δ 177.5, 78.9, 49.7, 30.2, 30.1, 30.0, 29.0.

**5-(4-((*tert*-Butyldimethylsilyl)oxy)butyl)dihydrofuran-2(3*H*)-one (3h)**^4^**:** yellow oil, 57.2 mg, 70% yield, (PE: CH_2_Cl_2_ = 4:1). ^1^H NMR (400 MHz, CDCl_3_) δ 4.57 – 4.43 (m, 1H), 3.62 (t, *J* = 6.1 Hz, 2H), 2.53 (dd, *J* = 9.5 Hz, 6.9 Hz, 2H), 2.33 (dq, *J* = 13.4 Hz, 6.7 Hz, 1H), 1.86 (ddd, *J* = 14.1 Hz, 10.9 Hz, 7.7 Hz, 1H), 1.81 – 1.70 (m, 1H), 1.68 – 1.41 (m, 5H), 0.89 (s, 9H), 0.05 (s, 6H). ^13^C NMR (101 MHz, CDCl_3_) δ 177.4, 81.1, 62.9, 35.4, 32.5, 29.0, 28.1, 26.1, 21.8, 18.4, -5.2.

**5-(2-Phenoxyethyl)dihydrofuran-2(3*H*)-one (3i):** yellow oil, 37.8 mg, 61% yield, (PE: CH_2_Cl_2_ = 4:1). ^1^H NMR (400 MHz, CDCl_3_) δ 7.28 (dt, *J* = 9.6 Hz, 4.8 Hz, 2H), 6.95 (t, *J* = 7.3 Hz, 1H), 6.89 (d, *J* = 7.9 Hz, 2H), 4.83 – 4.73 (m, 1H), 4.18 – 4.07 (m, 2H), 2.56 (dd, *J* = 9.5 Hz, 6.8 Hz, 2H), 2.46 – 2.38 (m, 1H), 2.15 (dd, *J* = 12.4 Hz, 6.1 Hz, 2H), 2.04 – 1.91 (m, 1H). ^13^C NMR (101 MHz, CDCl_3_) δ 177.0, 158.7, 129.7, 121.1, 114.6, 78.0, 63.9, 35.5, 28.9, 28.3. HRMS (EI+) calculated m/z for C_12_H_15_O_3_^+^ [M]^+^ : 207.1016, found 207.1017.

**5-(2,2-Diethoxyethyl)dihydrofuran-2(3*H*)-one (3j):** yellow oil, 28.5 mg, 47% yield, (PE: CH_2_Cl_2_ = 1:1). ^1^H NMR (400 MHz, CDCl_3_) δ 4.74 – 4.60 (m, 2H), 3.74 (dq, *J* = 9.4 Hz, 7.1 Hz, 1H), 3.70 – 3.61 (m, 1H), 3.58 – 3.49 (m, 2H), 2.54 (dd, *J* = 9.6 Hz, 6.7 Hz, 2H), 2.37 (dq, *J* = 13.2 Hz, 6.6 Hz, 1H), 2.07 – 1.84 (m, 3H), 1.22 (td, *J* = 7.1 Hz, 3.7 Hz, 6H). ^13^C NMR (101 MHz, CDCl_3_) δ 177.3, 100.3, 77.8, 63.1, 62.0, 40.4, 28.9, 28.4, 15.5, 15.4. HRMS (ESI+) calculated m/z for C_10_H_19_O_4_^+^ [M+H]^+^ : 203.1278, found 203.1276.

**5-(Tetrahydro-2*H*-pyran-2-yl)dihydrofuran-2(3*H*)-one (3k)**^4^**:** yellow oil, 23.0 mg, 45% yield, (PE: CH_2_Cl_2_ = 2:1). ^1^H NMR (400 MHz, CDCl_3_) δ 4.34 (td, *J* = 6.7 Hz, 4.6 Hz, 1H), 4.02 – 3.92 (m, 1H), 3.50 – 3.36 (m, 2H), 2.65 – 2.52 (m, 1H), 2.49 – 2.41 (m, 1H), 2.26 – 2.20 (m, 2H), 1.94 – 1.85 (m, 1H), 1.70 – 1.62 (m, 2H), 1.60 – 1.43 (m, 2H), 1.37 – 1.28 (m, 1H). ^13^C NMR (101 MHz, CDCl_3_) δ 177.6, 82.2, 78.5, 68.6, 28.4, 27.7, 26.0, 23.1, 23.0.

**5-((Methyl(phenyl)amino)methyl)dihydrofuran-2(3*H*)-one (3l):** yellow oil, 43.7 mg, 71% yield, (CH_2_Cl_2_ : CH_3_OH= 40:1). ^1^H NMR (400 MHz, CDCl_3_) δ 7.26 – 7.17 (m, 2H), 6.77 (d, *J* = 8.0 Hz, 2H), 6.71 (t, *J* = 7.2 Hz, 1H), 3.76 (t, *J* = 5.8 Hz, 2H), 3.64 (s, 3H), 3.45 (t, *J* = 5.8 Hz, 2H), 3.38 – 3.32 (m, 2H), 2.35 (t, *J* = 7.1 Hz, 2H), 1.95 – 1.88 (m, 2H). ^13^C NMR (101 MHz, CDCl_3_) δ 173.9, 148.3, 129.4, 117.2, 113.3, 60.1, 53.8, 51.7, 51.0, 31.4, 22.3. HRMS (ESI+) calculated m/z for C_12_H_15_NO_2_Na^+^ [M+Na]^+^ : 228.0995, found 228.0996.

***tert*-Butyl-((5-oxotetrahydrofuran-2-yl)methyl)carbamate (3m)**^5^**:** yellow oil, 39.4 mg, 61% yield, (CH_2_Cl_2_ : CH_3_OH= 20:1). ^1^H NMR (400 MHz, CDCl_3_) δ 5.00 (s, 1H), 4.64 – 4.59 (m, 1H), 3.72 – 3.63 (m, 1H), 3.37 – 3.19 (m, 1H), 2.56 (dd, *J* = 9.5 Hz, 7.3 Hz, 2H), 2.32 – 2.25 (m, 1H), 2.05 – 1.95 (m, 1H), 1.45 (s, 9H). ^13^C NMR (101 MHz, CDCl_3_) δ 176.9, 156.2, 80.0, 79.8, 44.1, 28.7, 28.4, 24.6.

**5-(2-(Methylthio)ethyl)dihydrofuran-2(3*H*)-one (3n):** yellow oil, 19.7 mg, 41% yield, (PE : CH_2_Cl_2_ = 30:1). ^1^H NMR (400 MHz, CDCl_3_) δ 4.66 (ddd, *J* = 14.9 Hz, 8.2 Hz, 4.5 Hz, 1H), 2.71 – 2.54 (m, 4H), 2.38 (td, *J* = 13.3 Hz, 6.7 Hz, 1H), 2.13 (s, 3H), 2.02 (dtd, *J* = 13.9 Hz, 8.2 Hz, 5.7 Hz, 1H), 1.89 (dtd, *J* = 12.5 Hz, 9.2 Hz, 4.9 Hz, 2H). ^13^C NMR (101 MHz, CDCl_3_) δ 177.0, 79.4, 35.3, 30.1, 28.9, 28.0, 15.7. HRMS (EI+) calculated m/z for C_7_H_12_SO_2_^+^ [M]^+^ : 160.0553, found 160.0552.

**5-(3-Chloropropyl)dihydrofuran-2(3*H*)-one (3o)**^6^**:** colorless oil, 31.2 mg, 64% yield, (PE : EA = 20:1). ^1^H NMR (400 MHz, CDCl_3_) δ 4.52 (ddd, *J* = 14.6 Hz, 7.9 Hz, 4.6 Hz, 1H), 3.67 – 3.53 (m, 2H), 2.56 (dd, *J* = 9.6 Hz, 6.9 Hz, 2H), 2.37 (dq, *J* = 13.4 Hz, 6.7 Hz, 1H), 2.09 – 1.96 (m, 1H), 1.95 – 1.75 (m, 4H). ^13^C NMR (101 MHz, CDCl_3_) δ 177.1, 80.2, 44.6, 33.1, 28.9, 28.6, 28.2.

**5-((4-Methylthiazol-5-yl)methyl)dihydrofuran-2(3*H*)-one (3p):** yellow oil, 24.8 mg, 42% yield, (CH_2_Cl_2_ : EA = 40:1). ^1^H NMR (400 MHz, CDCl_3_) δ 4.52 (ddd, *J* = 14.6 Hz, 7.9 Hz, 4.6 Hz, 1H), 3.67 – 3.53 (m, 2H), 2.56 (dd, *J* = 9.6 Hz, 6.9 Hz, 2H), 2.37 (dq, *J* = 13.4 Hz, 6.7 Hz, 1H), 2.09 – 1.96 (m, 1H), 1.95 – 1.75 (m, 4H). ^13^C NMR (101 MHz, CDCl_3_) δ 177.1, 80.2, 44.6, 33.1, 28.9, 28.6, 28.2. HRMS (ESI+) calculated m/z for C_9_H_11_NO_2_SNa^+^ [M+Na]^+^ : 220.0403, found 220.0401.

**5-(2-(Thiophen-2-yl)ethyl)dihydrofuran-2(3*H*)-one (3q):** yellow oil, 31.2 mg, 53% yield, (CH_2_Cl_2_ : EA = 50:1). ^1^H NMR (400 MHz, CDCl_3_) δ 7.13 (dd, *J* = 5.1 Hz, 1.1 Hz, 1H), 6.92 (dd, *J* = 5.1 Hz, 3.4 Hz, 1H), 6.84 – 6.81 (m, 1H), 4.51 (tdd, *J* = 8.2 Hz, 6.7 Hz, 4.6 Hz, 1H), 3.10 – 2.93 (m, 2H), 2.57 – 2.51 (m, 2H), 2.39 – 2.27 (m, 1H), 2.09 (dtd, *J* = 14.1 Hz, 8.5 Hz, 5.7 Hz, 1H), 2.02 – 1.93 (m, 1H), 1.88 (dtd, *J* = 12.8 Hz, 9.5 Hz, 8.1 Hz, 1H). ^13^C NMR (101 MHz, CDCl_3_) δ 177.1, 143.4, 127.0, 124.9, 123.5, 79.6, 37.7, 28.9, 28.0, 26.0. HRMS (EI+) calculated m/z for C_10_H_12_SO_2_^+^ [M]^+^ : 196.0553, found 196.0554.

**5-(2-(1*H*-Pyrazol-1-yl)ethyl)dihydrofuran-2(3*H*)-one (3r):** yellow oil, 24.9 mg, 46% yield, (CH_2_Cl_2_ : CH_3_OH = 20:1). ^1^H NMR (400 MHz, CDCl_3_) δ 7.53 (d, *J* = 1.4 Hz, 1H), 7.43 (d, *J* = 2.2 Hz, 1H), 6.25 (t, *J* = 2.0 Hz, 1H), 4.46 – 4.23 (m, 3H), 2.58 – 2.51 (m, 2H), 2.41 – 2.28 (m, 2H), 2.11 (ddd, *J* = 14.5 Hz, 10.2 Hz, 5.4 Hz, 1H), 1.85 (ddd, *J* = 18.0 Hz, 12.9 Hz, 9.6 Hz, 1H). ^13^C NMR (101 MHz, CDCl_3_) δ 176.8, 140.0, 130.0, 105.5, 77.6, 48.3, 36.3, 28.8, 27.9. HRMS (ESI+) calculated m/z for C_9_H_12_N_2_O_2_Na^+^ [M+Na]^+^ : 203.0791, found 203.0789.

**5-(2,6-Dimethylhept-5-en-1-yl)dihydrofuran-2(3H)-one (3s):** yellow oil, 32.8 mg, 52% yield, (PE : EA = 20:1). ^1^H NMR (400 MHz, CDCl_3_) δ 5.08 (t, *J* = 7.1 Hz, 1H), 4.66 – 4.51 (m, 1H), 2.53 (ddd, *J* = 9.6 Hz, 6.7 Hz, 2.9 Hz, 2H), 2.32 (dqd, *J* = 13.1 Hz, 6.6 Hz, 3.0 Hz, 1H), 2.09 – 1.90 (m, 2H), 1.89 – 1.72 (m, 2H), 1.68 (s, 3H), 1.66 – 1.51 (m, 5H), 0.95 (d, *J* = 6.3 Hz, 3H). ^13^C NMR (101 MHz, CDCl_3_) δ 177.4, 131.7, 124.5, 79.8, 79.3, 43.4, 43.0, 37.5, 36.9, 29.8, 29.6, 29.4, 29.0, 28.9, 28.8, 28.7, 25.9, 25.4, 20.0, 19.2, 17.8. HRMS (EI+) calculated m/z for C_13_H_22_O_2_^+^ [M]^+^ : 210.1615, found 210.1616.

**5-Hexyl-5-methyldihydrofuran-2(3*H*)-one (3t)**^7^**:** colorless oil, 31.5 mg, 57% yield, (PE : EA = 20:1). ^1^H NMR (400 MHz, CDCl_3_) δ 2.70 – 2.48 (m, 2H), 2.17 – 2.02 (m, 1H), 2.00 – 1.92 (m, 1H), 1.72 – 1.56 (m, 2H), 1.38 (s, 3H), 1.35 – 1.25 (m, 8H), 0.89 (t, *J* = 6.7 Hz, 3H). ^13^C NMR (101 MHz, CDCl_3_) δ 177.0, 87.1, 41.1, 33.1, 31.8, 29.6, 29.3, 25.8, 23.9, 22.7, 14.2.

**5-Benzyl-5-methyldihydrofuran-2(3*H*)-one (3u)**^8^**:** colorless oil, 39.9 mg, 70% yield, (PE : EA = 20:1). ^1^H NMR (400 MHz, CDCl_3_) δ 7.35 – 7.26 (m, 3H), 7.26 – 7.20 (m, 2H), 3.02 (d, *J* = 13.9 Hz, 1H), 2.88 (d, *J* = 13.9 Hz, 1H), 2.44 (ddd, *J* = 17.3 Hz, 9.8 Hz, 6.0 Hz, 1H), 2.20 (ddd, *J* = 12.4 Hz, 9.6 Hz, 6.0 Hz, 1H), 2.11 – 1.86 (m, 2H), 1.44 (s, 3H). ^13^C NMR (101 MHz, CDCl_3_) δ 176.9, 135.8, 130.5, 128.6, 127.2, 86.4, 46.9, 32.2, 29.4, 27.1.

**5-((Benzyloxy)methyl)-5-methyldihydrofuran-2(3*H*)-one (3v):** yellow oil, 42.9 mg, 65% yield, (CH_2_Cl_2_ : EA = 20:1). ^1^H NMR (400 MHz, CDCl_3_) δ 7.42 – 7.21 (m, 5H), 4.55 (q, *J* = 12.0 Hz, 2H), 3.52 (d, *J* = 10.2 Hz, 1H), 3.44 (d, *J* = 10.1 Hz, 1H), 2.74 (ddd, *J* = 18.4 Hz, 10.3 Hz, 8.4 Hz, 1H), 2.53 (ddd, *J* = 17.8 Hz, 10.3 Hz, 4.7 Hz, 1H), 2.34 (ddd, *J* = 12.8 Hz, 10.4 Hz, 4.7 Hz, 1H), 1.94 (ddd, *J* = 12.8 Hz, 10.3 Hz, 8.4 Hz, 1H), 1.39 (s, 3H). ^13^C NMR (101 MHz, CDCl_3_) δ 177.3, 137.8, 128.6, 127.9, 127.6, 85.5, 76.0, 73.7, 30.9, 29.9, 23.9. HRMS (EI+) calculated m/z for C_13_H_16_O_3_^+^ [M]^+^ : 220.1094, found 220.1094.

**1-Oxaspiro[4.4]nonan-2-one (3w)**^4^**:** colorless oil, 40.0 mg, 88% yield, (CH_2_Cl_2_ : EA = 50:1). ^1^H NMR (400 MHz, CDCl_3_) δ 2.59 (t, *J* = 8.1 Hz, 2H), 2.20 (t, *J* = 8.1 Hz, 2H), 2.07 – 1.97 (m, 2H), 1.93 – 1.77 (m, 2H), 1.74 – 1.63 (m, 4H). ^13^C NMR (101 MHz, CDCl_3_) δ 177.0, 95.2, 38.6, 32.6, 30.0, 23.9.

**1-Oxaspiro[4.5]decan-2-one (3x)**^4^**:** colorless oil, 30.0 mg, 65% yield, (CH_2_Cl_2_ : EA = 50:1). ^1^H NMR (400 MHz, CDCl_3_) δ 2.59 (t, *J* = 8.3 Hz, 2H), 2.01 (t, *J* = 8.3 Hz, 2H), 1.84 – 1.75 (m, 2H), 1.74 – 1.66 (m, 2H), 1.63 – 1.45 (m, 5H), 1.45 – 1.30 (m, 1H). ^13^C NMR (101 MHz, CDCl_3_) δ 176.9, 86.5, 37.0, 32.9, 28.7, 25.0, 22.7.

**(8*R*,10*R*,13*S*,14*S*,17*S*)-10,13-Dimethyl-1,3',4',6,7,8,9,10,11,12,13,14,15,16-tetradecahydro-5'*H*-spiro[cyclopenta[*a*]phenanthrene-17,2'-furan]-3,5'(2*H*)-dione (3y):** yellow oil, 47.2 mg, 46% yield, (CH_2_Cl_2_ : CH_3_OH = 40:1). ^1^H NMR (400 MHz, CDCl_3_) δ 5.75 (d, *J* = 6.2 Hz, 1H), 2.65 – 2.22 (m, 7H), 2.17 – 1.94 (m, 3H), 1.94 – 1.81 (m, 2H), 1.77 – 1.57 (m, 5H), 1.54 – 1.36 (m, 1H), 1.35 – 1.27 (m, 1H), 1.23 – 1.19 (m, 3H), 1.19 – 1.07 (m, 1H), 1.06 – 0.96 (m, 3H), 0.94 – 0.87 (m, 3H). ^13^C NMR (101 MHz, CDCl_3_) δ 220.6, 199.5, 176.9, 170.7, 170.5, 124.3, 124.2, 95.9, 53.9, 53.6, 51.0, 49.4, 47.6, 45.7, 38.8, 38.7, 36.0, 35.9, 35.8, 35.6, 35.3, 34.0, 32.8, 32.7, 31.8, 31.6, 31.4, 31.3, 30.9, 29.8, 29.4, 23.1, 21.9, 20.6, 20.4, 17.6, 17.5, 14.7, 13.8. HRMS (ESI+) calculated m/z for C_22_H_30_O_3_Na^+^ [M+Na]^+^ : 365.2088, found 365.2085.

**4-Hydroxy-*N,N*-dimethyl-5-phenylpentanamide (4a)**^9^**:** yellow oil, 41.1 mg, 62% yield, (CH_2_Cl_2_ : EA = 50:1). ^1^H NMR (400 MHz, CDCl_3_) δ 7.33 – 7.26 (m, 2H), 7.24 – 7.16 (m, 3H), 3.90 – 3.81 (m, 1H), 2.99 (s, 3H), 2.93 (s, 3H), 2.78 (qd, *J* = 13.5 Hz, 6.5 Hz, 2H), 2.57 – 2.40 (m, 2H), 1.93 – 1.85 (m, 1H), 1.80 – 1.68 (m, 1H). ^13^C NMR (101 MHz, CDCl_3_) δ 173.8, 138.8, 129.5, 128.5, 126.3, 72.6, 44.4, 37.4, 35.7, 31.1, 30.2.

**4-Hydroxy-*N,N*-dimethyl-6-(thiophen-2-yl)hexanamide (4b):** yellow oil, 49.2 mg, 68% yield, (CH_2_Cl_2_ : CH_3_OH = 40:1). ^1^H NMR (400 MHz, CDCl_3_) δ 7.10 (dd, *J* = 5.1 Hz, 1.2 Hz, 1H), 6.91 (dd, *J* = 5.1 Hz, 3.4 Hz, 1H), 6.83 – 6.77 (m, 1H), 3.69 – 3.64 (m, 2H), 3.08 – 2.87 (m, 8H), 2.51 (qdd, *J* = 16.4 Hz, 7.4 Hz, 5.5 Hz, 2H), 1.92 – 1.75 (m, 4H). ^13^C NMR (101 MHz, CDCl_3_) δ 174.0, 145.4, 126.9, 124.3, 123.0, 70.9, 39.9, 37.5, 35.8, 31.9, 30.4, 26.3. HRMS (ESI+) calculated m/z for C_12_H_19_NO_2_SNa^+^ [M+Na]^+^ : 264.1029, found 264.1027.

**4-Hydroxy-*N,N*-dimethyl-4-(tetrahydro-2*H*-pyran-4-yl)butanamide (4c):** yellow oil, 39.4 mg, 61% yield, (CH_2_Cl_2_ : CH_3_OH = 20:1). ^1^H NMR (400 MHz, CDCl_3_) δ 4.06 – 3.96 (m, 2H), 3.67 (s, 1H), 3.43 – 3.31 (m, 3H), 3.03 (s, 3H), 2.97 (s, 3H), 2.59 (ddd, *J* = 16.5 Hz, 7.4 Hz, 5.0 Hz, 1H), 2.48 (ddd, *J* = 16.5 Hz, 8.1 Hz, 5.0 Hz, 1H), 1.90 – 1.83 (m, 1H), 1.82 – 1.70 (m, 2H), 1.63 – 1.50 (m, 2H), 1.48 – 1.37 (m, 2H). ^13^C NMR (101 MHz, CDCl_3_) δ 174.1, 75.6, 68.2, 68.0, 41.7, 37.5, 35.8, 30.6, 29.1, 28.8, 28.4. HRMS (ESI+) calculated m/z for C_11_H_21_NO_3_Na^+^ [M+Na]^+^ : 238.1414, found 238.1413.

**5-([1,1'-Biphenyl]-4-yl)-4-hydroxy-*N,N*-dimethylpentanamide (4d):** yellow oil, 66.0 mg, 74% yield, (CH_2_Cl_2_ : CH_3_OH = 50:1). ^1^H NMR (400 MHz, CDCl_3_) δ 7.59 – 7.55 (m, 2H), 7.52 (d, *J* = 8.1 Hz, 2H), 7.41 (t, *J* = 7.6 Hz, 2H), 7.35 – 7.31 (m, 1H), 7.29 (d, *J* = 8.0 Hz, 2H), 3.89 (ddd, *J* = 12.3 Hz, 7.9 Hz, 2.9 Hz, 1H), 2.98 (s, 3H), 2.94 (s, 3H), 2.82 (qd, *J* = 13.6 Hz, 6.5 Hz, 2H), 2.63 – 2.40 (m, 2H), 1.96 – 1.89 (m, 1H), 1.86 – 1.73 (m, 1H). ^13^C NMR (101 MHz, CDCl_3_) δ 173.8, 141.1, 139.3, 137.9, 130.0, 128.8, 127.2, 127.2, 127.1, 72.7, 44.1, 37.5, 35.7, 31.2, 30.3. HRMS (ESI+) calculated m/z for C_19_H_23_NO_2_Na^+^ [M+Na]^+^ : 320.1621, found 320.1622.

**5-([1,1'-Biphenyl]-4-yl)-*N*-(*tert*-butyl)-4-hydroxypentanamide (4e):** yellow oil, 52.7 mg, 54% yield, (CH_2_Cl_2_ : CH_3_OH = 20:1). ^1^H NMR (400 MHz, CDCl_3_) δ 7.56 (dd, *J* = 5.1 Hz, 3.4 Hz, 2H), 7.51 (dd, *J* = 8.2 Hz, 1.7 Hz, 2H), 7.41 (t, *J* = 7.6 Hz, 2H), 7.34 – 7.29 (m, 1H), 7.28 – 7.24 (m, 2H), 3.94 – 3.82 (m, 1H), 2.84 – 2.73 (m, 2H), 2.28 (t, *J* = 6.8 Hz, 2H), 1.88 (dtd, *J* = 10.0 Hz, 6.9 Hz, 2.9 Hz, 1H), 1.78 – 1.65 (m, 1H), 1.32 (s, 9H). ^13^C NMR (101 MHz, CDCl_3_) δ 173.2, 141.0, 139.3, 137.8, 129.9, 128.8, 127.2, 127.2, 127.0, 72.4, 51.3, 44.0, 34.3, 32.0, 28.8. HRMS (ESI+) calculated m/z for C_21_H_27_NO_2_Na^+^ [M+Na]^+^ : 348.1934, found 348.1935.

**4-Hydroxy-5-phenylpentanenitrile (5a)**^10^**:** yellow oil, 40.4 mg, 77% yield, (CH_2_Cl_2_ : CH_3_OH = 20:1). ^1^H NMR (400 MHz, CDCl_3_) δ 7.32 (t, *J* = 7.3 Hz, 2H), 7.26 (d, *J* = 7.3 Hz, 1H), 7.21 – 7.17 (m, 2H), 3.96 – 3.87 (m, 1H), 2.82 (dd, *J* = 13.5 Hz, 4.5 Hz, 1H), 2.69 (dd, *J* = 13.5 Hz, 8.3 Hz, 1H), 2.50 (dd, *J* = 7.9 Hz, 6.5 Hz, 2H), 1.94 – 1.84 (m, 2H), 1.79 – 1.68 (m, 1H). ^13^C NMR (101 MHz, CDCl_3_) δ 137.4, 129.5, 128.9, 127.0, 119.9, 70.7, 44.1, 32.0, 13.9.

**5-([1,1'-Biphenyl]-4-yl)-4-hydroxypentanenitrile (5b):** yellow oil, 52.7 mg, 70% yield, (CH_2_Cl_2_ : CH_3_OH = 20:1). ^1^H NMR (400 MHz, CDCl_3_) δ 7.60 – 7.54 (m, 4H), 7.47 – 7.41 (m, 2H), 7.37 – 7.33 (m, 1H), 7.30 – 7.24 (m, 2H), 4.02 – 3.92 (m, 1H), 2.88 (dd, *J* = 13.6 Hz, 4.3 Hz, 1H), 2.74 (dd, *J* = 13.6 Hz, 8.5 Hz, 1H), 2.54 (dd, *J* = 8.0 Hz, 6.5 Hz, 2H), 1.99 – 1.89 (m, 1H), 1.85 – 1.74 (m, 2H).^13^C NMR (101 MHz, CDCl_3_) δ 140.8, 140.1, 136.4, 129.9, 128.9, 127.7, 127.5, 127.1, 119.9, 70.8, 43.8, 32.2, 14.0. HRMS (ESI+) calculated m/z for C_17_H_17_NONa^+^ [M+Na]^+^ : 274.1203, found 274.1202.

**4-Hydroxy-6-phenoxyhexanenitrile (5c):** yellow oil, 50.5 mg, 82% yield, (CH_2_Cl_2_ : CH_3_OH = 20:1). ^1^H NMR (400 MHz, CDCl_3_) δ 7.33 – 7.26 (m, 2H), 6.97 (t, *J* = 7.4 Hz, 1H), 6.90 (d, *J* = 7.9 Hz, 2H), 4.22 – 4.15 (m, 1H), 4.15 – 4.08 (m, 1H), 4.06 – 3.97 (m, 1H), 2.72 (s, 1H), 2.54 – 2.48 (m, 2H), 1.97 – 1.91 (m, 2H), 1.91 – 1.71 (m, 2H).^13^C NMR (101 MHz, CDCl_3_) δ 158.4, 129.7, 121.3, 120.0, 114.6, 68.5, 65.7, 36.3, 32.8, 13.7. HRMS (ESI+) calculated m/z for C_12_H_15_NO_2_Na^+^ [M+Na]^+^ : 228.0995, found 228.0995.

**6-Fluoro-4-hydroxyhexanenitrile (5d):** yellow oil, 26.7 mg, 68% yield, (CH_2_Cl_2_ : CH_3_OH = 20:1). ^1^H NMR (400 MHz, CDCl_3_) δ 4.79 – 4.64 (m, 1H), 4.63 – 4.50 (m, 1H), 3.98 (ddd, *J* = 12.5 Hz, 8.8 Hz, 3.5 Hz, 1H), 2.64 – 2.45 (m, 2H), 2.32 (s, 1H), 1.99 – 1.71 (m, 4H).^13^C NMR (101 MHz, CDCl_3_) δ 119.9, 81.6 (d, *J* = 163.3 Hz), 67.1 (d, *J* = 3.2 Hz), 37.6 (d, *J* = 18.9 Hz), 32.7, 13.7. ^19^F NMR (376 MHz, CDCl_3_) δ -221.8. HRMS (ESI+) calculated m/z for C_6_H_10_NOFNa^+^ [M+Na]^+^ : 154.0639, found 154.0641.

**Diethyl-(4-([1,1'-biphenyl]-4-yl)-3-hydroxybutyl)phosphonate (6a):** yellow oil, 81.5 mg, 75% yield, (CH_2_Cl_2_ : CH_3_OH = 20:1). ^1^H NMR (400 MHz, CDCl_3_) δ 7.60 – 7.52 (m, 4H), 7.47 – 7.39 (m, 2H), 7.36 – 7.31 (m, 1H), 7.30 – 7.22 (m, 2H), 4.21 – 4.01 (m, 4H), 3.98 – 3.82 (m, 1H), 2.81 (qd, *J* = 13.6 Hz, 6.4 Hz, 2H), 2.53 (s, 1H), 2.08 – 1.67 (m, 4H), 1.32 (td, *J* = 7.1 Hz, 2.5 Hz, 6H). ^13^C NMR (101 MHz, CDCl_3_) δ 141.0, 139.6, 137.4, 130.0, 128.9, 127.4, 127.3, 127.1, 72.5 (d, J = 13.3 Hz), 61.8 (dd, J = 6.4, 3.6 Hz), 43.6, 29.6 (d, J = 4.7 Hz), 22.3 (d, J = 141.7 Hz), 16.6 (d, J = 5.9 Hz). ^31^P NMR (162 MHz, CDCl_3_) δ 32.9. HRMS (ESI+) calculated m/z for C_20_H_27_O_4_PNa^+^ [M+Na]^+^ : 385.1540, found 385.1541.

**Diethyl-(3-hydroxy-5-(thiophen-2-yl)pentyl)phosphonate (6b):** yellow oil, 61.5 mg, 67% yield, (CH_2_Cl_2_ : CH_3_OH = 20:1). ^1^H NMR (400 MHz, CDCl_3_) δ 7.11 (dd, *J* = 5.1 Hz, 1.2 Hz, 1H), 6.91 (dd, *J* = 5.1 Hz, 3.4 Hz, 1H), 6.80 (dd, *J* = 3.2 Hz, 0.7 Hz, 1H), 4.20 – 3.95 (m, 4H), 3.76 – 3.64 (m, 1H), 3.13 – 2.80 (m, 3H), 1.99 – 1.64 (m, 6H), 1.32 (td, *J* = 7.1 Hz, 2.2 Hz, 6H). ^13^C NMR (101 MHz, CDCl_3_) δ 145.0, 126.9, 124.4, 123.1, 70.4 (d, *J* = 12.6 Hz), 61.9 (t, *J* = 6.1 Hz), 39.2, 30.3 (d, *J* = 4.8 Hz), 26.3, 22.9, 21.5, 16.6 (d, *J* = 5.9 Hz). ^31^P NMR (162 MHz, CDCl_3_) δ 33.9. HRMS (ESI+) calculated m/z for C_13_H_23_O_4_PSNa^+^ [M+Na]^+^ : 329.0947, found 329.0945.

**Diethyl-(5-fluoro-3-hydroxypentyl)phosphonate (6c):** yellow oil, 30.5 mg, 42% yield, (CH_2_Cl_2_ : CH_3_OH = 20:1). ^1^H NMR (400 MHz, CDCl_3_) δ 4.63 (ddtd, *J* = 19.8 Hz, 14.5 Hz, 9.2 Hz, 4.9 Hz, 2H), 4.19 – 4.00 (m, 4H), 3.92 – 3.85 (m, 1H), 3.25 (s, 1H), 1.96 – 1.64 (m, 6H), 1.33 (t, *J* = 7.0 Hz, 6H). ^13^C NMR (101 MHz, CDCl_3_) δ 81.65 (d, *J* = 162.9 Hz), 68.03 (dd, *J* = 12.6 Hz, 4.3 Hz), 61.90 (t, *J* = 5.1 Hz), 37.77 (d, *J* = 19.1 Hz), 30.46 (d, *J* = 4.8 Hz), 22.20 (d, *J* = 141.7 Hz), 16.55 (d, *J* = 5.9 Hz). ^19^F NMR (376 MHz, CDCl_3_) δ -220.5. ^31^P NMR (162 MHz, CDCl_3_) δ 33.7. HRMS (ESI+) calculated m/z for C_9_H_20_O_4_FPNa^+^ [M+Na]^+^ : 265.0976, found 265.0975.

**4. Mechanism experiments**

***Standard condition*** refers to “To a 25 mL sealed tube was added Ir[(dFCF_3_ppy)_2_dtbbpy]PF_6_ (3.4 mg, 1 mol%), quinucidine (6.7 mg, 20 mol%), and DBU (22.8 mg, 50 mol%). The tube was degassed by vacuum evacuation and back-filled with CO_2_ for three times. Then anhydrous CH_3_CN (1 mL) and 50 μL H_2_O was added into the tube use syringe. Afterwards, the corresponding alcohol (0.45 mmol - 0.6 mmol, 1.5 equiv. - 2.0 equiv.) and compound **2a** or **2c** (0.3 mmol) were added through a micro-syringe. Before the reaction starts, CO_2_ was bubbled into the solution for 2 minutes. Then the reaction tube was sealed and stirred at 40 ^o^C for 12 h under the irradiation of 15 W blue LED lamps. After completion of the reaction, it was carefully quenched with 2 M HCl (or H_2_O) and the mixture was extracted 3 times with CH_2_Cl_2_. The combined organic layers were dried over anhydrous Na_2_SO_4_ and concentrated under reduced pressure. The residual product was directly investgated by crude ^1^H NMR or purified by flash chromatography on silica gel to afford the corresponding product”

1. **Radical-inhibition experiment**

**Scheme S1.** After standard conditions were carried out, phenylethanol **1a** (0.6 mmol, 73.2 mg, 2.0 equiv.) and methyl acrylate **2a** (0.3 mmol, 25.8 mg) was added into the sealed tube. After that, 2,2,6,6-Tetramethylpiperidinooxy (TEMPO) (0.9 mmol, 140.6 mg, 3.0 equiv.) was added. The mixture was stirred at 40 ^o^C for 12 h under 15 W blue LED. Next, this reaction was extracted with CH_2_Cl_2_ 3 times after quenched by HCl (2 M). The combined organic phase was dried by anhydrous Na_2_SO_4_ and evaporated in vacuum. CH_2_Br_2_ (10.5 μL, 0.15 mmol) was added to the residual as an internal standard. Product **3a** was not detected by ^1^H NMR, and starting materials **1a** remained, proving the formation of radical intermediates, and the Giese-type radical addition process was inhibited.

1. **Radical clock experiment**

**Scheme S2.** After standard conditions were carried out, (2-phenylcyclopropyl) methanol **11** (0.6 mmol, 88.9 mg, 2.0 equiv.) and methyl acrylate **2a** (0.3 mmol, 25.8 mg) was added into the sealed tube. The mixture was stirred at 40 ^o^C for 12 h under 15 W blue LED. Next, this reaction was extracted with CH_2_Cl_2_ 3 times after quenched by HCl (2 M). The combined organic phase was dried by anhydrous Na_2_SO_4_ and evaporated in vacuum. CH_2_Br_2_ (10.5 μL, 0.15 mmol) was added to the residual as an internal standard. The ring-opening product 4-phenylbutanal **12** was detected in 19% yield by crude ^1^H NMR, while the desired product **13** was trace amount. This results are the direct evidence for the formation of α-OH carbon radical intermediate.


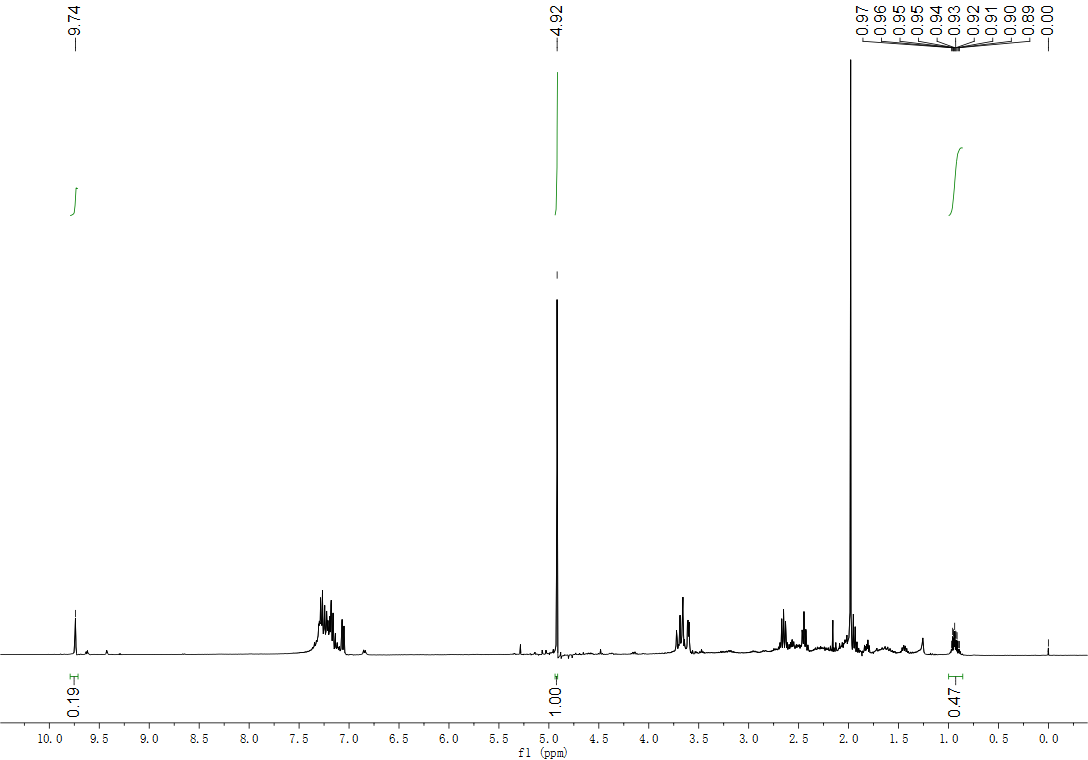


crude ^1^H NMR (400 MHz, CDCl_3_) spectrum of radical clock experiment

1. **Deuterium incorporation experiments**

① Synthesis of deuterium starting material ***d*-1c**

**Scheme S3.** LiAlD_4_ powder (252 mg, 6 mmol, 1.2 equiv.) was added to a stirred cold suspension (ice bath) of ethyl 2-(4-methoxyphenyl)acetate (5 mmol, 970.5 mg) in dry THF (20 mL) under N_2_ atmosphere.. Next, the mixture was warmed slowly to the refluxing temperature for 4 hours. After complete transform detected by GC-MS of the solution, 1 mL CH_3_OH was added to quench the reaction. Aq. solution of NaOH (10%, 15 mL) was then added to the mixture. Then, THF was evaporated from the filtrate in vacuum. Next, this reaction was extracted with CH_2_Cl_2_ (3 x 15 mL). The combined organic phase was dried by anhydrous Na_2_SO_4_ and evaporated in vacuum. The crude residue was purified by flash chromatography (petroleum ether/ethyl acetate = 20:1) to provide deuterium starting material ***d*-1c** in almost quantitive isolated yield as a colorless oil.

**2-(4-Methoxyphenyl)ethan-1,1-*d_2_*-1-ol** (***d*-1c**)^11^**:** colorless oil, 764.0 mg, quantitave yield (CH_2_Cl_2_: EA = 40:1). ^1^H NMR (400 MHz, CDCl_3_): δ 7.12 (d, *J* = 8.6 Hz, 2H), 6.88 – 6.80 (m, 2H), 3.77 (s, 3H), 2.76 (s, 2H), 1.95 (s, 1H).^13^C NMR (101 MHz, CDCl_3_): δ 158.3, 130.6, 130.0, 114.0, 63.1 (dt, *J* = 43.3 Hz, 21.7 Hz), 55.3, 38.1.

**
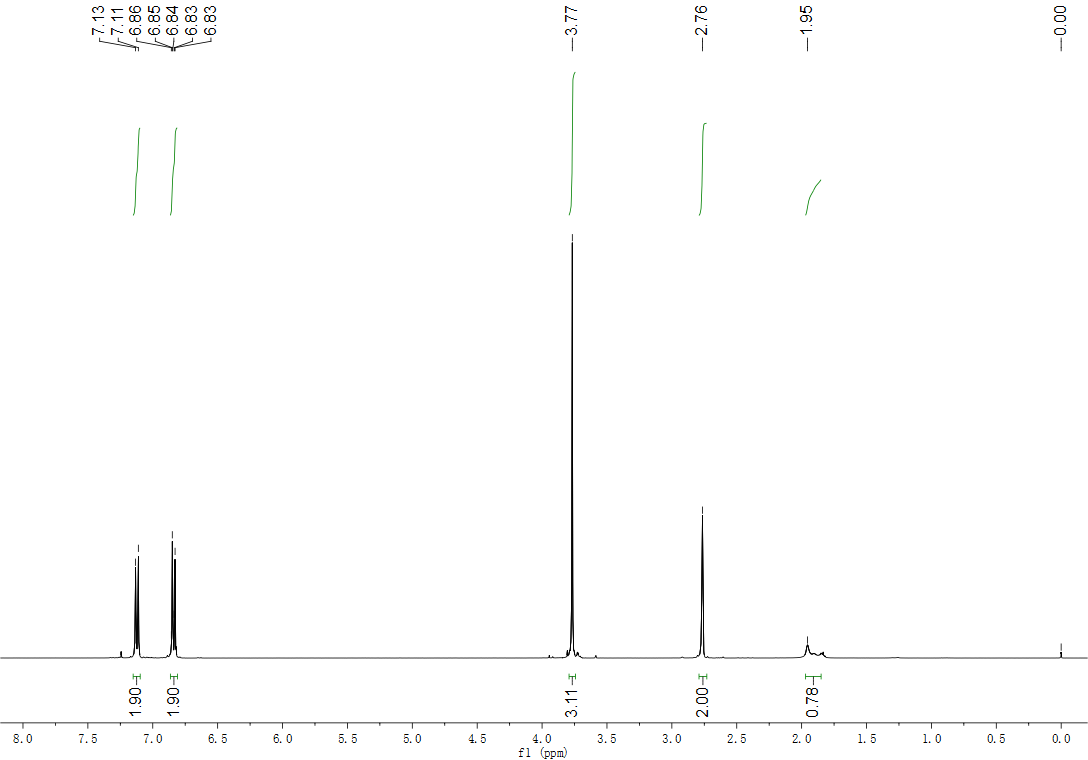
**

^1^H NMR (400 MHz, CDCl_3_) spectrum of ***d*-1c**

^
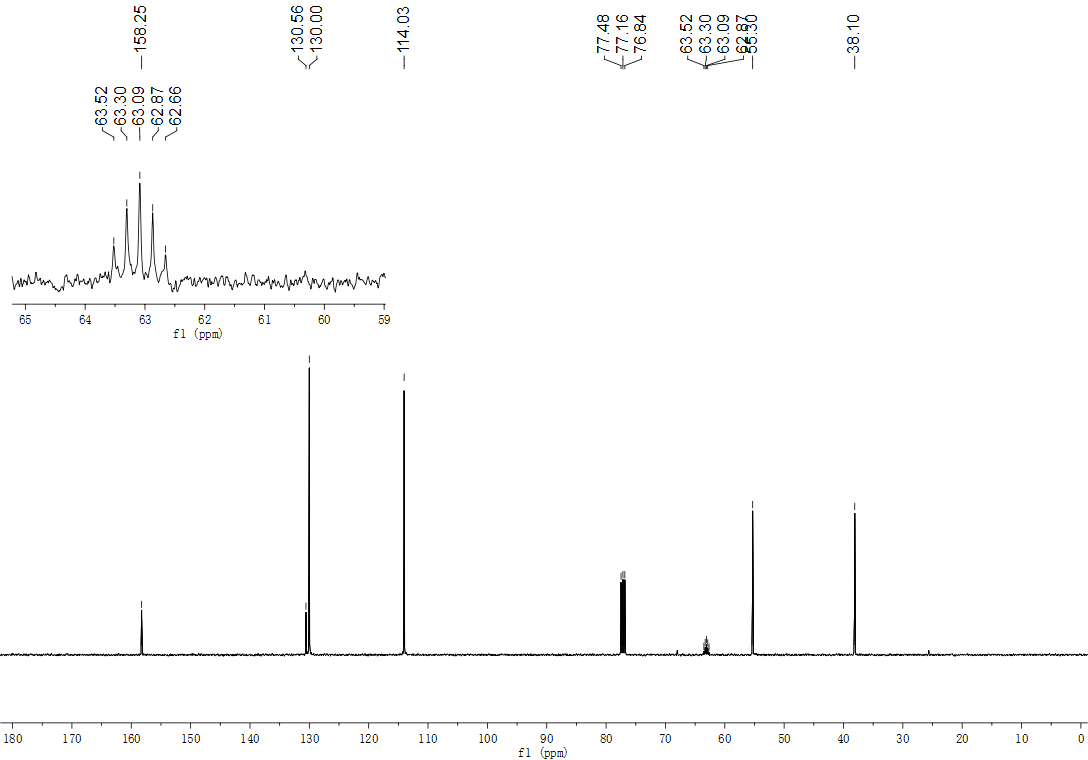
^

^13^C{^1^H} NMR (101 MHz, CDCl_3_) spectrum of ***d*-1c**

② Deuterium incorporation experiment A

**Scheme S4.** Standard conditions were carried out except for the H_2_O was replaced by D_2_O. The mixture was extracted with CH_2_Cl_2_ 3 times after quenched by H_2_O. The combined organic phase was dried by anhydrous Na_2_SO_4_ and evaporated in vacuum. The crude residue was purified by flash chromatography (petroleum ether/ethyl acetate = 10:1) to provide product ***d*-3c** in 57% isolated yield, with 73% and 62% deuteration rates on the α position of the ester group in ***d*-3c**.

*This experiment confirmed that H_2_O is the main hydrogen source in this system. Due to the strong alkalinity of DBU, the α-H for the ester group of ***d*-3c** may also undergo rapid proton exchange, resulting in two deuterium substitution sites in the product. In order to exclude the possible effects from α-H of the hydroxyl group in alcohol **1c**, control experiment with ***d*-1c** was then conducted and the results are disscussed in part ③.

**5-(4-Methoxybenzyl)dihydrofuran-2(3*H*)-one-3,3-*d_2_*** (***d*-3c**)**:** yellow oil, 35.6 mg, 57% yield (PE : EA = 20:1). ^1^H NMR (400 MHz, CDCl_3_): δ 7.15 (d, *J* = 8.6 Hz, 2H), 6.85 (d, *J* = 8.6 Hz, 2H), 4.73 – 4.67 (m, 1H), 3.80 (s, 3H), 3.00 (dd, *J* = 14.1 Hz, 5.9 Hz, 1H), 2.88 (dd, *J* = 14.1 Hz, 6.2 Hz, 1H), 2.51 – 2.38 (m, 0.38H), 2.37 – 2.30 (m, 0.27H), 2.29 – 2.18 (m, 1H), 1.99 – 1.87 (m, 1H). ^13^C NMR (101 MHz, CDCl_3_): δ 177.3, 158.7, 130.6, 127.9, 114.2, 81.1, 55.4, 40.5, 27.0 (t, *J* = 6.1 Hz).

**
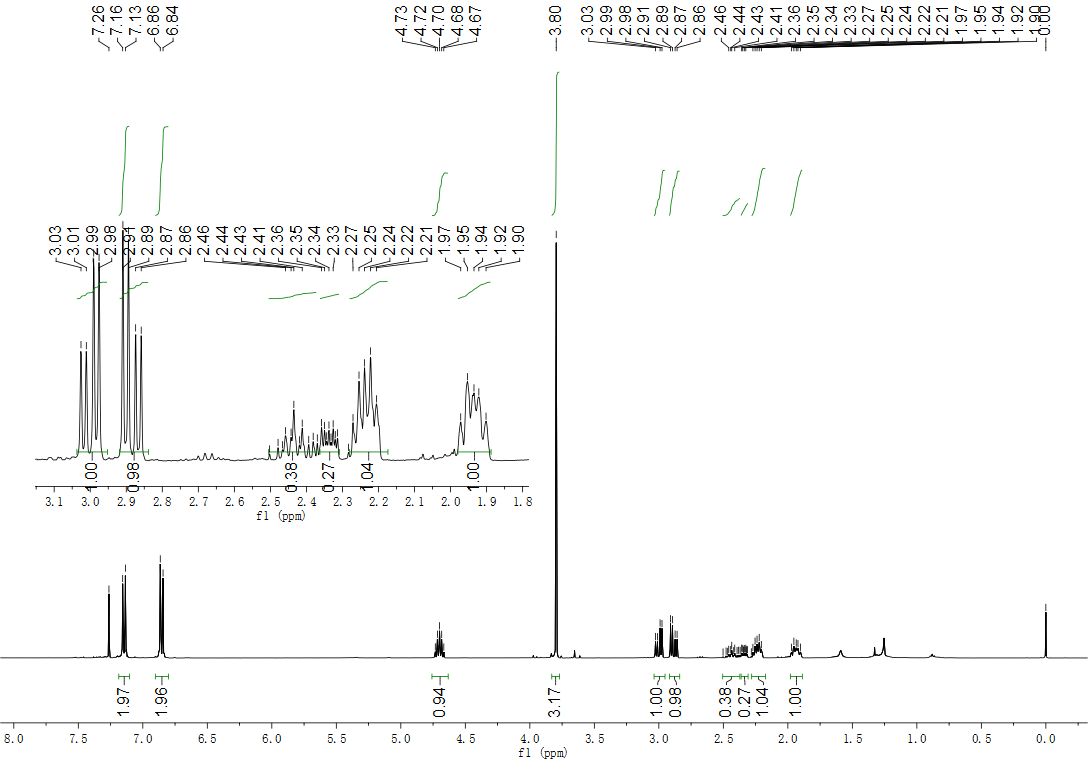
**

^1^H NMR (400 MHz, CDCl_3_) spectrum of ***d*-3c**

^
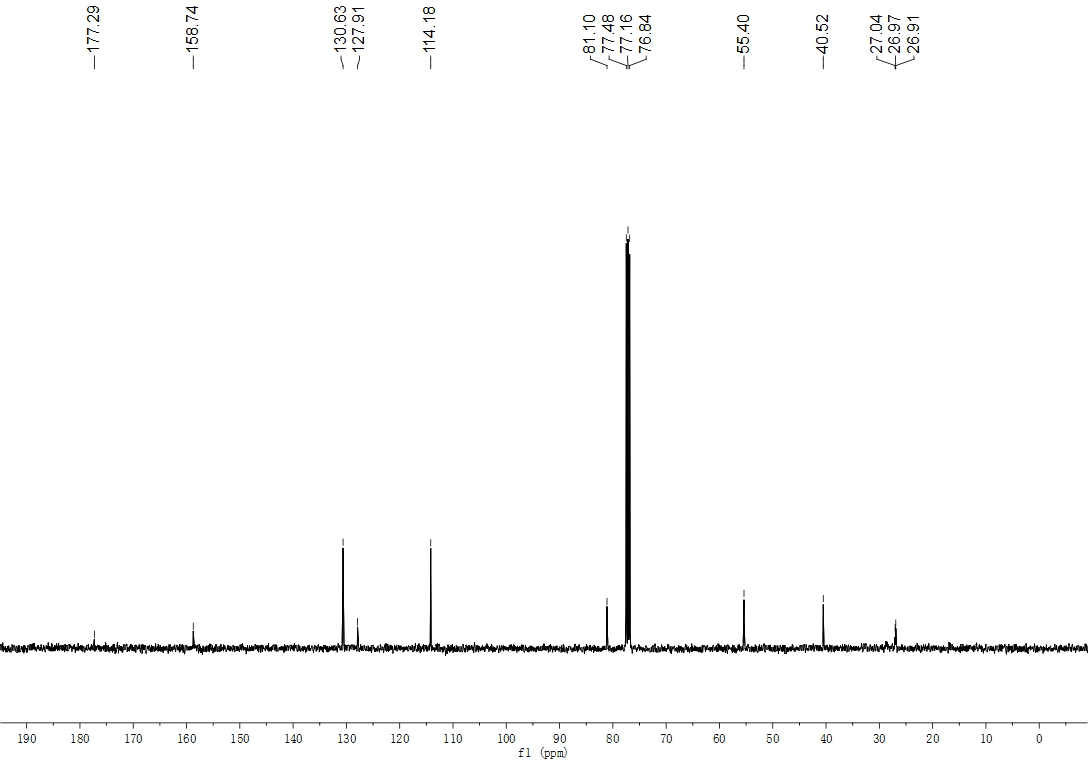
^

^13^C{^1^H} NMR (101 MHz, CDCl_3_) spectrum of ***d*-3c**

③ Deuterium incorporation experiment B

**Scheme S5.** Standard conditions were carried out with ***d*-1c** and **2a** as the starting materials. The mixture was extracted with CH_2_Cl_2_ 3 times after quenched by H_2_O. The combined organic phase was dried by anhydrous Na_2_SO_4_ and evaporated in vacuum. The crude residue was purified by flash chromatography (petroleum ether/ethyl acetate = 10:1) to provide product ***d*-3c'** in 46% isolated yield, with no deuteration on the α position of the ester group in ***d*-3c'**. This experiment confirmed that alcohol is not the hydrogen source for this reaction.

**5-(4-Methoxybenzyl)dihydrofuran-2(3*H*)-one-3,3-*d_2_*** (***d*-3c'**)**:** yellow oil, 26.1 mg, 42% yield (PE : EA = 20:1). ^1^H NMR (400 MHz, CDCl_3_): δ 7.14 (d, *J* = 8.6 Hz, 2H), 6.85 (d, *J* = 8.6 Hz, 2H), 3.79 (s, 3H), 2.98 (d, *J* = 14.1 Hz, 1H), 2.87 (d, *J* = 14.1 Hz, 1H), 2.49 – 2.29 (m, 2H), 2.26 – 2.11 (m, 1H), 1.93 (dt, *J* = 12.8 Hz, 9.2 Hz, 1H). ^13^C NMR (101 MHz, CDCl_3_): δ 177.2, 158.7, 130.6, 127.9, 114.1, 80.6 (t, *J* = 23.2 Hz), 80.4, 55.3, 40.3, 28.7, 26.9. HRMS (ESI+) calculated m/z for C_12_H_13_DO_2_Na^+^ [M+Na]^+^ : 230.0903, found 230.0899.

**
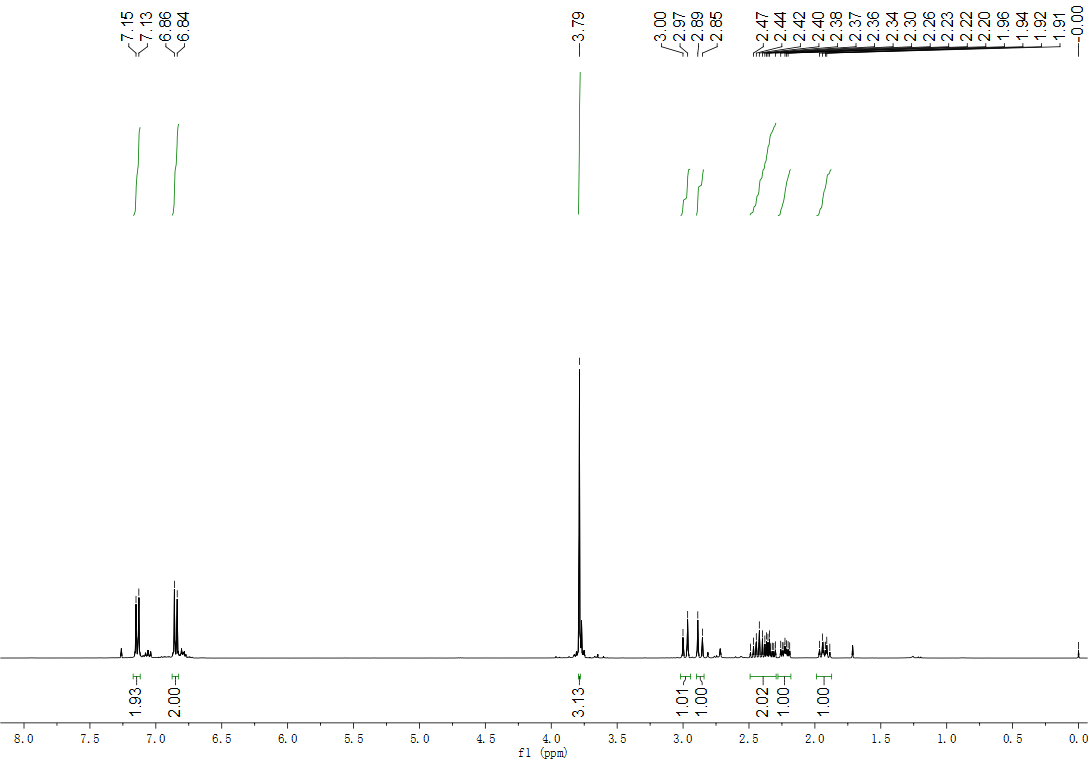
**

^1^H NMR (400 MHz, CDCl_3_) spectrum of ***d*-3c'**

^
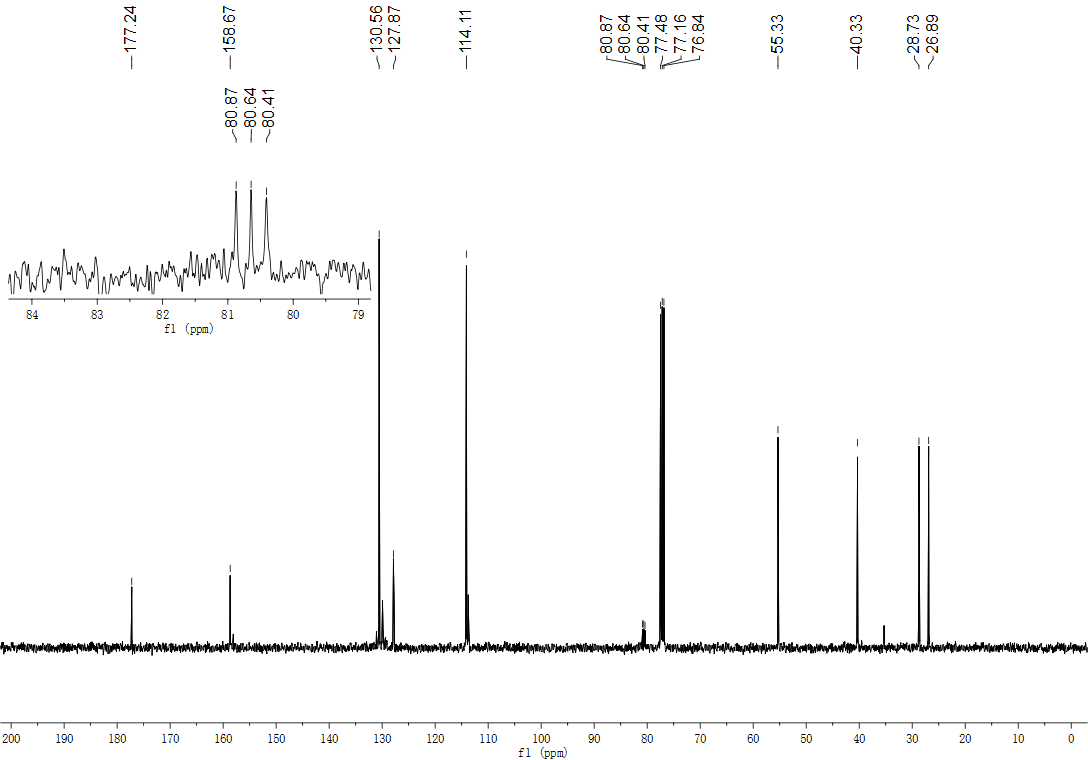
^

^13^C{^1^H} NMR (101 MHz, CDCl_3_) spectrum of ***d*-3c'**

1. **Parallel kinetic isotopic effect experiment**

**Scheme S6.** The reaction was started immediately, and then the reaction solution was stoped at reaction times of 10 min, 20 min, 1 h, and 2 h. The mixture was extracted with CH_2_Cl_2_ 3 times after quenched by H_2_O. The combined organic phase was dried by anhydrous Na_2_SO_4_ and evaporated in vacuum. The crude residue was investigated directly by ^1^H NMR, CH_2_Br_2_ as the internal standard to analyse the yield of **5e** and ***d*-5e**. The results were listed as following. The ***KIE*** was determined to be 2.1 using theequation: ***KIE*** = *k*_H_/*k*_D_.

**
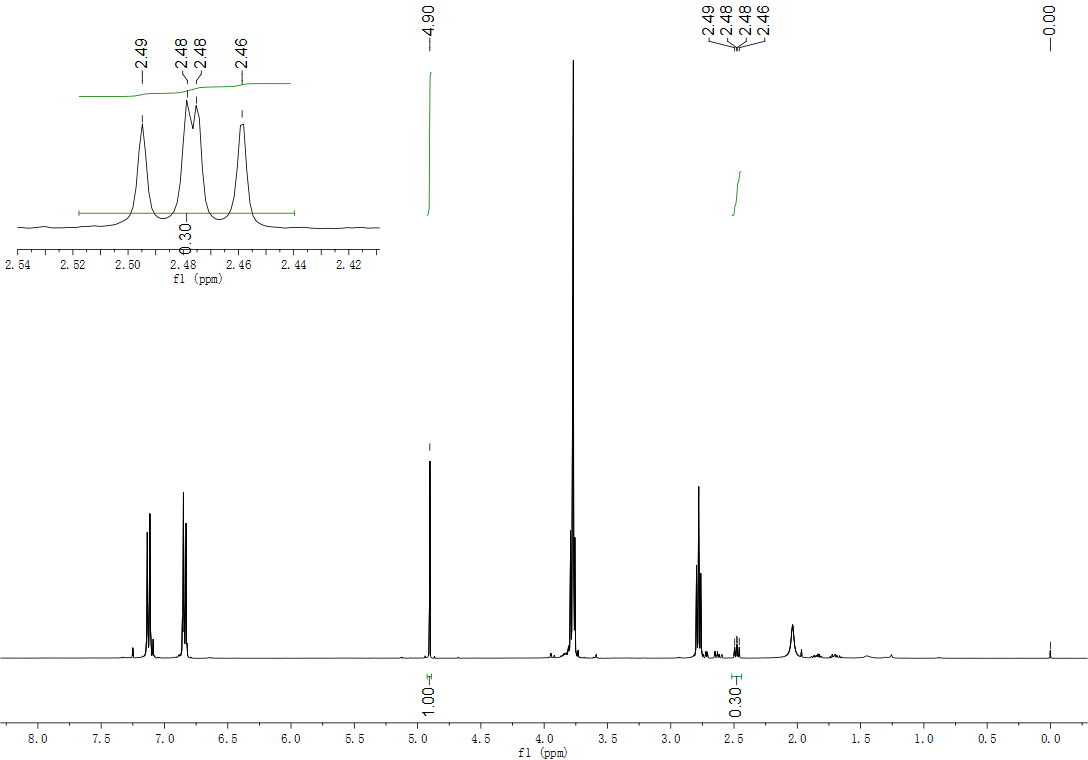
**

crude ^1^H NMR (400 MHz, CDCl_3_) spectrum of **5e**

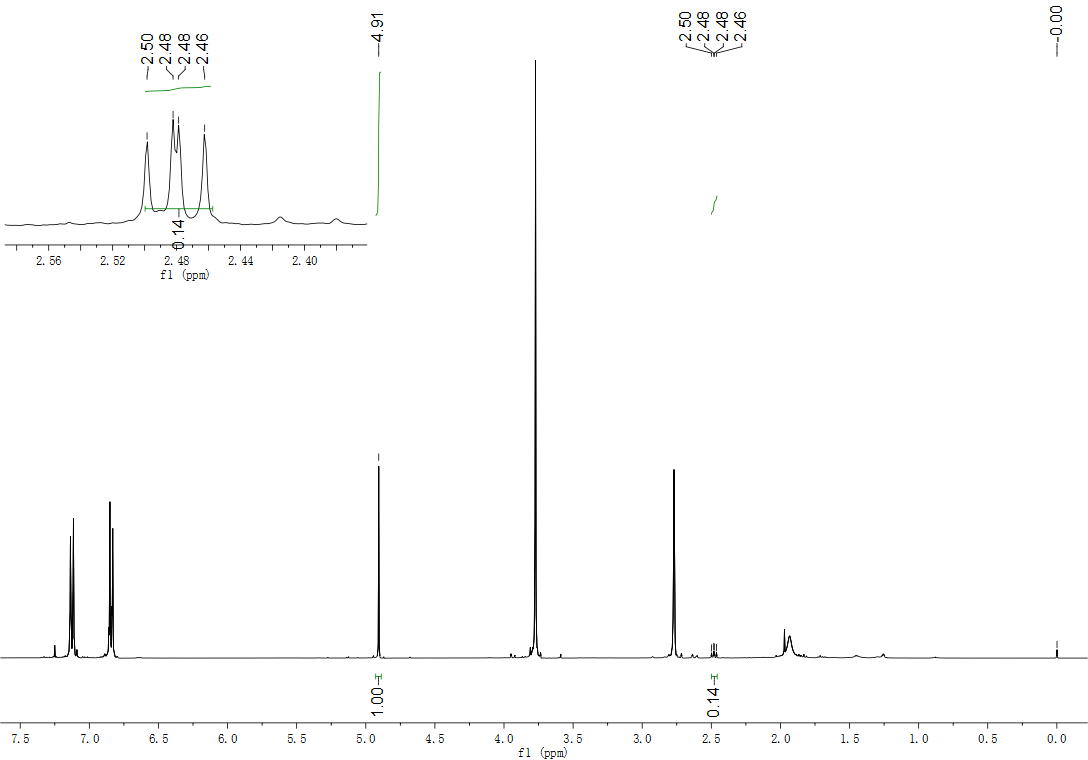


crude ^1^H NMR (400 MHz, CDCl_3_) spectrum of ***d*-5e**

1. **Identification of key intermediate**

① Synthesis and identification of DBU-CO_2_ additive

**Scheme S7.** To a 4 mL vial equipped with a magnetic barrel was added DBU (0.8 mmol, 107 μL) and 2 mL of hexane. The solution was stirred for 2 h with continuous CO_2_ bubbling. The resulting white precipitate was filtered off, washed with cold petroleum ether and dried in vacuo to afford pure adduct **14** as a white powder (125 mg, 80% yield).

**(2,3,4,6,7,8,9,10-Octahydro-1*H*-pyrimido[1,2-*a*]azepin-5-ium-1-yl)(1-oxidaneyl)methanolate** (**14**)^12,13^**:** white powder, 125.0 mg, 80% yield. ^1^H NMR (400 MHz, CDCl_3_): δ 3.35 – 3.28 (m, 2H), 3.26 – 3.18 (m, 4H), 2.53 – 2.43 (m, 2H), 1.91 – 1.79 (m, 2H), 1.72 – 1.65 (m, 4H), 1.62 – 1.55 (m, 2H). ^13^C NMR (101 MHz, CDCl_3_): δ 162.5, 77.4, 53.2, 48.5, 43.2, 36.5, 29.8, 28.4, 25.8, 22.1.

**
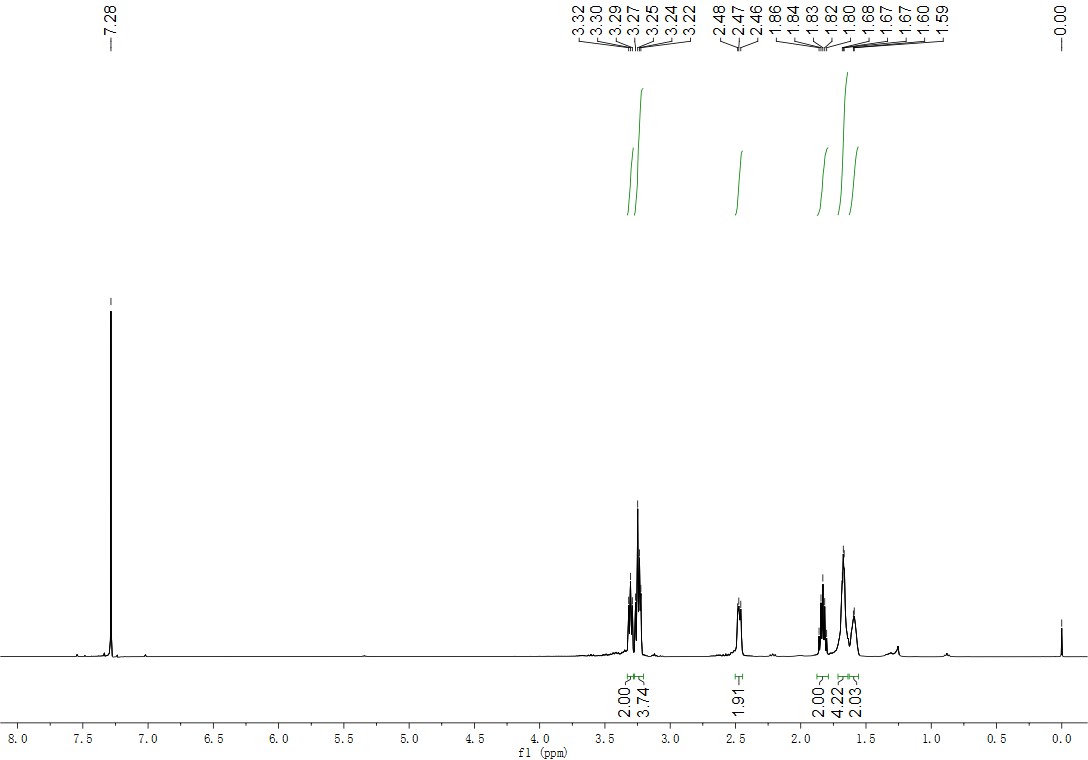
**

^1^H NMR (400 MHz, CDCl_3_) spectrum of **14**

^
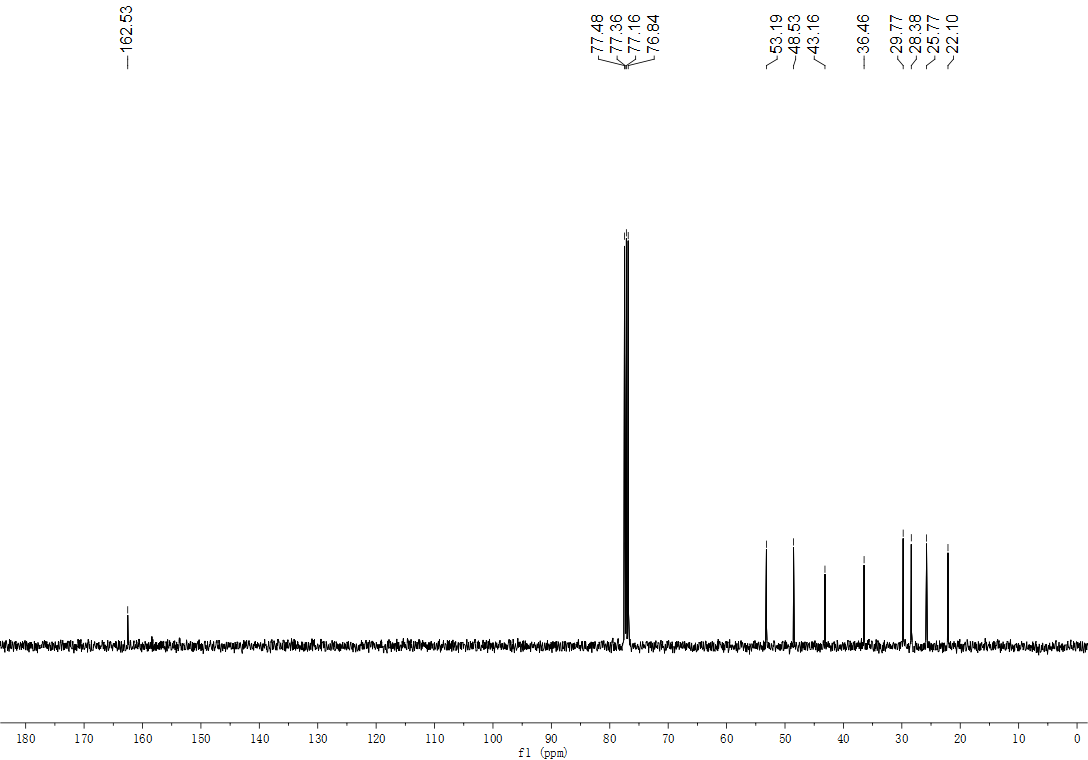
13^C{^1^H} NMR (101 MHz, CDCl_3_) spectrum of **14**

**
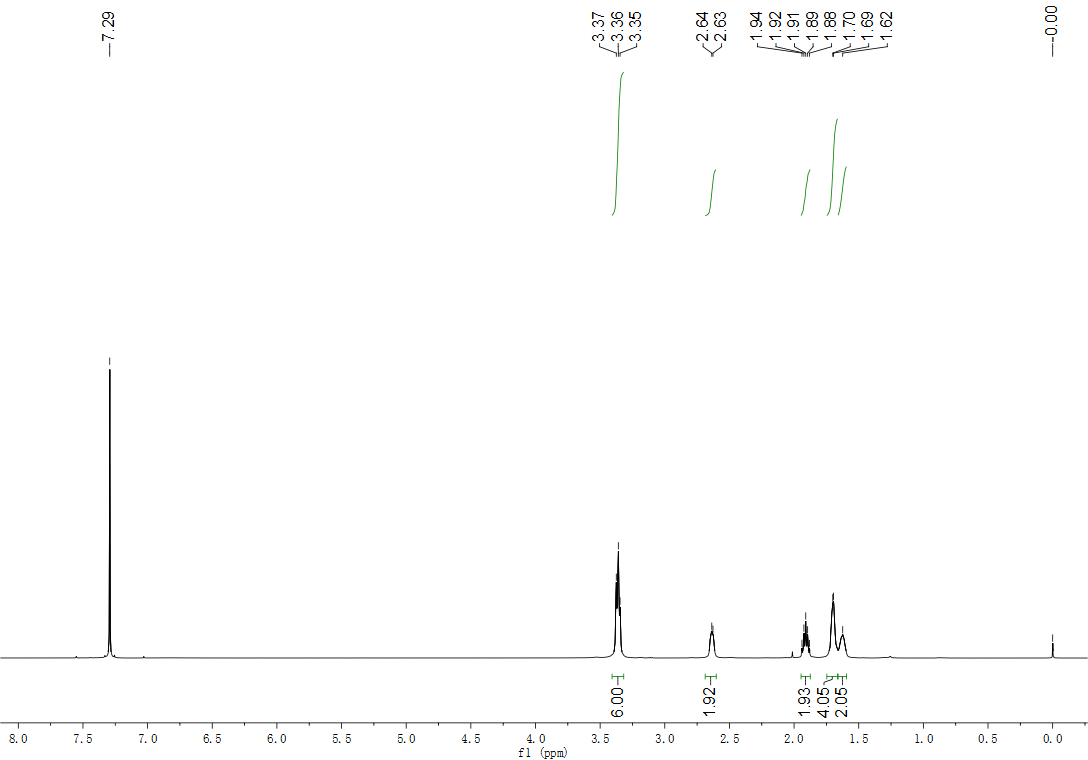
**

^1^H NMR (400 MHz, CDCl_3_) spectrum of **14** after 1 week in air

****^
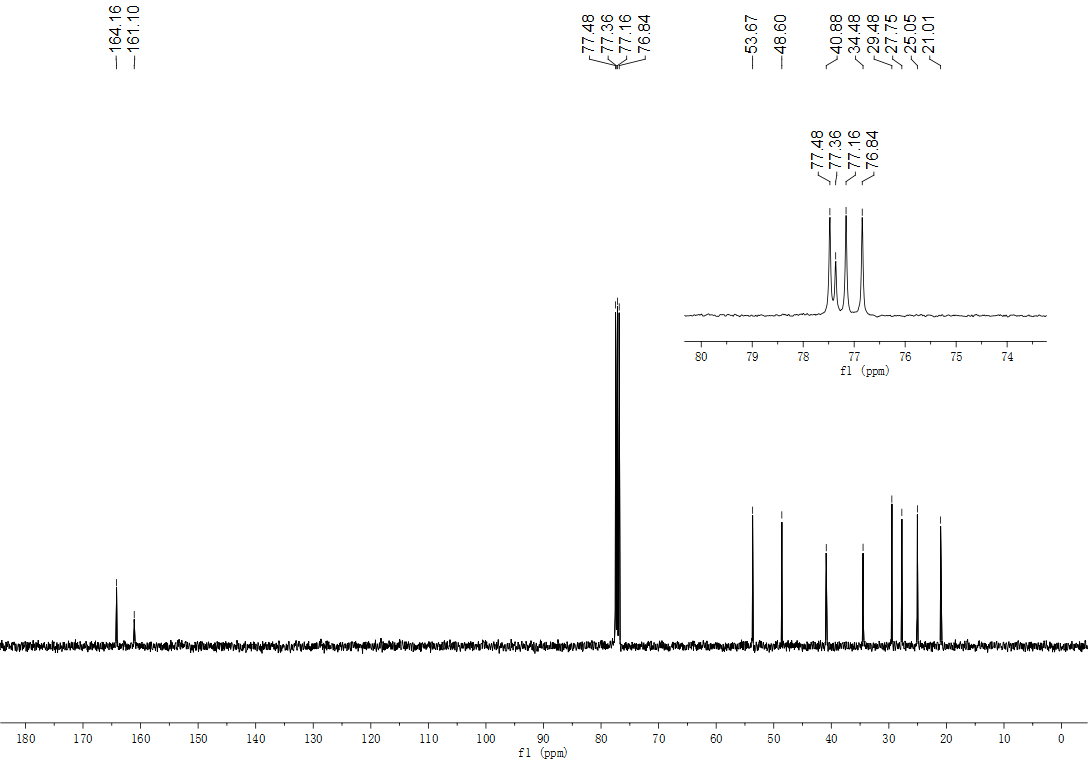
^

^13^C{^1^H} NMR (101 MHz, CDCl_3_) spectrum of **14** after 1 week in air

As shown in the ^1^H and ^13^C{^1^H} NMR spectrum of **14** after 1 week in air, after the newly prepared DBU-CO_2_ adduct was exposed to air for 1 week, the ^13^C NMR data for the DBU-CO_2_ adduct sample showed two signals at 161.1 ppm and 164.2 ppm corresponding to a carbamic and an amidinium carbon, respectively. This change confirmed that this complex could be associated with water, probably by a hydrogenbond interaction with the CO_2_ moiety, leading to the carbamic zwitterion [DBUH^+^][HCO_3_^-^] species. As a result, we suppose that the dynamic equilibrium of DBU-CO_2_-H_2_O in the system promotes the HAT process of alcohols.^13,14^

② Control experiments using DBU-CO_2_ additive as catalyst

*^a^*Crude ^1^H NMR yields. *^b^*Reaction under 1 atm CO_2._

**Scheme S8** Control experiments using DBU-CO_2_ additive as catalyst

**5. Details and notes on DFT calculations**

Geometry optimization and frequency analysis of all reagents, intermediates and transition states were done by Gaussian 16B package with hybrid functional B3LYP and Ahlrichs’ basis set def2-SVP; Grimme's dispersion correction methods of D3 with Becke-Johnson damping versions(GD3BJ) and PCM continuum solvation models were utilized to include weak interactions and implicit solvation effect.^15^ Based on structures at minimum or saddle points, single point energy were done by ORCA 6.0 package with double-hybrid functional PWBP95 and Ahlrichs’ basis set def2-TZVPP; Grimme's dispersion correction methods of D4 with Becke-Johnson damping versions(GD4BJ) and SMD continuum solvation models were utilized to include weak interactions and implicit solvation effect; resolution-of-the-identity (RI) approximation with auxiliary basis of def2/J and def2-TZVPP/C was utilized to accelerate the PWBP95-D4(BJ)/def2-TZVPP calculation.^16^ All stable species were optimized convergent and confirmed no imaginary frequency; all transition states were only one imaginary frequency with matching vibration mode. For all of reagents, intermediates and transition states, the numbers of $\Delta G$ are sum of thermal correction to free energy calculated in 298.15K, single point energy and +1.89 kcal/mol for the difference between standard conditions of gas phase and solution. In systems exhibiting complex hydrogen-bonding networks involving water, alcohols, and other donors and acceptors, the precise configuration of hydrogen-bonded complexes becomes inherently challenging to resolve. To circumvent complications arising from solvation energy and entropic effects, our computational analysis exclusively evaluates thermodynamic and kinetic parameters within individual hydrogen-bonded complex systems, employing consistent solvation models for comparative assessment across reactions. Scheme 4a presents models considering either a single water or alcohol molecule within the reactive complex, while Scheme 4b intentionally excludes water participation. Scheme S9 and S10 details alternative pathways incorporating explicit water-mediated reactions of DBU-CO_2_ system and HAT processes, modeled through cyclic hydrogen-bonded complexes. Comparative analysis between Schemes S9, S10 and Scheme4 demonstrates that water inclusion during the HAT step negligibly impacts qualitative conclusions, thereby validating the computational efficiency of simplified model in Scheme 4.

**Scheme S9.** Reactions of DBU-CO_2_ system calculated with consideration of hydrogen bonding networks

**Scheme S10.** HAT reactions calculated with consideration of hydrogen bonding networks

Below are all the geometric coordinates involved in the calculation section:

**DBU...HOBu...CO_2_**

**Denoted by Complex1-1**

0 1

C 1.62081400 1.56781800 -1.31464900

C 3.26152600 -0.06257400 0.48962600

C 0.80344200 -0.47660700 0.04069900

C 0.83185200 0.25169600 -1.29230400

H 4.00577600 -0.69996800 0.98777100

H 3.28964100 0.90901400 1.02148200

H 1.21378300 -0.43516500 -2.06636000

H -0.21817700 0.43505300 -1.54171900

N 1.97036500 -0.72458400 0.72049100

N -0.37424100 -0.85737400 0.43998300

C 1.90007600 -1.40335400 2.01827800

C 0.70833400 -2.34324200 2.08444500

H 1.84545900 -0.65588500 2.83323600

H 2.84004700 -1.95871500 2.15905500

C -0.54289900 -1.57042100 1.69459100

H 0.86077500 -3.18073800 1.38306800

H 0.62116700 -2.76893500 3.09557000

H -1.40829600 -2.24408600 1.59448000

H -0.80430100 -0.84962300 2.49317900

C -2.04218700 -2.42566500 -1.49252100

O -2.64563900 -2.85123000 -0.59293500

O -1.44921100 -2.06232200 -2.42497600

C -0.75128400 3.86389600 1.01524200

C -1.70032700 2.98875000 0.19937300

H 0.02665500 3.25238400 1.50319000

C -3.26808500 0.95897100 0.21739200

H -3.77967800 0.25752400 0.91075700

H -4.06673400 1.51512700 -0.30812900

O -2.52472300 0.26438500 -0.75602700

H -1.73816500 -0.15954400 -0.29691300

C -2.42461900 1.93651200 1.03955100

H -3.07697700 2.43019700 1.78292800

H -1.67905200 1.36111400 1.61697800

H -0.24103100 4.60901400 0.38395300

H -1.28750100 4.40877200 1.81051900

H -2.44665600 3.62101600 -0.31558100

H -1.13654400 2.47637800 -0.59592600

C 3.69171200 0.12535900 -0.96411400

C 3.10464800 1.36928600 -1.63912100

H 3.43149500 -0.78704400 -1.52421300

H 4.79130200 0.18724800 -0.98193300

H 3.23918000 1.27855800 -2.73013800

H 3.67130000 2.26369500 -1.33019600

H 1.16681900 2.24568900 -2.05431300

H 1.50542400 2.07342300 -0.34148500

**DBU...HOH...CO_2_**

**Denoted by Complex1-2**

0 1

N 1.99176600 0.69476600 0.16170200

N -0.34544500 0.91251300 0.52033800

C 2.10037400 2.00781900 -0.46952700

C 1.02146600 2.93912300 0.06400700

H 2.01727000 1.91764300 -1.57022300

H 3.10213000 2.41285900 -0.25745700

C -0.33443400 2.24854200 -0.05119000

H 1.23104100 3.16806300 1.12272700

H 1.03508300 3.89098300 -0.48910500

H -1.11782100 2.84952500 0.44103200

H -0.63324600 2.19191600 -1.11597800

O -5.67438800 -0.75777400 -0.34291400

C -2.82459600 -0.16668700 0.02739900

O -3.20094200 0.89157300 -0.28376800

O -2.52626000 -1.25462600 0.31168500

C 0.76102900 0.24611100 0.58436100

C 0.69086500 -1.16144800 1.13732900

C 0.82607000 -2.24972400 0.05645100

H 1.45579300 -1.31179600 1.91786100

H -0.29117900 -1.24291000 1.61582600

C 3.16095300 -0.17680500 0.08703700

C 2.26439400 -2.54225900 -0.37560700

H 0.21516400 -1.95654000 -0.81474300

H 0.37947500 -3.18040000 0.44314500

C 3.03975400 -1.33646900 -0.91163300

H 3.40844500 -0.57380400 1.08711800

H 4.00494200 0.46437200 -0.20162200

H 2.81526300 -2.95048500 0.49181800

H 2.25964700 -3.33980500 -1.13757000

H 4.05697700 -1.66427000 -1.18428600

H 2.57333300 -0.95564300 -1.83717300

H -5.51840100 -1.05216500 -1.25199800

H -5.54069000 0.19890100 -0.40543700

**DBU...HOBu...CO_2_**

**Denoted by TS1-1**

0 1

C -1.48005000 2.20328000 0.18911500

C -3.70183100 0.29952200 -0.19211700

C -1.28256900 -0.36766500 0.08654400

C -0.89842900 0.91849700 0.79220400

H -4.60580800 -0.32260700 -0.13738400

H -3.75132600 0.82756900 -1.16353700

H -1.18405500 0.83287400 1.85390200

H 0.19617100 0.94764100 0.77770100

N -2.57953800 -0.65322400 -0.20706300

N -0.29051800 -1.17507100 -0.17704100

C -2.88372100 -1.90199200 -0.91806500

C -1.89253700 -2.99611200 -0.55589300

H -2.88035600 -1.72054000 -2.00903600

H -3.90590900 -2.20094800 -0.64478900

C -0.48232600 -2.47920100 -0.79047600

H -2.01951000 -3.26862900 0.50475000

H -2.09122400 -3.89557800 -1.15677500

H 0.27060400 -3.16283800 -0.36762900

H -0.27327400 -2.41022800 -1.87389600

C 3.05843200 -1.85990700 0.99956100

O 4.17362700 -1.77370900 0.59473800

O 2.24477700 -2.37919700 1.69610600

C 2.52065400 3.83509800 -0.34549400

C 2.87275500 2.35800600 -0.17999700

H 1.42830800 3.98645300 -0.32520200

C 2.58345400 -0.01470300 -1.09518000

H 2.15604700 -0.58660700 -1.94281900

H 3.67363700 -0.18598600 -1.10617900

O 2.08685800 -0.51003900 0.12975200

H 0.97976900 -0.81537800 0.03624500

C 2.28669100 1.47209100 -1.27934200

H 2.68258100 1.78393800 -2.26288700

H 1.19305600 1.61964900 -1.33053800

H 2.95736100 4.44988800 0.45768200

H 2.88868500 4.22995100 -1.30741300

H 3.97084900 2.23330500 -0.16521500

H 2.51569200 1.99531300 0.79829700

C -3.74239200 1.30562400 0.95571100

C -2.86659900 2.54528700 0.74546600

H -3.47280800 0.78418600 1.88800700

H -4.79042700 1.62005300 1.08050000

H -2.76134600 3.07078800 1.70922500

H -3.37246100 3.24759900 0.06240100

H -0.78517600 3.03437600 0.38453800

H -1.51997100 2.10042600 -0.90821800

**DBU...HOH...CO_2_**

**Denoted by TS1-2**

0 1

C 2.00861400 -2.06224700 -0.61045800

C 3.08257000 0.61544200 -0.03642800

C 0.62063200 0.04977000 -0.07249700

C 0.84901900 -1.43069000 0.17056800

H 3.58407400 1.51345200 0.34977700

H 3.42905300 0.49622900 -1.08074400

H 0.99179600 -1.58741500 1.25328300

H -0.09969200 -1.91948900 -0.07881900

N 1.64732800 0.94197800 -0.02554800

N -0.62058300 0.39169400 -0.28991000

C 1.36658400 2.35623200 -0.30491900

C -0.03389600 2.73704900 0.14625600

H 1.49443100 2.55645700 -1.38509600

H 2.11971600 2.95721700 0.22458800

C -1.03117300 1.77444500 -0.47810200

H -0.09317700 2.67636400 1.24545700

H -0.25101800 3.77607300 -0.14183600

H -2.03090400 1.89012300 -0.03164300

H -1.13965900 1.98221000 -1.55864700

C -4.02825800 -0.53884200 0.31690200

O -4.90212000 -0.93348700 -0.38953800

O -3.66865900 0.10440400 1.25245900

O -2.46162300 -1.29443700 -0.35987500

H -1.60111100 -0.52456700 -0.34009000

C 3.52517500 -0.57937100 0.80501200

C 3.35168800 -1.93683700 0.11628600

H 2.98414500 -0.55033200 1.76432400

H 4.58756400 -0.43230300 1.05496800

H 3.44081600 -2.73276200 0.87441700

H 4.17281100 -2.09840600 -0.60171600

H 1.78152100 -3.12443800 -0.78937200

H 2.06697400 -1.59699500 -1.60865100

H -2.63105700 -1.53866600 -1.28208300

**DBUH...O_2_COBu**

**Denoted by Int1-1**

0 1

N -3.24791800 0.90312200 -0.32195000

N -1.06418900 0.51616400 0.37098700

C -3.12736900 2.34246000 -0.04260000

C -1.68311700 2.80470700 -0.18284600

H -3.51085000 2.54972400 0.97145700

H -3.77314800 2.87497800 -0.75394600

C -0.77269900 1.91546300 0.65074100

H -1.38346300 2.75207200 -1.24199200

H -1.59851400 3.85300700 0.13601500

H 0.28936200 2.06583400 0.41314100

H -0.91434900 2.11303300 1.72753400

C 4.17376800 -0.97964900 -0.85076200

H 3.94017200 -0.23843700 -1.63153400

H 4.69459500 -1.82937300 -1.32200600

O 2.96875200 -1.51667800 -0.31665200

C 1.92472600 -0.63544900 -0.07953900

O 0.92503500 -1.20475700 0.44349700

O 2.05919000 0.55811200 -0.38422400

C 5.05537100 -0.34835800 0.21961500

H 5.23896700 -1.09307500 1.01473800

H 4.49715400 0.48342800 0.68012100

C 6.38616800 0.16319200 -0.33249300

C 7.27407000 0.79986200 0.73568200

H 7.51953700 0.07661100 1.53122400

H 6.92789200 -0.67085400 -0.81454500

H 6.18870100 0.89773900 -1.13410500

H 8.22251300 1.16516800 0.31074100

H 6.76828400 1.65564400 1.21317100

C -2.21079200 0.08561100 -0.10708000

C -2.32824000 -1.39023700 -0.37723100

C -3.26267100 -2.11755500 0.60815000

H -2.67732800 -1.54153900 -1.41151800

H -1.31343100 -1.80241500 -0.31085500

C -4.56376500 0.39786000 -0.73932300

C -4.75192900 -1.94856400 0.30385400

H -3.03777200 -1.77591900 1.63305500

H -3.01226600 -3.18937800 0.57771800

C -5.25121200 -0.50204600 0.29208100

H -4.46984600 -0.12479600 -1.70560100

H -5.18143600 1.28487200 -0.92418900

H -4.96320600 -2.40062900 -0.68201400

H -5.33673100 -2.52569100 1.03845800

H -6.32964600 -0.50279100 0.06548600

H -5.14012300 -0.04361300 1.28971100

H -0.25800100 -0.18577900 0.45600400

**DBUH...O_2_COH**

**Denoted by Int1-2**

0 1

C 2.40373000 -2.01016700 -0.56391900

C 2.72441000 -0.91512200 0.46508100

C 0.60785800 0.27049200 0.60664300

C 0.03981200 -0.94116400 1.29645200

H 2.81813000 -2.95744700 -0.18123000

H 2.57255800 -1.29209300 1.48559800

H 2.92495500 -1.79736800 -1.51124900

H 3.77719300 -0.61125300 0.39950100

H 0.63634700 -1.11661000 2.20383300

H -0.97529300 -0.68170300 1.61704500

N 1.90860300 0.28951200 0.28392000

N -0.20153200 1.26939800 0.33574600

C 2.47246400 1.40733600 -0.48153500

C 1.70978600 2.69339300 -0.19048600

H 2.43993800 1.16707100 -1.55863200

H 3.52938900 1.50866300 -0.19861800

C 0.21624600 2.46572600 -0.38069300

H 1.90717200 3.00960100 0.84624800

H 2.06368600 3.49338900 -0.85559200

H -0.36861600 3.31616300 -0.00123200

H -0.03124200 2.35180600 -1.45054700

C 0.00972900 -2.21501100 0.40320500

C 0.90295500 -2.15854600 -0.84129600

H -1.02485400 -2.35869300 0.06432500

H 0.27840100 -3.08680500 1.02080800

H 0.73934700 -3.06828600 -1.44046000

H 0.56024000 -1.32293700 -1.47505700

C -3.27679600 0.04759000 -0.08542700

O -4.44939800 -0.31977900 -0.18317000

O -2.74764500 0.80676800 0.77610100

H -1.24703400 1.11854700 0.53017900

O -2.36952900 -0.41953900 -1.03890300

H -2.87840000 -0.99116300 -1.63296300

**DBU...CO_2_...HOBu**

**Denoted by Complex2-1**

0 1

N 1.73250300 -0.89176500 0.72968500

N -0.04292500 0.67659900 0.65150700

C 1.42160100 -1.28045600 2.10334500

C -0.06962500 -1.12952000 2.36349500

H 2.00110500 -0.66677700 2.82007000

H 1.73711800 -2.32497100 2.24649700

C -0.51259900 0.27570200 1.96749300

H -0.61630700 -1.87062100 1.75792600

H -0.29413700 -1.33396800 3.42158800

H -1.61319600 0.33986800 1.97166500

H -0.16055500 1.00885000 2.71851800

C -3.93980200 -0.44154600 -0.44249800

H -4.00507500 0.23570100 -1.31837700

H -4.80024700 -1.12953300 -0.51343800

O -4.09473100 0.28035400 0.76950600

C -1.29416800 2.82552800 -0.14253700

O -2.25570600 2.54062400 0.45666800

O -0.42575300 3.25891900 -0.78365200

C -2.63429200 -1.22606400 -0.49898600

H -2.60921600 -1.92281200 0.35801900

H -1.79915600 -0.52167600 -0.35254100

C -2.41578100 -1.99687400 -1.79979200

C -1.09535300 -2.76809700 -1.80527400

H -1.07092300 -3.51651900 -0.99561800

H -3.25510800 -2.69590000 -1.96929500

H -2.43383000 -1.29027900 -2.64956500

H -0.93430900 -3.29821900 -2.75741200

H -0.24043200 -2.09064500 -1.64636100

C 0.98847600 0.09433900 0.12885100

C 1.42725700 0.55118900 -1.24536300

C 2.70700300 1.40704200 -1.22384400

H 1.56864300 -0.31613700 -1.91168600

H 0.59874600 1.14114300 -1.65017300

C 2.99112100 -1.37517100 0.16665400

C 4.00529100 0.60297700 -1.12775100

H 2.63583100 2.12423400 -0.38768200

H 2.73594200 2.01183400 -2.14486700

C 4.10759800 -0.32462200 0.08536300

H 2.81847300 -1.81318000 -0.83155000

H 3.31674100 -2.20404400 0.80944600

H 4.10568900 -0.01018500 -2.04224000

H 4.86292200 1.29645000 -1.12875900

H 5.07464900 -0.85317900 0.04403500

H 4.10907700 0.26018900 1.02187700

H -3.47034000 1.02079300 0.74646800

**DBU...CO_2_...HOH**

**Denoted by Complex2-2**

0 1

N 1.99176600 0.69476600 0.16170200

N -0.34544500 0.91251300 0.52033800

C 2.10037400 2.00781900 -0.46952700

C 1.02146600 2.93912300 0.06400700

H 2.01727000 1.91764300 -1.57022300

H 3.10213000 2.41285900 -0.25745700

C -0.33443400 2.24854200 -0.05119000

H 1.23104100 3.16806300 1.12272700

H 1.03508300 3.89098300 -0.48910500

H -1.11782100 2.84952500 0.44103200

H -0.63324600 2.19191600 -1.11597800

O -5.67438800 -0.75777400 -0.34291400

C -2.82459600 -0.16668700 0.02739900

O -3.20094200 0.89157300 -0.28376800

O -2.52626000 -1.25462600 0.31168500

C 0.76102900 0.24611100 0.58436100

C 0.69086500 -1.16144800 1.13732900

C 0.82607000 -2.24972400 0.05645100

H 1.45579300 -1.31179600 1.91786100

H -0.29117900 -1.24291000 1.61582600

C 3.16095300 -0.17680500 0.08703700

C 2.26439400 -2.54225900 -0.37560700

H 0.21516400 -1.95654000 -0.81474300

H 0.37947500 -3.18040000 0.44314500

C 3.03975400 -1.33646900 -0.91163300

H 3.40844500 -0.57380400 1.08711800

H 4.00494200 0.46437200 -0.20162200

H 2.81526300 -2.95048500 0.49181800

H 2.25964700 -3.33980500 -1.13757000

H 4.05697700 -1.66427000 -1.18428600

H 2.57333300 -0.95564300 -1.83717300

H -5.51840100 -1.05216500 -1.25199800

H -5.54069000 0.19890100 -0.40543700

**DBU...CO_2_...HOBu**

**Denoted by TS2-1**

0 1

N 1.66784500 -0.82953800 0.80937900

N -0.04749200 0.77853800 0.59531000

C 1.33172100 -1.09876400 2.20680500

C -0.15578200 -0.88177100 2.43550800

H 1.92534600 -0.44820800 2.87670600

H 1.61225200 -2.13787700 2.43324900

C -0.54927800 0.50184800 1.93135100

H -0.72036000 -1.64961500 1.88259500

H -0.39747400 -0.99473300 3.50306600

H -1.64598600 0.59917400 1.90702200

H -0.18350200 1.27990400 2.62759300

C -3.94678500 -0.33100000 -0.45964400

H -3.98064300 0.26365500 -1.39543400

H -4.83684500 -0.98409800 -0.47124500

O -4.06776200 0.50586800 0.67862600

C -1.01640600 2.64067200 -0.25122800

O -2.06557600 2.53661300 0.27624500

O -0.15481900 3.09124500 -0.90664700

C -2.67845400 -1.17670900 -0.43956600

H -2.68215500 -1.78839100 0.48033200

H -1.81199000 -0.50051500 -0.35999400

C -2.49873400 -2.07707900 -1.66043300

C -1.20803300 -2.89442000 -1.59682800

H -1.20290700 -3.55887300 -0.71668800

H -3.36499700 -2.75663200 -1.75734100

H -2.49722600 -1.45575800 -2.57450100

H -1.07567800 -3.52205100 -2.49236600

H -0.32716600 -2.23684400 -1.51223600

C 0.96461700 0.11851000 0.11914400

C 1.41495600 0.42234000 -1.29179500

C 2.72597900 1.22744600 -1.35451700

H 1.52500900 -0.51749500 -1.85734600

H 0.61062400 0.99094700 -1.76620700

C 2.90233900 -1.42009600 0.29155200

C 3.99124100 0.38421600 -1.18477500

H 2.68694500 2.02431500 -0.59250300

H 2.77096400 1.73840200 -2.32981300

C 4.06022300 -0.42956400 0.10971500

H 2.70383600 -1.94430200 -0.65874500

H 3.19502100 -2.19541200 1.01157000

H 4.06357200 -0.31473800 -2.03814900

H 4.87598300 1.03928700 -1.24977300

H 5.00317200 -1.00110600 0.11699400

H 4.09144500 0.23921400 0.98750900

H -3.40006200 1.20519000 0.59535200

**DBU...CO_2_...HOH**

**Denoted by TS2-2**

0 1

N 1.83390700 0.81634400 0.22046000

N -0.52665100 0.70479900 0.22430500

C 1.85196800 2.17936900 -0.30981700

C 0.57950900 2.91390000 0.08209800

H 1.96333900 2.16118900 -1.41047900

H 2.73728500 2.69379500 0.09412200

C -0.62498200 2.05914400 -0.29306800

H 0.58101900 3.09307200 1.17040200

H 0.54027500 3.89461100 -0.41552300

H -1.55768000 2.50532200 0.08501800

H -0.73123900 2.02510200 -1.39471900

O -5.71475800 -0.79164700 -0.23853000

C -2.55108300 -0.32269400 0.07634000

O -3.20618100 0.65686500 0.05368900

O -2.26128600 -1.45886300 0.05535700

C 0.64146500 0.18324400 0.44623900

C 0.70235900 -1.23515000 0.96698600

C 1.12508000 -2.25779600 -0.10392700

H 1.38817100 -1.28913300 1.82827600

H -0.29899700 -1.47997900 1.33111400

C 3.11727200 0.11742300 0.29091800

C 2.63432900 -2.33006500 -0.34386800

H 0.59550500 -2.02338500 -1.04322700

H 0.76917400 -3.25304600 0.20797600

C 3.29258200 -1.00615800 -0.73957900

H 3.28481900 -0.27697100 1.30806100

H 3.88947400 0.88107100 0.13002300

H 3.11891300 -2.69549500 0.58010000

H 2.84386700 -3.08600200 -1.11903000

H 4.37313500 -1.17761300 -0.87727000

H 2.90389200 -0.65438700 -1.71121000

H -5.58578900 -0.98498500 -1.17796400

H -5.11913900 -0.03931400 -0.09707400

**DBUCO_2_...HOBu**

**Denoted by Int2-1**

0 1

N 1.83390700 0.81634400 0.22046000

N -0.52665100 0.70479900 0.22430500

C 1.85196800 2.17936900 -0.30981700

C 0.57950900 2.91390000 0.08209800

H 1.96333900 2.16118900 -1.41047900

H 2.73728500 2.69379500 0.09412200

C -0.62498200 2.05914400 -0.29306800

H 0.58101900 3.09307200 1.17040200

H 0.54027500 3.89461100 -0.41552300

H -1.55768000 2.50532200 0.08501800

H -0.73123900 2.02510200 -1.39471900

O -5.71475800 -0.79164700 -0.23853000

C -2.55108300 -0.32269400 0.07634000

O -3.20618100 0.65686500 0.05368900

O -2.26128600 -1.45886300 0.05535700

C 0.64146500 0.18324400 0.44623900

C 0.70235900 -1.23515000 0.96698600

C 1.12508000 -2.25779600 -0.10392700

H 1.38817100 -1.28913300 1.82827600

H -0.29899700 -1.47997900 1.33111400

C 3.11727200 0.11742300 0.29091800

C 2.63432900 -2.33006500 -0.34386800

H 0.59550500 -2.02338500 -1.04322700

H 0.76917400 -3.25304600 0.20797600

C 3.29258200 -1.00615800 -0.73957900

H 3.28481900 -0.27697100 1.30806100

H 3.88947400 0.88107100 0.13002300

H 3.11891300 -2.69549500 0.58010000

H 2.84386700 -3.08600200 -1.11903000

H 4.37313500 -1.17761300 -0.87727000

H 2.90389200 -0.65438700 -1.71121000

H -5.58578900 -0.98498500 -1.17796400

H -5.11913900 -0.03931400 -0.09707400

**DBUCO_2_...HOH**

**Denoted by Int2-2**

0 1

N 1.83390700 0.81634400 0.22046000

N -0.52665100 0.70479900 0.22430500

C 1.85196800 2.17936900 -0.30981700

C 0.57950900 2.91390000 0.08209800

H 1.96333900 2.16118900 -1.41047900

H 2.73728500 2.69379500 0.09412200

C -0.62498200 2.05914400 -0.29306800

H 0.58101900 3.09307200 1.17040200

H 0.54027500 3.89461100 -0.41552300

H -1.55768000 2.50532200 0.08501800

H -0.73123900 2.02510200 -1.39471900

O -5.71475800 -0.79164700 -0.23853000

C -2.55108300 -0.32269400 0.07634000

O -3.20618100 0.65686500 0.05368900

O -2.26128600 -1.45886300 0.05535700

C 0.64146500 0.18324400 0.44623900

C 0.70235900 -1.23515000 0.96698600

C 1.12508000 -2.25779600 -0.10392700

H 1.38817100 -1.28913300 1.82827600

H -0.29899700 -1.47997900 1.33111400

C 3.11727200 0.11742300 0.29091800

C 2.63432900 -2.33006500 -0.34386800

H 0.59550500 -2.02338500 -1.04322700

H 0.76917400 -3.25304600 0.20797600

C 3.29258200 -1.00615800 -0.73957900

H 3.28481900 -0.27697100 1.30806100

H 3.88947400 0.88107100 0.13002300

H 3.11891300 -2.69549500 0.58010000

H 2.84386700 -3.08600200 -1.11903000

H 4.37313500 -1.17761300 -0.87727000

H 2.90389200 -0.65438700 -1.71121000

H -5.58578900 -0.98498500 -1.17796400

H -5.11913900 -0.03931400 -0.09707400

**HOBu**

0 1

C -2.38926400 -0.15516000 -0.13833800

C -0.95679700 -0.31109700 0.36770800

H -2.43987800 -0.27778200 -1.23332800

C 1.45880200 0.51680300 0.21670300

H 2.09089600 1.32119300 -0.20878900

H 1.52281200 0.60542800 1.31455800

O 1.97972100 -0.76448000 -0.10083900

H 1.94076500 -0.86678900 -1.06240300

C 0.01283300 0.70773000 -0.23253000

H -0.30325500 1.73363900 0.02969600

H -0.02831300 0.65126400 -1.33738200

H -3.06215000 -0.90184000 0.31271500

H -2.79100100 0.84418700 0.09960200

H -0.93743500 -0.21926200 1.46905500

H -0.58365400 -1.32385800 0.14173200

**NC_7_H_13_, Quinine**

0 1

C -0.75761600 -0.23712100 -1.42575300

C 0.80016600 -0.22720100 -1.36388900

H -1.13202000 -1.19966300 -1.81031200

H -1.13155100 0.54843500 -2.10224100

H 1.21416100 -1.18417900 -1.71946500

H 1.21445500 0.56360700 -2.00939800

C -0.76198500 -1.11457900 0.91669000

H -1.13814900 -0.96569100 1.94181400

H -1.13737100 -2.09230300 0.57364700

C 0.79590400 -1.06935000 0.88027900

H 1.20825100 -0.89988000 1.88770500

H 1.20879900 -2.02463500 0.51887900

C 0.79785000 1.29532100 0.48740400

H 1.20922700 1.45926100 1.49613400

H 1.21318900 2.08198800 -0.16244200

C -0.75994500 1.35285800 0.50541400

H -1.13688300 1.54563300 1.52291400

H -1.13307100 2.16694700 -0.13691400

N 1.28456400 -0.00067000 0.00213200

C -1.29088400 0.00071300 -0.00205500

H -2.39192700 0.00133100 -0.00378600

**NC_7_H_13_^.+^_,_ Quinine^.+^**

1 2

C -0.75306300 0.27324700 -1.42438200

C 0.83751400 0.29861000 -1.36421500

H -1.06696500 -0.49812700 -2.14007800

H -1.10928600 1.24821900 -1.78254300

H 1.27350700 -0.47886100 -2.00243000

H 1.23192700 1.28226600 -1.64395200

C -0.73940800 -1.38715000 0.44733700

H -1.08677900 -1.62967300 1.46028600

H -1.05184500 -2.19164600 -0.23136200

C 0.85047000 -1.31217300 0.45492900

H 1.25337200 -1.46766100 1.46240600

H 1.28748000 -2.03660900 -0.24207200

C 0.79131200 1.06839800 0.94000200

H 1.19670400 0.83694800 1.93164300

H 1.18538100 2.02757200 0.58506700

C -0.80033000 1.06344000 0.94871000

H -1.14766300 0.87092600 1.97223200

H -1.15855000 2.05398400 0.63862500

N 1.16066000 0.02568700 0.01461400

C -1.27852000 -0.02840600 -0.01579400

H -2.37975200 -0.05294000 -0.02964100

**HNC_7_H_13_^.+^, Quinine-H^+^**

1 1

C -0.79750900 1.43925800 0.10231900

C 0.74414700 1.43585300 0.03765400

H -1.20256000 2.04769500 -0.71790800

H -1.13567000 1.89069400 1.04569700

H 1.13594300 1.91677800 -0.86731200

H 1.21231200 1.90646600 0.91070900

C -0.78249300 -0.63440900 -1.30348200

H -1.18249900 -1.65057500 -1.42366500

H -1.11445200 -0.04434700 -2.16947300

C 0.75902000 -0.68259900 -1.25362900

H 1.15535800 -1.70442100 -1.21188200

H 1.23197900 -0.16158400 -2.09458300

C 0.75079700 -0.74446100 1.22329700

H 1.22605700 -1.73220500 1.18916100

H 1.13987400 -0.20215300 2.09349800

C -0.79038700 -0.81509100 1.19393900

H -1.11916400 -1.86068600 1.11184600

H -1.19944800 -0.41308900 2.13110200

N 1.22572600 0.00669200 0.00400000

H 2.25010400 0.01383400 0.00735300

C -1.31157600 -0.00563100 -0.00417900

H -2.40991000 -0.01077300 -0.00805800

**Ir[dF(CF_3_)ppy]_2_(dtbbpy)]^+^**

**Denoted by Ir^III^**

1 1

N 0.91414900 0.27494600 1.30310100

C 0.84945900 0.57662200 2.60936500

C 1.98369700 0.71792800 3.39616800

C 3.25891000 0.54063500 2.83262100

C 3.30099900 0.21671200 1.47211900

C 2.12460200 0.08829400 0.72704500

N 0.89430500 -0.35401800 -1.29399500

C 0.81042600 -0.65846700 -2.59848300

C 1.93325300 -0.87975900 -3.38325900

C 3.21707200 -0.78358100 -2.81959500

C 3.27971700 -0.45791500 -1.46031900

C 2.11398600 -0.24822500 -0.71695000

H -0.15023000 0.71353300 3.02409000

H 1.85794500 0.96928700 4.44908100

H 4.25592500 0.06112200 0.98008900

H -0.19591000 -0.73023200 -3.01345700

H 1.79189600 -1.12878800 -4.43477200

H 4.24250300 -0.36523800 -0.96779800

C -2.86544500 1.52731400 1.70845000

C -2.59334300 -0.86206300 2.11824000

C -3.80989300 1.56629600 2.73017200

H -2.63318300 2.45160400 1.17889500

C -3.55557000 -0.76053100 3.13932100

C -1.89338800 -2.08224400 1.72702500

C -4.17521100 0.43766300 3.46342200

C -2.06734400 -3.36776700 2.27210300

N -0.98647000 -1.90115400 0.71798100

H -4.91638200 0.48755600 4.26029200

C -1.32449000 -4.43459100 1.78868600

H -2.78707500 -3.51681200 3.07101000

C -0.26693600 -2.93113100 0.24861300

C -0.40392900 -4.21535400 0.75528800

H -1.46141900 -5.43160500 2.21127700

H 0.43077600 -2.71499100 -0.55778100

N -0.82411100 1.96651200 -0.71887500

C -0.02785800 2.93592300 -0.24441900

C -1.70179400 2.21760000 -1.73870300

C -0.05144600 4.22446200 -0.75793500

H 0.63979000 2.66696200 0.57144300

C -1.76170100 3.51032600 -2.29120100

C -2.49769400 1.05849400 -2.13205100

C -0.93790600 4.51400600 -1.80376300

H -2.45711600 3.71480100 -3.09932300

C -3.45674200 1.03388000 -3.16083500

H -0.98616600 5.51665400 -2.23239900

C -4.17396700 -0.10871000 -3.48441400

C -2.97595900 -1.29664200 -1.71341100

C -3.91250100 -1.26023800 -2.74248400

H -4.91030900 -0.09944200 -4.28724400

H -2.82826700 -2.23465300 -1.17764600

Ir -0.80977400 0.02896300 0.00095000

F -3.71165900 2.14075700 -3.88019300

F -4.59709100 -2.37279900 -3.03938500

F -3.91016500 -1.84501200 3.85052500

F -4.39894100 2.73196400 3.02821400

C 0.85914600 5.28840100 -0.20783200

C 0.42165300 -5.34910900 0.21049500

F 0.16100600 6.34478300 0.24595600

F 1.61170100 4.83414600 0.80690800

F 1.69497800 5.75374800 -1.15392600

F 1.21077900 -5.88088600 1.16162200

F -0.35672100 -6.34533500 -0.24882400

F 1.21536300 -4.95681100 -0.79874300

C 4.46278400 -1.03468100 -3.67109000

C 4.47273000 -0.03347900 -4.84650400

C 5.75777100 -0.86257000 -2.86338800

C 4.40240100 -2.47941700 -4.21379600

H 3.58583800 -0.14280400 -5.48777700

H 4.50402900 1.00394000 -4.47910200

H 5.36222500 -0.20326800 -5.47252600

H 5.81549600 -1.57338000 -2.02499900

H 6.62333100 -1.04896000 -3.51592300

H 5.85758900 0.15751900 -2.46208700

H 5.29097700 -2.68309100 -4.83099100

H 4.38206000 -3.20864200 -3.38911000

H 3.51299800 -2.64579700 -4.83932200

C 4.51772300 0.70521500 3.68601100

C 4.46332200 -0.30480300 4.85270100

C 5.79958300 0.45851400 2.87673800

C 4.54909500 2.14592500 4.24166000

H 3.58455700 -0.14473300 5.49454900

H 4.42901200 -1.33894900 4.47642100

H 5.36125100 -0.19710200 5.48045000

H 5.90117000 1.16988800 2.04293200

H 6.67494400 0.58667900 3.53021500

H 5.83630000 -0.56315300 2.46887500

H 5.44958300 2.28757800 4.85894600

H 4.57365900 2.88247600 3.42363700

H 3.67293500 2.36237200 4.87051400

C -2.25288500 -0.14046100 -1.39589800

C -2.24174500 0.31495600 1.39000900

**Triplet State of Ir[dF(CF_3_)ppy]_2_(dtbbpy)]^+^**

**Denoted by T-Ir^III^**

1 3

N 0.88652900 0.31446800 1.32090900

C 0.80002700 0.49488000 2.64776500

C 1.91895000 0.69007700 3.44428300

C 3.20025400 0.70445200 2.86733600

C 3.26515900 0.51212300 1.48285100

C 2.10335300 0.31685500 0.72999400

N 0.91572800 -0.10361100 -1.32148400

C 0.85414100 -0.29354500 -2.64870100

C 1.98536200 -0.28956800 -3.45177300

C 3.25259600 -0.07877100 -2.88216100

C 3.29044600 0.12362400 -1.49816200

C 2.11744600 0.10870400 -0.73767300

H -0.20511800 0.48938900 3.07176200

H 1.77663200 0.83130400 4.51533500

H 4.22678900 0.51095700 0.97933800

H -0.13829900 -0.46064500 -3.06923700

H 1.86319200 -0.45472300 -4.52189200

H 4.23943300 0.29685200 -1.00056800

C -2.97995400 1.03399800 1.84440400

C -2.37079800 -1.36840900 2.08648100

C -3.89227300 0.87819600 2.87760100

H -2.88606900 2.01137200 1.37082600

C -3.34163600 -1.44733000 3.13783000

C -1.56137400 -2.41970000 1.61204600

C -4.08317700 -0.36555000 3.53511200

C -1.53214500 -3.78086300 2.05564300

N -0.67463700 -2.03165700 0.56426900

H -4.80909500 -0.45996300 4.34339200

C -0.66228400 -4.68516100 1.50692400

H -2.21818600 -4.08562800 2.84158500

C 0.17053800 -2.91858500 0.04650000

C 0.22837000 -4.24369000 0.47051800

H -0.64520600 -5.72072800 1.84748700

H 0.82568100 -2.56632100 -0.74928500

N -1.13320900 1.89447200 -0.59632800

C -0.46932900 2.94034500 -0.08495000

C -2.06193600 2.06618200 -1.58512600

C -0.69475500 4.23708700 -0.52528400

H 0.25469700 2.72635500 0.69917600

C -2.32731800 3.36222900 -2.06169500

C -2.67523100 0.82135500 -2.04417400

C -1.64496400 4.44813200 -1.53248600

H -3.06550600 3.50583400 -2.84459300

C -3.63806300 0.70902100 -3.06233500

H -1.84898000 5.45409700 -1.90326700

C -4.16078600 -0.51370400 -3.46124000

C -2.75133300 -1.61027600 -1.79230100

C -3.69915500 -1.65758000 -2.81045100

H -4.90340200 -0.56995900 -4.25647800

H -2.43413500 -2.54151100 -1.32316100

Ir -0.82443800 -0.07140200 0.00588600

F -4.08781400 1.80348500 -3.69676100

F -4.19438100 -2.84255800 -3.18539100

F -3.53657000 -2.61946500 3.77023900

F -4.63273000 1.91603900 3.28759600

C 0.05295300 5.39509600 0.07880400

C 1.19624800 -5.19950100 -0.14286800

F -0.77320600 6.21190500 0.75674800

F 1.00658800 4.98822100 0.93127000

F 0.64536200 6.14061500 -0.87026000

F 2.04267200 -5.71396500 0.77662600

F 0.56963500 -6.25729300 -0.70364900

F 1.94931000 -4.62970300 -1.10078800

C 4.50851400 -0.08071200 -3.75465900

C 4.36796200 1.01785300 -4.83040100

C 5.78001600 0.18718400 -2.93619700

C 4.63304900 -1.46301500 -4.43188400

H 3.49613300 0.84891300 -5.47946700

H 4.26342200 2.01102200 -4.36695600

H 5.26447500 1.02918600 -5.46917500

H 5.94320300 -0.58379000 -2.16779500

H 6.65330200 0.17888300 -3.60476700

H 5.74902600 1.17019600 -2.44195500

H 5.53321500 -1.48705900 -5.06523600

H 4.71957000 -2.26271600 -3.68014200

H 3.76601500 -1.68611000 -5.07103500

C 4.44155900 0.92100200 3.73394000

C 4.51205700 -0.20723100 4.78582300

C 5.73373900 0.90689700 2.90425100

C 4.31827300 2.28803000 4.44131400

H 3.62739300 -0.21511500 5.43952700

H 4.58893500 -1.19261700 4.30086700

H 5.39934300 -0.06694500 5.42224400

H 5.74319200 1.70629600 2.14770100

H 6.59594000 1.06750400 3.56794300

H 5.88250100 -0.05696200 2.39398800

H 5.20478000 2.46211400 5.07039500

H 4.25112900 3.10598700 3.70746800

H 3.43151700 2.33631000 5.09041900

C -2.23196300 -0.37227700 -1.39777300

C -2.20661900 -0.05536000 1.43123100

**Ir[dF(CF_3_)ppy]_2_(dtbbpy)]**

**Denoted by Ir^II^**

0 2

N -0.81114900 0.54884000 -1.25577100

C -0.72077000 0.98602500 -2.52419500

C -1.81119000 1.38154200 -3.27475800

C -3.11746400 1.33154600 -2.69144400

C -3.20962600 0.87061700 -1.39389900

C -2.05833300 0.46559200 -0.65387700

N -0.88189300 -0.33317900 1.28020000

C -0.86253000 -0.77864000 2.54888700

C -2.00037800 -0.96410700 3.31016300

C -3.28031100 -0.67617200 2.73711100

C -3.29705300 -0.20371200 1.44061900

C -2.09731800 -0.01669700 0.69043100

H 0.28857800 1.02089600 -2.94092200

H -1.65369300 1.72747300 -4.29572700

H -4.17768400 0.81368300 -0.90306600

H 0.12632200 -1.00251700 2.95600700

H -1.90045600 -1.33329000 4.33025800

H -4.24231400 0.02959600 0.95757800

C 3.09630000 1.18811100 -1.65024500

C 2.40944600 -1.07833500 -2.23296300

C 4.02141500 1.14528500 -2.68754900

H 3.03136600 2.09375200 -1.04536400

C 3.36303300 -1.06299800 -3.26640400

C 1.51832800 -2.19079700 -1.91482700

C 4.17781400 0.03224800 -3.51481500

C 1.45470600 -3.43809100 -2.56493900

N 0.67784600 -1.94195000 -0.86358500

H 4.90744600 0.01694100 -4.32363500

C 0.54623300 -4.39712800 -2.14293100

H 2.12006300 -3.64140100 -3.39843700

C -0.20363200 -2.86579400 -0.45456500

C -0.30350900 -4.10744500 -1.06636400

H 0.49637600 -5.36327500 -2.64832200

H -0.83853800 -2.58832600 0.38461300

N 1.12020400 1.77280100 0.85415600

C 0.45733300 2.87196300 0.46619100

C 2.02265400 1.82399000 1.88213900

C 0.65585700 4.10274800 1.07604800

H -0.24526400 2.74626000 -0.35550400

C 2.25915500 3.05208500 2.52867100

C 2.64908200 0.53915200 2.18125200

C 1.57856700 4.19170300 2.12753200

H 2.97664700 3.09878000 3.34215100

C 3.60139700 0.30732000 3.18972900

H 1.76303300 5.14383700 2.62825900

C 4.15414200 -0.94399200 3.42163300

C 2.79144400 -1.82280300 1.59048800

C 3.72894700 -1.99166900 2.60347600

H 4.88929700 -1.09514000 4.21123700

H 2.50797200 -2.68935500 0.99127000

Ir 0.82511900 -0.07593600 -0.00437900

F 4.01507100 1.31455700 3.98274500

F 4.25490300 -3.20884100 2.81278600

F 3.51799300 -2.13426000 -4.06824300

F 4.80307400 2.21319000 -2.91241500

C -0.11000400 5.31524900 0.62697500

C -1.29896700 -5.12337500 -0.58129900

F 0.71731900 6.31553100 0.26961100

F -0.90397100 5.05343400 -0.42405100

F -0.89248700 5.79654700 1.61185800

F -2.11491000 -5.52548700 -1.57382400

F -0.68843500 -6.22881300 -0.11328700

F -2.07220100 -4.64381400 0.40681600

C -4.54987100 -0.90934700 3.56015300

C -4.49014700 -0.04670600 4.83887600

C -5.82315600 -0.54281800 2.78378200

C -4.62537200 -2.40175600 3.94933800

H -3.61332700 -0.29148400 5.45646900

H -4.43905700 1.02402600 4.58557700

H -5.39047300 -0.21145900 5.45230400

H -5.93283400 -1.14919000 1.87163200

H -6.70734300 -0.72407100 3.41382500

H -5.83139200 0.51890900 2.49308200

H -5.53124000 -2.59548100 4.54596800

H -4.66293700 -3.03774900 3.05075300

H -3.75556100 -2.71164000 4.54740500

C -4.33044900 1.78746000 -3.50664400

C -4.43618700 0.92329300 -4.78152100

C -5.64304700 1.65842800 -2.71998500

C -4.13976800 3.26756700 -3.90289200

H -3.53431000 1.00444400 -5.40619800

H -4.57603600 -0.13825700 -4.52309900

H -5.29700000 1.24460400 -5.38961000

H -5.63480200 2.27704800 -1.80949400

H -6.48486200 1.99431200 -3.34459000

H -5.84014900 0.61631400 -2.42513000

H -4.99946400 3.61764700 -4.49656700

H -4.05876300 3.90381800 -3.00739800

H -3.23167700 3.41464600 -4.50620300

C 2.23155000 -0.55710200 1.36356000

C 2.27095000 0.08068600 -1.40675500

**[HOCHPr...H...NC_7_H_13_]^.+^**

**Denoted by TS3**

1 2

C -1.80875200 1.25892000 0.42541500

O -1.52049500 2.49807200 -0.11812200

C -2.93536500 0.48564900 -0.23341200

H -2.70733900 0.36103500 -1.30795400

H -3.85014800 1.10366000 -0.17583400

C -3.18117400 -0.88057900 0.40865500

C -4.32932200 -1.64389600 -0.24837500

H -4.12751600 -1.82256600 -1.31729000

H -2.25847700 -1.48344900 0.35277200

H -3.39103700 -0.74271300 1.48352800

H -4.48516300 -2.62183600 0.23259000

H -5.27367200 -1.07966300 -0.17786900

H -1.66487400 2.47383600 -1.07767500

C 0.71465500 -1.29559200 -0.38395100

C 2.21052600 -1.67507200 -0.66229100

H 0.25832000 -1.95275200 0.36633900

H 0.10820200 -1.32151300 -1.29760800

H 2.45489700 -2.60512500 -0.13051000

H 2.35465000 -1.85273800 -1.73729800

C 1.33391400 0.17640700 1.46072900

H 1.24243700 1.21533900 1.80112400

H 0.78471200 -0.47331700 2.15343300

C 2.82985000 -0.26034200 1.30613300

H 3.48495700 0.53439900 1.68929900

H 3.01589300 -1.16694700 1.89901300

C 2.77209100 0.74530100 -0.98131800

H 3.35974100 1.59810300 -0.61358800

H 3.00426400 0.60887700 -2.04668400

C 1.24249900 1.04228500 -0.81931400

H 1.04715700 2.05150900 -0.43493200

H 0.70239500 0.91578200 -1.76608900

N 0.71203500 0.07224900 0.13823800

H -1.93825700 1.38157000 1.51382000

H -0.84435700 0.59702800 0.35105200

C 3.10736100 -0.52329600 -0.18171200

H 4.16522800 -0.78754700 -0.32567700

**[HOCHPr]^.^**

**Denoted by Int3**

0 2

C -1.33814800 0.45809100 -0.01945000

O -2.54210700 -0.19186800 -0.01587500

C -0.07381900 -0.32680900 0.09255100

H -0.08301500 -1.14850700 -0.65180200

H -0.01533800 -0.83442000 1.08256600

C 1.18381300 0.52479100 -0.10584800

C 2.47775400 -0.27693300 0.01745300

H 2.51998900 -1.08328700 -0.73369300

H 1.13483700 1.01096700 -1.09557600

H 1.18274800 1.34356800 0.63557500

H 3.36281900 0.36229900 -0.12763000

H 2.56268400 -0.74667100 1.01153000

H -2.41468300 -1.09940300 -0.33096000

H -1.41079000 1.45556600 0.42876000

**[DBUCO_2_...HOCHPr...H...NC_7_H_13_]^.+^**

**Denoted by TS4**

1 2

N -3.83865900 0.37071500 -0.27935500

N -1.75316000 1.17683100 -0.94503700

C -4.08745000 1.56414500 0.54170400

C -3.46128900 2.76746900 -0.13256400

H -3.67259500 1.40588700 1.55160200

H -5.17373000 1.67930400 0.63915400

C -1.98619600 2.50505500 -0.34040900

H -3.96514700 2.95025900 -1.09504100

H -3.59101600 3.66435200 0.48862300

H -1.53158000 3.24358900 -1.01031100

H -1.43936600 2.56243300 0.61219000

C 1.29858100 1.25123500 2.06421100

O 1.17414700 2.44218900 1.36306500

C -0.31829800 0.90911400 -1.29528100

O 0.40554500 1.91828400 -1.15390400

O -0.00322800 -0.24273400 -1.59556400

C 0.08476200 0.32654200 2.04729900

H -0.12293300 0.00163100 1.01575100

H -0.79595300 0.90225200 2.38439700

C 0.26440400 -0.91039400 2.92789600

C -0.94475900 -1.84309300 2.88625900

H -1.12878200 -2.20687400 1.86274000

H 1.16583200 -1.46139000 2.60574500

H 0.45804100 -0.59473600 3.96794500

H -0.79992200 -2.72207300 3.53316600

H -1.85827700 -1.32461500 3.22142600

C -2.72084600 0.23967100 -0.98975100

C -2.59312000 -0.97510600 -1.87161500

C -2.29433200 -2.27655800 -1.10328900

H -3.54630200 -1.07335900 -2.41511900

H -1.80825400 -0.78773400 -2.60267800

C -4.84172500 -0.71019400 -0.19723400

C -3.53097000 -2.91331200 -0.47041300

H -1.52276900 -2.07027900 -0.34672800

H -1.84312200 -2.98794600 -1.81156300

C -4.31982300 -1.99115500 0.46181800

H -5.24733800 -0.91768800 -1.19995200

H -5.66680400 -0.30112700 0.39561200

H -4.20566400 -3.25421800 -1.27585000

H -3.23218100 -3.81742000 0.08448300

H -5.19372200 -2.53979100 0.84743700

H -3.71378600 -1.71086200 1.33948300

H 0.86517600 2.26827800 0.43805100

C 4.58022300 -0.36271900 1.15626800

C 5.61869000 -0.88404700 0.09400400

H 4.93727600 0.53962400 1.66731200

H 4.33995400 -1.13025700 1.90222800

H 6.53211900 -0.27581700 0.15001000

H 5.88784200 -1.92445400 0.32377300

C 3.52254600 1.10334700 -0.47951600

H 2.54662100 1.33351800 -0.92673800

H 3.86443600 1.96647800 0.10527600

C 4.57930400 0.67417500 -1.56515500

H 4.13421500 0.78094300 -2.56422600

H 5.45537300 1.33533800 -1.50920600

C 3.72427200 -1.66487300 -1.34312700

H 3.22332800 -1.58013300 -2.31791800

H 3.97974100 -2.72126700 -1.18036700

C 2.73965700 -1.18496100 -0.21109400

H 1.77243000 -0.88207500 -0.63650600

H 2.59276400 -1.95968900 0.55146200

N 3.37125100 -0.03325700 0.41526600

H 1.59608400 1.49462800 3.09856900

H 2.17199200 0.64963100 1.64509600

C 4.98116700 -0.78399700 -1.30006200

H 5.69948700 -1.11832500 -2.06392800

**[DBUCO_2_...HOCHPr]^.^**

**Denoted by Int4**

0 2

N -2.79471500 0.10949200 -0.00976900

N -0.81578200 1.33019500 -0.20122600

C -3.48081000 1.14191300 0.78181700

C -2.80963000 2.48787900 0.61224200

H -3.48511000 0.82931700 1.83940400

H -4.52651700 1.17502300 0.44372700

C -1.31246700 2.31675000 0.77327000

H -3.03097500 2.90330700 -0.38370800

H -3.19537700 3.19282000 1.36180900

H -0.77347900 3.25231300 0.59913300

H -1.05779300 1.97979500 1.79122100

C 4.32413900 0.76687900 0.05790100

O 3.89376200 2.05363400 0.05176900

C 0.61780500 1.55837900 -0.64888600

O 1.26782700 2.16018100 0.22833100

O 0.94815700 1.16474900 -1.76356500

C 3.38363400 -0.35173200 0.38060600

H 2.56835100 -0.38223600 -0.36762200

H 2.87155900 -0.16798800 1.35259500

C 4.07965900 -1.71405700 0.43008300

C 3.12677700 -2.87069800 0.72393400

H 2.35155900 -2.95467600 -0.05461000

H 4.59341300 -1.88913700 -0.53197600

H 4.87560100 -1.68542500 1.19597300

H 3.65951900 -3.83377400 0.77166200

H 2.60903200 -2.72566900 1.68702900

C -1.56296700 0.26157600 -0.50617400

C -1.00575300 -0.85332400 -1.34962700

C -0.56352300 -2.06353500 -0.50080700

H -1.77174200 -1.16111600 -2.07648100

H -0.16273400 -0.44791400 -1.90998000

C -3.52239400 -1.16989100 -0.12129100

C -1.70913800 -2.99907200 -0.11425900

H -0.03365700 -1.70384100 0.39713800

H 0.17791900 -2.62854000 -1.08596800

C -2.86158000 -2.32666200 0.63465800

H -3.67128000 -1.42398000 -1.18219000

H -4.51834000 -0.97997200 0.29473600

H -2.11209300 -3.45724600 -1.03521000

H -1.31451800 -3.82809100 0.49523200

H -3.64112700 -3.07874100 0.83558000

H -2.52483700 -1.95474100 1.61753400

H 2.89611400 2.09114000 0.06257400

H 5.38950500 0.66660200 0.30627400

**[DBUH...O_2_COCHPr...H...NC_7_H_13_]^.+^**

**Denoted by TS5**

1 2

N -3.22654900 0.32473600 0.72331900

N -1.87565500 -1.54399100 0.98020900

C -3.21417200 0.58299000 2.17310000

C -1.93547300 0.05953300 2.81099500

H -4.10397800 0.11667400 2.62885400

H -3.29875300 1.66832700 2.31606300

C -1.71291500 -1.39384400 2.42156100

H -1.08073300 0.66775100 2.47642200

H -2.00259700 0.15794500 3.90307400

H -0.69405800 -1.72341800 2.66291900

H -2.42636200 -2.05477200 2.94136400

C 2.91968100 -1.30706900 -1.14275400

O 1.78044100 -2.07005700 -1.25705200

C 0.92375800 -2.25122000 -0.12312200

O -0.02746000 -3.01212600 -0.37892800

O 1.19006900 -1.60455300 0.89409500

C 3.99436200 -1.75531700 -0.16967500

H 4.27807900 -2.78515500 -0.45998900

H 3.57426900 -1.81533900 0.84419400

C 5.22612800 -0.84986600 -0.19377400

C 6.33358100 -1.33907000 0.73740100

H 6.67476600 -2.34784000 0.45245200

H 5.61054800 -0.77621000 -1.22612800

H 4.93554700 0.17517100 0.09530800

H 7.20621400 -0.66832300 0.70977500

H 5.97989700 -1.39037800 1.78017100

C -2.55911300 -0.71262300 0.21606300

C -2.58295300 -0.99641200 -1.26083900

C -3.95610900 -1.48498400 -1.76185700

H -2.28508600 -0.08885400 -1.81004300

H -1.81596200 -1.75732300 -1.45271100

C -4.08784700 1.17858100 -0.11207700

C -4.98192200 -0.36922200 -1.96440300

H -4.34395200 -2.24457200 -1.06209300

H -3.79459300 -2.00073900 -2.72082700

C -5.29576000 0.45667900 -0.71516200

H -3.48071200 1.64840300 -0.90300400

H -4.42881600 1.98937000 0.54214600

H -4.61151500 0.31154400 -2.75173600

H -5.91634800 -0.80709700 -2.35075700

H -6.04131800 1.22431700 -0.97714900

H -5.75552500 -0.17432000 0.06448000

H -1.27865100 -2.25456500 0.50089000

C 2.11886900 3.56627300 -0.93895100

C 2.68209000 2.15979100 -1.31040100

H 1.80640800 4.09065900 -1.85294200

H 2.90169000 4.17038000 -0.45813300

H 2.50131600 1.90594000 -2.36276000

H 3.75847700 2.08016700 -1.11243900

C -0.09771700 2.44903000 -0.64757100

H -0.99591900 2.35170200 -0.02149500

H -0.41241600 2.85328100 -1.62033600

C 0.55968800 1.04829400 -0.83227000

H 0.12828800 0.29838700 -0.16194300

H 0.48814100 0.68458600 -1.86536200

C 2.17106900 1.40911900 0.96136100

H 1.77670800 0.53516200 1.49089800

H 3.24914800 1.48387900 1.15105100

C 1.42507900 2.73206200 1.31591500

H 0.57654900 2.52004900 1.98308500

H 2.10655500 3.40846500 1.85080100

N 1.98489400 1.16428400 -0.47970100

H 3.30513500 -1.13239900 -2.16074500

H 2.57558800 -0.19305500 -0.79892100

C 0.92724300 3.38296300 0.01508600

H 0.46533200 4.35674500 0.23209200

**[DBUH...O_2_COCHPr]^.^**

**Denoted by Int5**

0 2

C 3.61562900 -0.19173000 0.63766800

C 2.43716500 0.58186400 1.25262700

C 0.55954100 -0.13144600 -0.16314100

C 0.70145000 -1.48099200 0.50709000

H 4.20450900 -0.62728200 1.46258200

H 2.02020700 0.01794600 2.09892500

H 4.28543100 0.50399300 0.10586700

H 2.78068800 1.54064400 1.66586600

H 0.58546900 -1.35477400 1.59361000

H -0.13899900 -2.09353500 0.16325800

N 1.36941400 0.87040300 0.29927100

N -0.29334300 -0.01308100 -1.13497500

C 1.38594900 2.16538300 -0.37616300

C 0.02546600 2.44803500 -0.99789200

H 2.17438600 2.18340500 -1.15227800

H 1.64554300 2.93622900 0.36530800

C -0.39690800 1.25313500 -1.84786400

H -0.71368300 2.61090700 -0.19714400

H 0.06976900 3.36683000 -1.60167100

H -1.43580900 1.37404700 -2.19647600

H 0.22577100 1.20422600 -2.76108200

C -2.70846200 1.52126400 1.75961600

C -3.15469500 0.65474900 0.58418100

H -1.63963300 1.36037100 1.97998000

C -3.17849100 -1.74168100 -0.33608700

H -3.82017900 -2.62847900 -0.26997300

O -2.22146300 -1.84538000 -1.29182700

H -1.51115500 -1.11622600 -1.23101700

C -2.99615900 -0.85397700 0.85516900

H -3.72282700 -1.15460400 1.62971800

H -1.99361400 -1.01263600 1.30691000

H -2.84864200 2.59408700 1.55112400

H -3.27447000 1.28100700 2.67561100

H -4.20803200 0.86446900 0.32907100

H -2.56551200 0.91854200 -0.30599700

C 2.04456600 -2.19812600 0.18404100

C 3.16660600 -1.28880900 -0.33392800

H 1.85263800 -2.96533600 -0.58222200

H 2.38792100 -2.73782500 1.08187600

H 4.03627500 -1.90968000 -0.60323800

H 2.83320000 -0.82056500 -1.27596400

**[BuOCO_2_...HOBu]^-^**

**Denoted by Int6**

-1 1

C -0.52078000 2.16806300 1.03615400

C -1.57363800 1.81736200 -0.01404900

H -0.09185900 1.25182400 1.47232100

C -3.71345500 0.49633200 -0.51483800

H -4.48484900 -0.12822300 -0.01125100

H -4.25664000 1.35504000 -0.95502000

O -3.09987100 -0.19503500 -1.57606200

H -2.35983800 -0.75384800 -1.22244500

C -2.73856900 1.00324200 0.55108800

H -3.30589200 1.60686500 1.28372000

H -2.34079100 0.13699800 1.10600300

H 0.30842700 2.75068700 0.60233700

H -0.95267100 2.76344200 1.85879100

H -1.96055500 2.73879200 -0.48770000

H -1.10890500 1.22608700 -0.81787600

C -0.59268400 -1.60928700 0.41779000

O -0.92577400 -1.52218000 -0.79120500

O 0.70715000 -1.18699400 0.73262900

O -1.24443400 -2.00363600 1.39188900

C 1.50079100 -0.65574500 -0.31569600

C 2.84415600 -0.23694000 0.25804300

H 0.99428400 0.20844400 -0.77988300

H 1.63555400 -1.40676800 -1.11477300

C 3.78273000 0.34687100 -0.79853800

H 2.67574200 0.50585300 1.05804700

H 3.31776600 -1.11088100 0.73995200

C 5.13481400 0.77680800 -0.23171800

H 3.93733100 -0.39816800 -1.59974300

H 3.29248200 1.21153900 -1.28132000

H 5.78891300 1.19352300 -1.01407000

H 5.01212800 1.54704600 0.54799200

H 5.66261500 -0.07572500 0.22749600

**[BuOCO_2_...HOCHPr...H...NC_7_H_13_]^.^**

**Denoted by TS6**

0 2

C 1.42447300 -3.02719100 2.12642700

C 0.15293000 -2.37183400 1.59253600

H 2.03343100 -3.40452800 1.28999400

C -1.90869600 -2.69078000 0.06446000

H -2.76811700 -3.38107900 0.00777100

H -2.27445300 -1.81374800 0.66368200

O -1.60712000 -2.30175700 -1.24712000

H -0.69070200 -1.88303400 -1.31043800

C -0.74835400 -3.34604700 0.82735000

H -1.16367100 -4.08305400 1.53692800

H -0.14661100 -3.91145200 0.09586900

H 2.03880500 -2.31119000 2.69591500

H 1.19374500 -3.87563200 2.79302500

H -0.42402400 -1.92663100 2.42334800

H 0.42963600 -1.54007800 0.93111900

C 1.74496900 -1.63473400 -1.23331600

O 0.64107200 -1.04023300 -1.42780200

O 2.78350500 -0.84727000 -0.75664900

O 2.01948000 -2.82404000 -1.40014700

C -1.08943900 2.33201800 -0.05344400

C -2.52067200 2.73804600 -0.42547300

C -1.12564300 0.82944300 0.43599600

H -0.41568400 2.40703800 -0.91819100

H -0.69294700 2.97087900 0.74801200

H -0.58762700 0.17402200 -0.26237700

H -0.70266900 0.73045500 1.44268300

C -3.04758300 1.75826100 -1.48443900

H -4.04556200 2.04912300 -1.84053300

H -2.37086800 1.72135700 -2.34940800

C -3.14757300 0.32978500 -0.81681800

H -4.19301100 0.03092500 -0.67218500

H -2.61519600 -0.44748500 -1.38880600

H -2.52900100 3.76288700 -0.82803300

C -3.30472200 1.20706800 1.43784100

H -4.29510400 0.74824900 1.54610900

H -2.78742500 1.20691200 2.40455700

C -3.40655100 2.66685400 0.82854000

H -4.45440800 2.88443300 0.57932200

H -3.07571700 3.39033300 1.58621900

N -2.52338400 0.45650500 0.47891600

C 2.51143300 0.51552300 -0.46023900

C 3.80993200 1.20165800 -0.07389900

H 1.78539000 0.58497300 0.36966000

H 2.04914000 1.01273500 -1.32990200

C 3.60768200 2.67522600 0.28180500

H 4.25809100 0.66627100 0.78180900

H 4.52651800 1.11000900 -0.90924700

C 4.90281500 3.38261900 0.67688700

H 3.14798400 3.19622100 -0.57728100

H 2.87688100 2.75260800 1.10707200

H 4.72621400 4.44068000 0.92717500

H 5.36572000 2.90283500 1.55529900

H 5.63943000 3.35034200 -0.14311400

**[BuOCO_2_...HOCHPr]^.-^**

**Denoted by Int7**

-1 2

C -0.59744500 2.26040600 0.83348400

C -1.62803700 1.82054200 -0.20521900

H -0.20354000 1.38947300 1.38178200

C -3.77582500 0.47586600 -0.56826600

H -4.86060700 0.62845800 -0.50874200

O -3.42536900 -0.67571800 -1.19289900

H -2.46315700 -0.92732400 -1.01021500

C -2.85674000 1.12622400 0.41841600

H -3.43186700 1.86587000 1.00283100

H -2.48882700 0.37077400 1.14405800

H 0.25865600 2.77254800 0.36459000

H -1.03727400 2.95263700 1.57202600

H -1.96822500 2.68620600 -0.80106200

H -1.15672400 1.11623500 -0.90734600

C -0.61143400 -1.46100300 0.43203400

O -0.95030400 -1.33185600 -0.77805700

O 0.70325600 -1.10843900 0.73986900

O -1.27907100 -1.84587100 1.39579700

C 1.52683000 -0.61703400 -0.30684800

C 2.88311200 -0.25786500 0.27631100

H 1.06301800 0.26710700 -0.77654500

H 1.63209200 -1.37775800 -1.10109000

C 3.85554800 0.27881900 -0.77463600

H 2.74119000 0.49454100 1.07250400

H 3.31250000 -1.15048000 0.76521400

C 5.21971700 0.65285400 -0.19722100

H 3.98494600 -0.47679100 -1.57034400

H 3.40794000 1.16128000 -1.26647400

H 5.89859100 1.03540500 -0.97587100

H 5.12346100 1.43290300 0.57640200

H 5.70538400 -0.21886700 0.27228900

**[HOCO_2_...HOBu]^-^**

**Denoted by Int8**

-1 1

C -3.33462700 -1.43620500 -0.03046700

C -2.00438900 -0.72138800 0.19952400

H -3.77424400 -1.15506300 -1.00243200

C -0.81347800 1.55168500 0.37449700

H -1.02215300 2.61931200 0.56248600

H -0.28003400 1.17121900 1.27088500

O 0.01652400 1.48263500 -0.76804700

H 0.36037300 0.53500500 -0.86081100

C -2.13588100 0.80307300 0.21756100

H -2.80764800 1.10097100 1.04377500

H -2.61825400 1.14846700 -0.71542400

H -3.21427500 -2.53151500 -0.02347900

H -4.07033400 -1.17583300 0.74943600

H -1.56515000 -1.04992100 1.15940200

H -1.27483100 -1.02169100 -0.56930100

C 2.26901100 -0.54398600 -0.01603600

O 1.24229800 -0.76943000 -0.73279500

O 3.11086600 -1.35125700 0.38218500

O 2.46123800 0.78006500 0.36259300

H 1.73531800 1.30386500 -0.03650100

**[HOCO_2_...HOCHPr...H...NC_7_H_13_]^.^**

**Denoted by TS7**

0 2

C 4.32506800 -1.28957300 1.40345000

C 3.04759700 -0.78160500 0.73678400

H 5.22179700 -0.92686000 0.87388600

C 1.74509600 -0.70082800 -1.51484000

H 1.67767600 -1.18289800 -2.50594000

H 0.74793600 -1.06632900 -0.98424500

O 1.67894300 0.65500800 -1.67289900

H 1.61066700 1.15948000 -0.78522700

C 2.92032900 -1.25501400 -0.71178300

H 2.85584300 -2.35619200 -0.74277600

H 3.83652900 -0.98120300 -1.26874700

H 4.39387300 -0.95105200 2.44920900

H 4.36544700 -2.39168300 1.40425700

H 2.17229600 -1.12621200 1.31085900

H 3.01512400 0.31812000 0.77606000

C 0.07479500 2.61608300 0.41111800

O 1.15873200 1.94848200 0.45132700

O -0.78022100 2.73661400 1.28613300

O -0.19050800 3.25278800 -0.79314600

C -2.77524800 0.36103100 -0.55618300

C -3.07906200 -0.66468000 0.54667300

C -1.38538000 0.01464500 -1.17185800

H -3.54304400 0.33363100 -1.34269500

H -2.74646000 1.37489700 -0.13342400

H -1.47570000 -0.35727900 -2.20065500

H -0.70932600 0.87424800 -1.18305200

C -2.97015000 -2.08699900 -0.03254600

H -3.21611300 -2.83457600 0.73513500

H -3.67796200 -2.21642400 -0.86422300

C -1.50494300 -2.32119500 -0.54071300

H -0.98739700 -3.09419600 0.04101500

H -1.47944700 -2.59968300 -1.60192100

H -4.09155400 -0.50103400 0.94463100

C -0.62067500 -0.71071700 1.04850900

H -0.09421400 -1.54336600 1.53137400

H -0.00057300 0.19099100 1.11100200

C -2.03862500 -0.49245300 1.66238300

H -2.21574700 -1.21606500 2.47142600

H -2.08676800 0.52173000 2.08115300

N -0.78623500 -1.05687800 -0.37027100

H 0.56238000 3.09478200 -1.38247900

**[HOCO_2_...HOCHPr]^.-^**

**Denoted by Int9**

-1 2

C 2.09549600 2.12025900 -0.06242000

C 1.76084200 0.74469200 0.51047700

H 1.40803600 2.38305000 -0.88408300

C 1.46007900 -1.73799700 -0.03530000

H 2.12165500 -2.28360100 0.65181100

O 0.14110400 -2.00861400 0.15993700

H -0.44834300 -1.40476600 -0.43674400

C 1.87205100 -0.38829200 -0.53474700

H 2.91537800 -0.43950600 -0.89502400

H 1.24509700 -0.11420900 -1.40266100

H 2.01872600 2.91017700 0.70224800

H 3.12079900 2.14669400 -0.46911200

H 2.42934000 0.51549200 1.35952100

H 0.73641100 0.74753200 0.91486100

C -1.98751400 0.29001800 -0.11441300

O -1.40027000 -0.39664600 -1.01172200

O -2.81497700 1.18635900 -0.26259400

O -1.64536500 -0.01695300 1.20166200

H -0.99676100 -0.74610900 1.17933800

**CH_2_CHCN**

0 1

C -0.58655900 0.50769000 0.00002500

C -1.60888800 -0.36066300 0.00000600

H -0.75624000 1.58897800 -0.00003300

H -2.63940300 0.00128800 -0.00008000

H -1.44743500 -1.44141700 0.00000400

C 0.78404800 0.09179600 -0.00001000

N 1.90163900 -0.22597000 -0.00000300

**[CH_2_CHCN...HOCHPr]^.^**

**Denoted by Int10**

0 2

C 1.24073400 -0.31224800 -0.12390100

O 0.54544600 -1.47014500 -0.29371400

C 2.71663500 -0.45070700 0.03128300

H 3.09791800 -1.12993000 -0.75548100

H 2.96600200 -0.95747800 0.99237300

C 3.45905400 0.88694600 -0.03035800

C 4.97013200 0.74691800 0.14268600

H 5.40326800 0.10747600 -0.64470200

H 3.23508600 1.37790300 -0.99363900

H 3.05791600 1.55761900 0.75094600

H 5.47562600 1.72450000 0.09534500

H 5.21929500 0.28769200 1.11397100

H -0.41177000 -1.27181700 -0.21936100

H 0.71359800 0.50920900 0.38345400

C -4.53836200 0.17770000 0.38682800

C -5.05450800 1.19815800 -0.31455000

H -5.11228700 -0.33765700 1.16305300

H -6.07396600 1.53849700 -0.12029200

H -4.48246900 1.71364200 -1.08971200

C -3.21004800 -0.30690400 0.17247000

N -2.13623000 -0.71395400 0.01243000

**[CH_2_CHCN...HOCHPr]^.^**

**Denoted by TS8**

0 2

C 0.36751900 -0.79100300 0.40813800

O -0.49408500 -1.65005000 -0.20028200

C 1.73688800 -0.73658300 -0.16569400

H 1.66143600 -0.64827500 -1.26619600

H 2.26404400 -1.70095700 0.01013900

C 2.58203900 0.40808500 0.39963300

C 3.99592500 0.44685000 -0.17597400

H 3.97604300 0.57442300 -1.27113100

H 2.07042600 1.36551000 0.20157700

H 2.63070500 0.31129900 1.49879800

H 4.58037600 1.27858100 0.24762800

H 4.53934300 -0.48849900 0.03794000

H -1.33344500 -1.68055600 0.28227300

H 0.18476100 -0.55467300 1.46262800

C -2.19964600 1.34483600 0.22209300

C -1.06132000 1.43249400 -0.50009200

H -2.43033900 2.05536900 1.02105700

H -0.36199400 2.25381500 -0.33149100

H -0.84932300 0.74353300 -1.31743500

C -3.11186500 0.26230000 0.05421600

N -3.83379900 -0.64443400 -0.06106400

**[NCCHCH_2_CH(OH)Pr]^.^**

**Denoted by Int11**

0 2

C -0.09745100 1.07341400 0.38395600

O 0.80398700 1.81339000 -0.42035200

C -1.19864300 0.43170800 -0.46056900

H -0.73489100 -0.27020500 -1.17930900

H -1.65341800 1.23501900 -1.06485000

C -2.28742100 -0.29420300 0.33105700

C -3.42354300 -0.80004000 -0.55678500

H -3.04840500 -1.50281700 -1.31913500

H -1.84909200 -1.14632300 0.87801800

H -2.69180000 0.38727000 1.10062500

H -4.19221900 -1.32378300 0.03273900

H -3.91492200 0.03230300 -1.08714900

H 1.04752900 1.27451000 -1.18658200

H -0.55485500 1.80940300 1.06654800

C 0.67309500 0.07044400 1.29084400

C 1.32044000 -1.04175700 0.53372700

H 1.43485800 0.65931300 1.82670100

H -0.01789500 -0.34869700 2.03562900

C 2.47234000 -0.85146700 -0.22600600

N 3.44725000 -0.68697100 -0.85873700

H 0.85957100 -2.03291200 0.49339300

**[NCCHCH_2_CH(OH)Pr]^-^**

**Denoted by Int12**

-1 1

C -0.29158200 0.94244500 0.43074300

O 0.39399800 1.92501300 -0.32295500

C -1.49728800 0.40843700 -0.35592300

H -1.12723100 -0.15862600 -1.23002000

H -2.03499800 1.28197400 -0.76613300

C -2.47084600 -0.45225800 0.44976700

C -3.68236000 -0.90921000 -0.36242100

H -3.37214500 -1.51047800 -1.23360900

H -1.94523000 -1.33693700 0.84892600

H -2.81247500 0.11778800 1.33333900

H -4.36983900 -1.52442400 0.24025000

H -4.25307300 -0.04665200 -0.74583800

H 1.10717000 1.36059100 -0.73478700

H -0.67314500 1.42052100 1.35455900

C 0.73377200 -0.16060800 0.80916800

C 1.61960200 -0.50006800 -0.37792900

H 1.36538200 0.23537300 1.62323200

H 0.19368800 -1.02894900 1.23813200

C 2.99280800 -0.64927600 -0.20552800

N 4.16625200 -0.73487400 -0.07403900

H 1.18151600 -1.14293600 -1.15339500

**[NCCH_2_CH_2_CH(OH)Pr]**

**Denoted by Product**

0 1

C -0.43664800 1.19570100 0.43805900

O -0.11887300 2.38738500 -0.27471800

C -1.53214100 0.40672800 -0.28317900

H -1.18098400 0.13435000 -1.29667200

H -2.38167600 1.09421600 -0.43504600

C -2.01372200 -0.85113600 0.44159000

C -3.18977600 -1.52471900 -0.26408400

H -2.91881900 -1.82222300 -1.29072200

H -1.18385900 -1.57261100 0.53223700

H -2.30107200 -0.58650700 1.47474600

H -3.51598100 -2.42977900 0.27185800

H -4.05474200 -0.84464500 -0.33401600

H -0.12804200 2.18966700 -1.22133300

H -0.84014200 1.53254700 1.40862800

C 0.83737900 0.39706600 0.75219400

C 1.53852800 -0.14115500 -0.50693300

H 1.52846200 1.06404100 1.29001900

H 0.59976800 -0.43562200 1.43174700

C 2.80388000 -0.80587700 -0.20584700

N 3.80478700 -1.32957400 0.05485300

H 0.90123800 -0.86151500 -1.04448400

H 1.74833100 0.68637100 -1.20399200

**[DBU...CO_2_...HOH...HOBu]**

**Denoted by Complex2-3**

0 1

N -2.45578200 0.79893400 -0.03671500

N -0.30283700 1.15591500 -0.96031800

C -2.34375200 1.94667200 0.86334600

C -1.43566000 3.00424800 0.25290700

H -1.95200200 1.62811900 1.84788300

H -3.35189900 2.35190900 1.03469500

C -0.11922700 2.35263000 -0.15577900

H -1.92788400 3.43708100 -0.63425000

H -1.26764600 3.82110600 0.97117600

H 0.50669300 3.06011700 -0.72355200

H 0.46563000 2.10203800 0.75012300

C 3.33657300 0.26782900 1.59423800

O 4.06498300 0.25755900 0.36500500

C 1.87645400 0.51046300 -2.00910800

O 2.38657500 1.52320900 -1.72368900

O 1.59545900 -0.56272200 -2.37279200

C 1.97373100 -0.40664400 1.48725200

H 2.12466000 -1.43244100 1.11272400

H 1.36536900 0.11026400 0.72634300

C 1.21169300 -0.41574600 2.81183300

C -0.17415400 -1.04471800 2.69084400

H -0.10879500 -2.08857200 2.34235300

H 1.80272900 -0.95309200 3.57503500

H 1.11212000 0.62149700 3.18030500

H -0.71034200 -1.04353900 3.65291200

H -0.79017900 -0.49596000 1.96260800

C -1.41366800 0.49132700 -0.87446400

C -1.56623400 -0.73996200 -1.73924100

C -1.54518200 -2.06083100 -0.94797000

H -2.49694700 -0.67393400 -2.32775800

H -0.73346200 -0.72141800 -2.44774700

C -3.66891100 -0.01501300 0.04724300

C -2.88930200 -2.44247300 -0.32333700

H -0.76224500 -2.00040700 -0.17343000

H -1.23722600 -2.86787800 -1.63240300

C -3.47672900 -1.41379300 0.64575800

H -4.14282800 -0.09696200 -0.94646800

H -4.37243300 0.54880200 0.67389600

H -3.61657000 -2.60594300 -1.13977800

H -2.78850900 -3.41080800 0.19497200

H -4.46270300 -1.77421300 0.98394800

H -2.84952900 -1.32820700 1.54791500

H 3.70275600 0.94738800 -0.21129100

H 3.22577000 1.30550600 1.95868200

H 3.96308000 -0.26514200 2.32811600

O 3.61399100 -2.28960700 -0.76196400

H 2.85749900 -2.07858900 -1.32716600

H 3.87337700 -1.41211600 -0.41422300

**[DBU...CO_2_...HOH...HOBu]**

**Denoted by TS2-3**

0 1

C -4.42816400 -0.58280300 0.04183600

C -3.71612200 0.39055100 -0.91488500

C -1.38383000 0.13708500 -0.21227300

C -1.47992600 -1.24539500 -0.81386000

H -5.12699800 -1.18779600 -0.55923700

H -3.50865500 -0.09962100 -1.87587400

H -5.04062400 -0.01308700 0.75917100

H -4.36532900 1.24584400 -1.14094600

H -1.85481300 -1.12190100 -1.83973200

H -0.48082500 -1.67462900 -0.90136500

N -2.46555400 0.92995400 -0.36898600

N -0.27893600 0.51925200 0.40412700

C -2.47662400 2.31470100 0.11123900

C -1.05637900 2.84834200 0.17816200

H -2.96142700 2.36239100 1.10206400

H -3.09042200 2.90588900 -0.58338700

C -0.19946600 1.86885500 0.96341800

H -0.65433900 2.96409400 -0.84191700

H -1.04678900 3.83875200 0.65543500

H 0.85511500 2.17627000 0.96581300

H -0.51625900 1.84715600 2.02002300

C 0.84664000 -0.69346800 1.46545000

O 1.45257800 0.04658600 2.17267100

O 0.61904700 -1.81871100 1.13121700

C 4.94435300 -0.30564900 1.13236000

C 4.26714500 -0.21679400 -0.23396100

H 4.19681000 -0.24069700 1.93956300

C 2.68029400 1.14859000 -1.71694100

H 2.26437200 2.16441500 -1.85516300

H 3.30934100 0.93371400 -2.59678500

O 1.63035900 0.18825500 -1.73387000

H 1.00995000 0.37440200 -0.99676100

C 3.52255500 1.10349300 -0.44598200

H 4.24323200 1.94040800 -0.47811600

H 2.86897500 1.29647900 0.42224400

H 5.49082500 -1.25479900 1.25313800

H 5.66569000 0.51635500 1.27774400

H 5.01695000 -0.33959200 -1.03691700

H 3.56200200 -1.05423500 -0.35476800

O 1.78961800 -2.56455800 -1.39205000

H 1.81668900 -1.61229900 -1.62440700

H 1.48161000 -2.53566100 -0.47221700

C -2.40556100 -2.19907300 -0.01583600

C -3.47509300 -1.49298200 0.82490400

H -1.77599700 -2.79685300 0.65903000

H -2.88064700 -2.90050300 -0.72048800

H -4.06692800 -2.24769800 1.36687600

H -2.97183700 -0.89654100 1.60546900

**[DBU...HOBu...CO_2_...HOH...HOH]**

**Denoted by Complex1-3**

0 1

C -2.93512300 1.17744800 0.60594500

C -3.69686000 -1.51998400 -0.26669300

C -1.31175700 -0.79463100 0.18498300

C -1.73589000 0.35161600 1.08810900

H -4.11263800 -2.52708900 -0.41331700

H -4.00981600 -0.93118400 -1.15177200

H -1.93298200 -0.04510900 2.09882800

H -0.84818300 0.98700700 1.17678200

N -2.23916500 -1.70068700 -0.27036100

N -0.03910100 -0.85559100 -0.06905600

C -1.79205500 -2.75182000 -1.18858200

C -0.36138600 -3.16510100 -0.88824700

H -1.87947200 -2.40350500 -2.23622000

H -2.47549000 -3.60754100 -1.07963300

C 0.51973300 -1.92447600 -0.88164000

H -0.32231200 -3.65356100 0.10002200

H -0.01529900 -3.89808300 -1.63273900

H 1.52117600 -2.16743700 -0.49803400

H 0.66032000 -1.55662300 -1.91664300

C 3.66059300 0.36383900 1.46846200

O 4.32995300 1.09996700 0.86227700

O 3.10431800 -0.42286400 2.12308200

C -1.33832500 4.40184400 -0.90513700

C -0.09263000 3.69984000 -0.36718300

H -2.16767500 3.68894100 -1.04193400

C 1.67261900 1.88457600 -0.77229500

H 2.01467000 1.15983500 -1.53812000

H 2.48592600 2.62485500 -0.66295800

O 1.50639100 1.25328500 0.47514200

H 0.93805200 0.42784500 0.32553800

C 0.41361200 2.58503000 -1.28247300

H 0.62973700 2.99809500 -2.28499300

H -0.38108800 1.83083900 -1.42351400

H -1.68746700 5.19186600 -0.22101000

H -1.14154400 4.86963100 -1.88448300

H 0.71604500 4.43897800 -0.21947500

H -0.30113800 3.27870300 0.63034100

C -4.31618400 -0.92853900 0.99836500

C -4.27923000 0.60196800 1.06276200

H -3.81231800 -1.37239000 1.87188900

H -5.36260600 -1.26863500 1.04882300

H -4.48806800 0.91942400 2.09828400

H -5.08825900 1.02103900 0.44126400

H -2.82969800 2.21016700 0.97225700

H -2.90370300 1.24825200 -0.49417800

O 3.59903900 -2.90289400 0.39816500

H 3.25444100 -2.41725300 1.16055400

H 3.75501000 -2.19116500 -0.25474500

O 3.93311700 -0.81108600 -1.41745600

H 4.25110300 0.07057700 -1.17259100

H 3.25590800 -0.65343700 -2.09025900

**[DBU...HOBu...CO_2_...HOH...HOH]**

**Denoted by TS1-3**

0 1

C -2.85365200 1.31940200 0.55206800

C -3.74129000 -1.35262900 -0.29641700

C -1.32757900 -0.72485400 0.12953500

C -1.68953700 0.44265100 1.03027300

H -4.19326500 -2.34630500 -0.42402100

H -4.04460700 -0.76375100 -1.18399500

H -1.89746200 0.05533700 2.04208300

H -0.77425000 1.03736800 1.11870300

N -2.28945100 -1.58391600 -0.32423400

N -0.05758700 -0.85138300 -0.13203400

C -1.89068200 -2.65726100 -1.24150800

C -0.48474300 -3.14370800 -0.93275300

H -1.95539200 -2.30180400 -2.28762700

H -2.61773900 -3.47583800 -1.13571900

C 0.46218700 -1.95386300 -0.92867000

H -0.47654700 -3.63051800 0.05684600

H -0.17498600 -3.89577800 -1.67373000

H 1.44236800 -2.23711100 -0.51863000

H 0.64088600 -1.60203700 -1.96201200

C 3.08508600 0.33500000 1.45833200

O 4.03618200 0.88123000 1.00418600

O 2.55414300 -0.42037300 2.20069000

C -0.93698700 4.38993300 -0.94558700

C 0.24552400 3.59882700 -0.38945700

H -1.80545300 3.73418100 -1.12057300

C 1.89415200 1.67575900 -0.79868200

H 2.22940300 0.94393200 -1.55487400

H 2.74643800 2.34689300 -0.60147400

O 1.59758300 1.01256300 0.42190000

H 0.90672700 0.21066900 0.23988000

C 0.70550400 2.47629400 -1.32031500

H 0.98999000 2.90079800 -2.30004600

H -0.13393800 1.78624700 -1.51727500

H -1.25437700 5.18576500 -0.25310000

H -0.68288100 4.86437400 -1.90827000

H 1.09500400 4.27996000 -0.20054700

H -0.02071800 3.17044300 0.59101900

C -4.31863100 -0.72454000 0.97028200

C -4.21788500 0.80351300 1.02098100

H -3.82522700 -1.18210100 1.84262800

H -5.37753700 -1.02053000 1.03424400

H -4.40713900 1.13746800 2.05492100

H -5.01218100 1.25057700 0.40029100

H -2.69994400 2.34724700 0.91458700

H -2.82635900 1.38494700 -0.54841400

O 3.40446700 -2.86739700 0.60380200

H 3.01714600 -2.30135200 1.28819300

H 3.67673900 -2.22385900 -0.08372200

O 4.04910300 -0.89188200 -1.27712500

H 4.34840100 -0.14665300 -0.72794600

H 4.79554600 -1.08175400 -1.86366900

**[DBUH...BuOCO_2_...HOH...HOH]**

**Denoted by Int1-3**

0 1

N -2.12417000 -0.75503100 -0.31569300

N -0.61088900 0.19998600 -1.79260500

C -2.44379100 -1.82519400 -1.27401600

C -1.25047500 -2.12206600 -2.17023300

H -3.32333700 -1.52726900 -1.86986500

H -2.72213700 -2.71609300 -0.69614400

C -0.75218100 -0.83540600 -2.80951200

H -0.44375700 -2.57121700 -1.57011000

H -1.53862100 -2.84892500 -2.94205400

H 0.23512300 -0.97441000 -3.26897600

H -1.44806100 -0.48954800 -3.59194900

C 2.50879300 -1.66105000 -0.33563100

O 2.13749900 -0.68898200 -1.31585200

C 2.45847100 0.62680600 -1.05516000

O 1.81967300 1.44394800 -1.77349500

O 3.29001700 0.87587000 -0.16404700

C 1.58492600 -1.60831200 0.87546900

H 0.54296100 -1.73720500 0.53555600

H 1.65402700 -0.60522800 1.32681900

C 1.90468200 -2.66398400 1.93284900

C 0.96122600 -2.58993700 3.13278900

H -0.08650200 -2.73966200 2.82212500

H 1.85138200 -3.66986500 1.47883500

H 2.94837100 -2.53824600 2.27249400

H 1.20046500 -3.35525600 3.88788400

H 1.02111600 -1.60347200 3.62196900

C -1.22360500 0.18056600 -0.62292100

C -0.89248600 1.27331900 0.34989100

C -2.05219000 2.25526100 0.59716800

H -0.55734000 0.82763900 1.29900400

H -0.04603800 1.84183900 -0.04583500

C -2.85908700 -0.73751800 0.95991200

C -3.12068600 1.74842100 1.56426400

H -2.50704600 2.52404200 -0.37177800

H -1.60746900 3.18052400 0.99450300

C -3.80758300 0.45153900 1.13267200

H -2.13964200 -0.78398100 1.79397900

H -3.43084300 -1.67241000 0.98742800

H -2.65478400 1.58750600 2.55317500

H -3.88290300 2.53181500 1.70612200

H -4.55156300 0.17149700 1.89572900

H -4.36361500 0.59963500 0.19111300

H 0.22745000 0.81639500 -1.87822400

H 2.43256400 -2.63403900 -0.84471100

H 3.55461600 -1.50345300 -0.03104300

O 1.99616700 1.99433200 1.89834100

H 2.57564100 1.59017600 1.20385100

H 2.61026800 2.27806900 2.58986800

O 1.30685200 3.70764400 -0.23168600

H 1.56904200 3.01807400 -0.87856700

H 1.52829200 3.27121100 0.61555400

**[DBU...HOH...CO_2_...HOBu...HOH]**

**Denoted by Complex1-4**

0 1

C -1.31192600 3.28548600 -0.86841000

C 0.98442300 2.48208200 0.77756100

C -1.11231200 1.09934200 0.49604500

C -1.47889300 1.75957600 -0.82158200

H 1.96058500 2.22822500 1.21313200

H 0.62216500 3.36381100 1.34142200

H -0.89675700 1.28849400 -1.63113000

H -2.52422900 1.48591900 -1.00153500

N 0.12609300 1.32100000 1.05248300

N -2.00926300 0.30349900 0.99408300

C 0.48102800 0.63378400 2.29917200

C -0.28357100 -0.66887800 2.45785800

H 0.28856800 1.30030900 3.16168700

H 1.56600500 0.44505200 2.27997900

C -1.76475500 -0.38984600 2.24799200

H 0.05976500 -1.41204500 1.72039600

H -0.09217200 -1.08898300 3.45726600

H -2.34565700 -1.32573300 2.24178800

H -2.16043400 0.21639500 3.08535300

C -2.18939100 -1.97453900 -0.96949900

O -2.44281800 -2.66280300 -0.06522200

O -1.83726800 -1.38245800 -1.90945500

O -4.19246100 -0.35481400 -0.47720500

H -3.45336100 -0.05503600 0.13216700

C 1.19730400 2.84855500 -0.69054800

C 0.09357400 3.72583400 -1.29100300

H 1.31524700 1.91961600 -1.26822600

H 2.16202700 3.37423600 -0.76806900

H 0.17784900 3.69694400 -2.39034600

H 0.25047400 4.77690600 -0.99611500

H -2.05350300 3.70520300 -1.56585400

H -1.56292500 3.70489300 0.12041200

H -4.73533100 -0.92627500 0.08238500

O 0.97651700 -2.18853900 -1.83275800

H 0.16270500 -1.92235400 -2.28551500

C 1.63950400 -1.00953700 -1.37390200

C 2.95402200 -1.40324800 -0.72432800

H 1.01092800 -0.46667400 -0.64628000

H 1.82627400 -0.32726600 -2.22392300

C 3.73025900 -0.20352100 -0.18142500

H 2.74030300 -2.11208200 0.09406100

H 3.56726500 -1.94816800 -1.46347500

C 5.05362300 -0.59382600 0.47331900

H 3.91633900 0.51526800 -0.99916000

H 3.10216400 0.33065700 0.55158700

H 5.58739200 0.28764300 0.86208800

H 4.88924400 -1.28599500 1.31579700

H 5.71926500 -1.09998600 -0.24538500

O 0.49175400 -3.53023500 0.55547100

H 0.62541800 -3.14100000 -0.33485500

H -0.46655800 -3.49624600 0.68025700

**[DBU...HOH...CO_2_...HOBu...HOH]**

**Denoted by TS1-4**

0 1

C -0.29178800 3.50524600 -1.03121400

C 1.63977100 2.20803200 0.75687200

C -0.74502400 1.44371900 0.46205100

C -0.89122200 2.09787300 -0.89978300

H 2.48937400 1.71869800 1.25125800

H 1.52921700 3.19394000 1.24672200

H -0.46239200 1.42458100 -1.66056600

H -1.96850000 2.12763500 -1.09778100

N 0.47551500 1.36339300 1.06789900

N -1.83750000 0.93530200 0.96089400

C 0.60402200 0.64569800 2.34363900

C -0.48310200 -0.40167500 2.51223700

H 0.57830300 1.36942800 3.17938200

H 1.59877900 0.17445500 2.35958300

C -1.83111800 0.25028900 2.24541800

H -0.33670000 -1.23743300 1.80891000

H -0.43654400 -0.81756000 3.52999700

H -2.63761400 -0.49930600 2.23462900

H -2.07772300 0.96988800 3.04821200

C -2.78984100 -1.30873100 -0.92325600

O -3.12725200 -2.06225800 -0.06967100

O -2.13308200 -1.03222200 -1.87531800

O -3.75719700 0.31182300 -0.57339900

H -2.99819300 0.69092200 0.10608600

C 1.98006500 2.38607100 -0.72188200

C 1.18932000 3.49534700 -1.42261800

H 1.83554500 1.42175400 -1.23124900

H 3.05707900 2.60400500 -0.79375800

H 1.28545900 3.36358900 -2.51325600

H 1.63420700 4.47670800 -1.18812500

H -0.86755800 4.06720600 -1.78259500

H -0.43397000 4.04672400 -0.08107700

H -4.50878700 0.07074600 -0.00936500

O 0.27564500 -2.46156000 -1.73830200

H -0.53881400 -2.01367200 -2.03048100

C 1.18493900 -1.46210000 -1.29001900

C 2.40428300 -2.13506600 -0.68443100

H 0.70921900 -0.80875500 -0.53709600

H 1.49066800 -0.81666700 -2.13604200

C 3.44384500 -1.14134300 -0.16716500

H 2.06684200 -2.78513700 0.14156800

H 2.85780800 -2.79726600 -1.44294100

C 4.66783500 -1.81903000 0.44520400

H 3.75951800 -0.47623800 -0.99037500

H 2.97267200 -0.48650300 0.58540900

H 5.39519100 -1.08014400 0.81680900

H 4.38098800 -2.46389100 1.29242100

H 5.18236200 -2.45458600 -0.29450100

O -0.59824900 -3.42504600 0.72062100

H -0.28188400 -3.20668100 -0.18188200

H -1.51149100 -3.09919900 0.70679000

**[DBUH...HOCO_2_...HOBu...HOH]**

**Denoted by Int1-4**

0 1

C -3.54531100 -0.86477200 1.61548600

C -3.95792300 -0.09904400 0.34851300

C -1.84790100 0.07917700 -0.83940600

C -1.91862700 -1.40449000 -1.07350100

H -4.29476000 -1.65545700 1.78337400

H -4.32160100 -0.79348800 -0.42069400

H -3.59129900 -0.19428500 2.48854100

H -4.78347900 0.59255800 0.55881600

H -2.85035200 -1.60761800 -1.62158300

H -1.09055900 -1.68241900 -1.73578100

N -2.85936000 0.69255700 -0.21881700

N -0.77391000 0.73121500 -1.23983400

C -2.78723000 2.12383400 0.10361500

C -1.91524600 2.85736900 -0.90688600

H -2.39235400 2.24562100 1.12696200

H -3.81193100 2.51941300 0.09527000

C -0.56870500 2.16062000 -1.03149400

H -2.41969600 2.87121500 -1.88609400

H -1.77683600 3.89968000 -0.58819900

H 0.02025400 2.54231400 -1.87533600

H 0.03647800 2.30887700 -0.12129200

C 2.99816000 2.57425500 2.28715300

C 2.67755800 1.31510700 1.48488600

H 3.61035200 2.33694200 3.17344200

C 1.53779300 -0.99178000 1.51618700

H 0.91507600 -1.63894500 2.15594600

H 0.91874500 -0.72058300 0.64210900

O 2.65555700 -1.76616600 1.10891700

H 3.03716700 -1.34472100 0.29850100

C 1.90830400 0.26846100 2.29248300

H 0.97224200 0.71614800 2.67520300

H 2.50008100 -0.02271500 3.17930300

H 3.55453700 3.30931600 1.68379100

H 2.07697900 3.06399100 2.64571300

H 2.09142900 1.58596400 0.58943900

H 3.60684400 0.86638100 1.09878600

C -1.84829400 -2.24578300 0.23418200

C -2.14019900 -1.47172400 1.52399200

H -0.83626200 -2.67237500 0.28242300

H -2.54763500 -3.09198200 0.14223900

H -1.98038500 -2.13916900 2.38526700

H -1.39375100 -0.66732400 1.63613600

C 2.39711000 -0.15107400 -1.75497500

O 3.41574500 -0.55368500 -1.16194900

O 2.37635500 1.15911400 -2.17025100

H 0.00315100 0.17025400 -1.66007800

O 1.33865000 -0.78463900 -2.01344300

H 3.20951800 1.55900400 -1.87561700

O 1.21913000 -3.27613400 -0.77915400

H 1.23912700 -2.45152500 -1.31280100

H 1.75138600 -3.00034400 -0.00759500

**HOBu...2H_2_O**

0 1

C -0.06139200 -0.91805200 0.05546300

O 1.06022500 -0.63360400 0.88140300

C -1.26338800 -0.04110700 0.38458000

H -0.97312400 1.01595300 0.24199600

H -1.50337200 -0.15842400 1.45628200

C -2.49348200 -0.35564900 -0.46739500

C -3.69076100 0.53734700 -0.14533200

H -3.44846600 1.60056300 -0.30956400

H -2.23180900 -0.25181000 -1.53581100

H -2.77281200 -1.41523200 -0.32577400

H -4.56072800 0.29070700 -0.77433000

H -3.99665400 0.42756800 0.90843300

H 1.27955400 0.31842600 0.74411100

H -0.31236500 -1.98090800 0.20920000

O 2.02449300 1.72092200 -0.06089100

H 2.65308800 1.05655500 -0.43246600

O 3.34256700 -0.59873600 -0.64219300

H 2.61363500 -0.90400600 -0.05383300

H 4.13224900 -0.65470500 -0.08401200

H 2.55623900 2.21400100 0.58098500

H 0.20041800 -0.80258300 -1.01568000

**[2H_2_O...HOCHPr...H...NC_7_H_13_]^.+^**

**Denoted by TS3’**

1 2

C 1.81799800 0.40762100 -0.96758900

O 1.74812000 1.77281500 -0.78729200

C 2.80344000 -0.31189100 -0.05888700

H 2.49273400 -0.17428800 0.99250800

H 3.77957700 0.19620000 -0.16131400

C 2.95503600 -1.79916900 -0.37916800

C 3.92605400 -2.50937200 0.56190700

H 3.58319300 -2.44514000 1.60770700

H 1.97029700 -2.29341800 -0.33041400

H 3.29673600 -1.90965000 -1.42289700

H 4.02497700 -3.57541900 0.30509900

H 4.93000800 -2.05662400 0.51312100

H 1.71348000 1.99884000 0.18515300

C -0.72765400 -1.63115700 0.57802200

C -2.18938500 -1.97209200 1.03069500

H -0.27526200 -2.45509500 0.01323900

H -0.08252800 -1.38431200 1.43014500

H -2.44116400 -2.99533500 0.71855000

H -2.25601400 -1.92697600 2.12668100

C -1.53729600 -0.72353400 -1.53607100

H -1.46710600 0.16737500 -2.17215200

H -1.05672200 -1.56632100 -2.04806600

C -3.01984000 -1.04495500 -1.13977100

H -3.69501400 -0.32659400 -1.62538000

H -3.28604100 -2.05095300 -1.49343200

C -2.75880000 0.45494900 0.84629000

H -3.42667400 1.20436200 0.39882900

H -2.83562200 0.54271700 1.93907700

C -1.28215000 0.73542200 0.39959200

H -1.22169500 1.59179100 -0.28142200

H -0.62135600 0.93064000 1.25280100

N -0.80748200 -0.45962500 -0.29538900

H 1.98344500 0.19470700 -2.03744200

H 0.76501000 -0.05627600 -0.75934800

C -3.14802700 -0.95837000 0.38879200

H -4.18265300 -1.17860000 0.69056500

O 1.08733400 2.62776800 1.60757700

H 0.51118200 3.22406100 1.07236600

O -0.26919500 3.68671100 -0.50045700

H 0.37349700 3.16826400 -1.02212600

H -0.17733400 4.59674200 -0.81767400

H 1.68709400 3.21362200 2.09206400

**[2H_2_O...HOCHPr]^.^**

**Denoted by Int3’**

0 2

C -0.04614000 -1.40044400 -0.14994100

O 1.30266100 -1.35238800 -0.37298100

C -0.84090600 -0.13533800 -0.17611900

H -0.74591400 0.35286100 -1.16609600

H -0.41152800 0.59676700 0.54408200

C -2.32166500 -0.34915900 0.14368400

C -3.13183400 0.94496000 0.11787100

H -3.08998100 1.42118800 -0.87589000

H -2.74335700 -1.07159900 -0.57671700

H -2.40946700 -0.82328400 1.13734300

H -4.19115200 0.76306900 0.35806300

H -2.74140500 1.67209000 0.84930100

H 1.53000100 -0.53252800 -0.88763700

O 2.08366000 1.06309400 -1.20433400

H 2.22715700 1.18043700 -0.23559300

O 2.34656800 0.55602000 1.44615000

H 2.07559100 -0.32918200 1.13210400

H 1.64573700 0.81629800 2.06180400

H 1.36941200 1.67842800 -1.42433100

H -0.33494100 -2.21847400 0.51991500

**[DBUCO_2_...HOH...HOBu]**

**Denoted by Int2-3**

0 1

C -4.36598900 -2.00223300 0.33080400

C -4.30352800 -0.95335400 -0.79369900

C -2.13796400 0.07584200 -0.35843600

C -1.54360600 -1.23801300 -0.79920400

H -4.69043200 -2.95229700 -0.12403300

H -3.92881600 -1.40116500 -1.72318600

H -5.14426500 -1.71680600 1.05626500

H -5.30540900 -0.56840900 -1.01789100

H -2.00218000 -1.46337200 -1.77232100

H -0.47027800 -1.13355600 -0.97480600

N -3.46530400 0.20600100 -0.45062600

N -1.37048400 1.07744400 0.07167000

C -4.13428200 1.45339400 -0.06367900

C -3.22935400 2.63014900 -0.38335600

H -4.38187000 1.42102500 1.01120200

H -5.07716500 1.51252800 -0.62198700

C -1.88979400 2.43611900 0.29907000

H -3.09605300 2.70203700 -1.47445300

H -3.68926000 3.56698700 -0.03979200

H -1.12955800 3.12755000 -0.08289800

H -1.97464000 2.59837600 1.38625800

C 0.07485200 0.87790500 0.43253900

O 0.79531100 1.84226200 0.12681500

O 0.33806000 -0.18384900 1.00949900

C 7.17038100 -0.93316300 -0.53624800

C 6.09782400 0.12779900 -0.29730200

H 6.73837900 -1.83537700 -1.00108000

C 3.86190000 0.66935100 0.81695900

H 3.10969100 0.27238000 1.52301900

H 4.28137900 1.59111800 1.27038800

O 3.25259400 0.96798800 -0.43061800

H 2.38364600 1.39926800 -0.23371200

C 4.96982900 -0.35343300 0.61637400

H 5.37940700 -0.62778500 1.60495100

H 4.51160700 -1.26589200 0.19622400

H 7.96837600 -0.56413100 -1.20031900

H 7.64136700 -1.24581400 0.41109100

H 6.55805900 1.03174100 0.14232300

H 5.66218800 0.44571700 -1.25911900

O 1.99737300 -1.49543700 -0.79591700

H 2.50272000 -0.66137200 -0.90309200

H 1.45479400 -1.26056000 -0.01854300

C -1.80820100 -2.39506700 0.19654000

C -3.04286000 -2.19633400 1.08139000

H -0.92770300 -2.48424400 0.84753500

H -1.89463500 -3.33325700 -0.37406900

H -3.14352900 -3.05649300 1.76167100

H -2.86724200 -1.32381300 1.73433300

**[DBUCO_2_...HOH...HOCHPr...H...NC_7_H_13_]^.+^**

**Denoted by TS4’**

1 2

N -3.18633000 -1.08688000 -0.95839700

N -1.43119800 -1.68440900 0.45264600

C -2.51150900 -1.70617100 -2.10888100

C -1.78686900 -2.95204100 -1.63981300

H -1.81480600 -0.97781800 -2.55745700

H -3.27765400 -1.94309700 -2.85601000

C -0.80649000 -2.57048900 -0.55281200

H -2.52232700 -3.68199600 -1.26611900

H -1.24604100 -3.41895700 -2.47436000

H -0.42669100 -3.44699100 -0.01597500

H 0.06757300 -2.05665800 -0.98096300

C 3.44382400 -0.68584700 1.12471200

O 2.70041300 -0.83002600 2.28226600

C -0.54321200 -1.33702800 1.59238900

O 0.48636600 -2.03928900 1.63659700

O -0.87162000 -0.37476600 2.29830300

C 3.54268000 -1.94007100 0.26733800

H 2.52566400 -2.25942800 -0.01585000

H 3.95412400 -2.74615400 0.90195200

C 4.40959500 -1.76098200 -0.97809000

C 4.49341700 -3.02971000 -1.82472600

H 3.49331900 -3.34393700 -2.16637700

H 4.00697100 -0.93781900 -1.59201200

H 5.42363800 -1.44555600 -0.67577700

H 5.12144400 -2.87786200 -2.71637000

H 4.92401200 -3.86484700 -1.24802100

C -2.63742200 -1.11194300 0.25119600

C -3.42133300 -0.52261200 1.39412000

C -3.47987800 1.01704000 1.39097400

H -4.44106200 -0.93417500 1.32000200

H -2.98907700 -0.87732700 2.32900300

C -4.45394800 -0.37764800 -1.22739500

C -4.52212900 1.59148100 0.43272800

H -2.47623700 1.40696700 1.16746200

H -3.71234600 1.34334700 2.41601100

C -4.37213400 1.13814600 -1.02076400

H -5.25876700 -0.81740100 -0.61765300

H -4.69727500 -0.59579800 -2.27268100

H -5.52755400 1.30146600 0.78641100

H -4.48684200 2.69209200 0.47339600

H -5.17651300 1.59276700 -1.62040100

H -3.42121800 1.49909000 -1.44782800

H 1.86548400 -1.35922300 2.09467500

H 4.43601200 -0.27895100 1.38850700

H 2.98628600 0.14754900 0.46651000

O 1.12667900 1.31495800 3.20176100

H 0.33120400 0.77313600 3.01258800

H 1.83847800 0.66633300 3.04081400

C -0.15251500 2.19929700 -0.80736800

C 0.77643400 3.31940900 -1.29794000

C 0.73270200 1.06127700 -0.19130300

H -0.84721000 2.57656800 -0.04405100

H -0.75023500 1.79414400 -1.63615900

H 0.53056500 0.92670200 0.87474400

H 0.59777500 0.11196000 -0.72172300

C 1.60055700 3.84050900 -0.11106300

H 2.27822200 4.64349800 -0.43336500

H 0.94106200 4.24574600 0.66935600

C 2.43674300 2.64883300 0.48053100

H 3.51604900 2.83605600 0.42171900

H 2.14126400 2.42906300 1.51519100

H 0.18167800 4.13789300 -1.73012000

C 2.56055000 1.58263200 -1.71570800

H 3.63795100 1.78717400 -1.73085400

H 2.36788500 0.62704600 -2.21834200

C 1.73113800 2.75226500 -2.35941400

H 2.41598600 3.53246700 -2.71979300

H 1.16871200 2.36901200 -3.22224500

N 2.12389800 1.47397700 -0.32875500

**[DBUCO_2_...HOH...HOCHPr]^.^**

**Denoted by Int4’**

0 2

N 2.77905000 -0.85228500 -0.15793900

N 0.61249800 -0.51390000 -0.94920100

C 2.56058200 -2.30048800 -0.02416200

C 1.59563600 -2.76914300 -1.09468100

H 2.17719100 -2.51962400 0.98666300

H 3.53541500 -2.79315100 -0.12581900

C 0.32116300 -1.95885400 -1.00704500

H 2.06027300 -2.64824900 -2.08628200

H 1.36766000 -3.83538900 -0.95949400

H -0.32560700 -2.11235000 -1.87707100

H -0.25965300 -2.23259000 -0.11083400

C -4.40200700 -0.15452600 -0.26675400

O -4.04166400 0.26635600 -1.51443500

C -0.58910000 0.35926400 -1.13857500

O -1.46752500 -0.16371400 -1.84668100

O -0.58343400 1.43902300 -0.53517800

C -3.36320600 -0.47592300 0.75123700

H -2.67136700 0.38480700 0.82075400

H -2.72931500 -1.33140600 0.42557000

C -3.94082000 -0.78260100 2.13421600

C -2.86186000 -1.09656800 3.16870400

H -2.16346500 -0.25030400 3.28040500

H -4.54460600 0.07839800 2.47102500

H -4.64148800 -1.63328400 2.05647600

H -3.29562800 -1.30766000 4.15895900

H -2.26728900 -1.97561200 2.86820200

C 1.82307600 -0.04335200 -0.61453000

C 2.15690100 1.41575300 -0.78285000

C 2.25821000 2.18450200 0.54918000

H 3.11764300 1.46707300 -1.31943900

H 1.40221900 1.87646000 -1.42013800

C 4.08066600 -0.33597300 0.31016800

C 3.60416300 2.01505700 1.25242400

H 1.42663300 1.87258500 1.19896600

H 2.09552400 3.25136800 0.33248400

C 3.99107500 0.56435300 1.54641600

H 4.59323100 0.18020600 -0.51689200

H 4.68097000 -1.22173400 0.54468500

H 4.39234000 2.46670400 0.62365000

H 3.59782300 2.58554600 2.19525700

H 4.97995600 0.55110500 2.03186800

H 3.28502400 0.10871900 2.26144900

H -3.05822400 0.14672500 -1.64784200

H -5.43999400 -0.49281600 -0.17988100

O -3.00893100 2.79700600 -0.53364900

H -2.11669900 2.39102300 -0.53611900

H -3.53386100 2.11838500 -0.98957400

**[2H_2_O...OOCOBu]^-^**

**Denoted by Int1-1’**

-1 1

C -1.07333400 -1.51177100 0.23453100

O 0.21420900 -1.79570400 -0.30232000

C 1.28447800 -1.04334800 0.17203200

O 2.34401600 -1.22787900 -0.45325100

O 1.06613300 -0.28256000 1.14687300

C -1.69166800 -0.25983100 -0.37893800

H -1.74715900 -0.39047700 -1.47481900

H -1.01898900 0.59271200 -0.19174900

C -3.08070000 0.05179100 0.17849500

C -3.69604700 1.31463600 -0.42285900

H -3.80926800 1.22016300 -1.51581900

H -3.75216700 -0.80826500 0.00214800

H -3.01374300 0.16212700 1.27611600

H -4.69149600 1.52408100 0.00020000

H -3.05821400 2.19405200 -0.23295700

H -1.69330000 -2.39534400 0.00932700

O 0.99969000 2.23458200 0.33906300

H 1.01925900 1.32085900 0.73644800

H 1.12388600 2.82420300 1.09595400

O 3.53173100 1.28809800 -0.58719300

H 3.23638000 0.35340800 -0.52340000

H 2.71073800 1.76283800 -0.35485500

H -1.00853800 -1.40151500 1.32846600

**[2H_2_O...O_2_COCHPr...H...NC_7_H_13_]^.^**

**Denoted by TS5’**

0 2

C 1.05188800 0.77733000 -1.53100600

O 1.53950100 -0.39973100 -2.07962000

C 1.92910700 -1.40541800 -1.12999700

O 3.11659300 -1.71940900 -1.16524000

O 0.98883900 -1.81075800 -0.41849400

C 1.98735200 1.44249100 -0.52869400

H 2.90869300 1.71592800 -1.07583400

H 2.30190000 0.71663800 0.23936000

C 1.39261200 2.68123000 0.13817800

C 2.38661200 3.37052900 1.07168900

H 3.27176800 3.72311800 0.51711900

H 1.04534600 3.39202100 -0.63244200

H 0.49612500 2.39367700 0.71328600

H 1.93438000 4.24065800 1.57253400

H 2.74075800 2.67577900 1.85089700

C -3.74386200 1.14552800 -0.02552700

C -2.32065400 1.41636200 -0.62348600

H -4.50227000 1.27261100 -0.81091400

H -3.95808000 1.87330100 0.77015000

H -2.35222100 1.55799200 -1.71096900

H -1.84713500 2.29442200 -0.16698300

C -3.40476200 -1.26643800 -0.59098800

H -3.45468400 -2.30396600 -0.23080600

H -4.10546900 -1.16840300 -1.43262800

C -1.94659500 -0.95055900 -1.06480700

H -1.23560600 -1.75225400 -0.84296900

H -1.90452500 -0.73045100 -2.13949600

C -1.33659300 0.00645700 1.09824500

H -0.57897200 -0.76817300 1.25579700

H -0.98394500 0.93967100 1.55478600

C -2.73889200 -0.40969300 1.65997800

H -2.69777800 -1.44407200 2.03069500

H -3.00317600 0.24085500 2.50575900

N -1.50047200 0.23958000 -0.33687600

H 0.77689400 1.44622500 -2.36256900

H 0.05129300 0.55339200 -0.99064600

C -3.77525500 -0.28620300 0.53242000

H -4.77979400 -0.51619800 0.91746900

O 1.24158400 -1.99666700 2.19947300

H 1.15220600 -2.08218000 1.21440700

H 1.29780600 -2.90655900 2.52354600

O 3.67538900 -0.79939200 1.52991800

H 3.68604800 -1.15895000 0.62384500

H 2.86274100 -1.20418000 1.90041000

**[2H_2_O...O_2_COCHPr]^.-^**

**Denoted by Int5’**

-1 2

C -1.12023800 -1.87026200 -0.16965200

O 0.23481000 -2.05962700 -0.17075400

C 1.10383100 -1.04141400 0.29362000

O 2.27841200 -1.20545200 -0.06708400

O 0.59388900 -0.14074100 0.99208400

C -1.74281300 -0.59590800 -0.64965600

H -2.43849000 -0.81922000 -1.48321600

H -0.95822000 0.05701500 -1.06352400

C -2.51297500 0.19688800 0.42407900

C -2.95232300 1.57423100 -0.07006700

H -3.57288400 1.50090500 -0.97975700

H -3.38710000 -0.39150800 0.75622800

H -1.85014700 0.30739200 1.29489800

H -3.53971800 2.11193400 0.69200200

H -2.06553500 2.18265500 -0.31047900

H -1.65849900 -2.82163000 -0.22317300

O 0.59099500 2.23997600 -0.20521200

H 0.54585600 1.38879400 0.30723500

H 0.48740700 2.92320200 0.47206400

O 3.33299300 1.38102800 -0.28872500

H 3.07430900 0.44318800 -0.16092500

H 2.46133500 1.81459400 -0.35377300

**[BuOCO_2_...H_2_O...HOBu]^-^**

**Denoted by Int6’**

-1 1

C 1.02050000 2.06814500 -1.62148000

C 1.51679400 1.94045900 -0.18239900

H 1.00996200 1.07965400 -2.10690200

C 3.34043700 0.92158500 1.31707000

H 4.43892900 0.93569000 1.42052600

H 2.94555200 1.62859000 2.07757100

O 2.93127800 -0.40469800 1.60400600

H 1.98009300 -0.55559500 1.34616800

C 2.94848800 1.40247800 -0.08349600

H 3.65621400 2.18477600 -0.41230700

H 3.08226100 0.54280800 -0.76001000

H -0.00311200 2.47503200 -1.66356100

H 1.66993000 2.73359500 -2.21614400

H 1.46406700 2.92054300 0.32609600

H 0.84378300 1.26983500 0.37217800

C 0.13186500 -1.14157700 -0.31855900

O 0.44316300 -0.99488400 0.88884300

O -1.17671400 -0.85557500 -0.66952800

O 0.83746400 -1.50659900 -1.27800300

C -2.04791700 -0.37596600 0.34608300

C -3.43565300 -0.21616500 -0.25056900

H -1.68029000 0.58943800 0.73827100

H -2.06376400 -1.07706200 1.19812000

C -4.45988500 0.29859700 0.76137800

H -3.37792800 0.47420600 -1.11079500

H -3.76412500 -1.18999700 -0.65509500

C -5.85935000 0.46018000 0.17005600

H -4.49936300 -0.39292900 1.62218800

H -4.11578200 1.26689700 1.16736200

H -6.57575400 0.83116800 0.92006900

H -5.85547600 1.17292200 -0.67145300

H -6.24228800 -0.50039100 -0.21312100

O 3.48394400 -1.89597700 -0.71950400

H 2.53472700 -1.80495700 -0.97391000

H 3.46761200 -1.46878100 0.15973600

**[BuOCO_2_...H_2_O...HOCHPr...H...NC_7_H_13_]^.^**

**Denoted by TS6’**

0 2

C -1.14586200 2.42799400 2.69141400

C 0.00539100 1.77049900 1.93408900

H -1.78448000 2.99401800 1.99490600

C 1.90534900 2.22667900 0.22663900

H 2.86130300 2.77652400 0.20174100

H 2.21244000 1.16009200 0.55875500

O 1.41751300 2.15454800 -1.06427800

H 0.48039800 1.73792600 -1.09745300

C 0.94281700 2.79003000 1.27816400

H 1.53881200 3.29201700 2.05867400

H 0.35794700 3.56863300 0.76040400

H -1.77733900 1.67756100 3.19317200

H -0.77749500 3.12646000 3.46155200

H 0.59083100 1.12832600 2.61598000

H -0.40575300 1.10579000 1.16265100

C -1.92453600 1.50762500 -0.85690100

O -0.82485000 0.97579500 -1.18856100

O -2.93474400 0.65776500 -0.48624400

O -2.21200600 2.71417900 -0.81204300

C 1.33302200 -2.68479600 -0.12965700

C 2.81737400 -2.93494400 -0.43138100

C 1.16157500 -1.18328800 0.28837900

H 0.71566500 -2.89422900 -1.01468800

H 0.98711900 -3.33631100 0.68531300

H 0.50963200 -0.62861400 -0.39882300

H 0.75890700 -1.09317900 1.30379500

C 3.25353700 -1.98974900 -1.56110200

H 4.30609100 -2.15912300 -1.82855000

H 2.64461300 -2.15868000 -2.46039000

C 3.07597400 -0.51397500 -1.06306600

H 4.03868600 0.00721900 -0.98876000

H 2.40748800 0.07563600 -1.70366400

H 2.96898200 -3.98057500 -0.73825300

C 3.39193500 -1.14750100 1.26333400

H 4.32009100 -0.56275300 1.27642000

H 2.91385700 -1.08408800 2.24860100

C 3.64591200 -2.63706000 0.82935800

H 4.71663800 -2.78540700 0.63000200

H 3.35794300 -3.31085200 1.64849100

N 2.48996500 -0.57434400 0.27083700

C -2.65460700 -0.73945500 -0.43402600

C -3.93221200 -1.47361400 -0.07002600

H -1.87030200 -0.93622000 0.31730400

H -2.26255300 -1.08368000 -1.40553500

C -3.72664600 -2.98556100 0.03368300

H -4.31072300 -1.07896300 0.88929300

H -4.70396800 -1.24886500 -0.82709400

C -5.00215400 -3.74034300 0.40422800

H -3.33650800 -3.36628300 -0.92727400

H -2.94163900 -3.19502200 0.78254200

H -4.82471800 -4.82526500 0.47225000

H -5.39496000 -3.40277900 1.37774700

H -5.79342600 -3.57608500 -0.34601000

O -0.23747000 4.51413700 -1.38977800

H -0.98569200 3.89706600 -1.20494900

H 0.50359500 3.89346400 -1.48553900

**[BuOCO_2_...H_2_O...HOCHPr]^.-^**

**Denoted by Int7’**

-1 2

C -0.59744500 2.26040600 0.83348400

C -1.62803700 1.82054200 -0.20521900

H -0.20354000 1.38947300 1.38178200

C -3.77582500 0.47586600 -0.56826600

H -4.86060700 0.62845800 -0.50874200

O -3.42536900 -0.67571800 -1.19289900

H -2.46315700 -0.92732400 -1.01021500

C -2.85674000 1.12622400 0.41841600

H -3.43186700 1.86587000 1.00283100

H -2.48882700 0.37077400 1.14405800

H 0.25865600 2.77254800 0.36459000

H -1.03727400 2.95263700 1.57202600

H -1.96822500 2.68620600 -0.80106200

H -1.15672400 1.11623500 -0.90734600

C -0.61143400 -1.46100300 0.43203400

O -0.95030400 -1.33185600 -0.77805700

O 0.70325600 -1.10843900 0.73986900

O -1.27907100 -1.84587100 1.39579700

C 1.52683000 -0.61703400 -0.30684800

C 2.88311200 -0.25786500 0.27631100

H 1.06301800 0.26710700 -0.77654500

H 1.63209200 -1.37775800 -1.10109000

C 3.85554800 0.27881900 -0.77463600

H 2.74119000 0.49454100 1.07250400

H 3.31250000 -1.15048000 0.76521400

C 5.21971700 0.65285400 -0.19722100

H 3.98494600 -0.47679100 -1.57034400

H 3.40794000 1.16128000 -1.26647400

H 5.89859100 1.03540500 -0.97587100

H 5.12346100 1.43290300 0.57640200

H 5.70538400 -0.21886700 0.27228900

**[HOCO_2_...H_2_O...HOBu]^-^**

**Denoted by Int8’**

-1 1

C -3.55985200 -0.68881900 0.03795100

C -2.09161200 -0.26802700 0.03226700

H -4.14673700 -0.09676300 -0.68469400

C -0.43100900 1.66428500 0.39788800

H -0.38225400 2.70322200 0.76845900

H 0.15748800 1.04833400 1.10333000

O 0.16944300 1.64711800 -0.88658200

H 0.45144800 0.70722500 -1.06633900

C -1.88728300 1.20409600 0.39527200

H -2.31148900 1.39791100 1.39827400

H -2.45447100 1.83957900 -0.30976000

H -3.67946800 -1.75173600 -0.22728600

H -4.01654400 -0.53895200 1.03109100

H -1.51825700 -0.89957700 0.73431000

H -1.64591300 -0.46121900 -0.95616800

C 1.33465200 -1.34597600 -0.05718900

O 0.91582200 -0.86264600 -1.14154600

O 0.86003000 -2.60576300 0.26046700

O 2.11109700 -0.85071200 0.77931500

H 0.25778700 -2.84910600 -0.45868800

O 2.75985800 1.76510700 0.20306600

H 2.61002200 0.81975100 0.43697400

H 1.95900900 1.94314700 -0.32439900

**[HOCO_2_...H_2_O...HOCHPr...H...NC_7_H_13_]^.^**

**Denoted by TS7’**

0 2

C -4.23678000 -1.83724700 -1.17199100

C -2.94103300 -1.23744600 -0.62832800

H -5.11848900 -1.37459900 -0.69809400

C -1.62160200 -0.75403300 1.56643700

H -1.49055700 -1.11060300 2.60265600

H -0.61070300 -1.12606700 1.04469200

O -1.62860000 0.61207400 1.56367500

H -1.61213000 1.04418000 0.62789800

C -2.78441700 -1.46190300 0.87691900

H -2.68758500 -2.54066800 1.08688600

H -3.70108400 -1.12433200 1.39691200

H -4.32305500 -1.68617600 -2.25947600

H -4.28884500 -2.92176700 -0.97867100

H -2.08271800 -1.68855400 -1.15279700

H -2.89931100 -0.16130100 -0.85435900

C -0.36212400 2.38976800 -0.99416700

O -1.38351900 1.69184500 -0.71116400

O -0.07538900 2.49197100 -2.33051500

O 0.42347800 2.96278900 -0.22663800

C 2.86331800 0.29128900 0.42166100

C 3.13139000 -0.80147100 -0.62357600

C 1.48178700 0.00927800 1.08437300

H 3.64943200 0.29973000 1.19018700

H 2.84062700 1.28185900 -0.05474700

H 1.58190900 -0.25705700 2.14429400

H 0.81814700 0.87526100 1.02185800

C 3.04857100 -2.18301700 0.05101300

H 3.24894800 -2.97905600 -0.68028900

H 3.80204300 -2.26402800 0.84765000

C 1.61505300 -2.37417400 0.65643100

H 1.07679900 -3.20566600 0.18462200

H 1.64738600 -2.54289400 1.74008000

H 4.12763400 -0.66310300 -1.06866700

C 0.65992000 -0.92255600 -1.03798600

H 0.15572700 -1.81236300 -1.43374800

H -0.00860200 -0.06497800 -1.16074300

C 2.05032500 -0.70537700 -1.70967700

H 2.21204700 -1.46589300 -2.48704700

H 2.07280600 0.28200200 -2.19282300

N 0.87143900 -1.13689200 0.40094000

H -0.76461500 2.00112400 -2.80450700

O 0.06482600 2.71714600 2.48101800

H 0.18657700 2.89992600 1.52239000

H -0.56527000 1.97801400 2.45160400

**[HOCO_2_...H_2_O...HOCHPr]^.-^**

**Denoted by Int9’**

-1 2

C -2.90546800 -1.73361700 0.48687400

C -1.78260500 -0.71330100 0.31155500

H -3.48344400 -1.85443800 -0.44522100

C -1.28282200 1.77231400 -0.15302600

H -1.62986000 2.80296900 -0.00327800

O -0.20204600 1.70850800 -0.97581600

H 0.24601600 0.79378200 -1.00991900

C -2.29933000 0.67376600 -0.10121600

H -3.08919500 0.99196100 0.60251400

H -2.80732500 0.56949600 -1.08886600

H -2.51198700 -2.72473700 0.76460400

H -3.61386900 -1.42255400 1.27359800

H -1.20432600 -0.61639200 1.24649200

H -1.06701900 -1.06938800 -0.44406300

C 1.66197300 -0.95594500 -0.09725100

O 1.11116400 -0.49042700 -1.13493300

O 2.14060300 -2.24322100 -0.21258400

O 1.82562500 -0.42204100 1.01118000

H 1.92935500 -2.52874300 -1.11436700

O 1.85132100 2.32943300 0.86118400

H 1.85920100 1.35737200 1.01198000

H 1.20863900 2.38334400 0.13267300

**6. References**

1. Hamilton, D. S.; Nicewicz, D. A. Direct Catalytic Anti-Markovnikov Hydroetherification of Alkenols. *J. Am. Chem. Soc.* **2012**, *134*, 18577-18580.
2. Ge, L.; Zhang, C.; Pan, C.; Wang, D.-X.; Liu, D.-Y.; Li, Z.-Q.; Shen, P.; Tian, L.; Feng, C. Photoredox-catalyzed C–C bond cleavage of cyclopropanes for the formation of C(sp^3^)–heteroatom bonds. *Nat. Commun.* **2022**, *13*, 5938.
3. Deziel, R.; Malenfant, E. Asymmetric Ring Closure Reactions Mediated by a Chiral *C_2_* Symmetrical Organoselenium Reagent. *J. Org. Chem.* **1995**, *60*, 4660-4662.
4. Jeffrey, J. L.; Terrett, J. A.; MacMillan, D. W. C. O–H hydrogen bonding promotes H-atom transfer from α C–H bonds for C-alkylation of alcohols. *Science* **2015**, *349*, 1532-1536.
5. Lei, H.; Conway, J. H., Jr.; Cook, C. C.; Rovis, T. Ligand Controlled Ir-Catalyzed Regiodivergent Oxyamination of Unactivated Alkenes. *J. Am. Chem. Soc.* **2019**, *141*, 11864-11869.
6. Grayson, D. H.; McCarthy, Ú.; Roycroft, E. D. Intramolecular acylative ring-switching reactions of 3-(tetrahydro-2′-furyl)propanoic acid derivatives to give butanolides: mechanism and scope. *Org. Biomol. Chem.* **2003**, *1*, 1930-1937.
7. Näf, R.; Jaquier, A. New lactones in liquorice (*Glycyrrhiza glabra* L.). *Flavour. Frag. J.* **2006**, *21*, 193-197.
8. Maji, R.; Ghosh, S.; Grossmann, O.; Zhang, P.; Leutzsch, M.; Tsuji, N.; List, B. A Catalytic Asymmetric Hydrolactonization. *J. Am. Chem. Soc.* **2023**, *145*, 8788-8793.
9. Ketterer, C.; Wünsch, B. Lipase-Catalyzed Kinetic Resolution of 2-Phenylethanol Derivatives and Chiral Oxa-Pictet–Spengler Reaction as the Key Steps in the Synthesis of Enantiomerically Pure Tricyclic Amines. *Eur. J. Org. Chem.* **2012**, *2012*, 2428-2444.
10. Saito, H.; Kanetake, T.; Osaka, K.; Maeda, K.; Morita, T.; Yoshimi, Y. A strategy for generating alkyl radicals from aliphatic esters and lactones via sequential hydrolysis and photoinduced decarboxylation. *Tetrahedron Lett.* **2015**, *56*, 1645-1648.
11. Maji, A.; Reddi, Y.; Sunoj, R. B.; Maiti, D. Mechanistic Insights on Orthogonal Selectivity in Heterocycle Synthesis. *ACS Catal.* **2018**, *8*, 10111-10118.
12. Pérez, E. R.; Santos, R. H. A.; Gambardella, M. T. P.; de Macedo, L. G. M.; Rodrigues-Filho, U. P.; Launay, J.-C.; Franco, D. W. Activation of Carbon Dioxide by Bicyclic Amidines. *J. Org. Chem.* **2004**, *69*, 8005-8011.
13. Lv, M.; Wang, P.; Yuan, D.; Yao, Y. Conversion of Carbon Dioxide into Oxazolidinones Mediated by Quaternary Ammonium Salts and DBU. *ChemCatChem* **2017**, *9*, 4451-4455.
14. Archer, G.; Meyrelles, R.; Eder, I.; Kovács, N.; Maryasin, B.; Médebielle, M.; Merad, J. Photoredox-Catalyzed α−C−H Monoalkylation of Symmetric Polyols in the Presence of CO_2_. *Angew. Chem. Int. Ed.* **2024**, *63*, e202315329.
15. (a) Frisch, M.; Trucks, G.; Schlegel, H. B.; Scuseria, G.; Robb, M.; Cheeseman, J.; Scalmani, G.; Barone, V.; Petersson, G.; Nakatsuji, H., Gaussian 16. Gaussian, Inc. Wallingford, CT: **2016**. (b) Lee, C.; Yang, W.; Parr, R. G., Development of the Colle-Salvetti correlation-energy formula into a functional of the electron density. *Phys. Rev. B.* **1988,** *37*, 785. (c) Becke, A. D., Density-functional thermochemistry. I. The effect of the exchange-only gradient correction. *J. Chem. Phys.* **1992,** *96*, 2155-2160. (d) Goerigk, L.; Grimme, S., A thorough benchmark of density functional methods for general main group thermochemistry, kinetics, and noncovalent interactions. *Phys. Chem. Chem. Phys.* **2011,** *13*, 6670-6688. (e) Weigend, F.; Ahlrichs, R., Balanced basis sets of split valence, triple zeta valence and quadruple zeta valence quality for H to Rn: Design and assessment of accuracy. *Phys. Chem. Chem. Phys.* **2005,** *7*, 3297-3305. (f) Grimme, S.; Antony, J.; Ehrlich, S.; Krieg, H., A consistent and accurate ab initio parametrization of density functional dispersion correction (DFT-D) for the 94 elements H-Pu. *J. Chem. Phys.* **2010,** *132*. (g) Liotard, D. A.; Hawkins, G. D.; Lynch, G. C.; Cramer, C. J.; Truhlar, D. G., Improved methods for semiempirical solvation models. *J. Comput. Chem.* **1995**, *16*, 422-440. (h) A. V. Marenich, C. J. Cramer and D. G. Truhlar, Universal Solvation Model Based on Solute Electron Density and on a Continuum Model of the Solvent Defined by the Bulk Dielectric Constant and Atomic Surface Tensions, *J. Phys. Chem. B.* **2009**, *113*, 6378-6396. (i) Grimme, S.; Ehrlich, S.; Goerigk, L., Effect of the damping function in dispersion corrected density functional theory. *J. Comput. Chem.* **2011,** *32*, 1456-1465.
16. (a) Neese, F. The ORCA Program System. Wiley Interdiscip. Rev.: *Comput. Mol. Sci.* **2012,** *2*, 73–78. (b) Neese, F. Software update: The ORCA program system—Version 5.0, *WIREs Comput. Mol. Sci.* **2022,** *12*, e1606; (c) Neese, F. The SHARK integral generation and digestion system, *J. Comput. Chem.* **2023,** *44*, 381-396; (d) Goerigk, L.; Grimme, S. Efficient and Accurate Double-Hybrid-Meta-GGA Density Functionals Evaluation with the Extended GMTKN30 Database for General Main Group Thermochemistry, Kinetics, and Noncovalent Interactions. *J. Chem. Theory Comput.* **2011,** *7*, 291-309; (e) Weigend, F., Accurate Coulomb-fitting basis sets for H to Rn, *Phys. Chem. Chem. Phys.* **2006,** *8*, 1057-1065; (f) Hellweg, A.; Hättig, C.; Höfener, S. and Klopper, W. Optimized accurate auxiliary basis sets for RI-MP2 and RI-CC2 calculations for the atoms Rb to Rn, *Theor. Chem. Acc.* **2007,** *117*, 587-597; (g) Caldeweyher, E.; Ehlert, S.; Hansen, A.; Neugebauer, H.; Spicher S.; Bannwarth,C. and Grimme, S. A generally applicable atomic-charge dependent London dispersion correction. J. Chem. Phys. **2019**; *150,* 154122.

**7. Copies of NMR spectra for products**


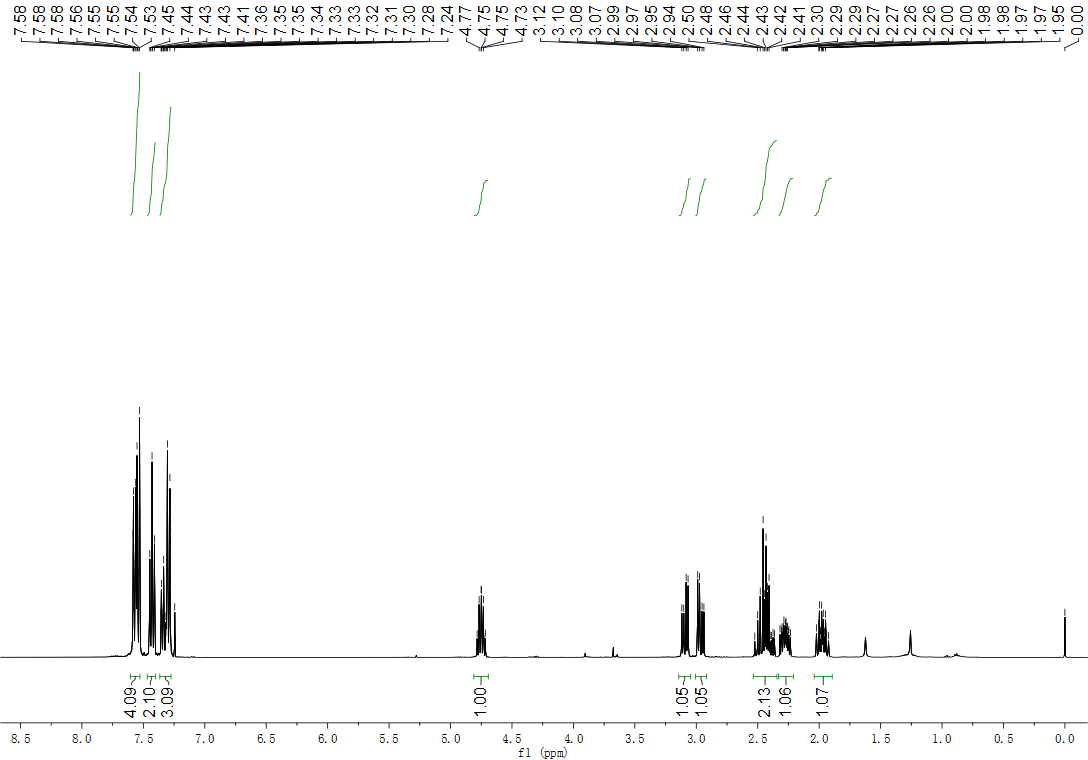


^1^H NMR (400 MHz, CDCl_3_) spectrum of **3b**

**
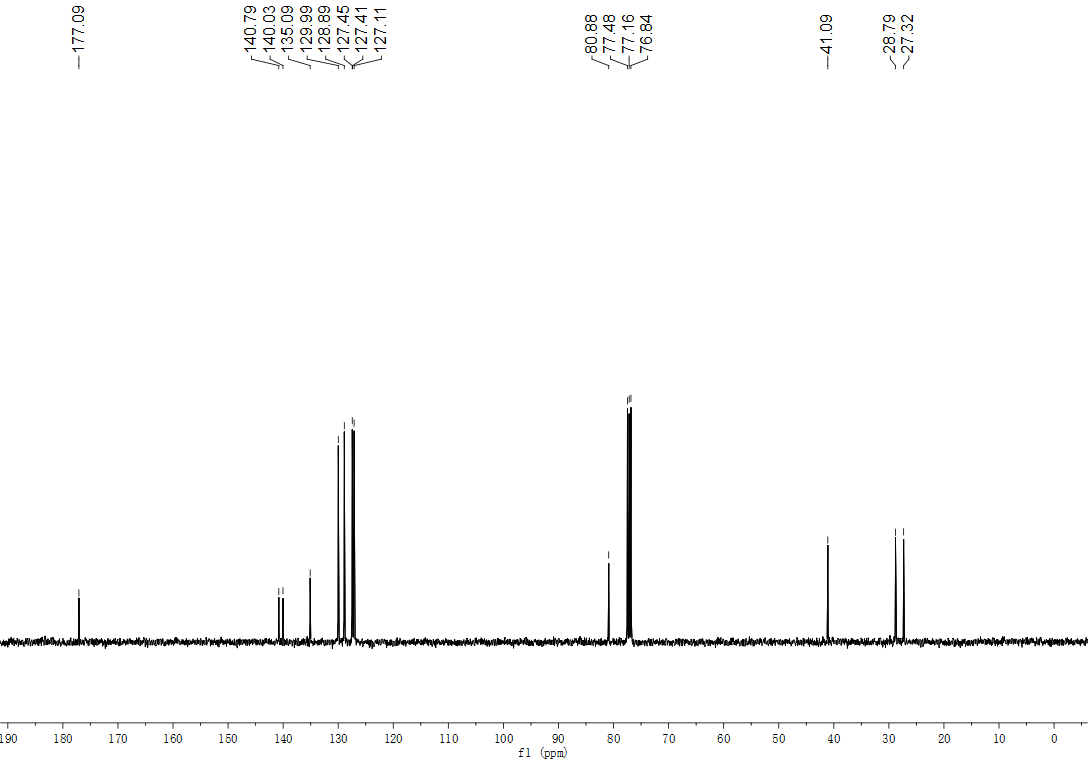
**

^13^C{^1^H} NMR (101 MHz, CDCl_3_) spectrum of **3b**

**
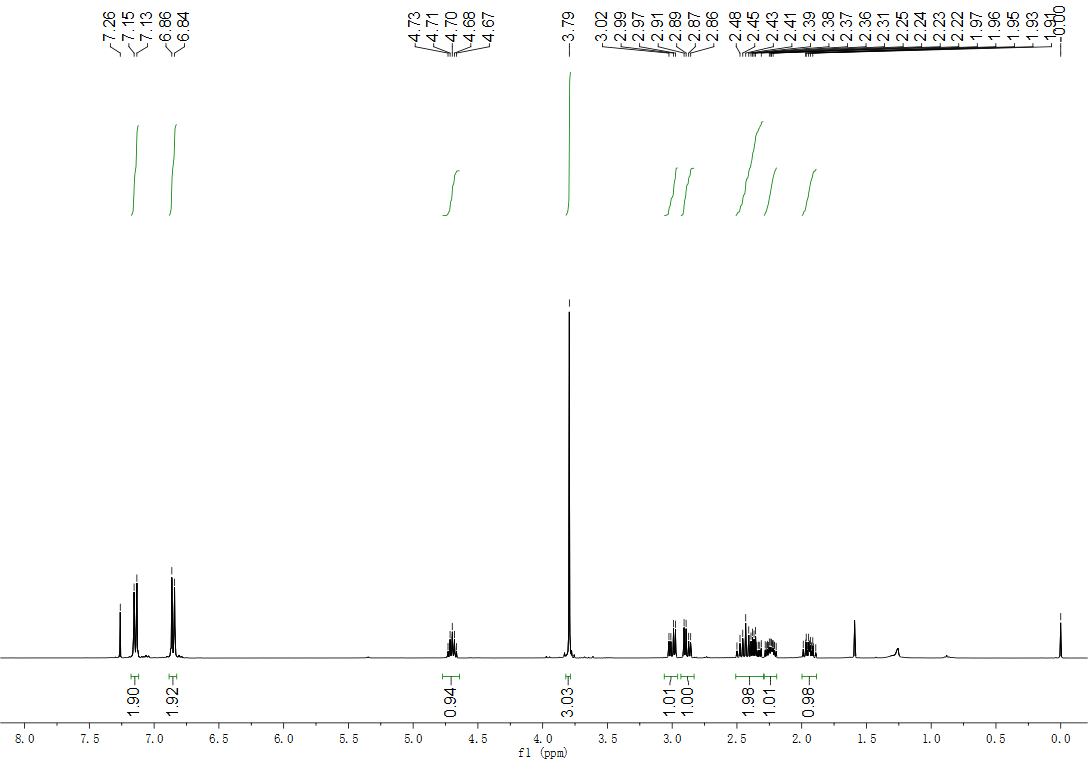
**

^1^H NMR (400 MHz, CDCl_3_) spectrum of **3c**

^
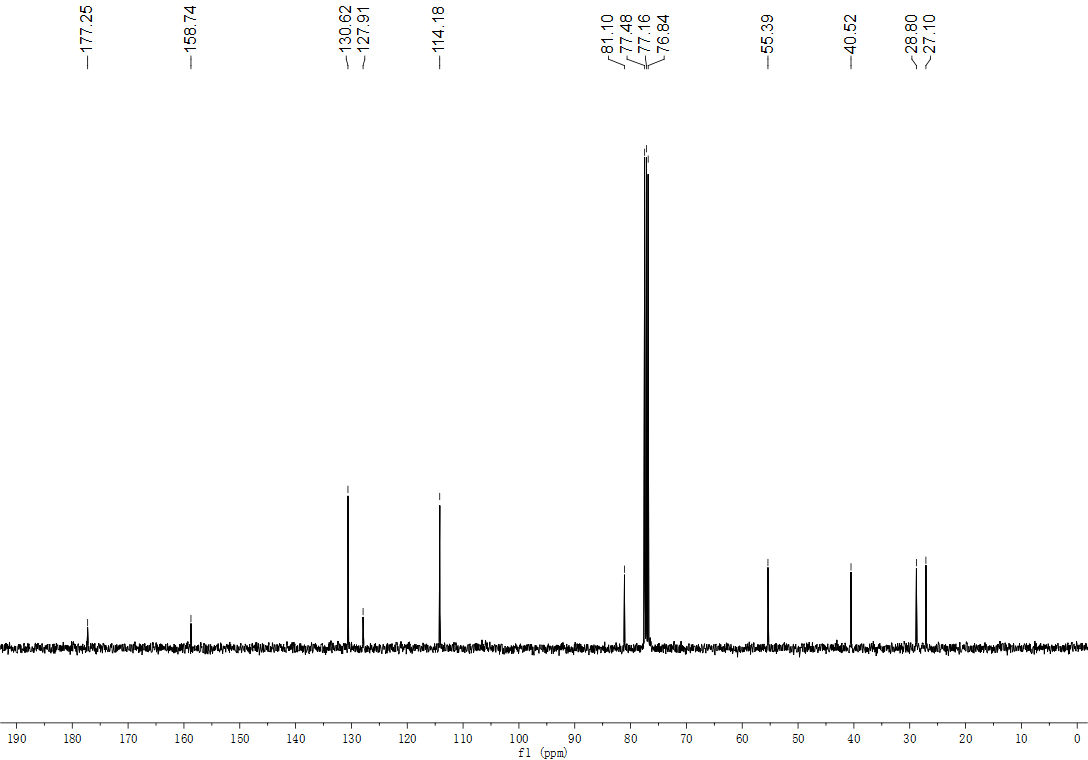
13^C{^1^H} NMR (101 MHz, CDCl_3_) spectrum of **3c**

**
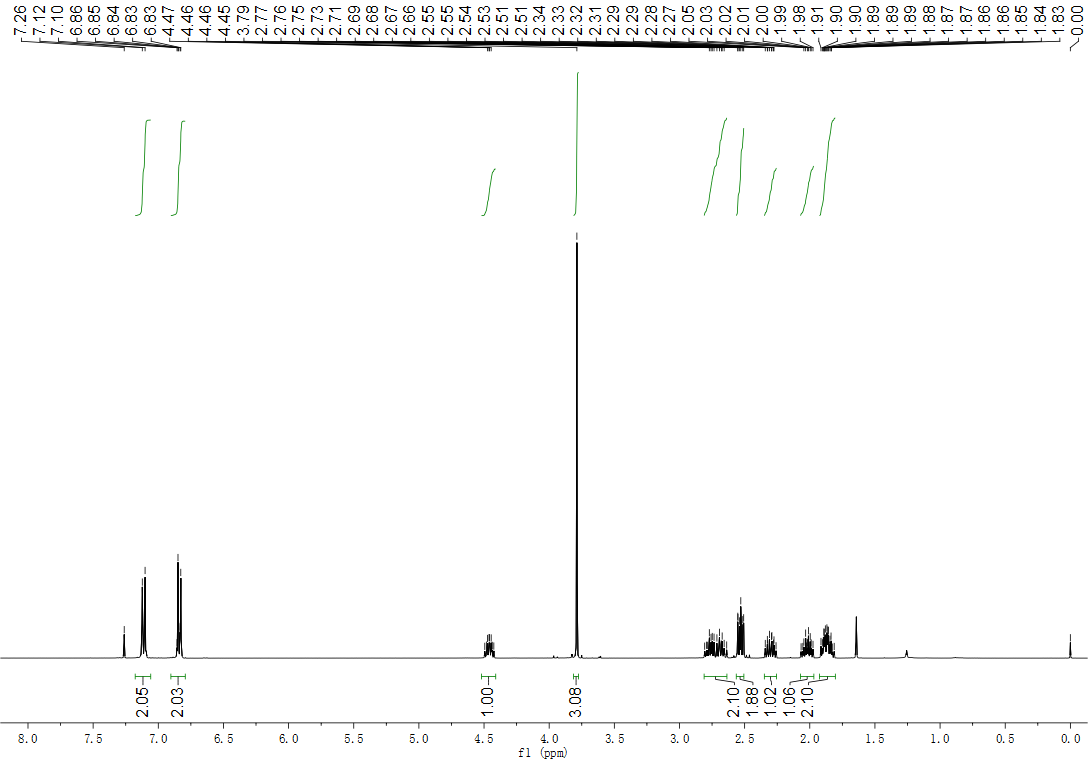
**

^1^H NMR (400 MHz, CDCl_3_) spectrum of **3d**

**
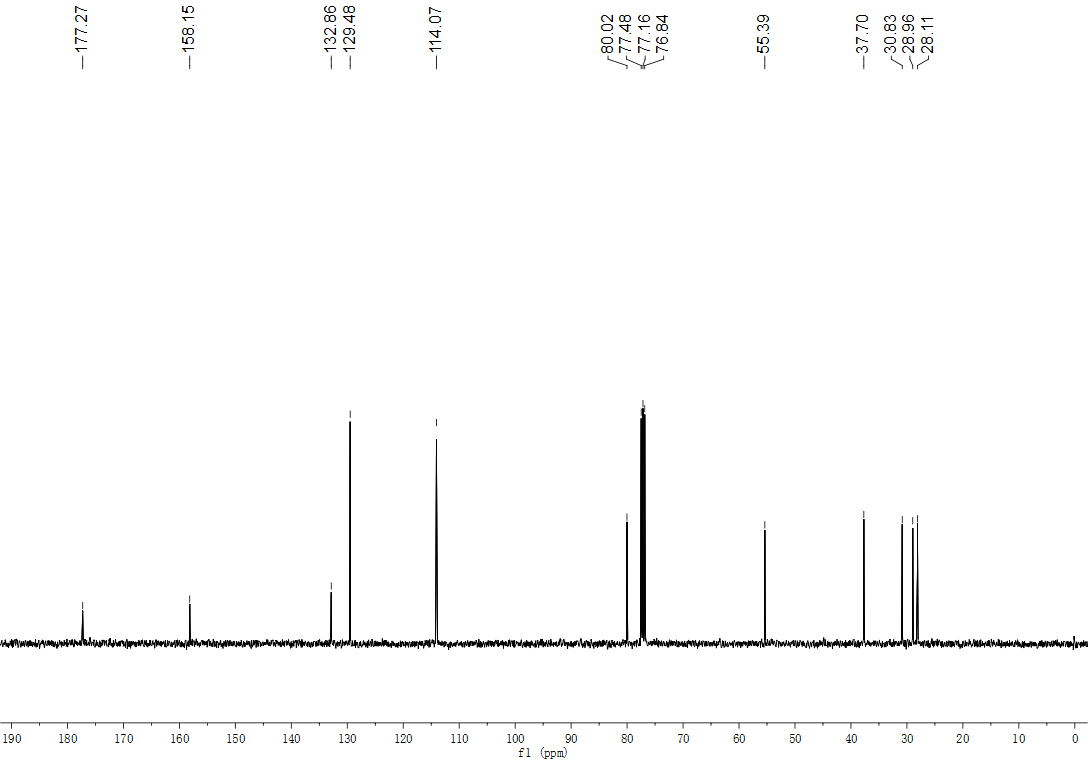
**

^13^C{^1^H} NMR (101 MHz, CDCl_3_) spectrum of **3d**

**
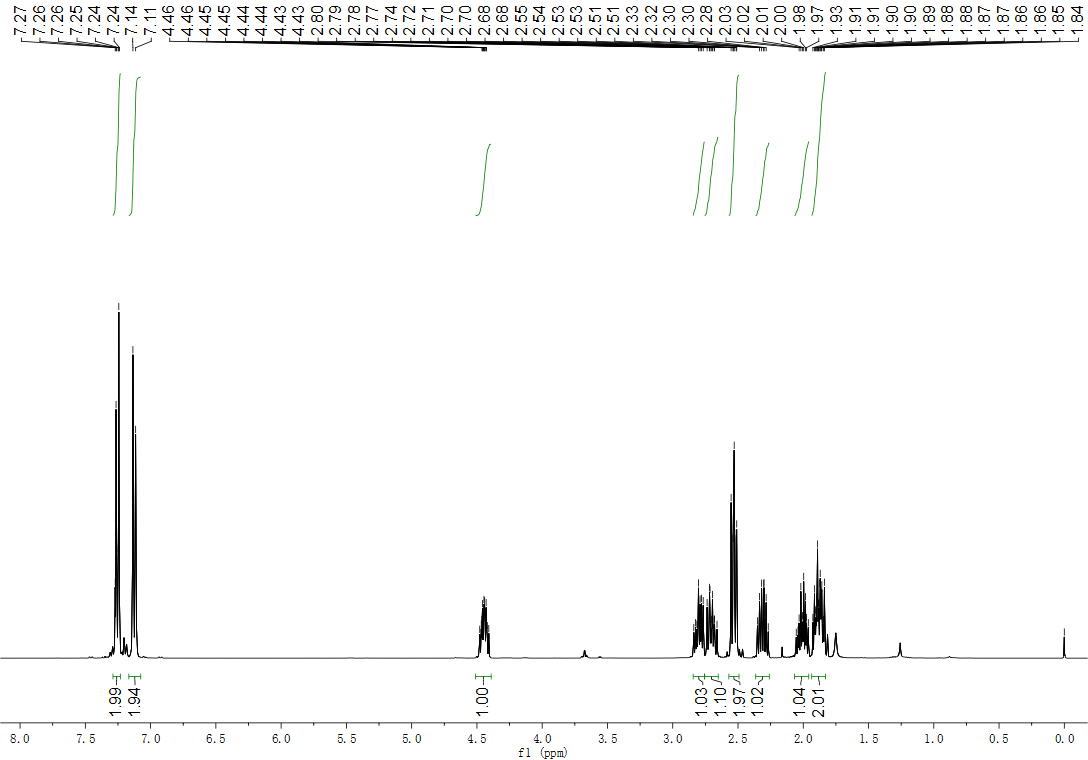
**

^1^H NMR (400 MHz, CDCl_3_) spectrum of **3e**

^
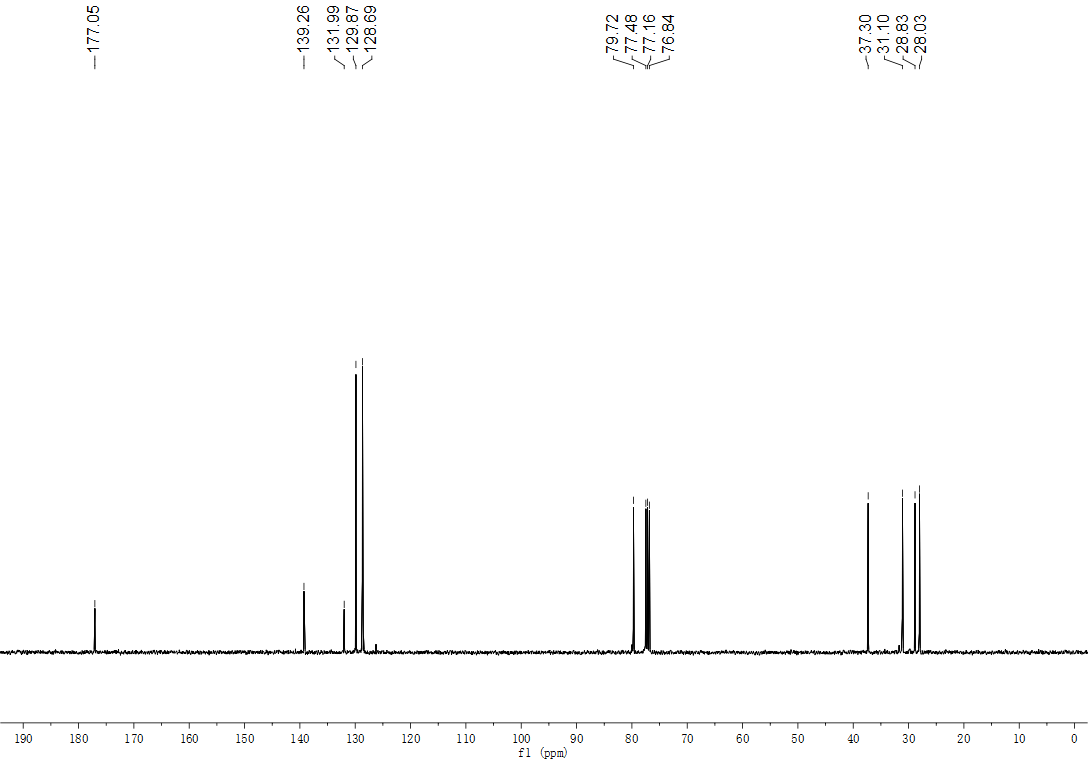
^

^13^C{^1^H} NMR (101 MHz, CDCl_3_) spectrum of **3e**

**
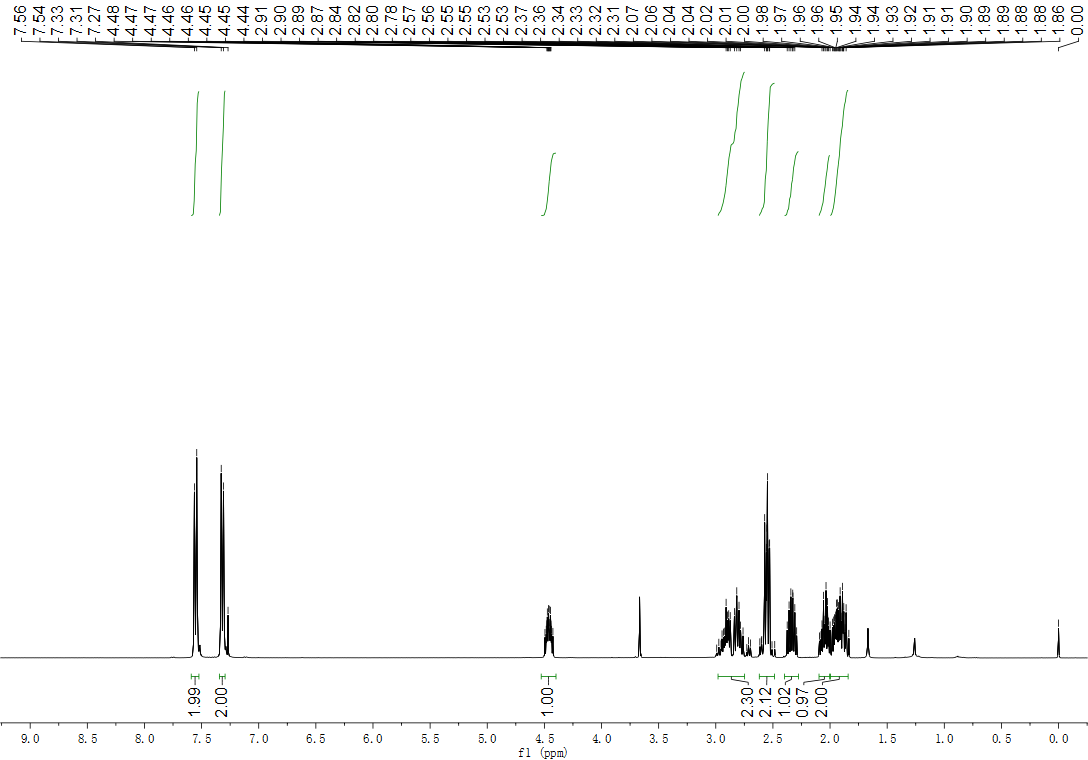
**

^1^H NMR (400 MHz, CDCl_3_) spectrum of **3f**

^
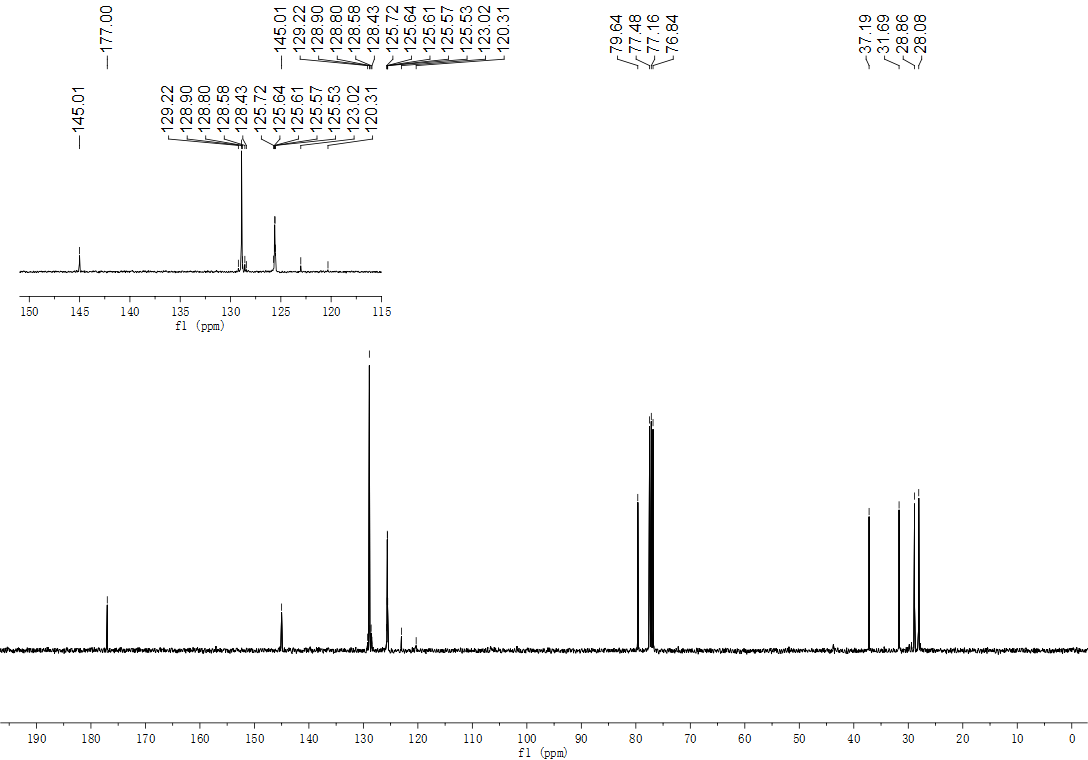
^

^13^C{^1^H} NMR (101 MHz, CDCl_3_) spectrum of **3f**

^
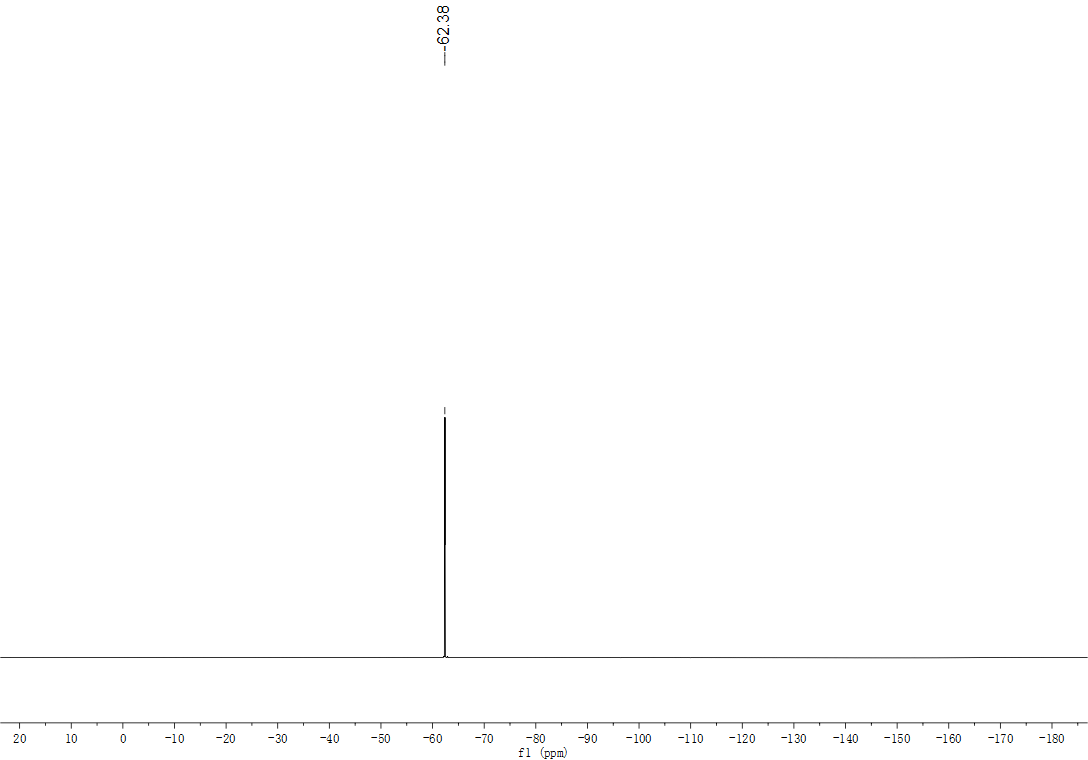
^

^19^F NMR (376 MHz, CDCl_3_) spectrum of **3f**

**
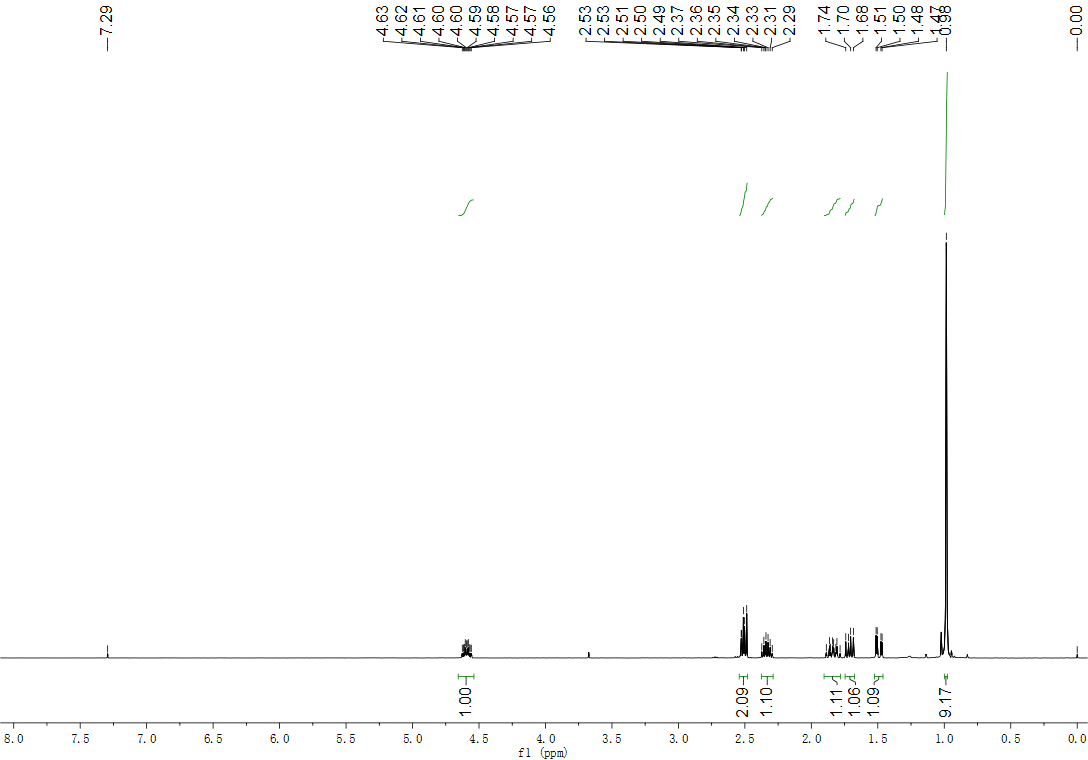
**

^1^H NMR (400 MHz, CDCl_3_) spectrum of **3g**

^
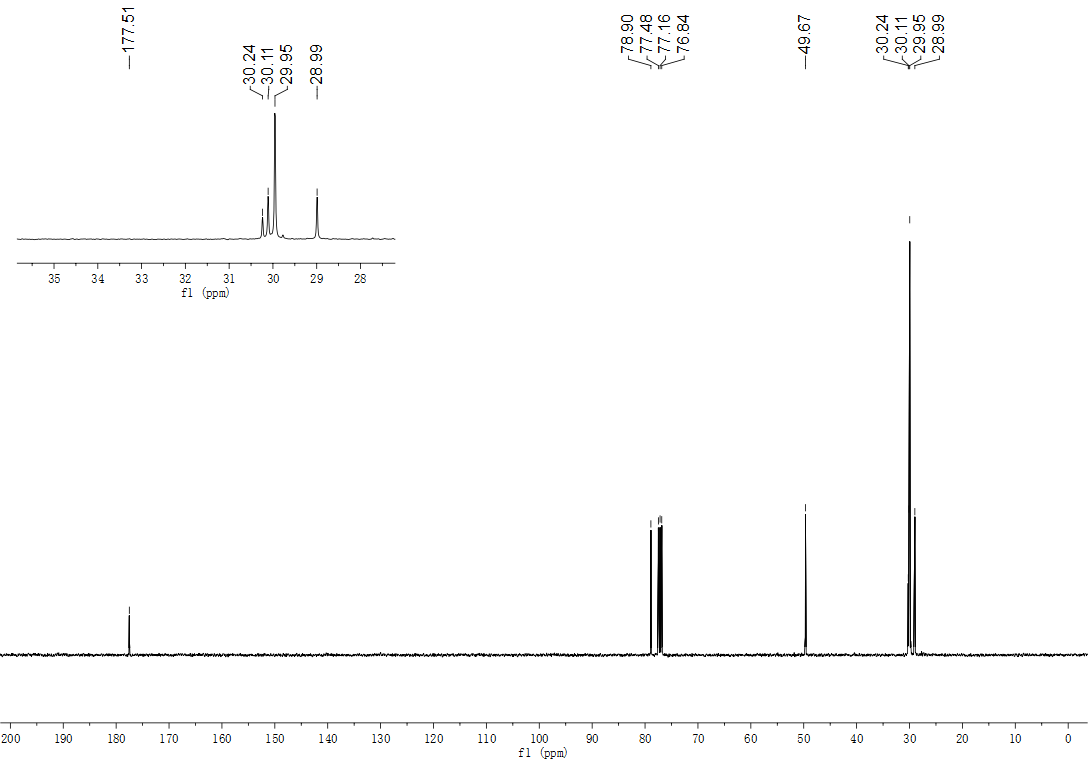
^

^13^C{^1^H} NMR (101 MHz, CDCl_3_) spectrum of **3g**

**
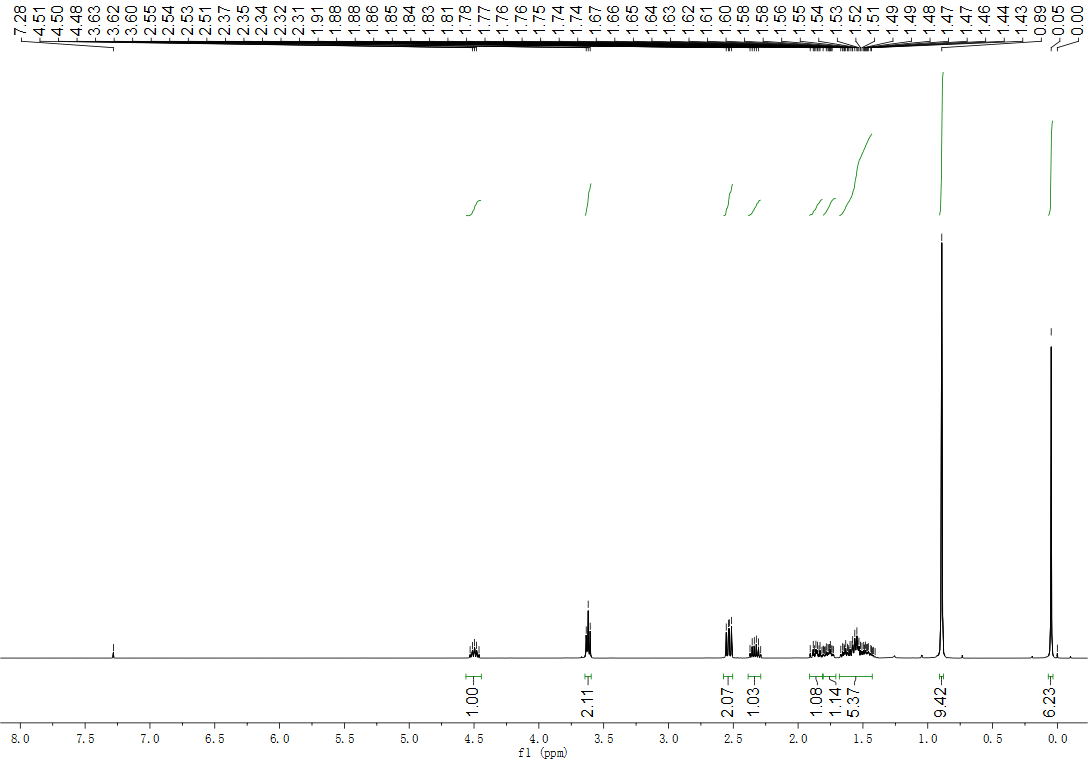
**

^1^H NMR (400 MHz, CDCl_3_) spectrum of **3h**

^
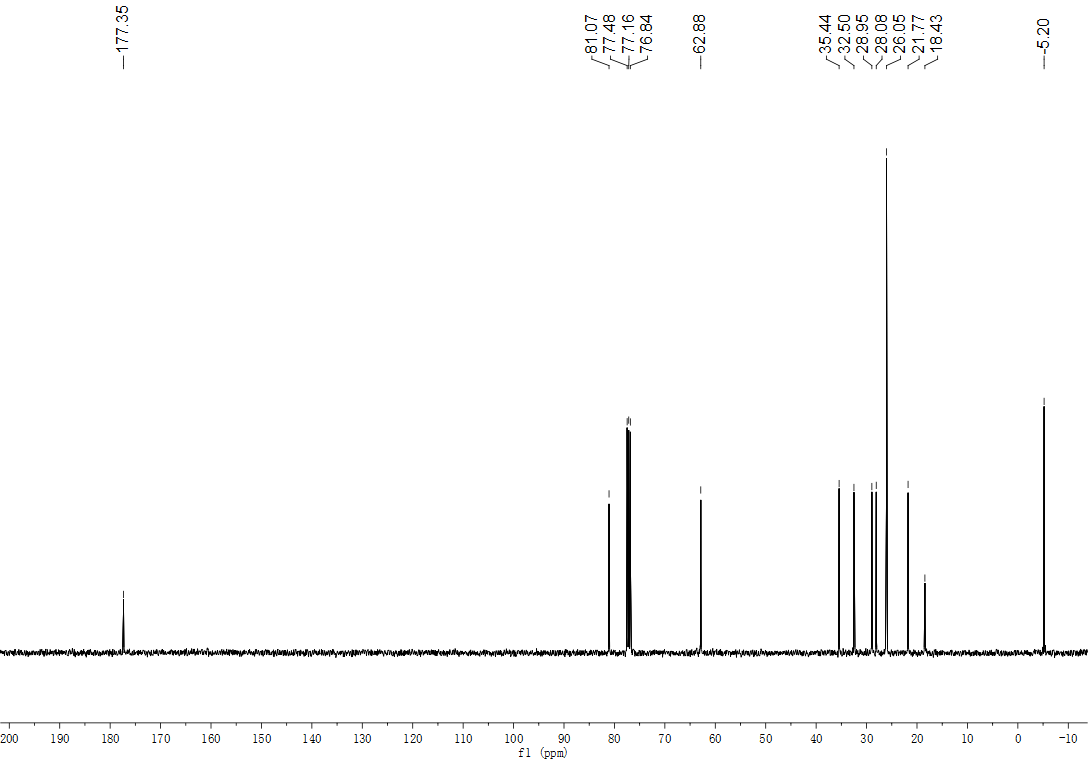
^

^13^C{^1^H} NMR (101 MHz, CDCl_3_) spectrum of **3h**

**
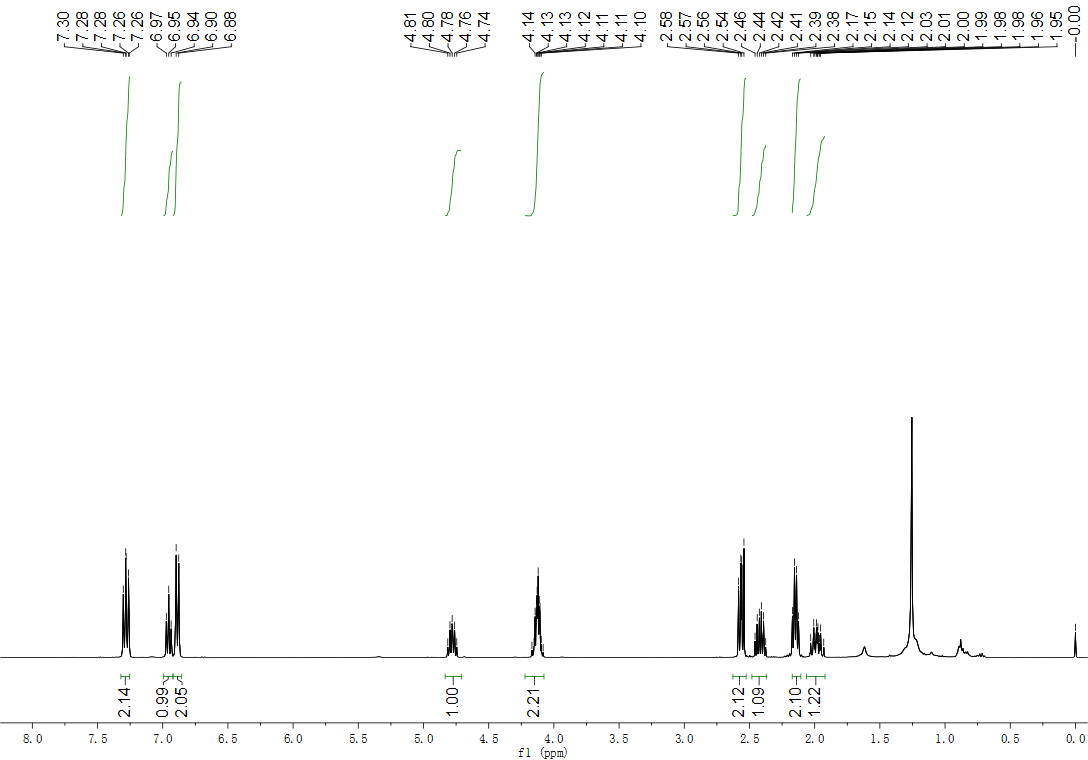
**

^1^H NMR (400 MHz, CDCl_3_) spectrum of **3i**

^
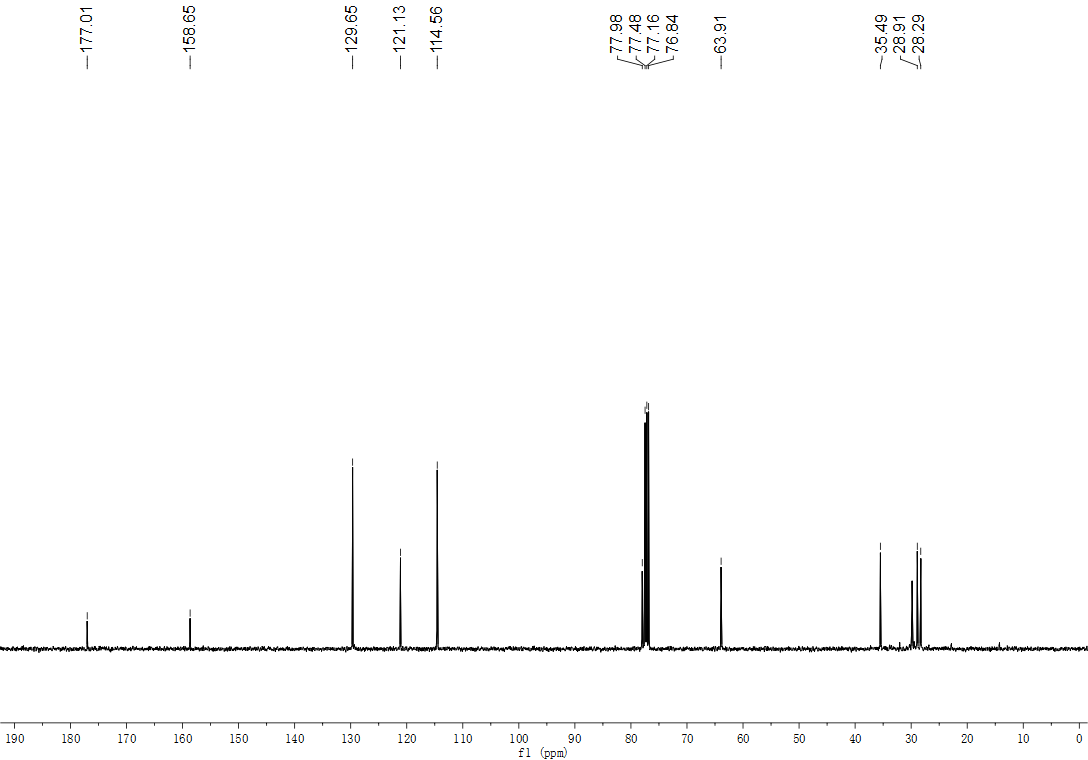
^

^13^C{^1^H} NMR (101 MHz, CDCl_3_) spectrum of **3i**

**
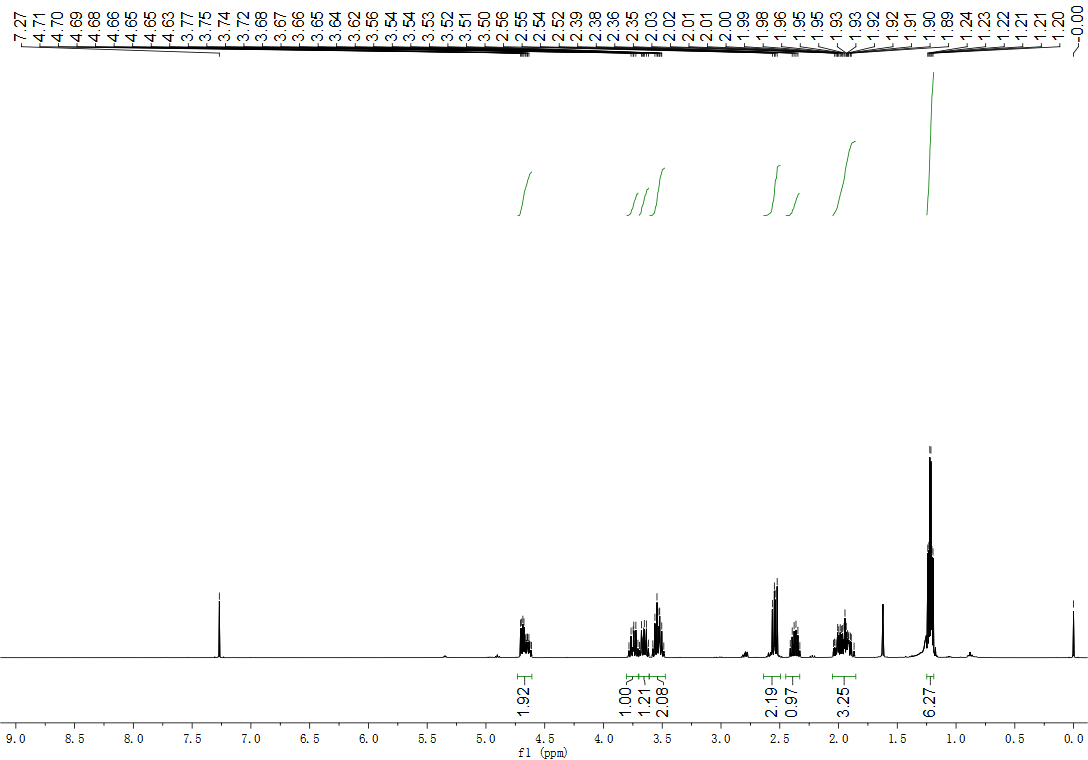
**

^1^H NMR (400 MHz, CDCl_3_) spectrum of **3j**

^
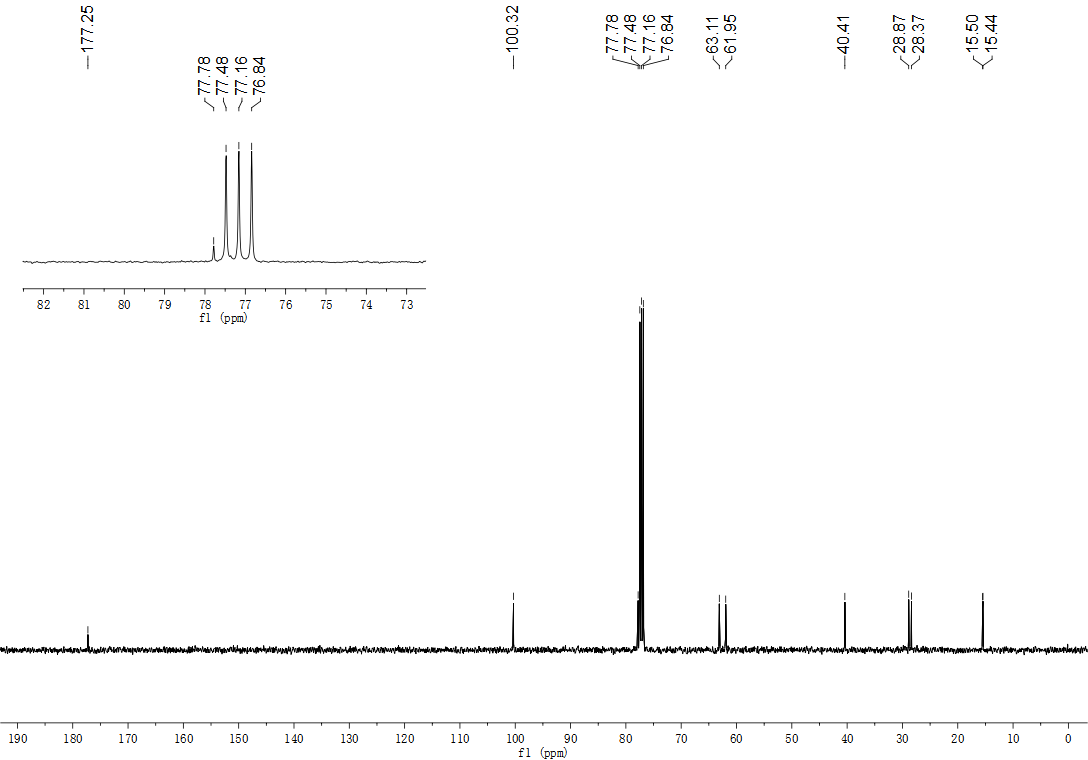
^

^13^C{^1^H} NMR (101 MHz, CDCl_3_) spectrum of **3j**

**
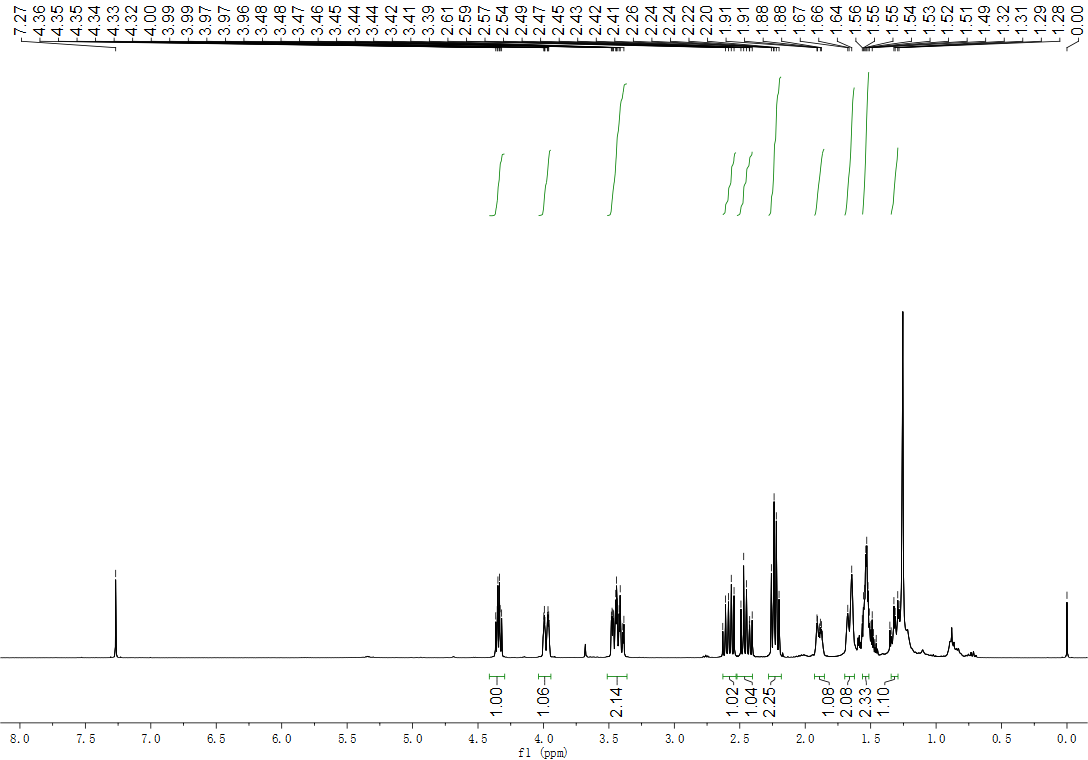
**

^1^H NMR (400 MHz, CDCl_3_) spectrum of **3k**

^
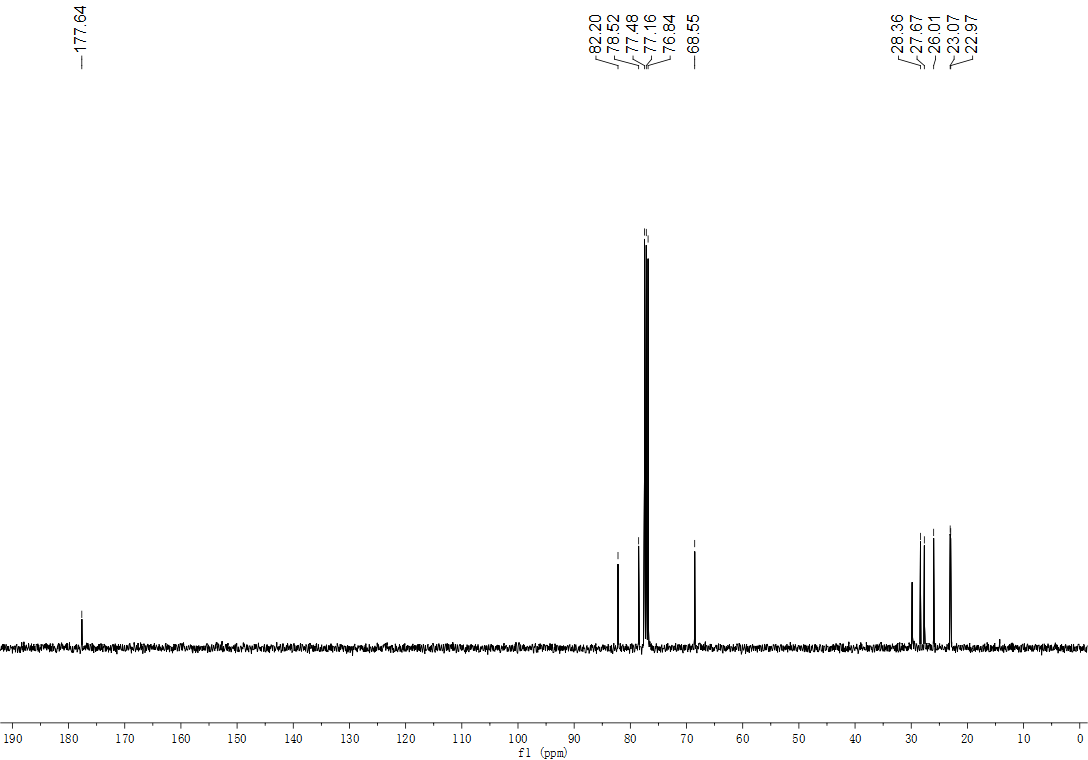
^

^13^C{^1^H} NMR (101 MHz, CDCl_3_) spectrum of **3k**

**
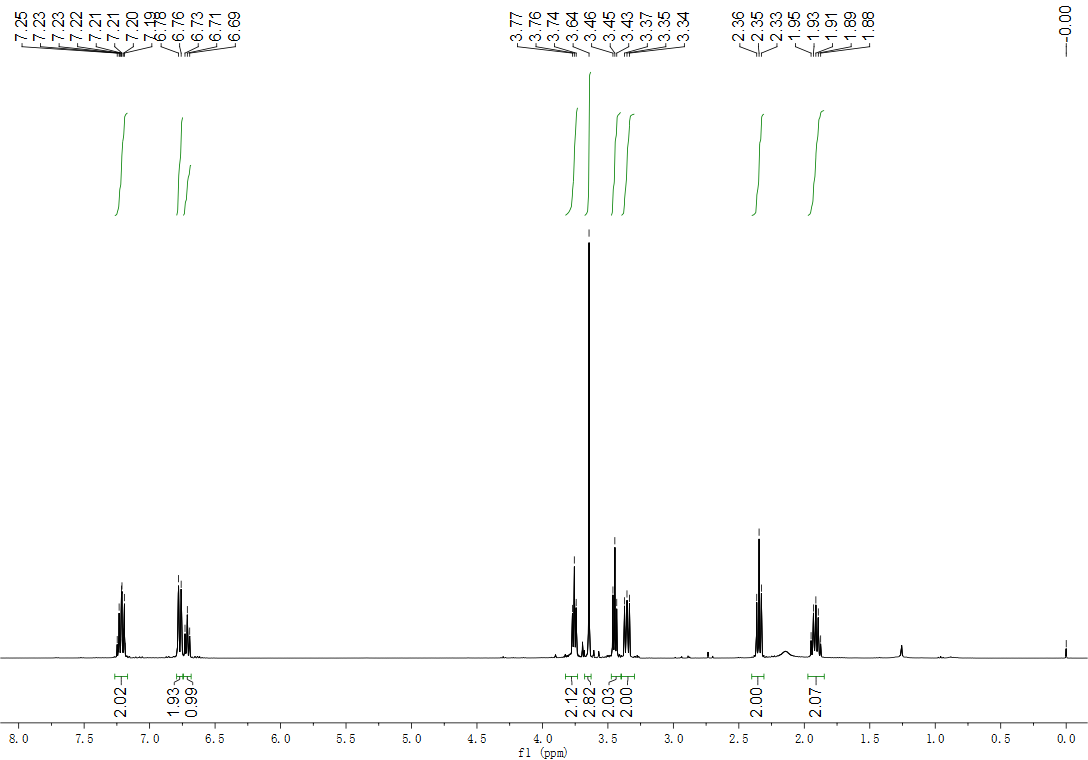
**

^1^H NMR (400 MHz, CDCl_3_) spectrum of **3l**

^
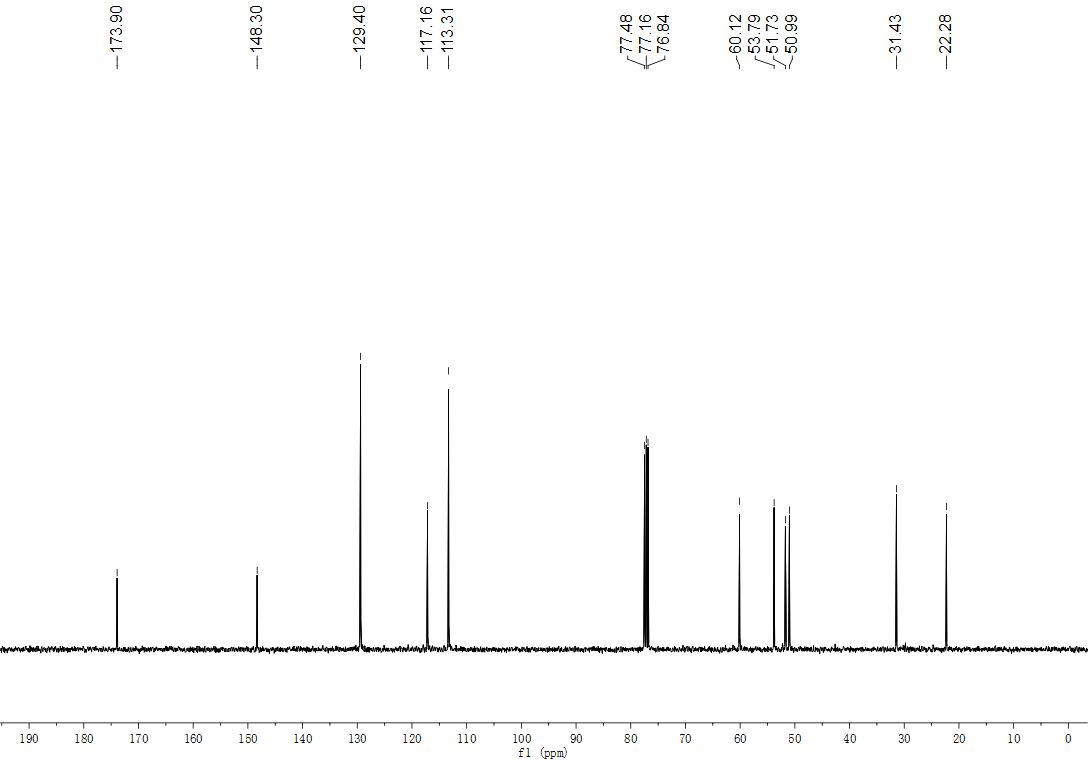
^

^13^C{^1^H} NMR (101 MHz, CDCl_3_) spectrum of **3l**

**
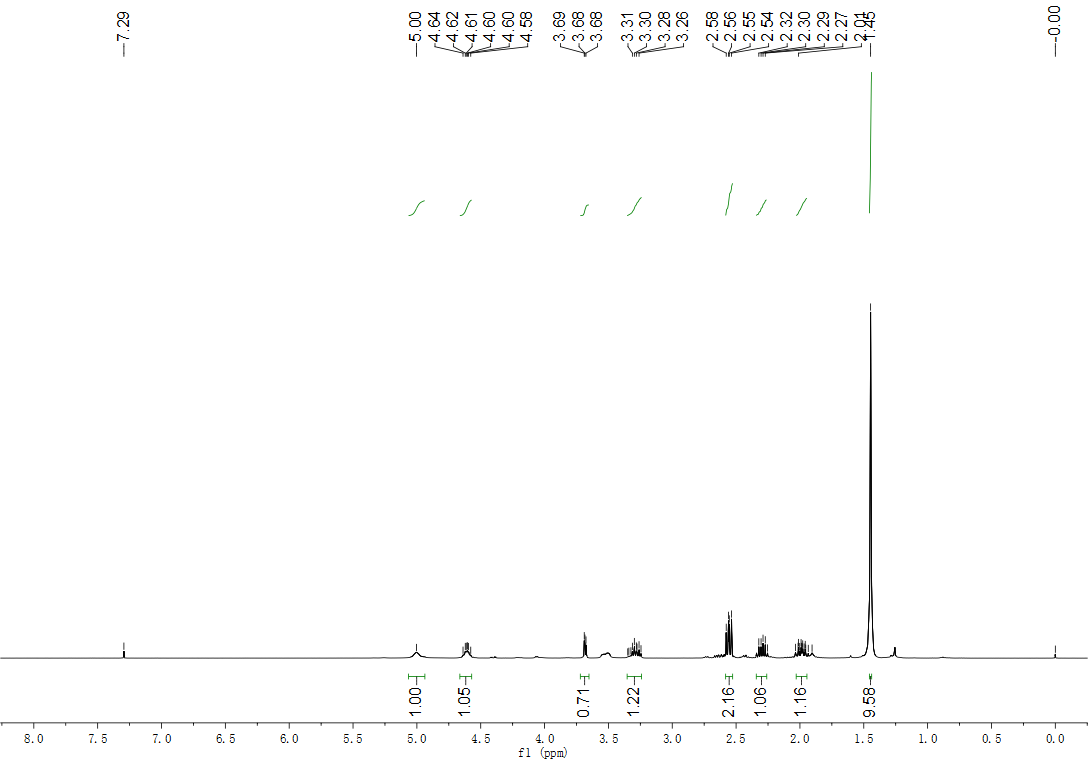
**

^1^H NMR (400 MHz, CDCl_3_) spectrum of **3m**

^
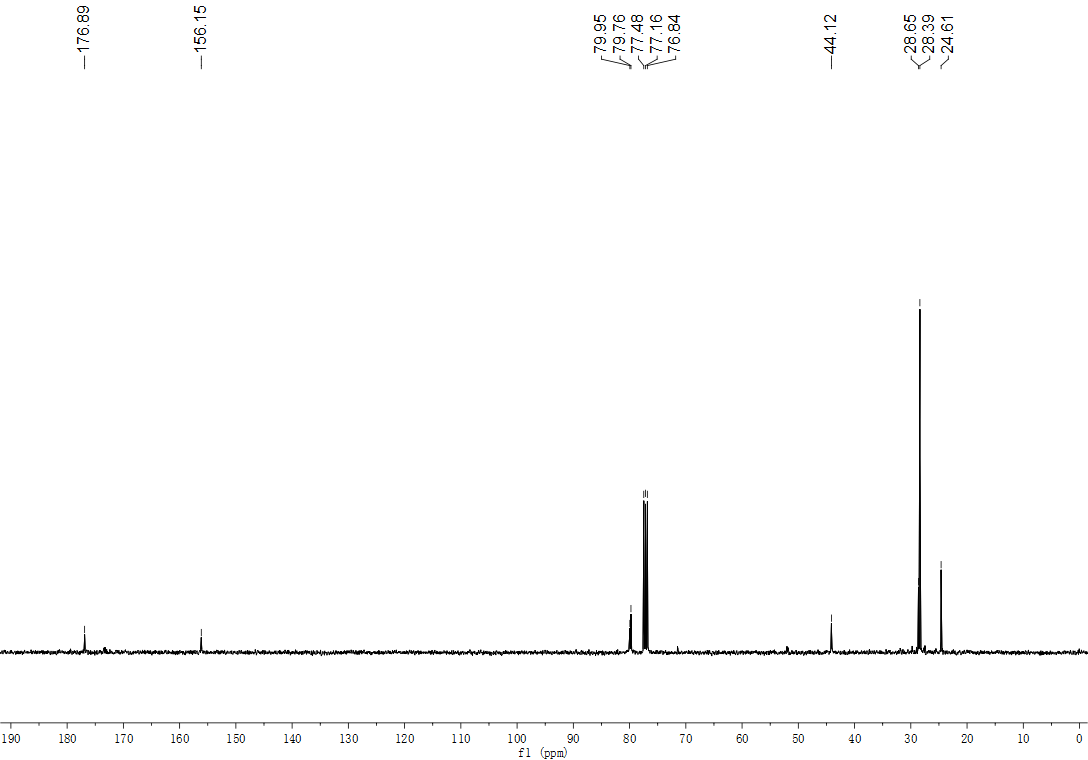
^

^13^C{^1^H} NMR (101 MHz, CDCl_3_) spectrum of **3m**

**
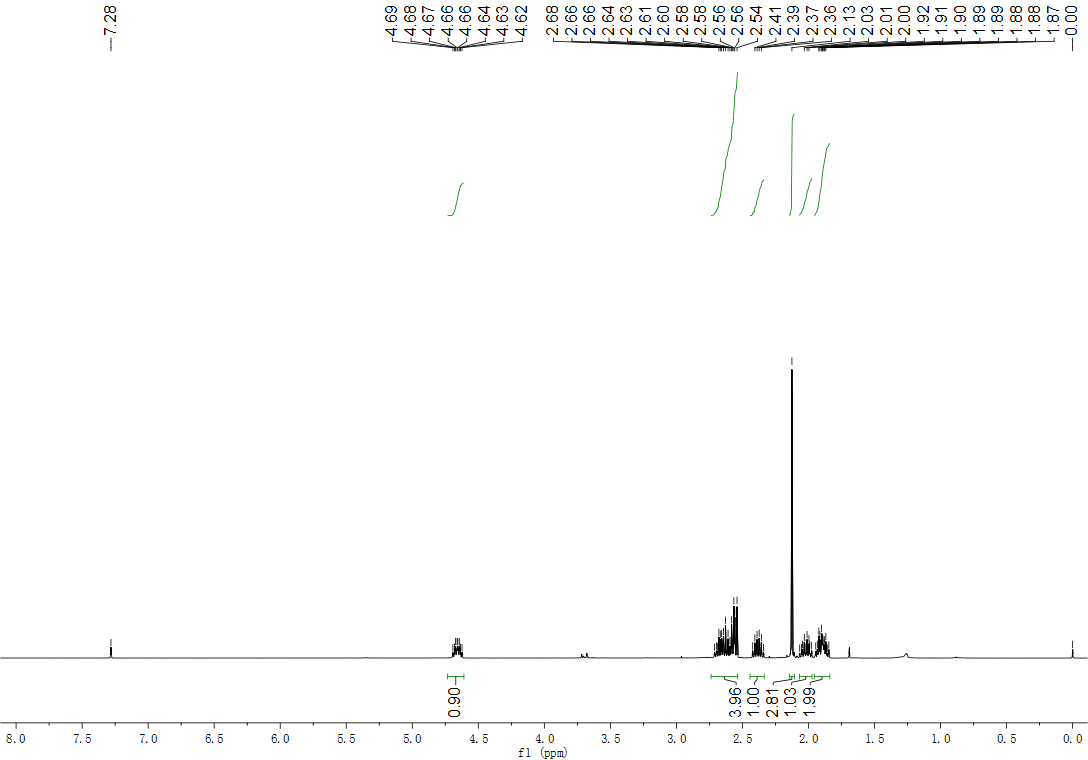
**

^1^H NMR (400 MHz, CDCl_3_) spectrum of **3n**

^
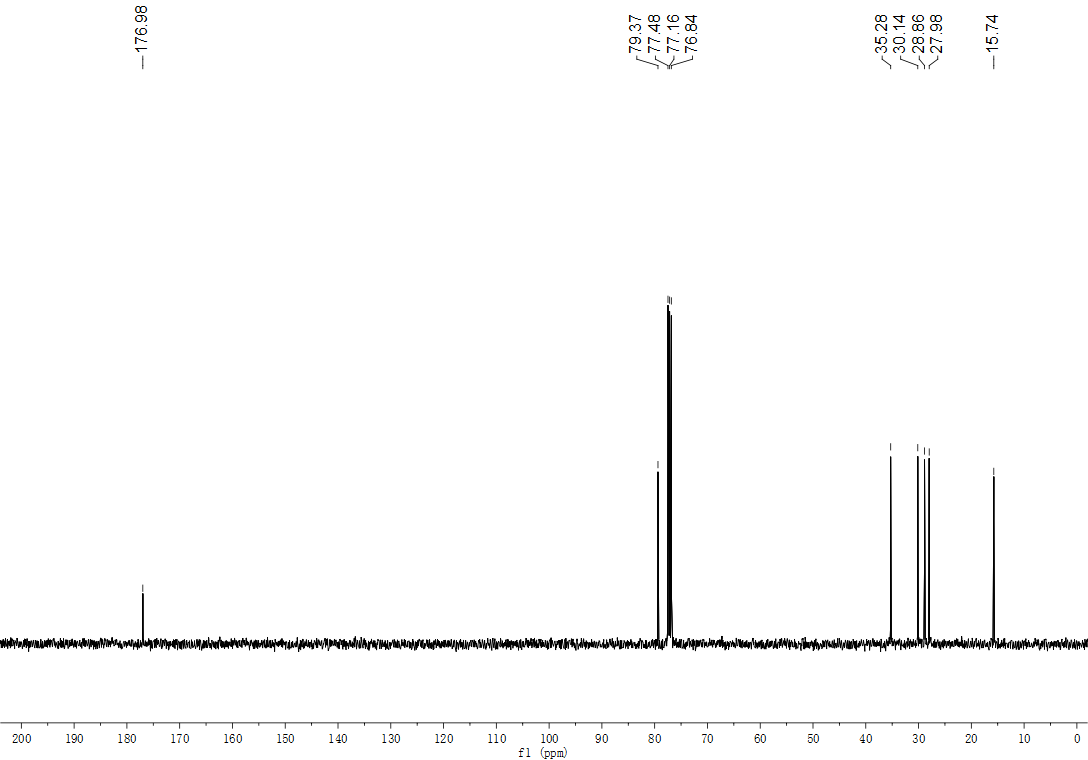
^

^13^C{^1^H} NMR (101 MHz, CDCl_3_) spectrum of **3n**

**
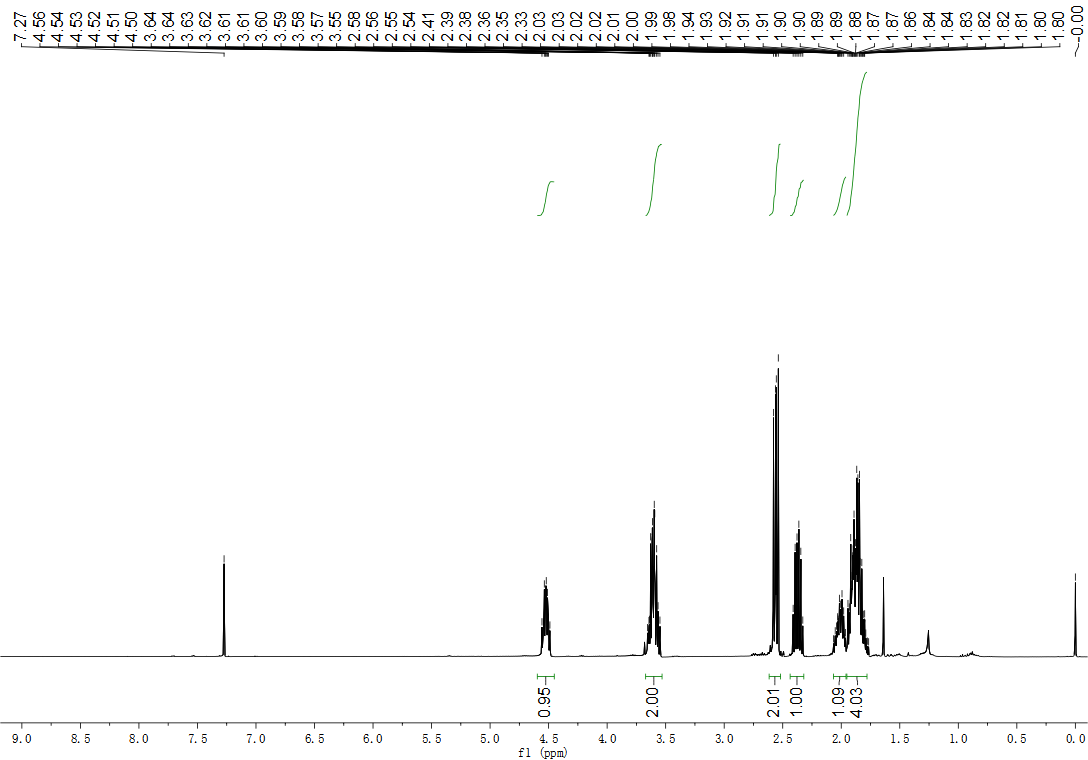
**

^1^H NMR (400 MHz, CDCl_3_) spectrum of **3o**

^
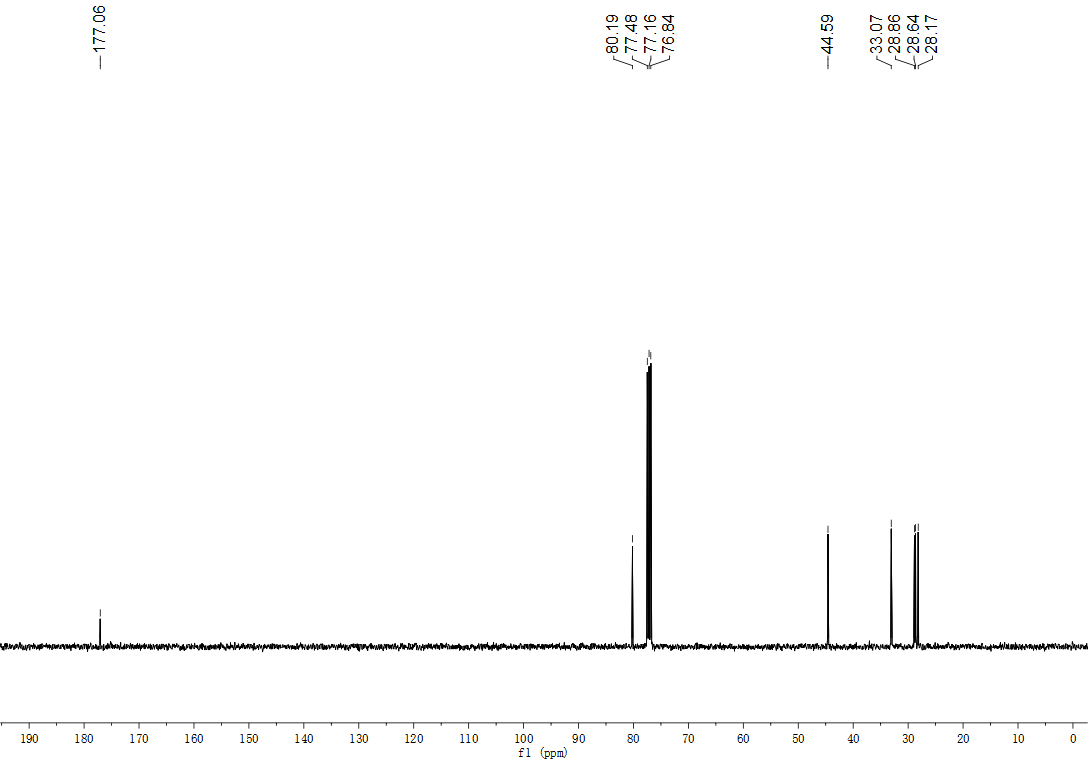
^

^13^C{^1^H} NMR (101 MHz, CDCl_3_) spectrum of **3o**

**
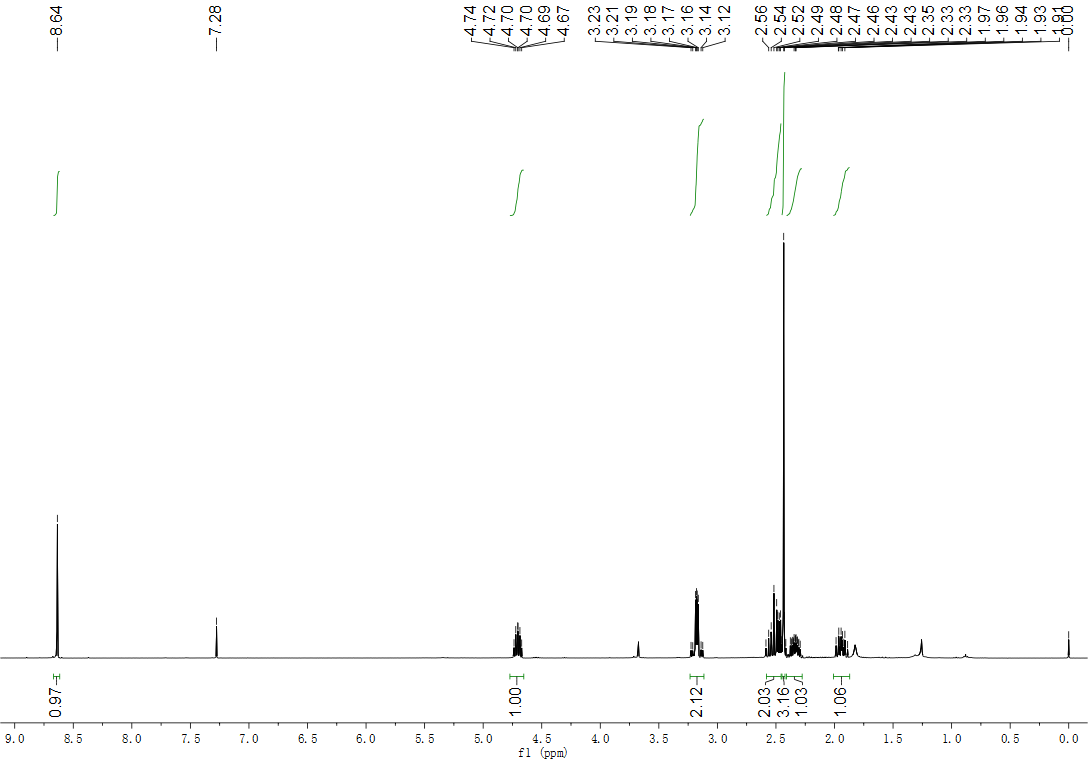
**

^1^H NMR (400 MHz, CDCl_3_) spectrum of **3p**

^
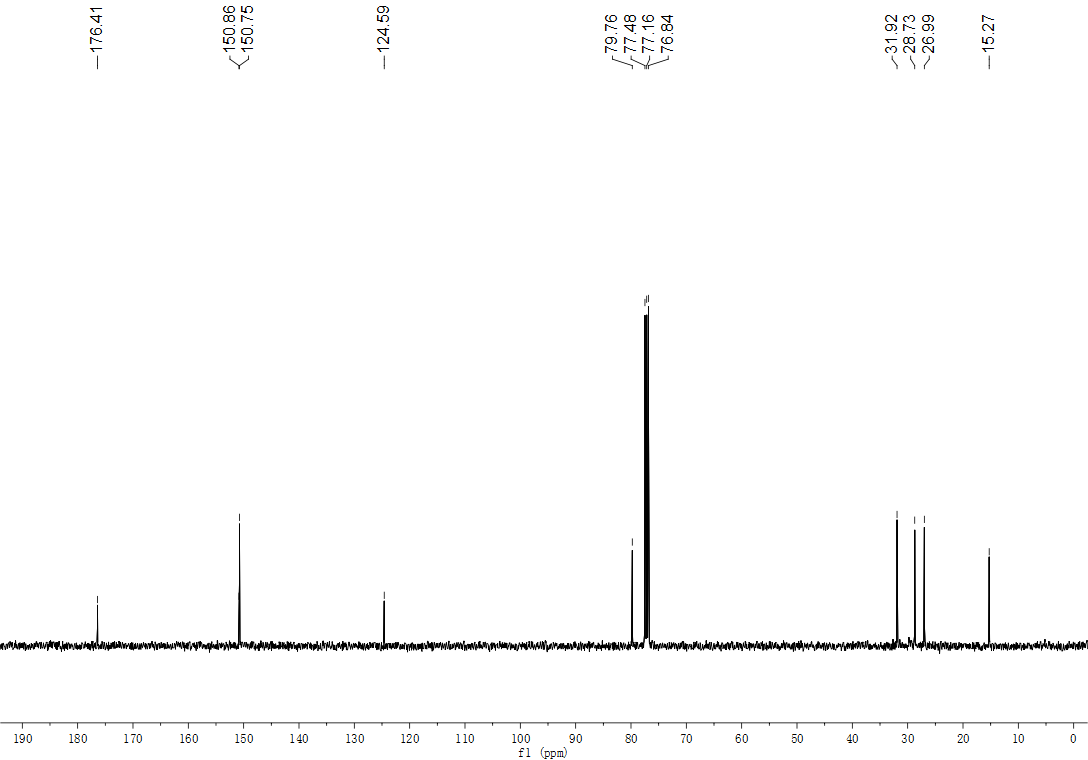
^

^13^C{^1^H} NMR (101 MHz, CDCl_3_) spectrum of **3p**

**
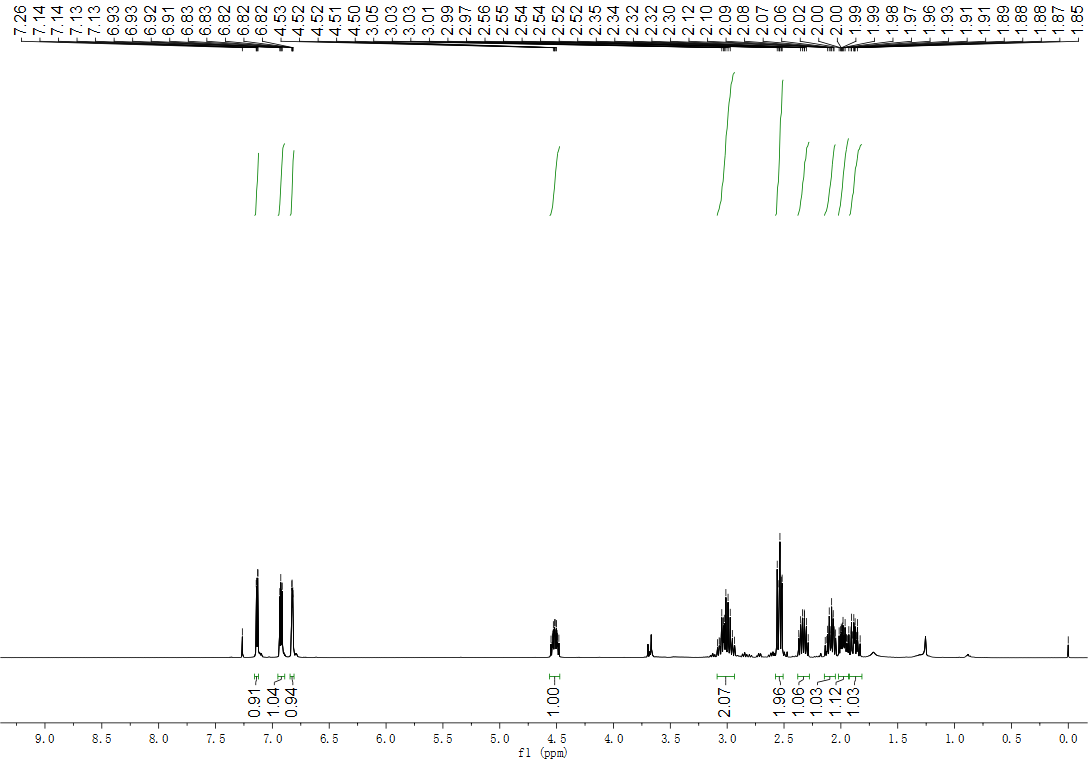
**

^1^H NMR (400 MHz, CDCl_3_) spectrum of **3q**

^
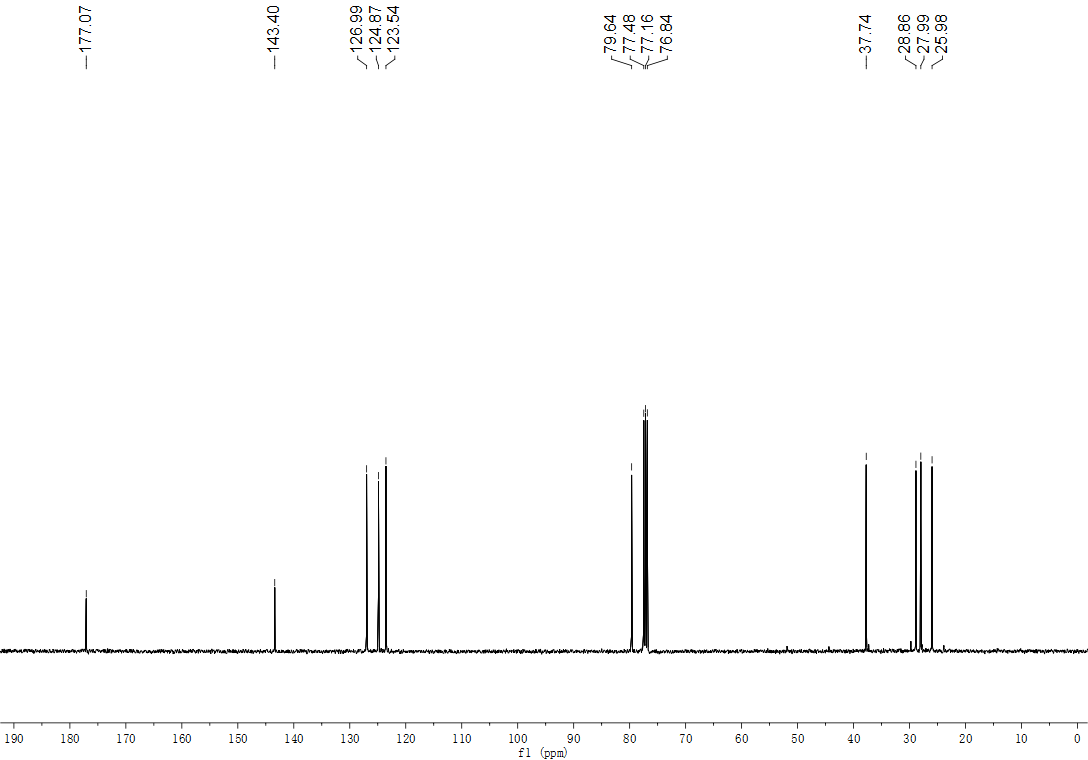
^

^13^C{^1^H} NMR (101 MHz, CDCl_3_) spectrum of **3q**

**
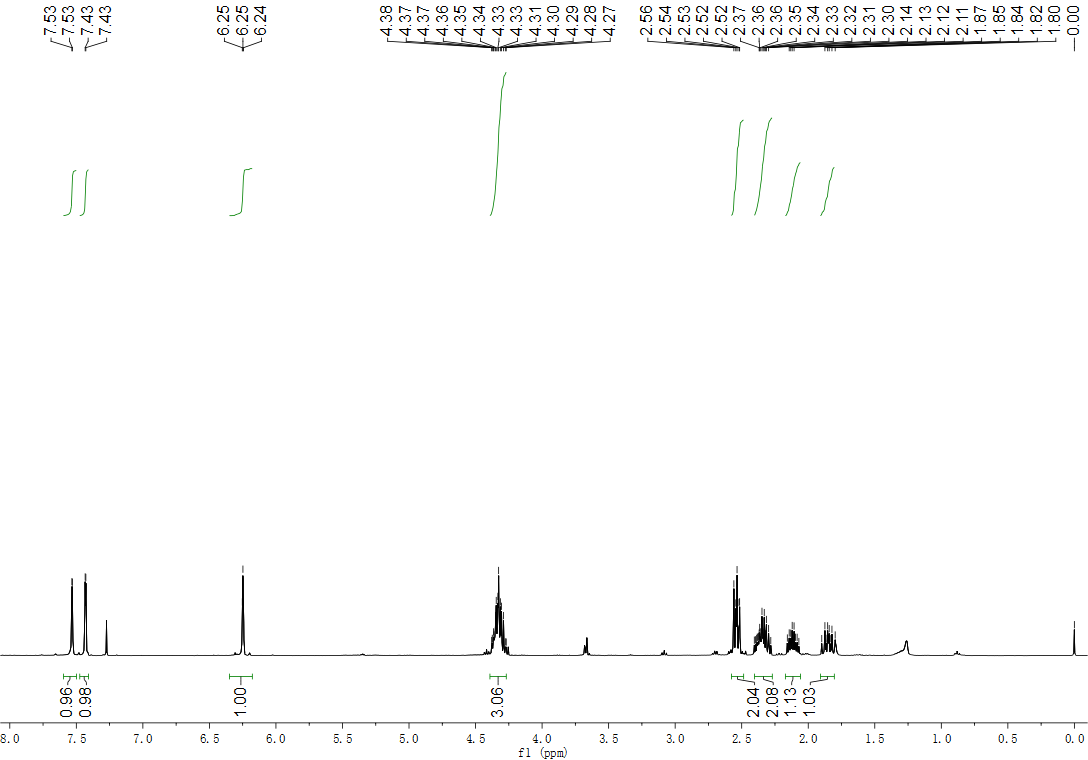
**

^1^H NMR (400 MHz, CDCl_3_) spectrum of **3r**

^
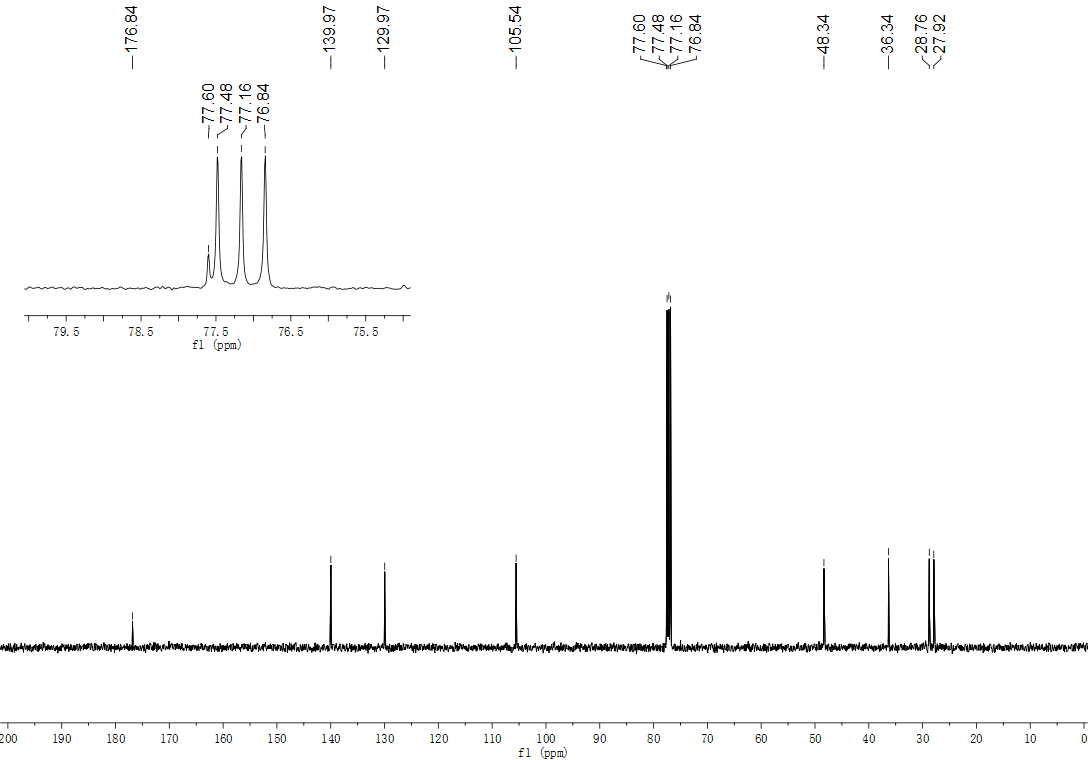
^

^13^C{^1^H} NMR (101 MHz, CDCl_3_) spectrum of **3r**

**
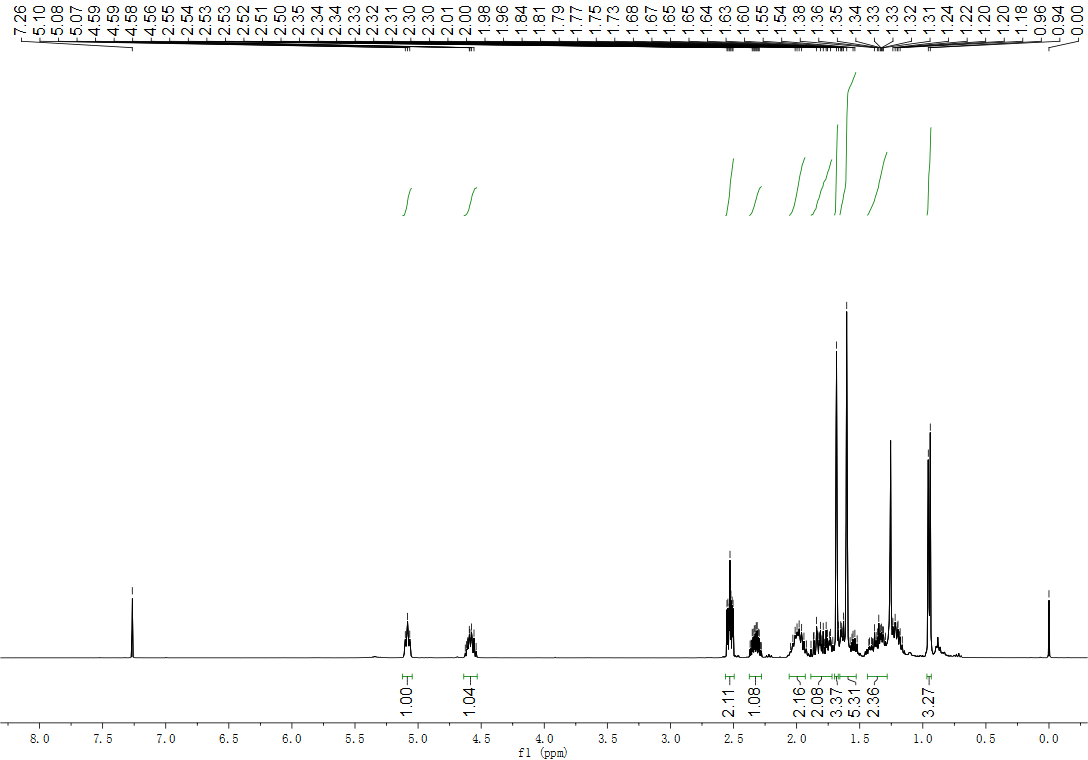
**

^1^H NMR (400 MHz, CDCl_3_) spectrum of **3s**

^
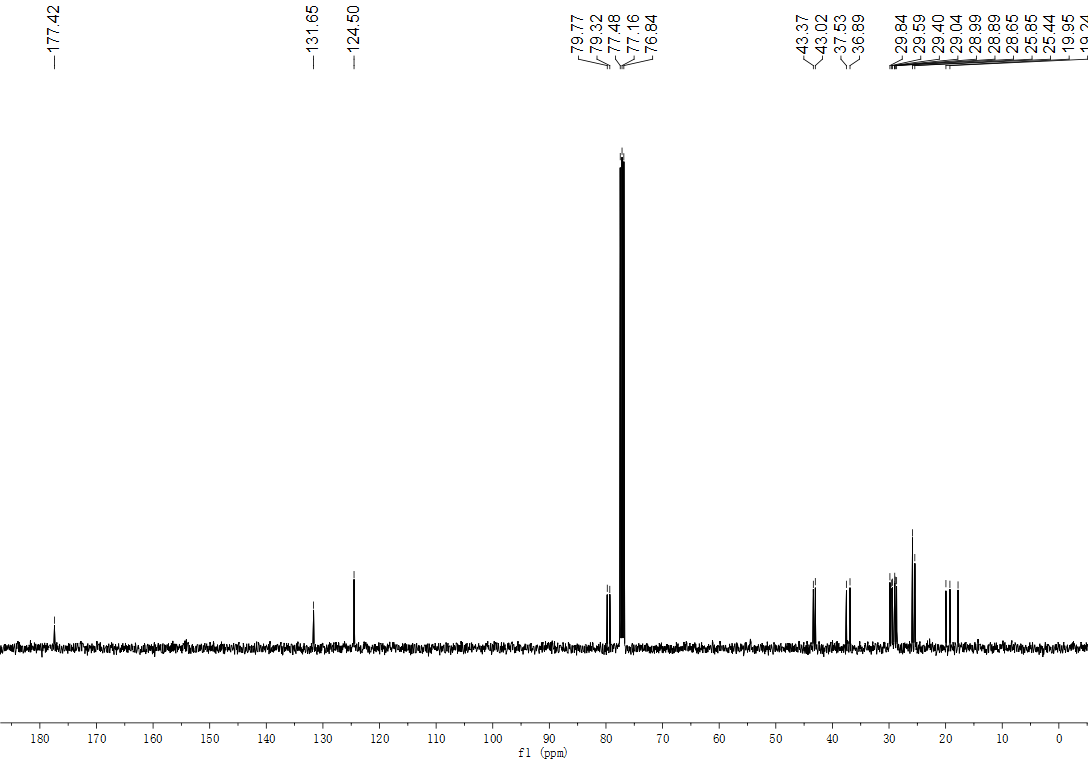
^

^13^C{^1^H} NMR (101 MHz, CDCl_3_) spectrum of **3s**

**
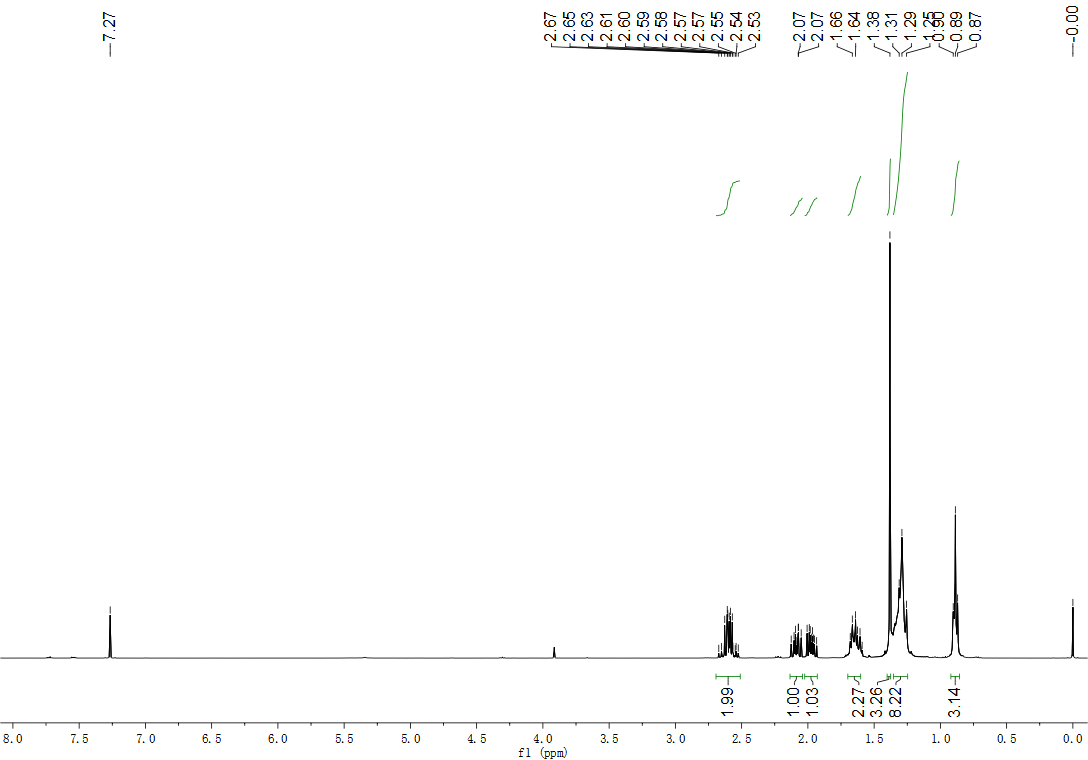
**

^1^H NMR (400 MHz, CDCl_3_) spectrum of **3t**

^
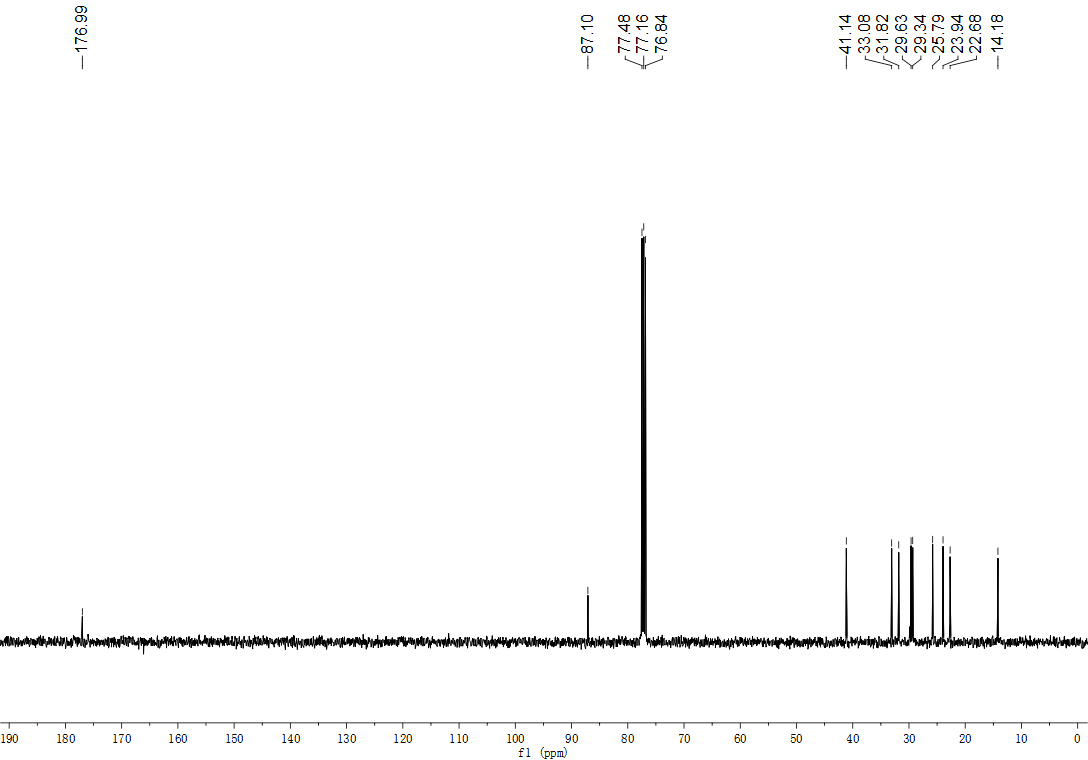
^

^13^C{^1^H} NMR (101 MHz, CDCl_3_) spectrum of **3t**

**
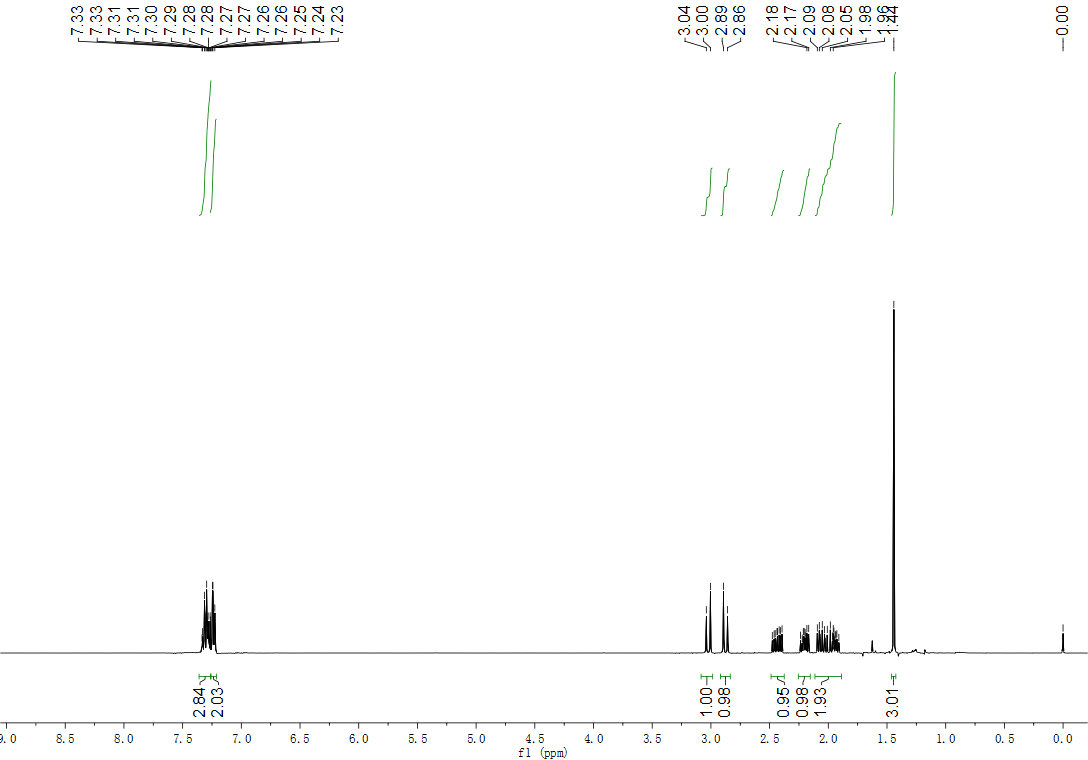
**

^1^H NMR (400 MHz, CDCl_3_) spectrum of **3u**

^
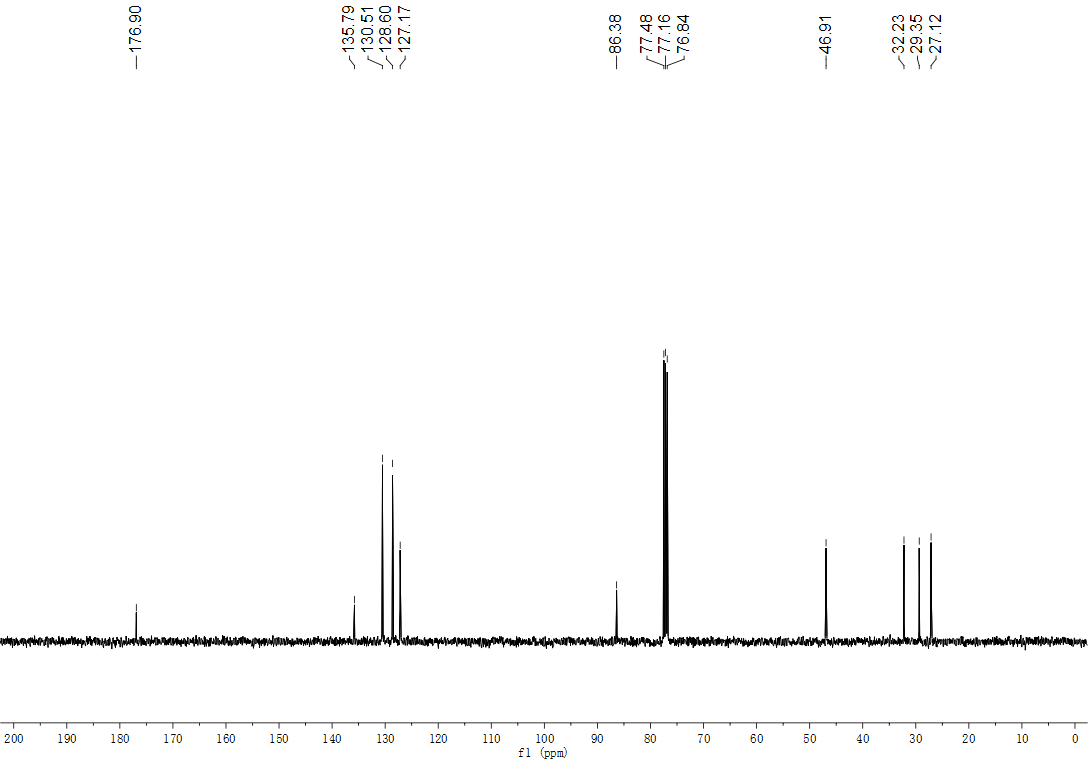
^

^13^C{^1^H} NMR (101 MHz, CDCl_3_) spectrum of **3u**

**
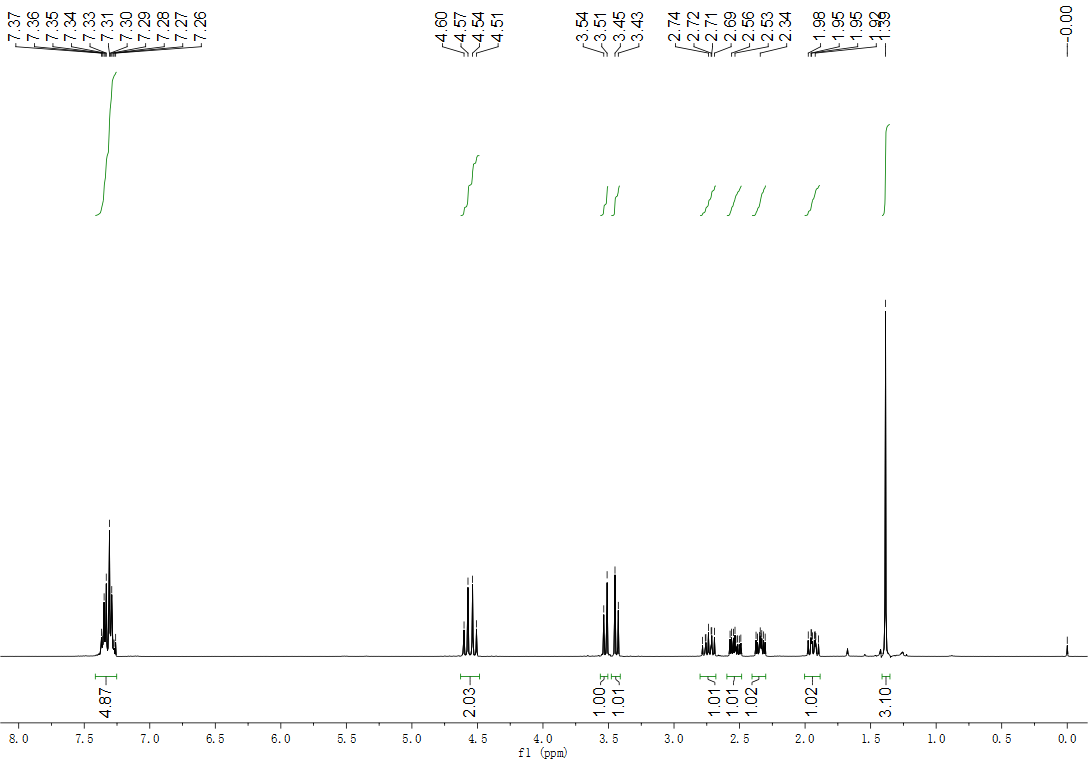
**

^1^H NMR (400 MHz, CDCl_3_) spectrum of **3v**

^
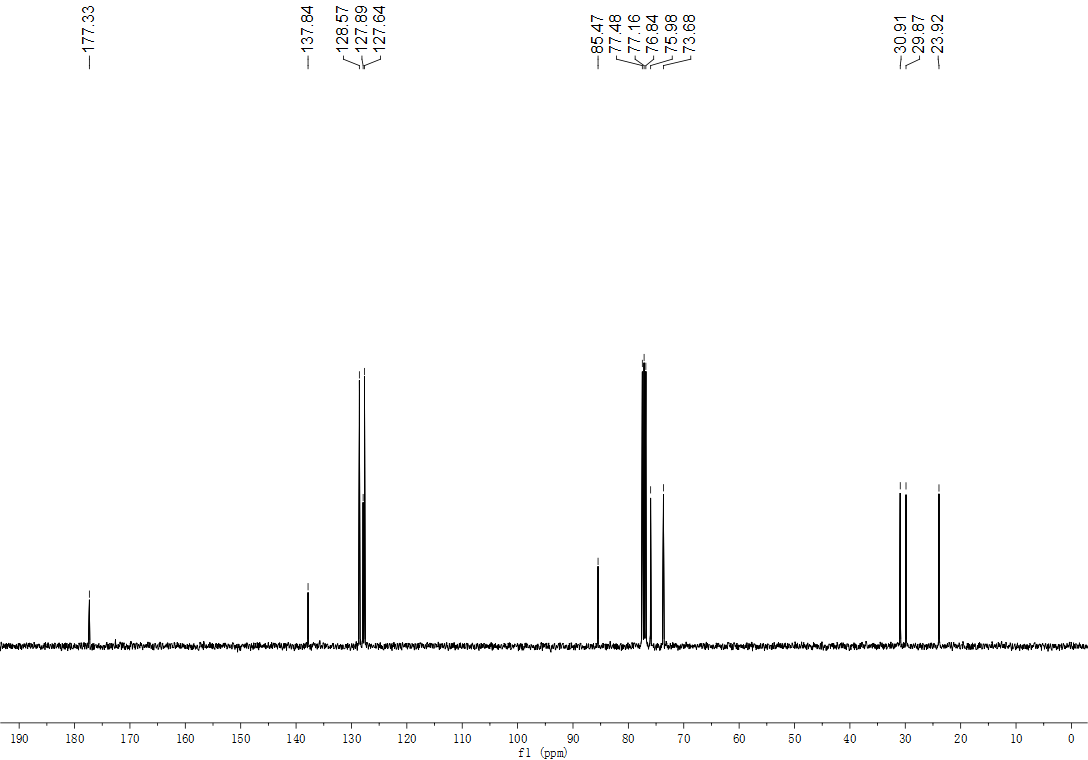
^

^13^C{^1^H} NMR (101 MHz, CDCl_3_) spectrum of **3v**

**
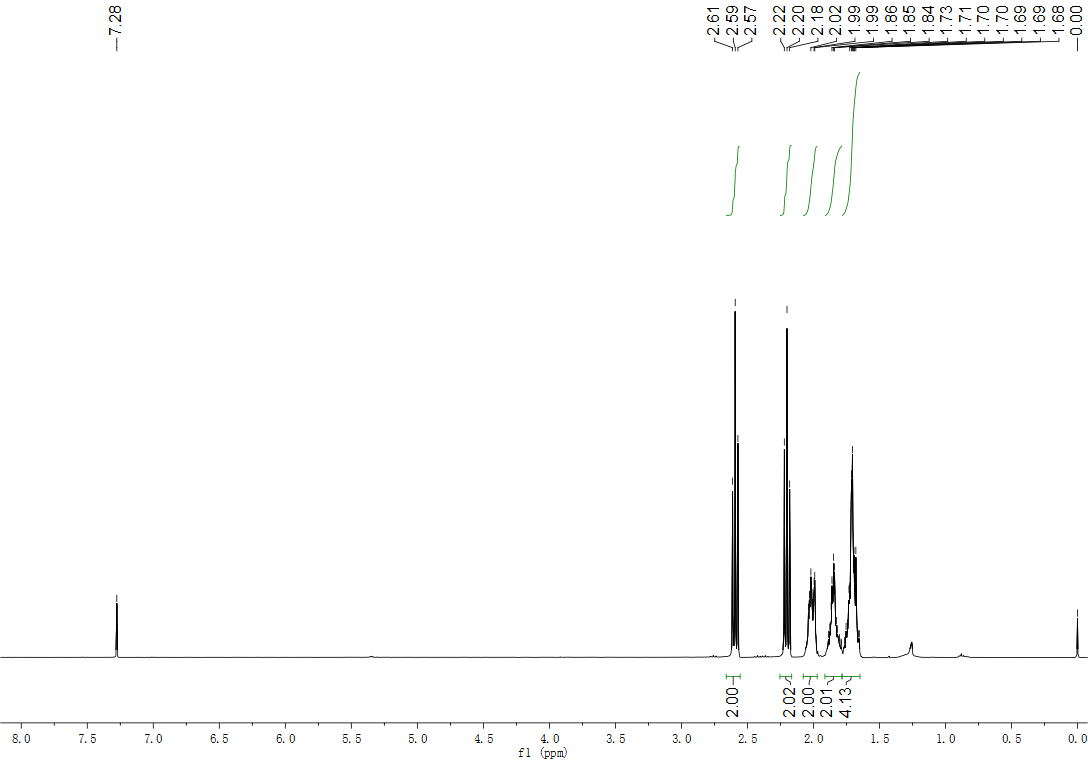
**

^1^H NMR (400 MHz, CDCl_3_) spectrum of **3w**

^
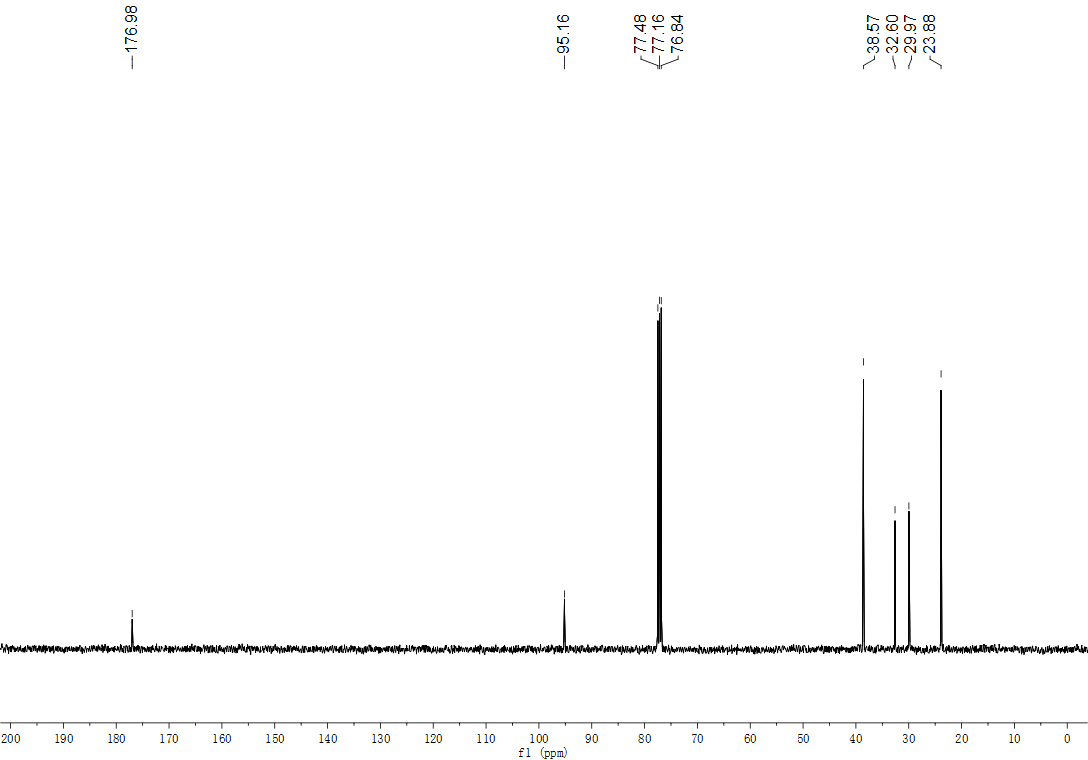
^

^13^C{^1^H} NMR (101 MHz, CDCl_3_) spectrum of **3w**

**
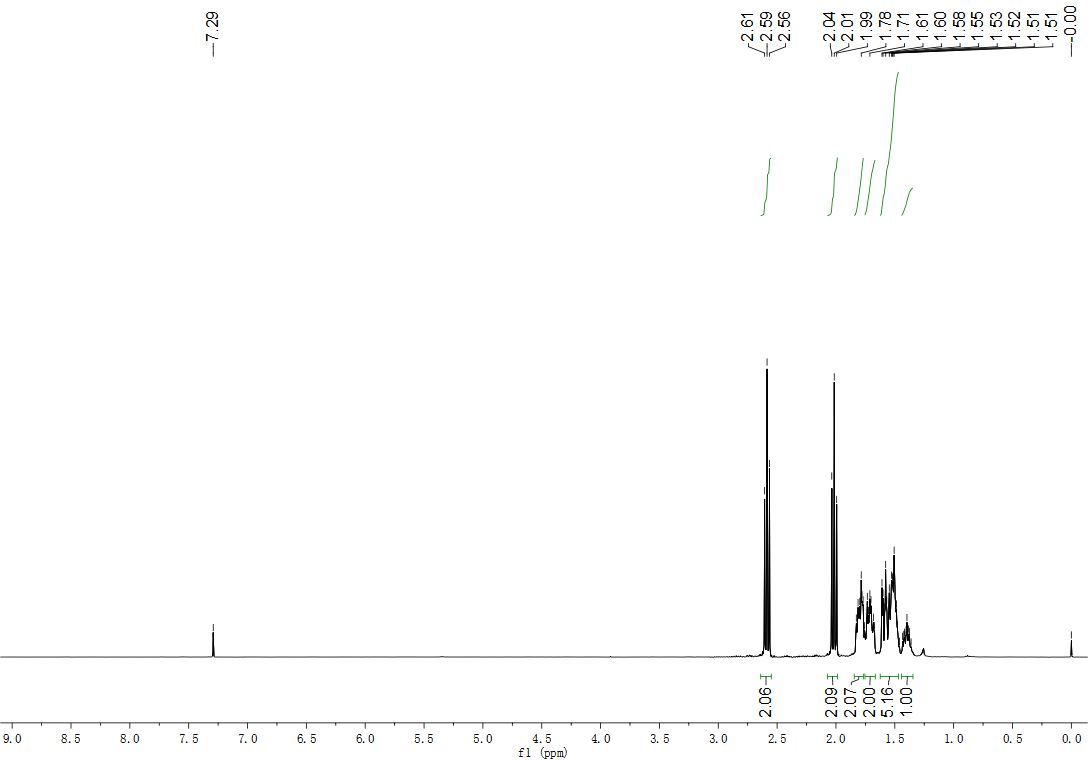
**

^1^H NMR (400 MHz, CDCl_3_) spectrum of **3x**

^
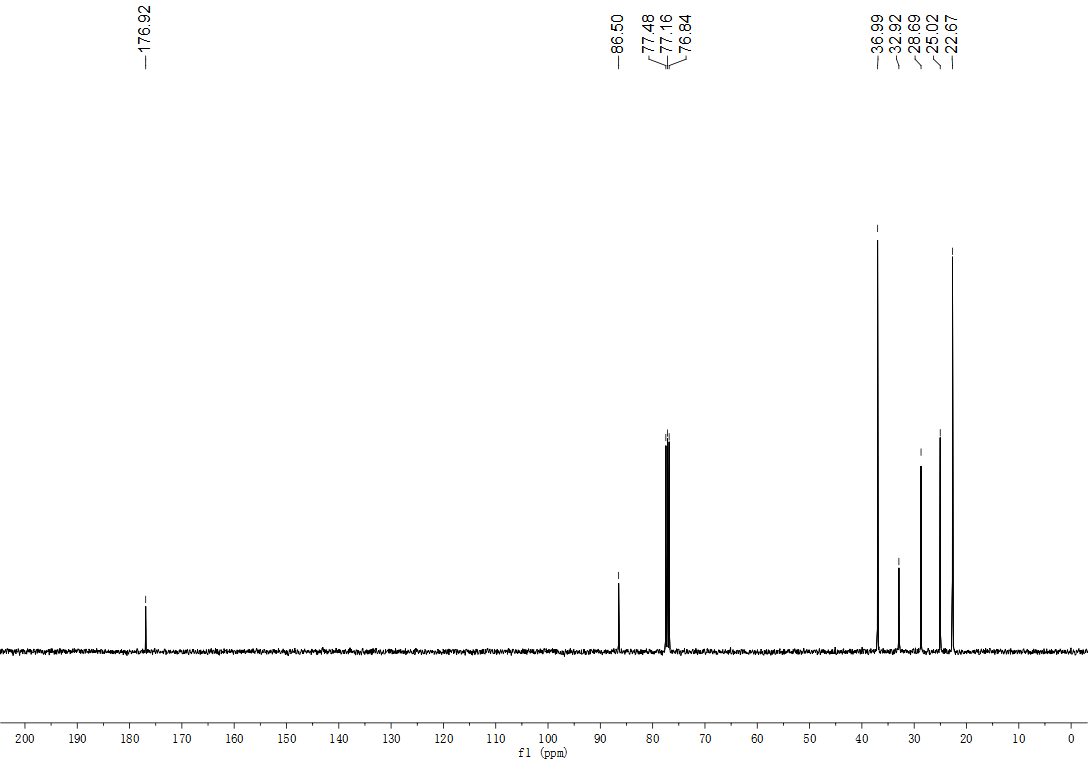
^

^13^C{^1^H} NMR (101 MHz, CDCl_3_) spectrum of **3x**

**
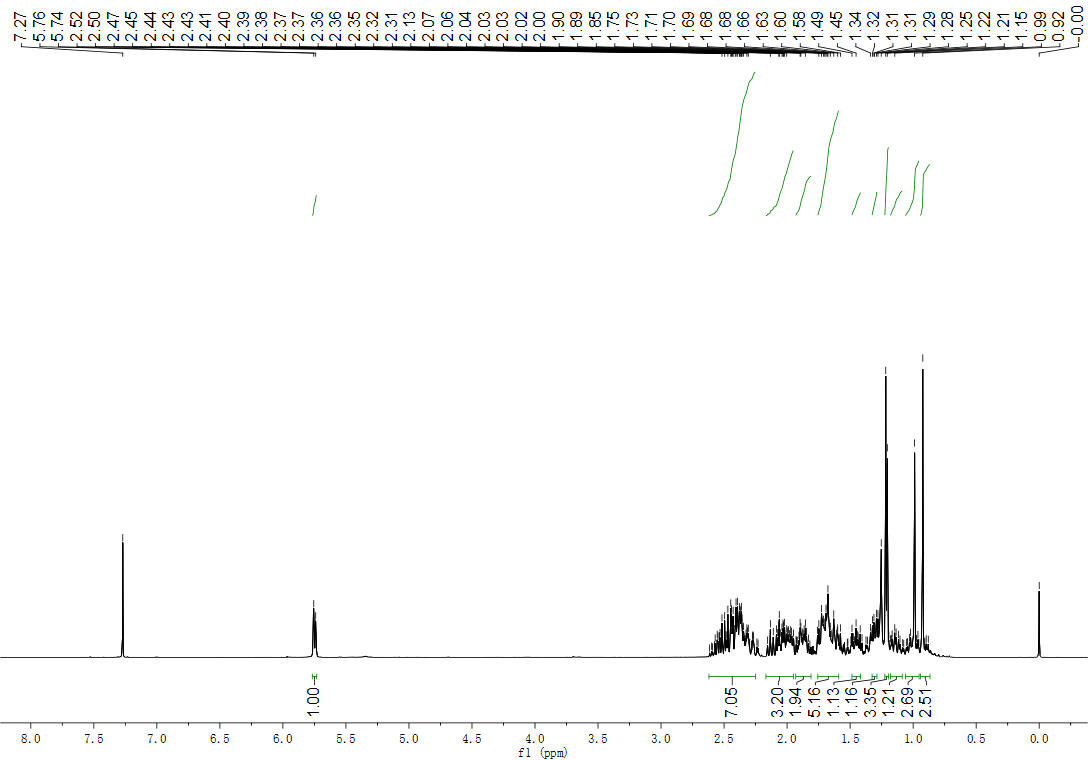
**

^1^H NMR (400 MHz, CDCl_3_) spectrum of **3y**

^
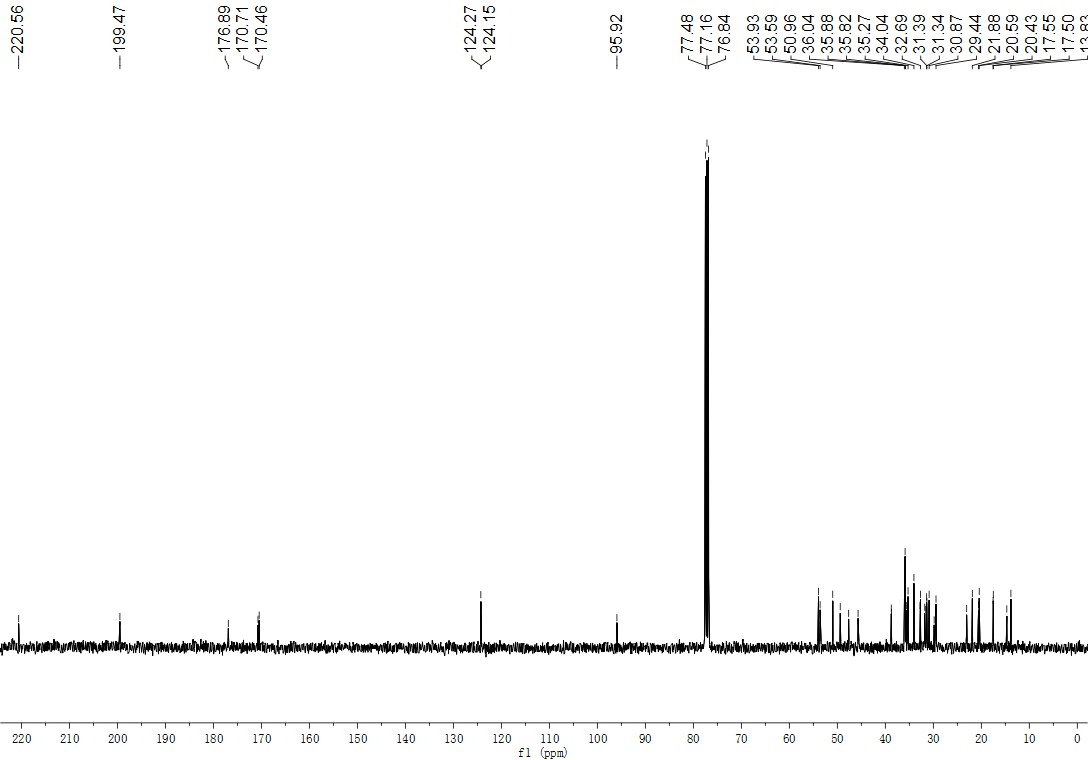
^

^13^C{^1^H} NMR (101 MHz, CDCl_3_) spectrum of **3y**

**
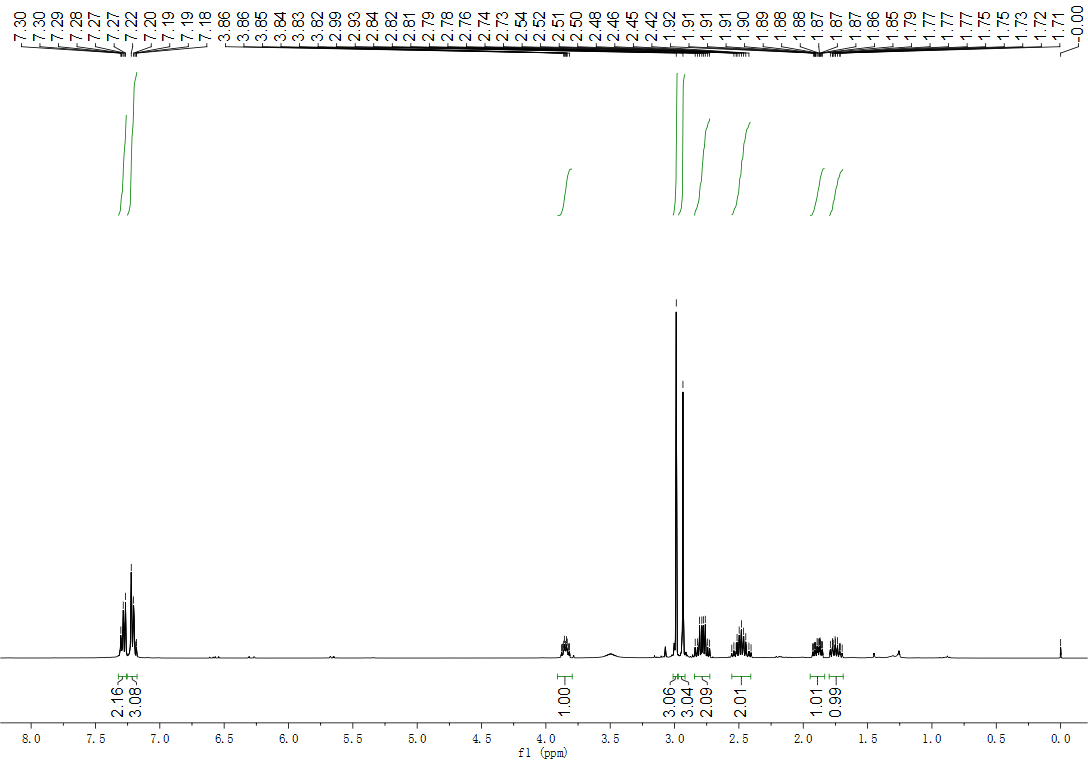
**

^1^H NMR (400 MHz, CDCl_3_) spectrum of **4a**

^
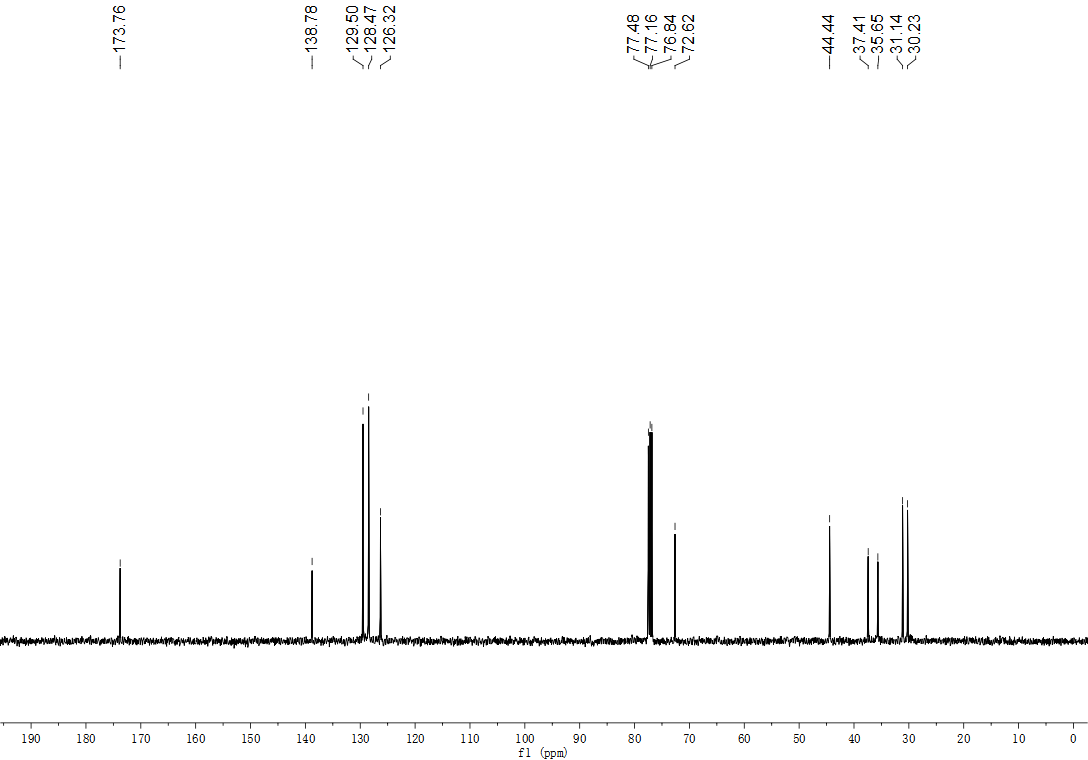
^

^13^C{^1^H} NMR (101 MHz, CDCl_3_) spectrum of **4a**

**
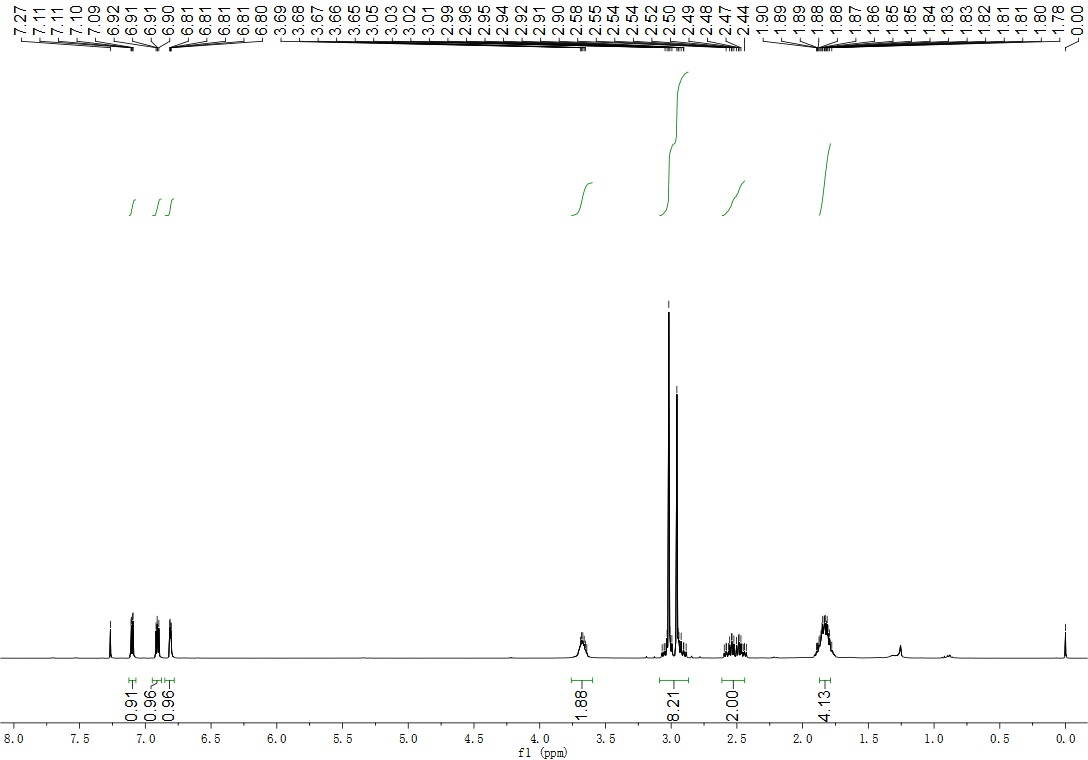
**

^1^H NMR (400 MHz, CDCl_3_) spectrum of **4b**

^
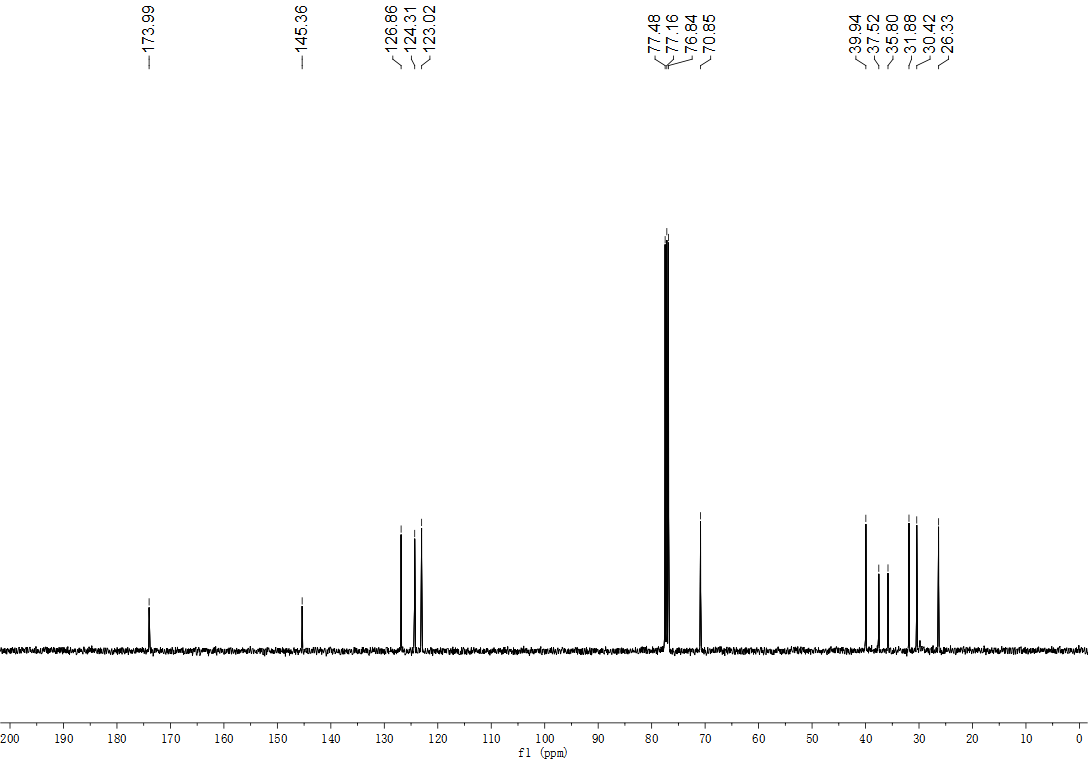
^

^13^C{^1^H} NMR (101 MHz, CDCl_3_) spectrum of **4b**

**
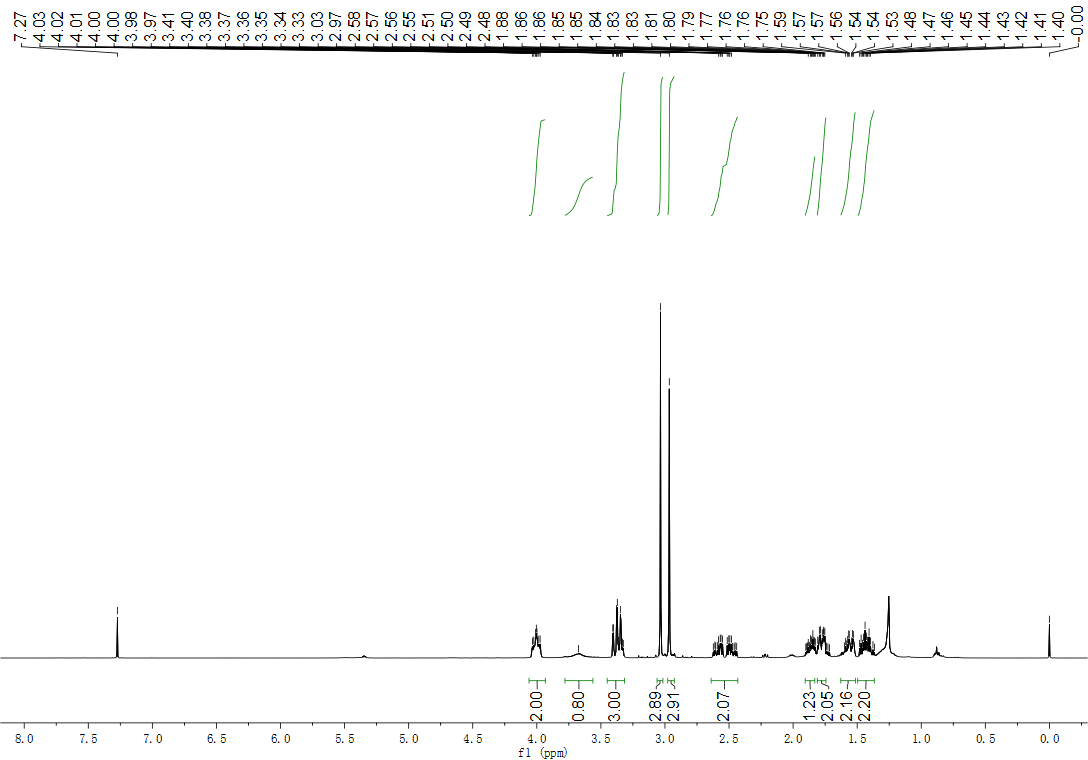
**

^1^H NMR (400 MHz, CDCl_3_) spectrum of **4c**

^
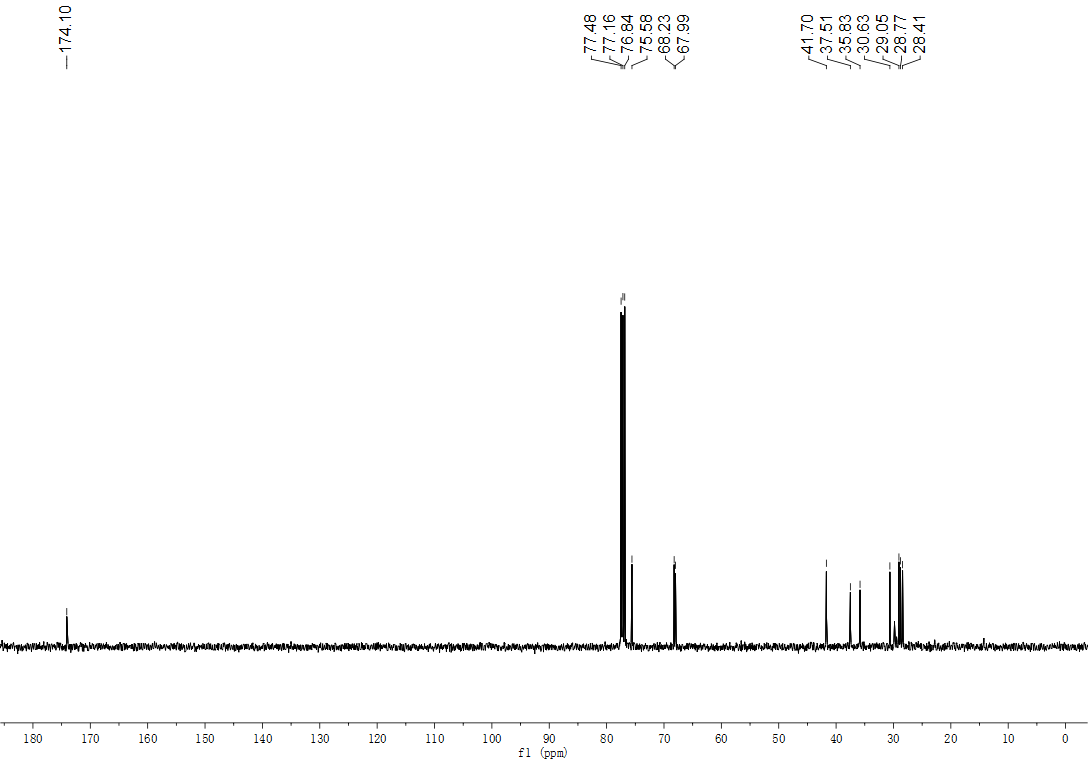
^

^13^C{^1^H} NMR (101 MHz, CDCl_3_) spectrum of **4c**

**
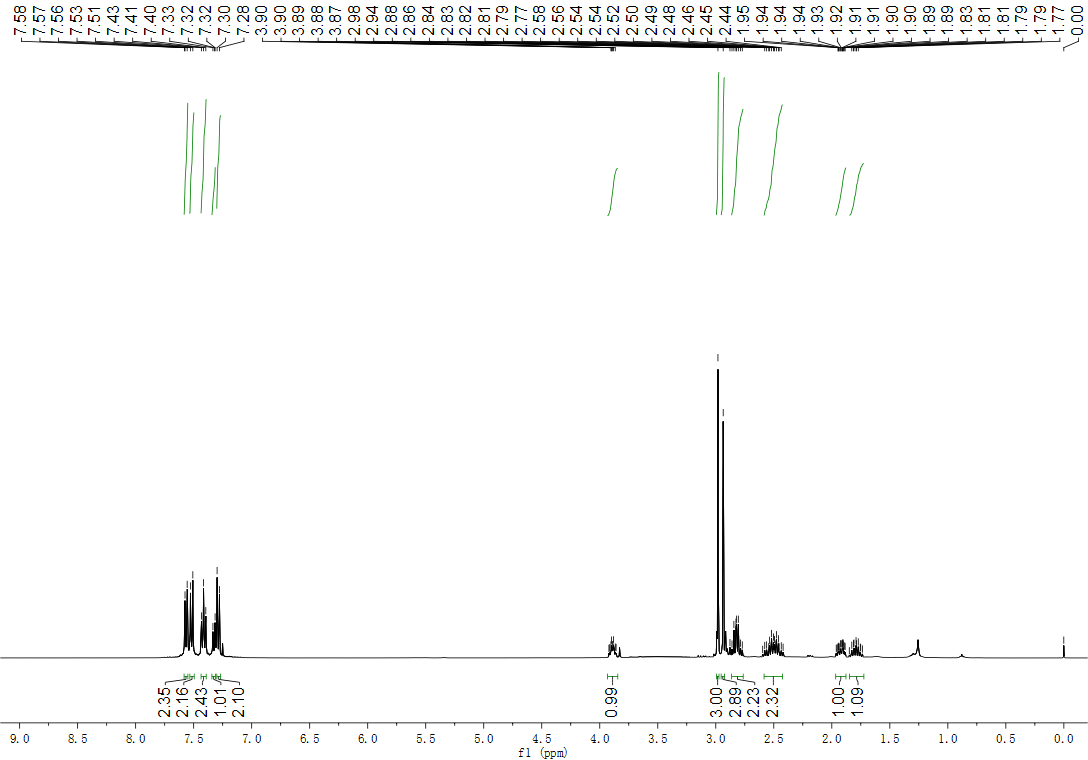
**

^1^H NMR (400 MHz, CDCl_3_) spectrum of **4d**

^
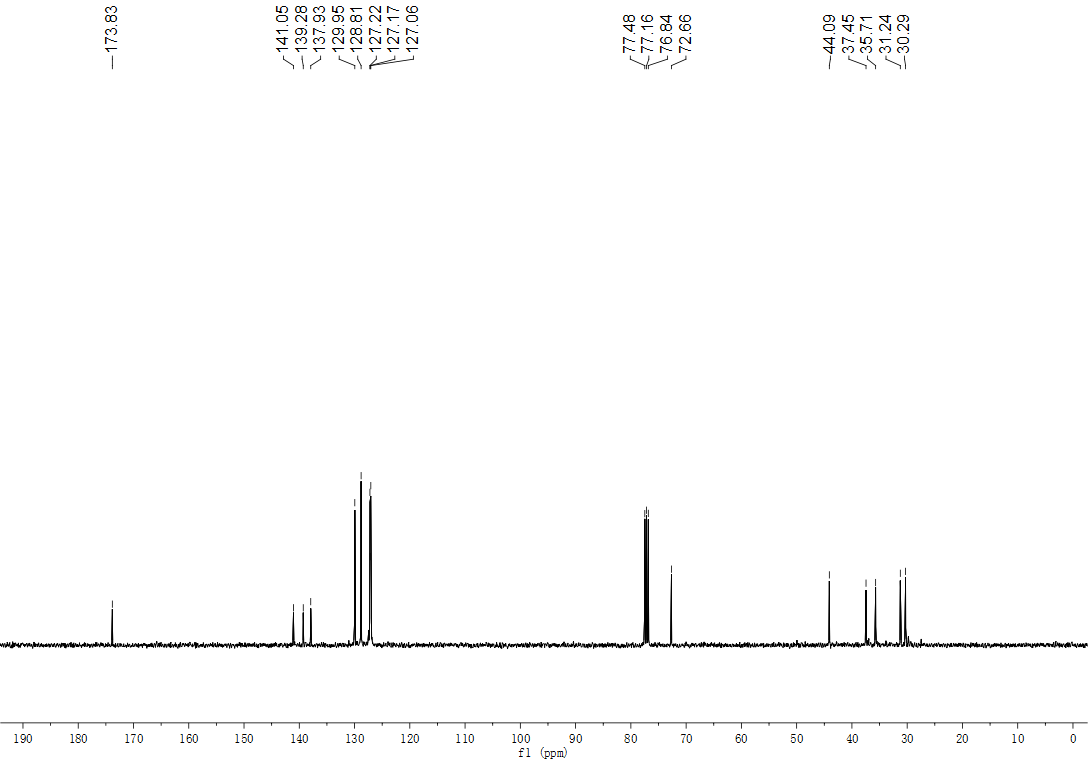
^

^13^C{^1^H} NMR (101 MHz, CDCl_3_) spectrum of **4d**

**
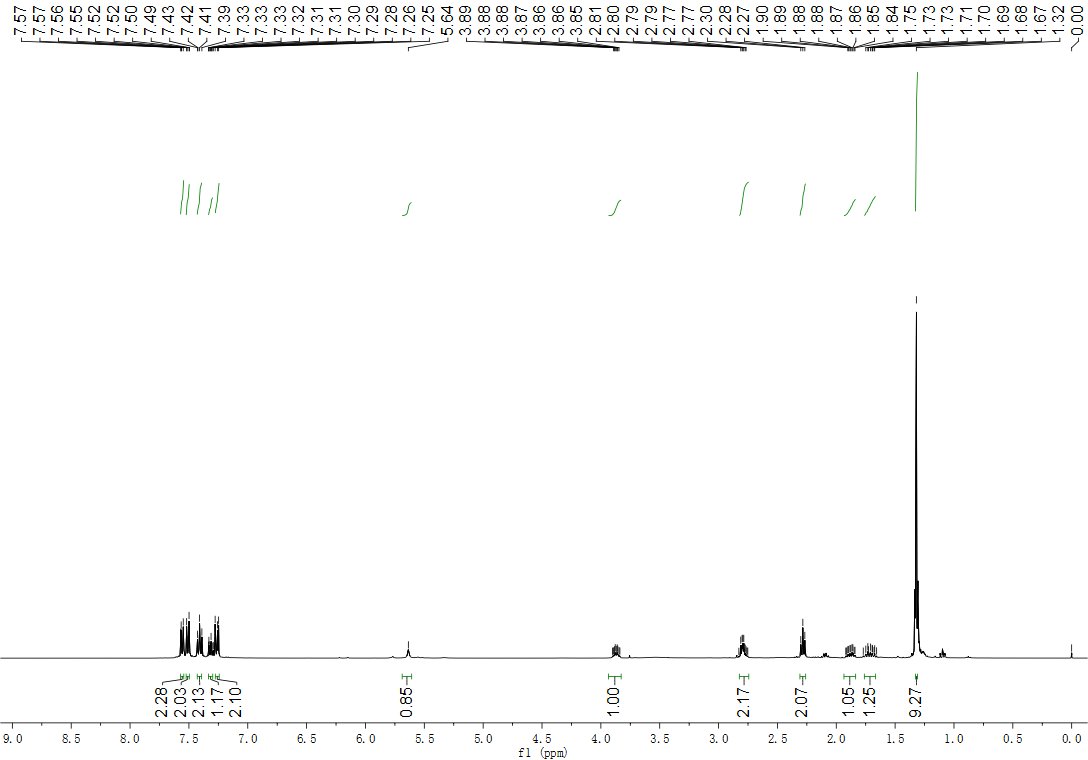
**

^1^H NMR (400 MHz, CDCl_3_) spectrum of **4e**

^
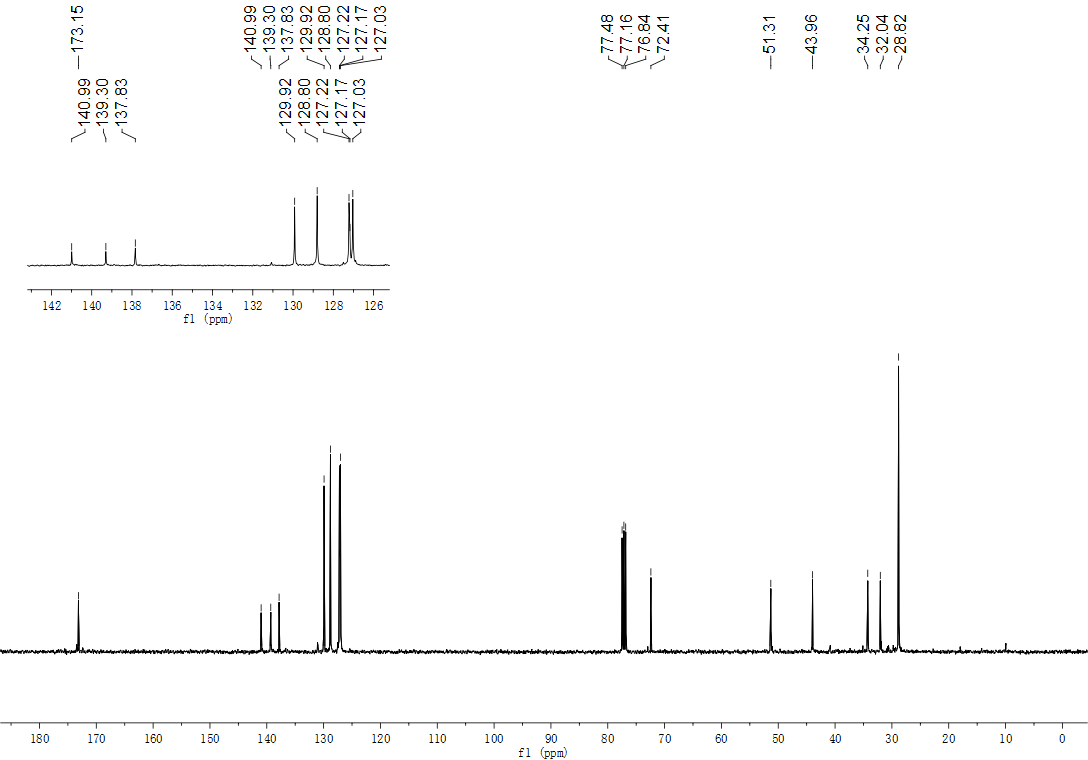
^

^13^C{^1^H} NMR (101 MHz, CDCl_3_) spectrum of **4e**

**
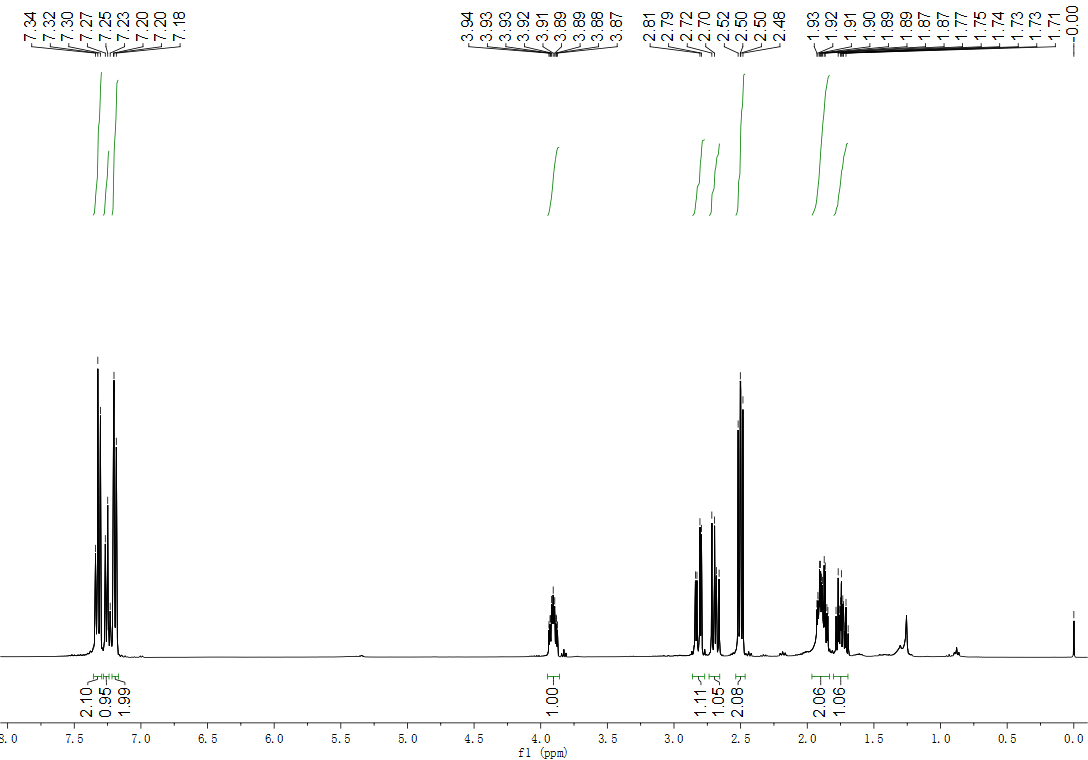
**

^1^H NMR (400 MHz, CDCl_3_) spectrum of **5a**

^
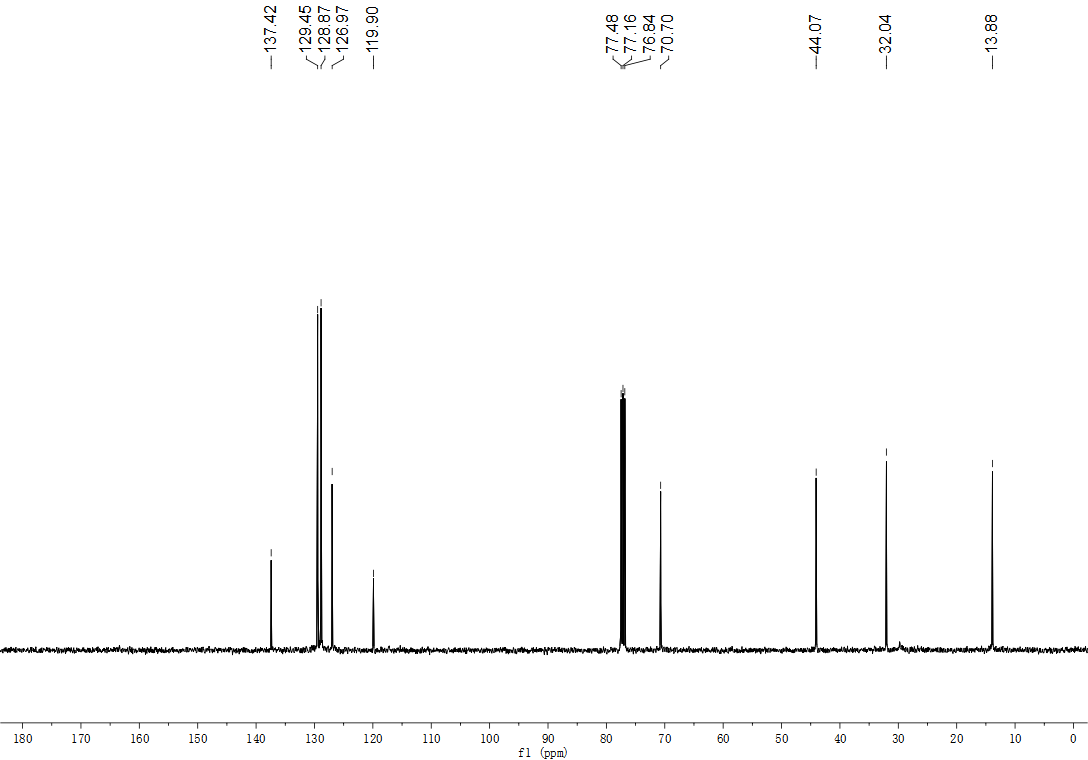
^

^13^C{^1^H} NMR (101 MHz, CDCl_3_) spectrum of **5a**

**
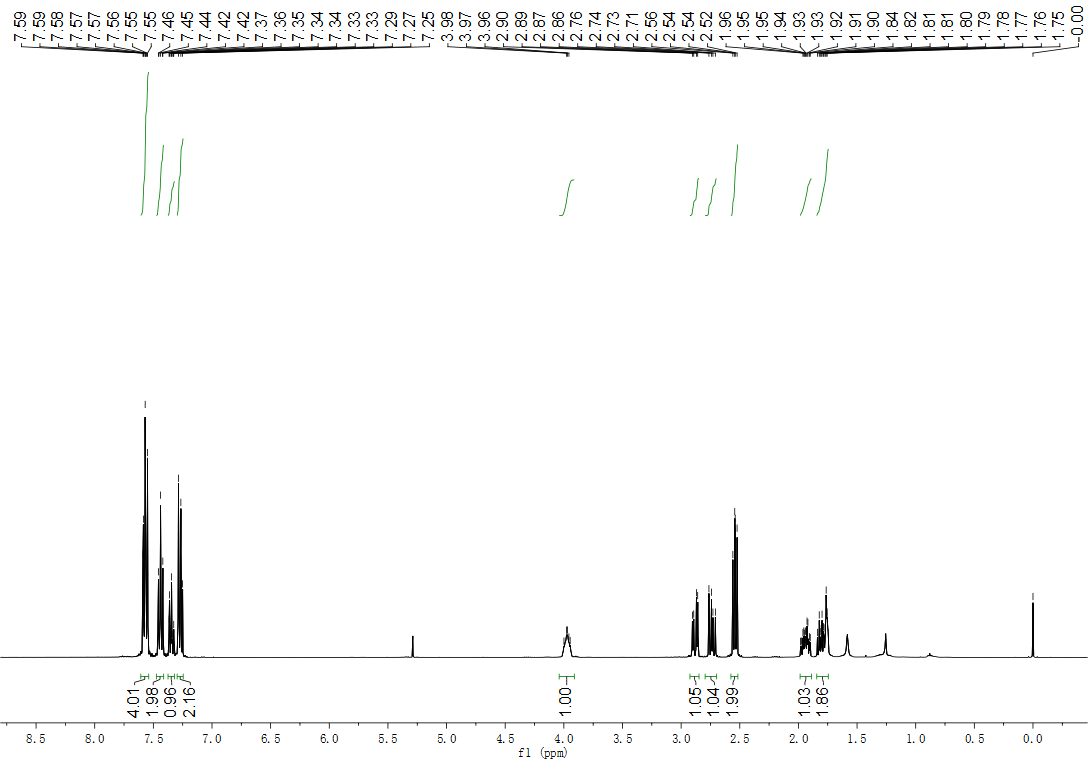
**

^1^H NMR (400 MHz, CDCl_3_) spectrum of **5b**

^
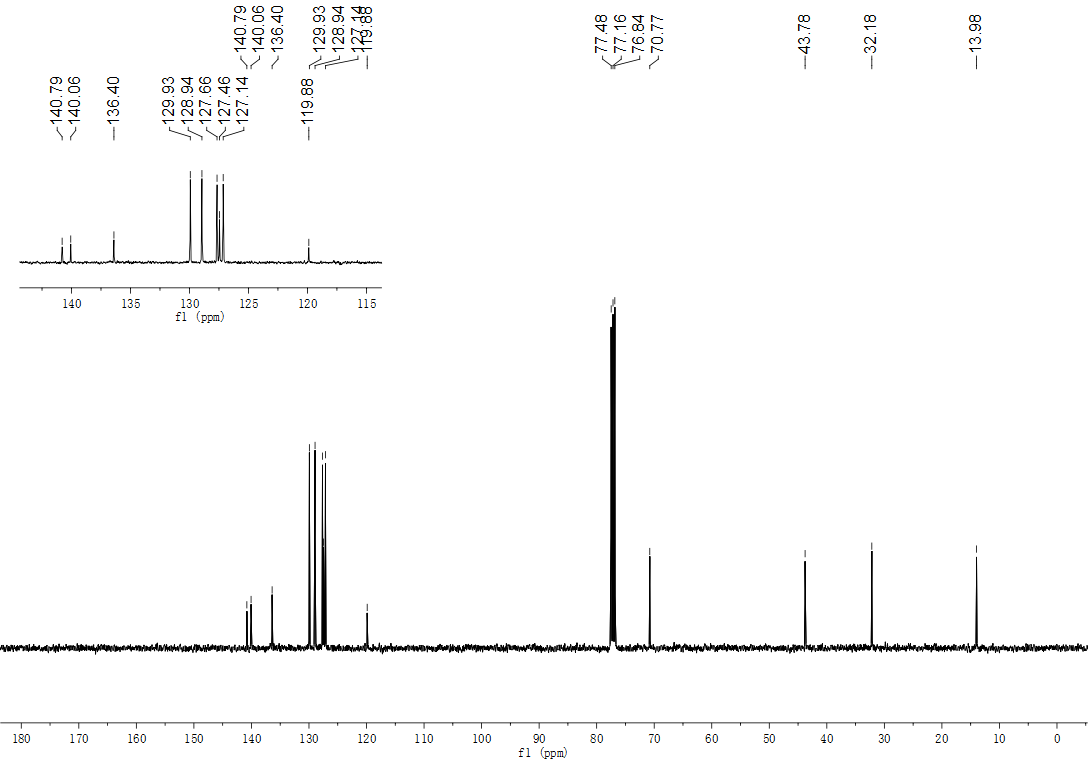
^

^13^C{^1^H} NMR (101 MHz, CDCl_3_) spectrum of **5b**

**
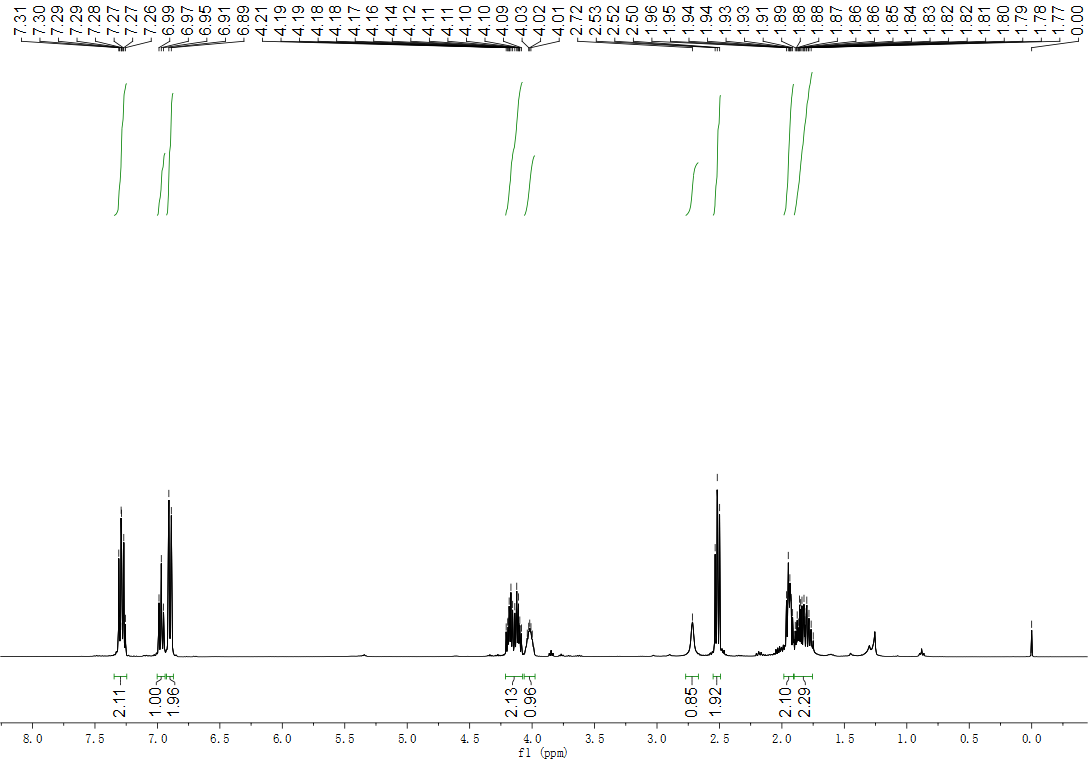
**

^1^H NMR (400 MHz, CDCl_3_) spectrum of **5c**

^
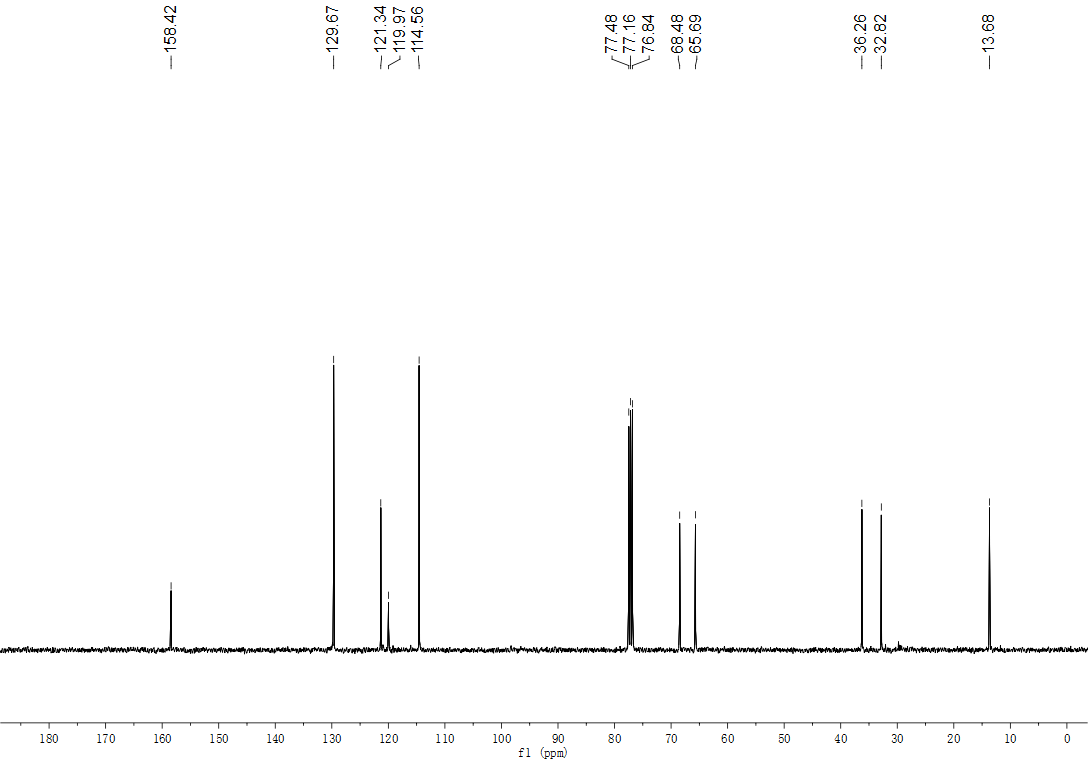
^

^13^C{^1^H} NMR (101 MHz, CDCl_3_) spectrum of **5c**

**
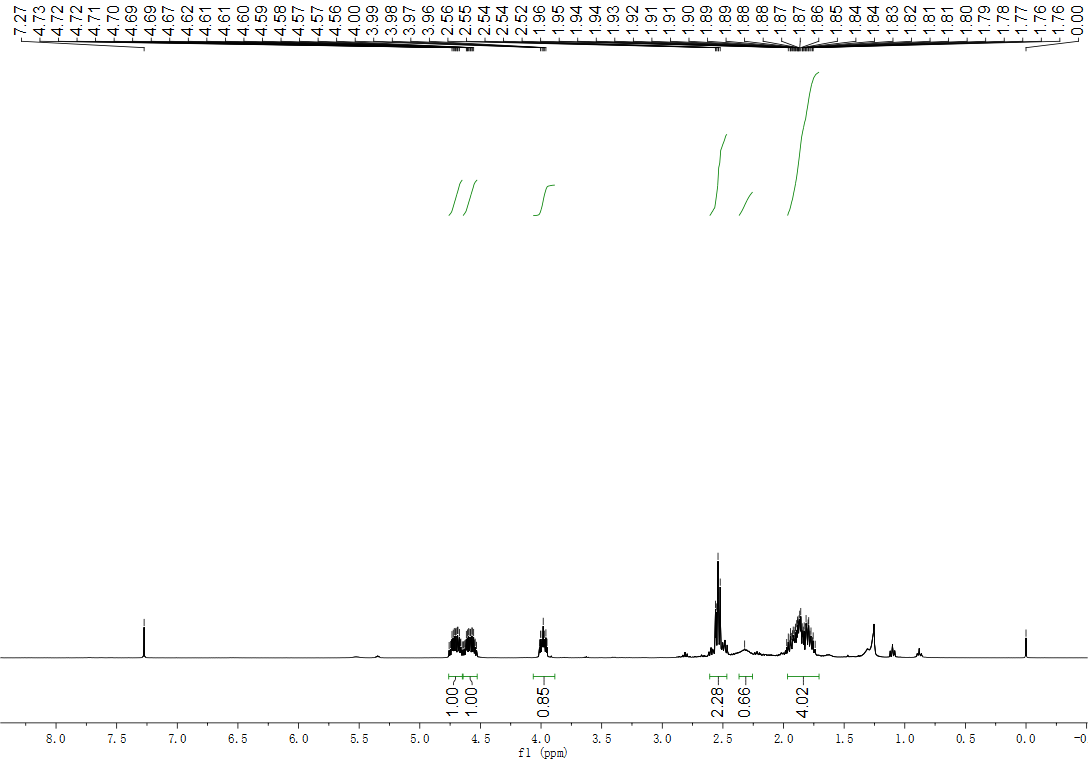
**

^1^H NMR (400 MHz, CDCl_3_) spectrum of **5d**

^
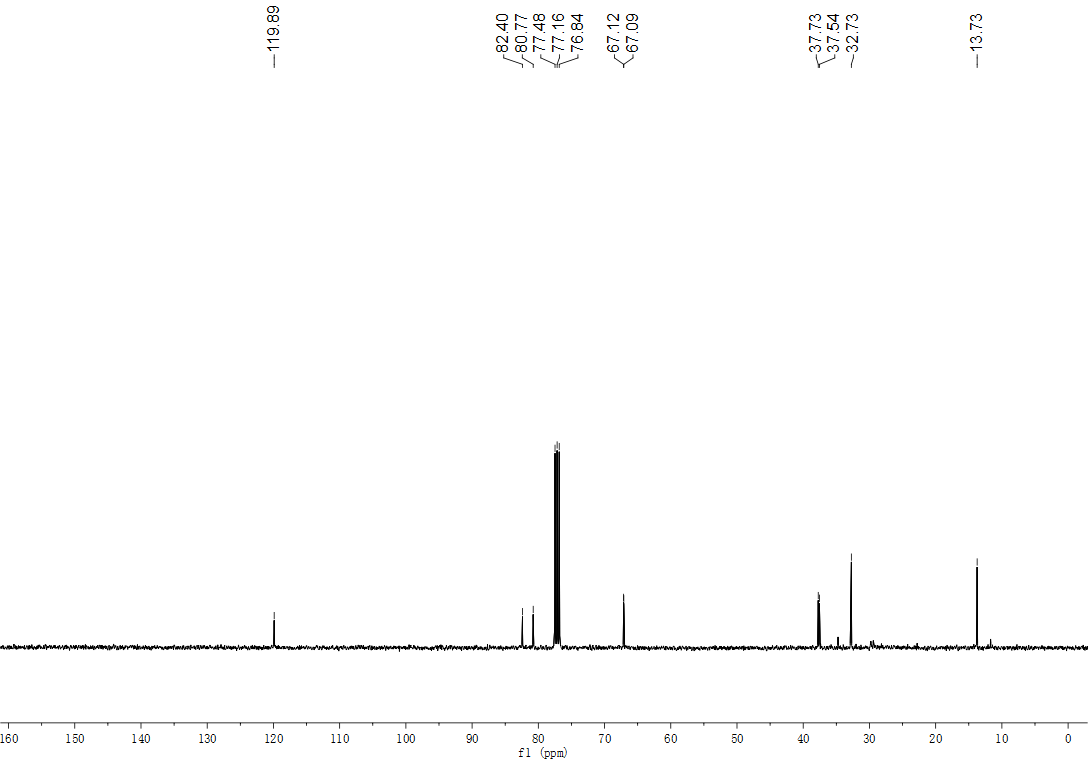
^

^13^C{^1^H} NMR (101 MHz, CDCl_3_) spectrum of **5d**

^
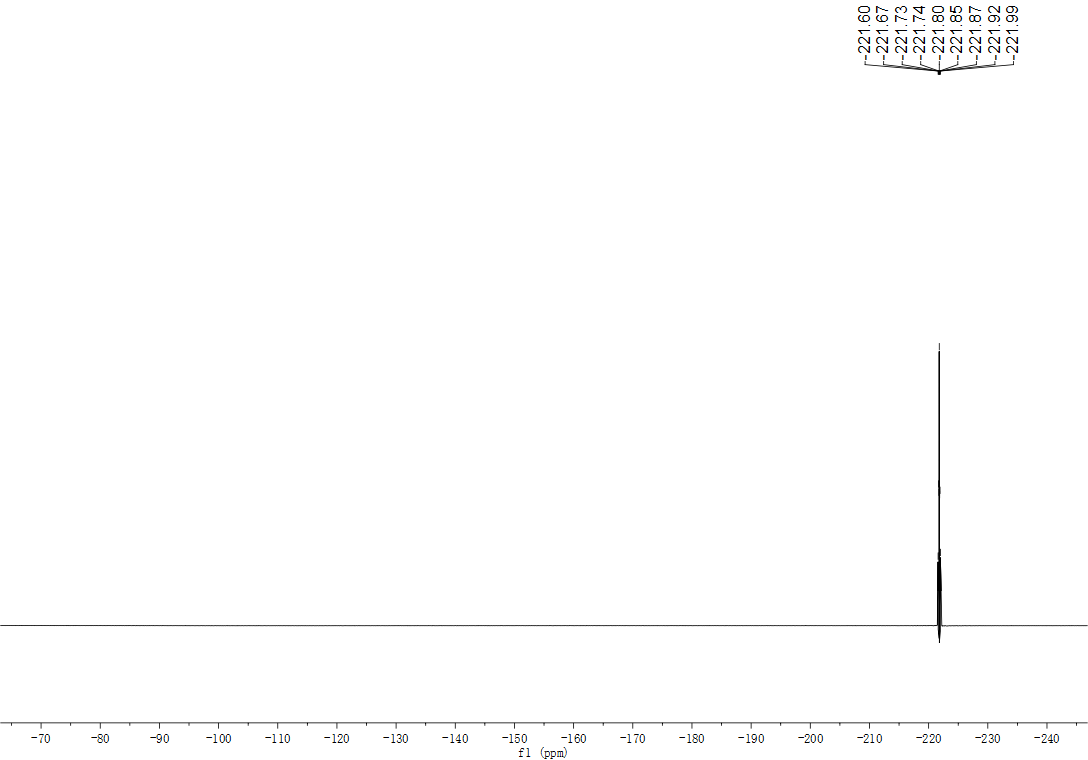
^

^19^F NMR (376 MHz, CDCl_3_) spectrum of **5d**

**
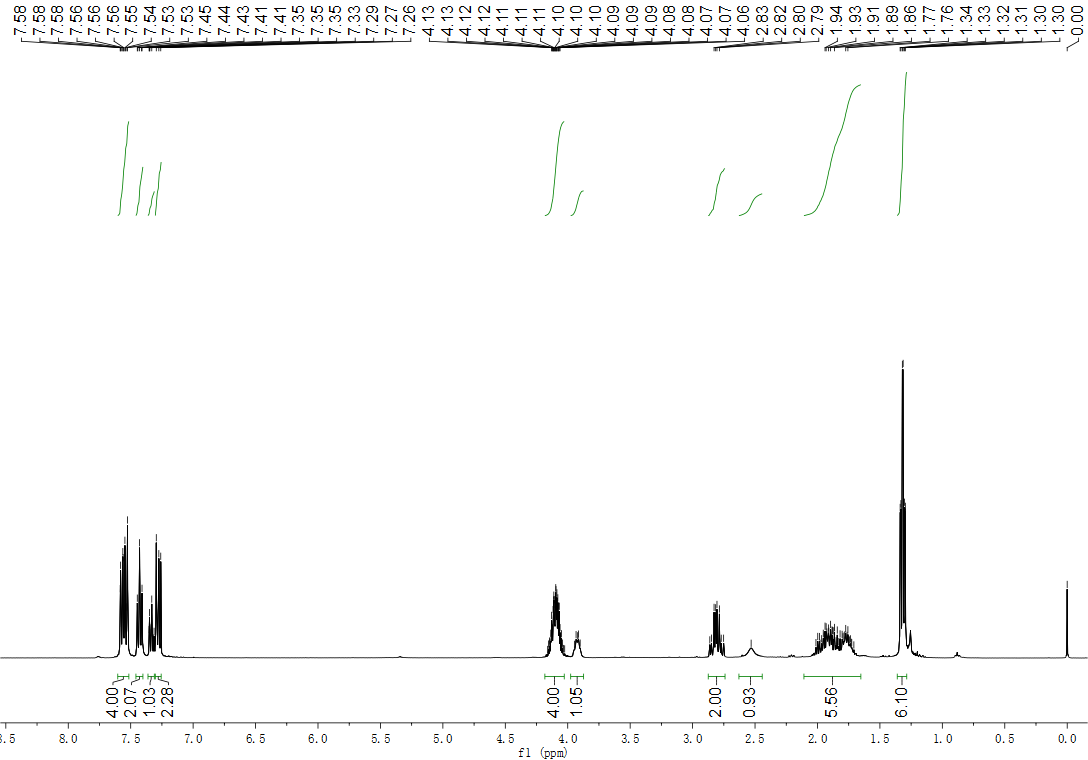
**

^1^H NMR (400 MHz, CDCl_3_) spectrum of **6a**

^
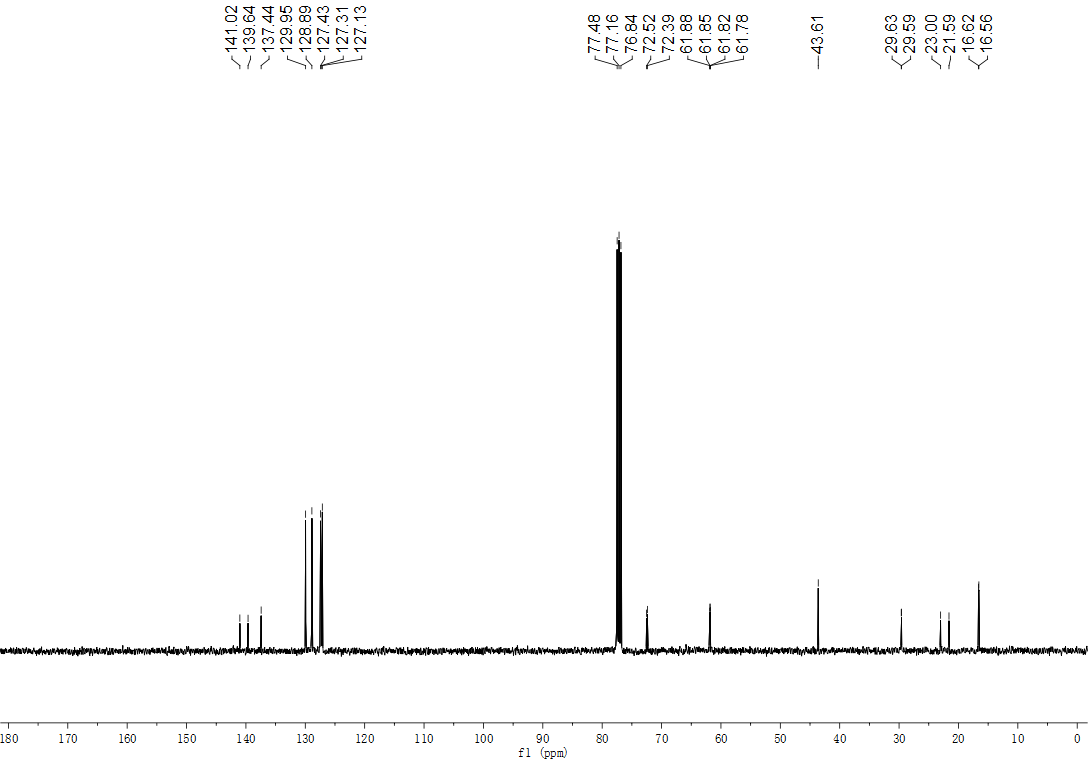
^

^13^C{^1^H} NMR (101 MHz, CDCl_3_) spectrum of **6a**

^
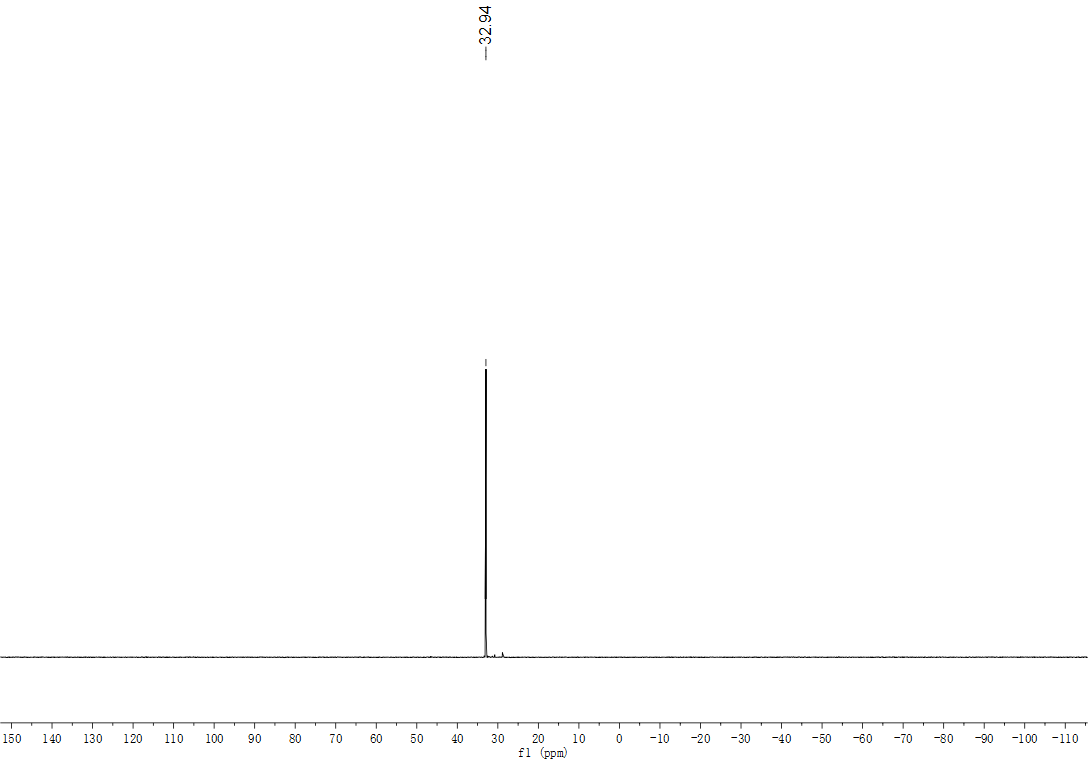
^

^31^P NMR (162 MHz, CDCl_3_) spectrum of **6a**

**
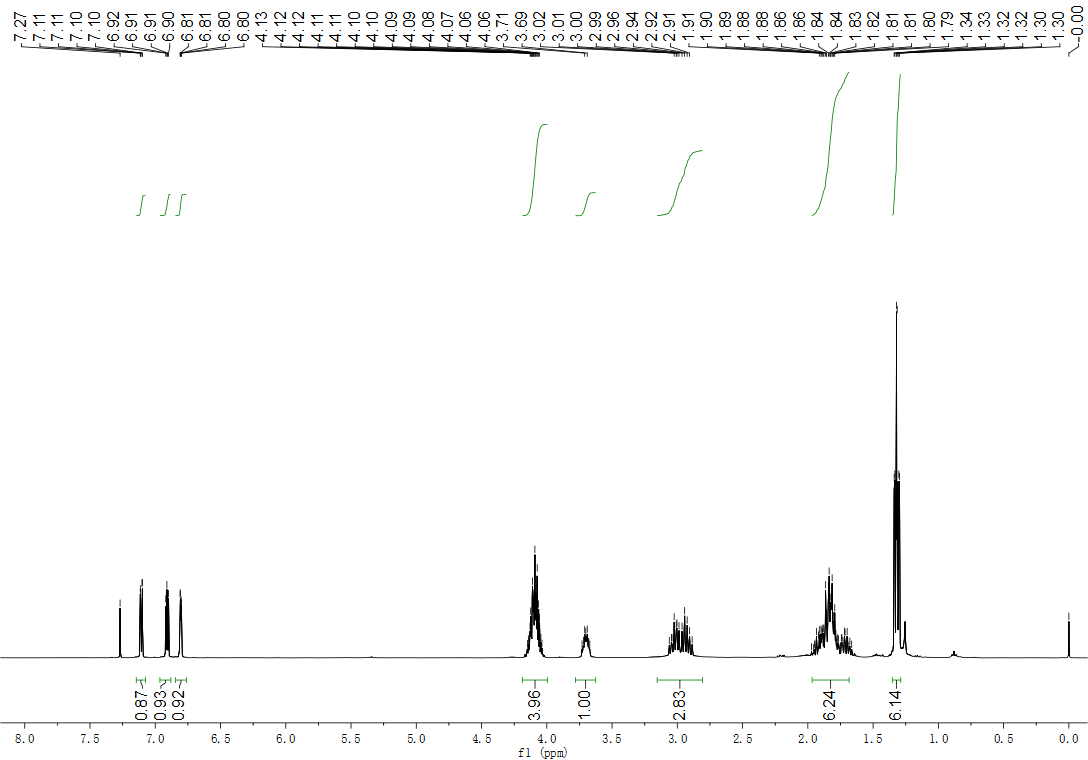
**

^1^H NMR (400 MHz, CDCl_3_) spectrum of **6b**

^
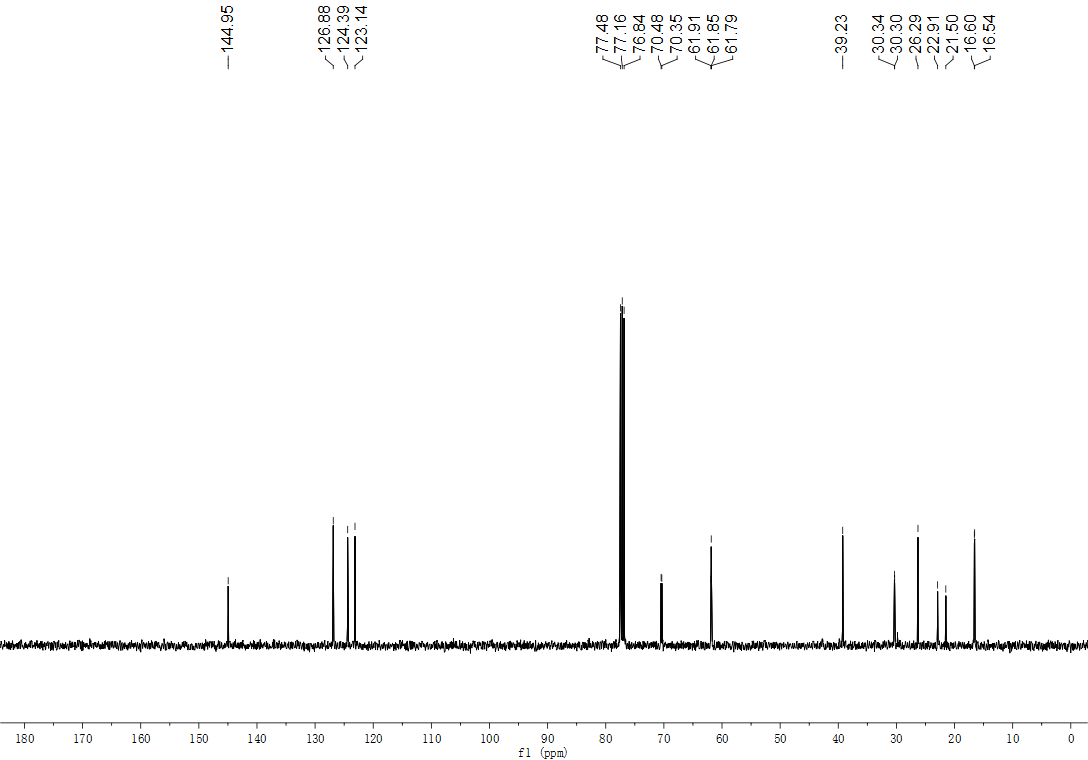
^

^13^C{^1^H} NMR (101 MHz, CDCl_3_) spectrum of **6b**

^
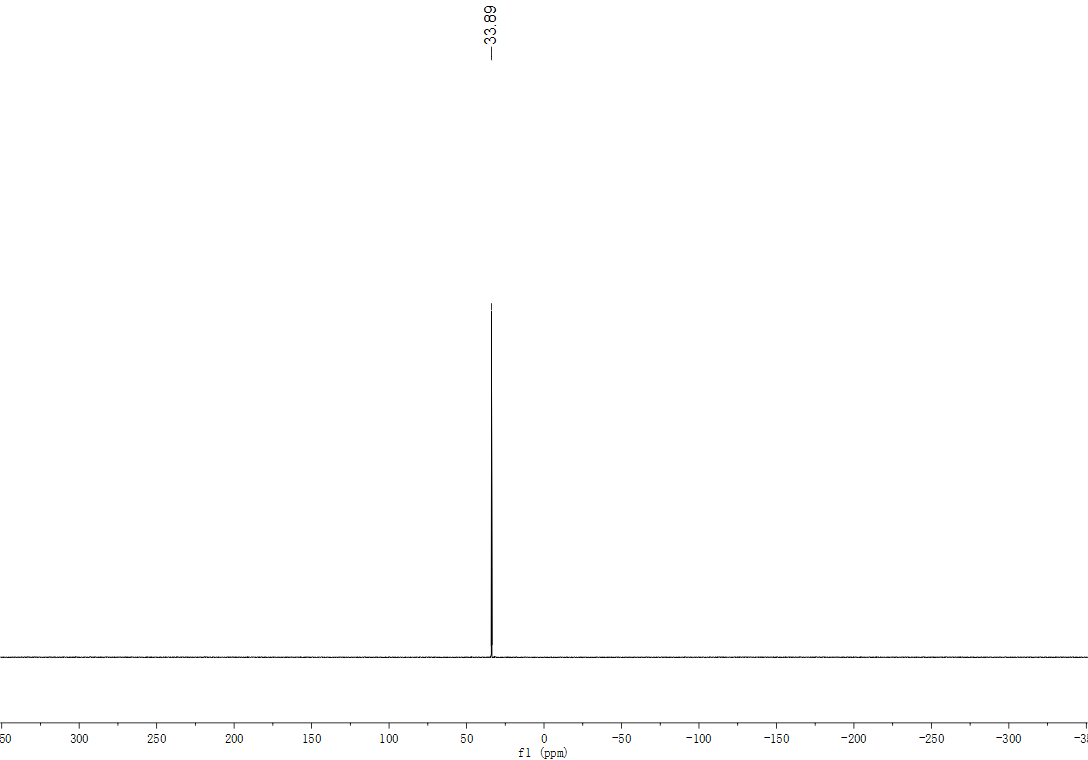
^

^31^P NMR (162 MHz, CDCl_3_) spectrum of **6b**

**
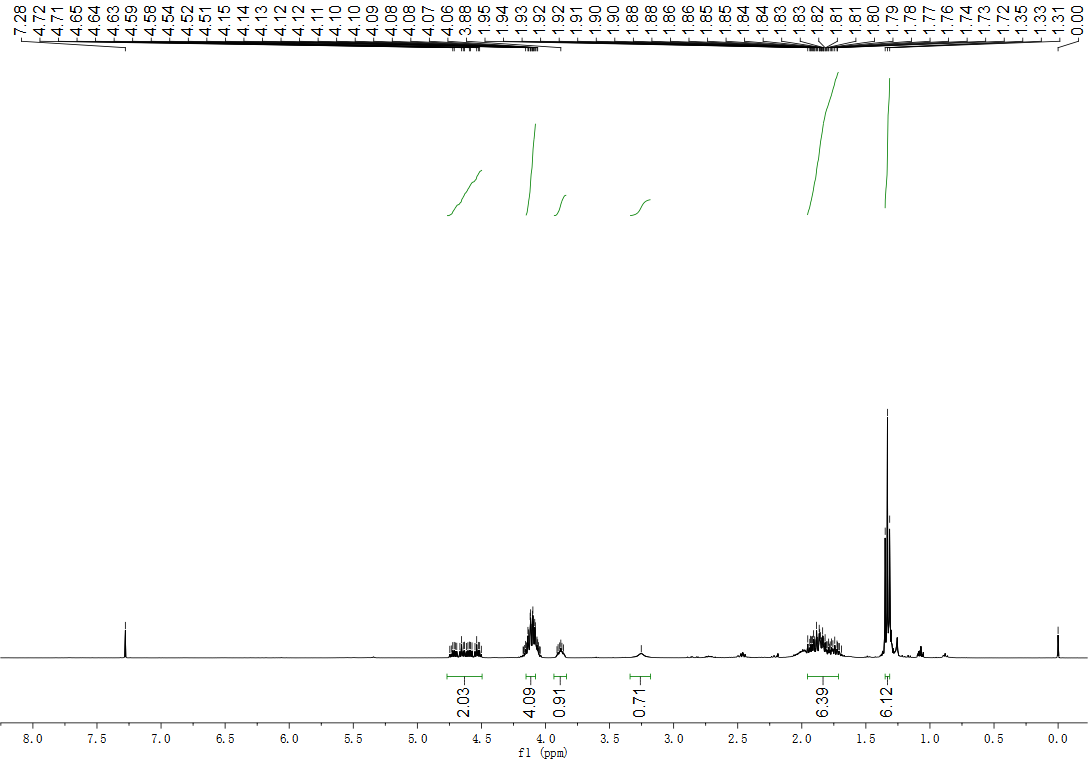
**

^1^H NMR (400 MHz, CDCl_3_) spectrum of **6c**

^
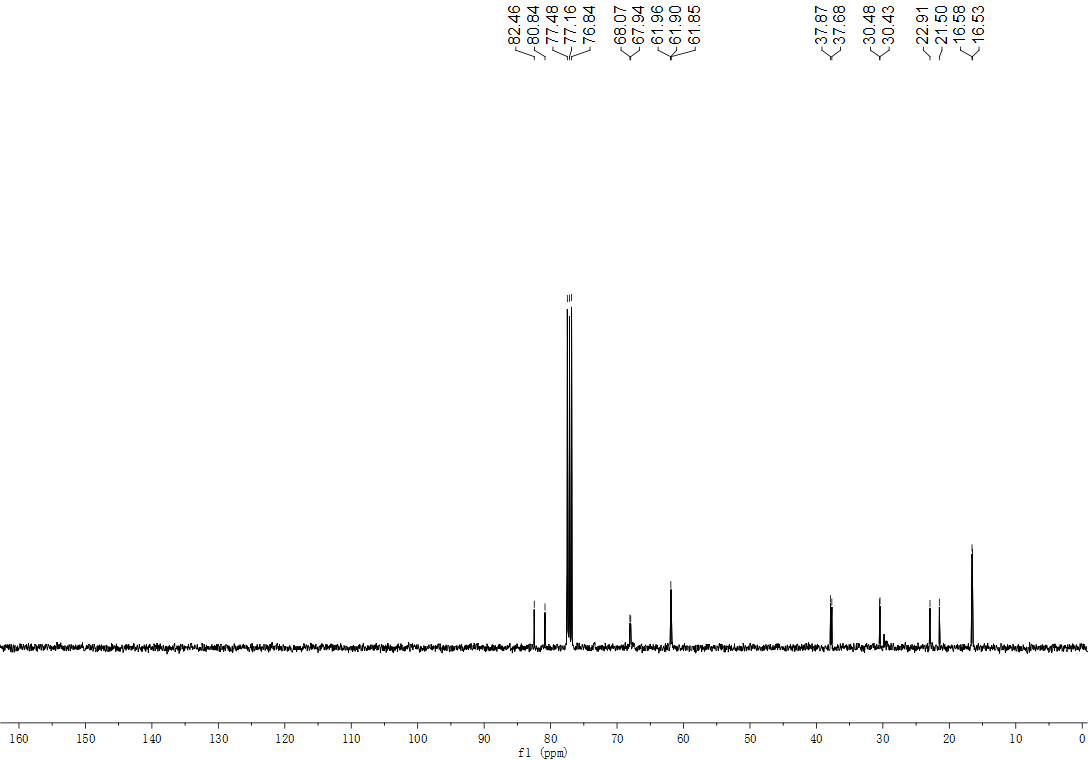
^

^13^C{^1^H} NMR (101 MHz, CDCl_3_) spectrum of **6c**

^
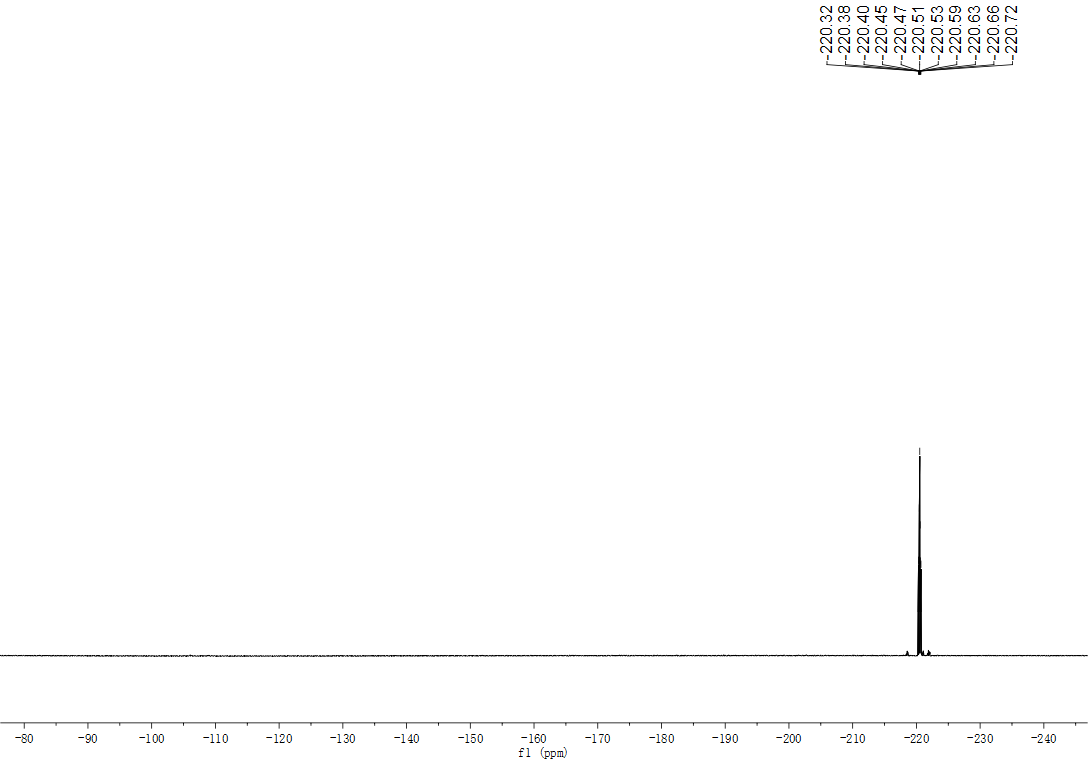
^

^19^F NMR (376 MHz, CDCl_3_) spectrum of **6c**

^
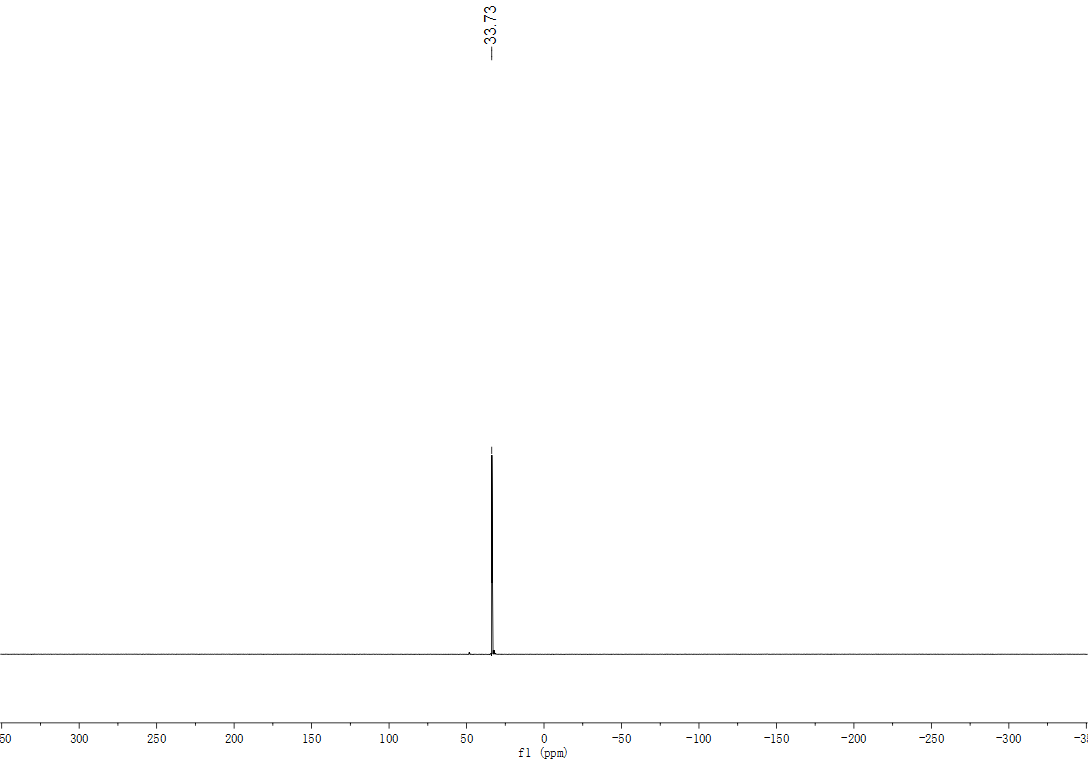
^

^31^P NMR (162 MHz, CDCl_3_) spectrum of **6c**
